# A Novel Parvovirus Associated with the Whitefly *Bemisia tabaci*

**DOI:** 10.3390/pathogens14070714

**Published:** 2025-07-19

**Authors:** Fani Gousi, Zineb Belabess, Nathalie Laboureau, Michel Peterschmitt, Mikhail M. Pooggin

**Affiliations:** 1 PHIM Plant Health Institute, University of Montpellier, INRAE, CIRAD, IRD, Institute Agro, 34398 Montpellier, France; fgousi@gmail.com (F.G.); nathalie.laboureau@cirad.fr (N.L.); michel.peterschmitt@cirad.fr (M.P.); 2 Plant Protection Laboratory, Regional Center of Agricultural Research of Meknes, National Institute of Agricultural Research, Meknes 50000, Morocco; zineb.belabess@inra.ma

**Keywords:** whitefly, *Bemisia tabaci*, parvoviridae, densovirus, homotelomeric, ambisense, episomal, endogenous viral elements, neofunctionalization

## Abstract

The whitefly *Bemisia tabaci* (Hemiptera: Aleyrodoidea) causes direct feeding damage to crop plants and transmits pathogenic plant viruses, thereby threatening global food security. Although whitefly-infecting RNA viruses are known and proposed as biocontrol agents, no insect DNA virus has been found in any member of Aleyrodoidea. Using rolling circle amplification (RCA) of viral DNA from whiteflies collected from crop fields in Morocco, followed by Illumina sequencing of the RCA products, we found a novel insect single-stranded (ss) DNA parvovirus (family *Parvoviridae)* in addition to plant ssDNA geminiviruses transmitted by whiteflies. Based on its genome organization with inverted terminal repeats and evolutionarily conserved proteins mediating viral DNA replication (NS1/Rep) and encapsidation (VP), encoded on the forward and reverse strands, respectively, we named this virus *Bemisia tabaci* ambidensovirus (BtaDV) and classified it as a founding member of a new genus within the subfamily *Densovirinae.* This subfamily also contains three distinct genera of ambisense densoviruses of other hemipteran insects (Aphidoidea, Coccoidea, and Psylloidea). Furthermore, we provide evidence for the genetic variants of BtaDV circulating in whitefly populations and for its partial sequences integrated into the *B. tabaci* genome, with one integrant locus potentially expressing a fusion protein composed of viral Rep endonuclease and host DNA-binding domains. This suggests a long-term virus-host interaction and neofunctionalization of BtaDV-derived endogenous viral elements.

## 1. Introduction

The family *Parvoviridae* comprises small non-enveloped viruses with linear single-stranded DNA (ssDNA) genomes ranging from 3.9 to 6.3 Kb. These viral genomes encode, on their left and right halves, respectively, non-structural (NS) proteins, including a replication-initiator protein (Rep/NS1) that mediates viral DNA replication, and structural/virion proteins (VPs) that encapsidate viral DNA into icosahedral particles. The protein-coding region is flanked at the 5′ and 3′-termini by telomeres of 100 to 550 nucleotides (nts), containing imperfect terminal palindromes folding into either similar (homotelomeric) or different (heterotelomeric) hairpin structures. These telomeres play essential roles in both the replication and encapsidation of viral genomic DNA. Upon release from the virion, viral ssDNA is converted to a double-stranded form by the host DNA polymerase, using the 3′-terminal hairpin as a primer, followed by end joining by the host DNA ligase. The resulting covalently closed duplex serves as a template for both transcription by host RNA Polymerase II (Pol II), producing viral mRNAs, and rolling-hairpin replication, initiated by the viral Rep protein. The Rep protein binds to the 3′-telomere (or to either telomere in the case of homotelomeric viruses), introduces a nick in the duplex just downstream of the hairpin sequence, and thereby establishes a replication fork. The freed 3′- end of the hairpin serves as a primer for DNA synthesis by the host DNA polymerase, while Rep remains associated with the 5′- end of the displaced strand, promoting replication through its helicase activity. The sequential unfolding and refolding of hairpin termini reverses the direction of DNA synthesis, allowing replication to proceed back and forth along the genome strands. After each round of bidirectional rolling-hairpin replication, progeny ssDNA genomes are excised by the viral Rep protein. Telomeres contain cis-acting packaging signals that ensure that only complete genomes are encapsidated. Homotelomeric parvoviruses package both forward and reverse strands of the genome in separate virions, whereas heterotelomeric parvoviruses package only one strand ([1,2]; https://ictv.global/report/chapter/parvoviridae/parvoviridae; accessed on 1 April 2025).

Based on the phylogenetic analysis of the NS1/Rep protein, parvovirids are currently classified into three subfamilies: (i) *Parvovirinae*, which infect vertebrates such as mammals, birds and reptiles, (ii) *Densovirinae*, which infect invertebrates such as insects, crustacean and echinoderms, and (iii) *Hamaparvovirinae*, which infect both vertebrates and invertebrates ([3]; https://ictv.global/taxonomy/taxondetails?taxnode_id=202404215&taxon_name=Parvoviridae; accessed on 1 April 2025). The subfamily *Densovirinae* includes several genera of homotelomeric ambisense densoviruses (ambidensoviruses), which encode NS and VP proteins on opposite strands and utilize a bidirectional transcription strategy with converging transcriptional units, in which transcription is regulated by the 5′-terminal rightward/NS and 3′-terminal leftward/VP promoters and the NS and VP terminators that overlap in the center of the genome. In contrast, the other two subfamilies contain only monosense parvoviruses [1,3].

The whitefly *Bemisia tabaci* (order Hemiptera, superfamily Aleyrodoidea) is an agriculturally important polyphagous pest that damages crop plants worldwide both through direct feeding and by transmitting pathogenic plant viruses. Previous studies have identified a diversity of insect RNA viruses hosted by *B. tabaci*, some of which have been evaluated as potential biocontrol agents [4]. However, to date, no parvovirus or other insect DNA viruses have been reported to infect *B. tabaci* or any other member of the Aleyrodoidea. Nonetheless, endogenous viral elements related to large double-stranded DNA viruses of the family *Nudiviridae* have been identified in the *B. tabaci* genome [5]. Among other hemipteran insects, only aphids (Aphidoidea), mealybugs (Coccoidea), and psyllids (Psylloidea) have been found to host parvoviruses. All these viruses possess homotelomeric ambisense genomes and fall into three distinct genera of the subfamily *Densovirinae*: the genus *Hemiambidensovirus*, which includes ambidensoviruses of aphids (*Dysaphis plantaginea*, *Myzus persicae*, and *Sitobion miscanthi*), the genus *Scindoambidensovirus*, which contains an ambidensovirus of the mealybug *Planococcus citri*, and the genus *Diciambidensovirus*, which includes ambidensoviruses of psyllids (*Diaphorina citri* and *Bactericera trigonica*) ([3]; https://ictv.global/taxonomy; accessed on 1 April 2025). In this study, we identified and sequenced the complete episomal genome of a homotelomeric ambisense parvovirus associated with the whitefly *B. tabaci*. Based on its genome organization and phylogenetic analysis of its NS/Rep and VP proteins, we classified this virus as a novel species representing a putative new genus within the subfamily *Densovirinae*, which is most closely related to the genus *Diciambidensovirus*. In addition to the episomal form, we found partial genome sequences of this virus integrated into the *B. tabaci* nuclear genome and potentially expressed from the genome into a viral Rep endonuclease-host DNA binding domain fusion protein, suggesting a long-term virus-host interaction and neofunctionalization of BtaDV-derived endogenous viral elements.

## 2. Results and Discussion

### 2.1. Discovery, Complete Genome Assembly, and Phylogenomic Analysis of a Novel Whitefly Parvovirus

#### 2.1.1. Illumina Sequencing Analysis of Whiteflies Sampled in Crop Fields of Morocco Reveals a Novel Parvovirus

As part of a collaborative project investigating the prevalence and genetic diversity of invasive geminiviruses transmitted by insect vectors in the Mediterranean region, we collected samples of the whitefly *B. tabaci* from tomato and cucurbit crop fields in the Berkane region of Morocco in 2020. Total DNA was extracted from pools of 10 whiteflies per field and subjected to rolling circle amplification (RCA) to enrich the circular viral DNA. The RCA products were then pooled and sequenced using Illumina technology, followed by de novo assembly of the 150 nt paired-end reads (see Materials and Methods section). BlastN analysis of the resulting contigs assembled from one of the Illumina-seq libraries (ALYU-390) revealed, in addition to contigs representing the genomes of *B. tabaci*, its endosymbionts (Portiera and Rickettsia) and three geminiviruses (tomato yellow leaf curl virus, tomato yellow leaf curl Sardinia virus and tomato yellow leaf curl New Delhi virus) (Table S1), a 4968 nt contig containing a 230 nt internal sequence with 68–71% identities to different members of *Parvoviridae.* Other sequences of this contig did not match any sequences in the NCBI GenBank database using BlastN, which finds somewhat similar sequences. Further analysis using BlastX revealed that this contig contained open reading frames (ORFs) encoding proteins with conserved parvoviridae domains. One ORF encoded a protein with both Phospholipase A2-like and Denso_VP4 domains, while another ORF encoded a protein with a Parvo_NS1 domain (Dataset S1A). These findings suggest that the contig represents a novel parvovirus species.

#### 2.1.2. Assembly and Sequence Analysis of a Complete Parvovirus Genome Reveals Its Homotelomeric Ambisense Nature

**Figure 1:**
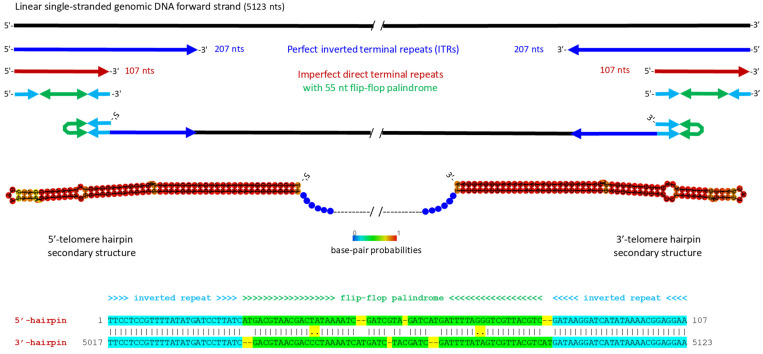
Structures of the 5′- and 3′-inverted terminal repeats (ITRs) of Bemisia tabaci ambidensovirus (BtaDV), which form symmetrical I-shaped hairpin telomeres. The 5123 nt linear single-stranded genomic DNA forward strand is represented by a solid black line. Perfect inverted repeats of the 5′- and 3′-ITR sequences of 207 nts are indicated by blue arrows. The 5′- and 3′-terminal regions of 107 nts represent imperfect direct repeats (shown as red arrows), which differ by an internal inversion of a 55 nt flip-flop palindrome and form symmetrical hairpins (shown schematically as connected cyan-green-cyan arrows, where the flip-flop palindrome is green). Secondary structures of the 5′ and 3′-hairpin telomeres, predicted using Matthews model’s DNA parameters at the RNAfold Webserver http://rna.tbi.univie.ac.at/cgi-bin/RNAWebSuite/RNAfold.cgi (accessed on 23 May 2025), are depicted both schematically and as images exported from the Webserver. The color code indicates base pair probabilities ranging from 0 (blue) to 1 (red). The annotated alignment of the 5′- and 3′-hairpin sequences is shown below.

The termini of the initial parvovirus contig contained a 120 nt inverted repeat with an additional unique 19 nt extension at one end (Dataset S1A). Secondary structure prediction did not reveal terminal hairpins, which are typically found in all parvoviral telomeres, suggesting that the termini were incomplete. To reconstruct the full-length genome, we mapped the Illumina 150 nt reads to the contig termini and retrieved the mapped reads containing overhanging sequences. Manual extension of the terminal regions, followed by verification of the complete genome sequence through read mapping (see Materials and Methods), allowed us to assemble a full-length consensus genome sequence. This genome contained perfect inverted terminal repeats (ITRs) of 207 nts, forming symmetrical 5′- and 3-terminal I-shaped hairpin structures. The hairpin-forming sequences are imperfect direct repeats of 107 nts, which differ by an internal 55 nt sequence inversion (a so-called “flip-flop” palindrome), making their secondary structures symmetrical (Figure 1; Dataset S1B). This structural configuration, with long ITRs and symmetrical hairpins, is characteristic of homotelomeric parvoviruses, which are currently classified into ten genera. These include the genus *Brevihamaparvovirus* of the subfamily *Hamaparvovirinae*, the *Dependoparvovirus* and *Erythroparvovirus* genera of the subfamily *Parvovirinae*, and seven out of eight genera of the subfamily *Densovirinae*, specifically those with an ambisense genome organization ([3]; ICTV *Parvoviridae* Study Group’s taxonomic proposal 2022: https://ictv.global/taxonomy/taxondetails?taxnode_id=202404215&taxon_name=Parvoviridae; accessed on 1 April 2025).

Interestingly, we identified non-B form DNA G-quadruplex elements in both 5′- and 3′-telomeres, located downstream and upstream of the hairpin-forming sequences, respectively (Dataset S1B). G-quadruplexes have also been identified in the telomeres of other parvoviruses [2], suggesting their potential roles in viral replication or packaging.

**Figure 2:**
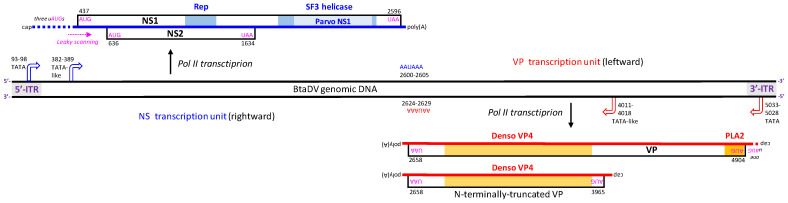
Genome organization and transcriptional map of Bemisia tabaci ambidensovirus (BtaDV). Viral genomic (forward and reverse) ssDNA molecules encapsidated in separate virions are depicted as black lines, with inverted terminal repeats (ITRs) shown as gray boxes. Predicted transcription start sites of the rightward non-structural (NS) and leftward virion protein (VP) genes are shown with blue and red bent arrows, respectively, with the positions of the respective upstream TATA-box or TATA-like promoter elements indicated. Viral mRNAs transcribed from the NS/rightward and VP/leftward units are depicted as blue and red lines, respectively, with the positions of their 5′-cap and poly(A) sites and ORFs’ start and stop codons indicated. The encoded proteins are named with their evolutionarily conserved domains colored within ORFs. The pink arrow indicates ribosome leaky scanning that would allow for the translation of NS1 and NS2 ORFs.

Analysis of the complete viral genome sequence using the NCBI ORF Finder and Protein Blast revealed three main ORFs encoding distant homologs of parvoviridae NS and VP proteins (Figure 2; Dataset S1C). A large ORF on the reverse strand, located in the 3′-half of the genome, encodes a VP/capsid protein of 749 amino acids that contains two conserved domains characteristic of *Densovirinae* capsid proteins: a Phospholipase A2-like domain (PLA2; amino acids 5–45) and a Denso_VP4/capsid protein domain (amino acids 342–669), both identified using InterPro [6] (Figure 2 and Figure S1). Two ORFs on the forward strand, located in the 5′-half of the genome, encode an NS1/Rep protein of 719 amino acids (larger ORF), which contains an SF3 Helicase 1 domain (amino acids 447–652) with an internal Parvo NS1 domain (amino acids 507–617), both identified using InterPro (Figure S1), and an NS2 protein of 332 amino acids (smaller internal ORF), showing distant homology to parvovirid NS proteins, including the NS2 proteins of ambisense densoviruses (Dataset S1C). Inspection of the NS1 protein sequence revealed two conserved motifs likely representing a HuH endonuclease/nickase domain, HuH (HVH; amino acids 256–258) and YuxK (YIQK; amino acids 307–310) [7], both of which are conserved in NS1/Rep proteins of most closely related ambidensoviruses (Dataset S1C).

Further analysis of the viral genome sequence revealed cis-acting elements likely involved in Pol II-mediated transcription of the NS and VP genes (Figure 2). In the NS transcription unit, a TATA-like promoter element was identified at nucleotide positions 382–389 (TAAATAAA), with a predicted transcription start site (positions 414–416) located just upstream of the NS1 ORF start codon (AUG at positions 437–439). A canonical TATA-box (TATAAA), located in the 5′-ITR at positions 93–98, can also initiate Pol II transcription. However, three upstream AUG codons (uAUGs) and structured GC-rich sequences, including the G-quadruplex region (Dataset S1B), can impede ribosome scanning and reduce the efficiency of translation initiation at the NS1 start codon. Since no AUG occurs between the start codons of the NS1 ORF and the NS2 ORF (AUG at positions 636–638), NS2 translation is likely initiated via ribosomal leaky scanning through the NS1 start codon (Figure 2). A canonical polyadenylation signal (AAUAAA) was identified at positions 2600–2605, just downstream of the NS1 ORF stop codon (UAA at positions 2594–2596).

In the VP transcription unit, a canonical TATA-box (TATAAA) was identified in the 3′-ITR at positions 5023–5028, located 124 nts upstream of the VP ORF start codon (AUG, positions 4902–4904). However, one intervening AUG codon and a GC-rich sequence (Dataset S1B) are present between them, which can hinder ribosome scanning and translation initiation. As no closer TATA-like motif could be identified upstream of the VP ORF start codon, we hypothesized that Pol II transcription might instead be initiated from non-TATA promoter elements. Interestingly, the same TATA-like motif (TAAATAAA) found upstream of the NS1 ORF is also present within the VP ORF at positions 4011–4018. If this motif serves as part of a functional core promoter, transcription from this site could produce an mRNA that would be translated from the first downstream AUG codon into an N-terminally truncated VP protein of 435 amino acids. This truncated protein retains the Denso_VP4 capsid protein domain but lacks the N-terminal PLA2 domain (Figure 2). We further hypothesized that both full-length and truncated VP might participate in viral DNA encapsidation and virion formation. In such virions, the PLA2 domain of the full-length protein might be exposed on the virion surface, enabling clathrin-mediated endocytosis and subsequent nuclear trafficking, as previously demonstrated for a lepidopteran ambisense densovirus [8].

Taken together, the genomic and transcriptional features classify the newly identified virus as a homotelomeric ambisense member of the subfamily *Densovirinae*. It possesses a rightward NS transcription unit for the expression of the non-structural proteins NS1/Rep and NS2, which likely mediate rolling-hairpin replication of viral DNA, and a converging leftward VP unit for the expression of the virion protein VP with conserved PLA2 and Denso_VP4 domains and its shorter version lacking PLA2, which likely mediates viral DNA encapsidation. Therefore, we named this novel virus Bemisia tabaci ambidensovirus (BtaDV) and deposited its complete genome sequence in the NCBI GenBank under the accession number PV695490.

#### 2.1.3. Phylogenetic Analysis of NS1/Rep and Other Viral Proteins, Along with Their Expression Strategies, Classify BtaDV into a Putative New Genus Within the Densovirinae

**Figure 3:**
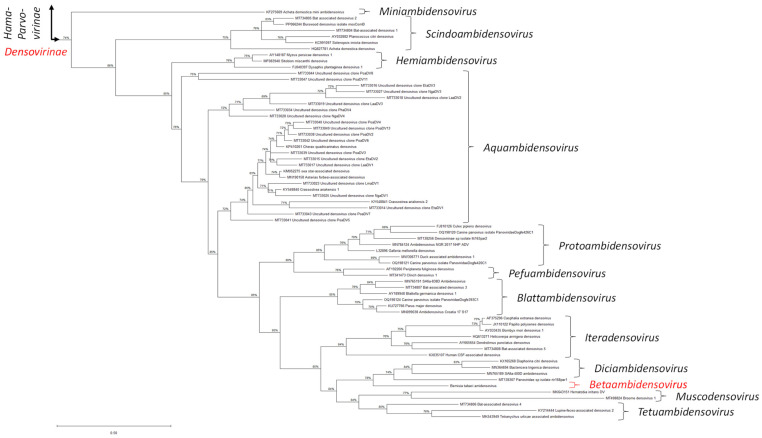
Maximum likelihood (ML) phylogenetic tree based on the Parvo NS1 domain of Bemisia tabaci ambidensovirus (BtaDV) and all currently classified members of the family *Parvoviridae*. To construct the tree, the protein sequences were aligned using MUSCLE. The alignment was then analyzed using the default ML parameters of MEGA v. 12.0.11. Part of the tree representing the subfamily *Densovirinae* is shown, with all currently established genera indicated with brackets and respective names, and the position of BtaDV and its tentative new genus, *Betaambidensovirus*, indicated in red. The lengths of the tree branches indicate the evolutionary distance between sequences, measured in amino acid substitutions per site (the scale bar of 0.50 substitutions is shown below the tree on the left). The complete tree is presented in Figure S2B.

Following the current classification criteria set by the ICTV *Parvoviridae* Study Group, we performed a comparative phylogenetic analysis of the conserved Parvo NS1 domain of NS1/Rep proteins encoded by BtaDV and all officially recognized species of the family *Parvoviridae* as of 2022 (ICTV *Parvoviridae* Taxonomic Proposal 2022: https://ictv.global/taxonomy/taxondetails?taxnode_id=202404215&taxon_name=Parvoviridae; accessed on 1 April 2025). Maximum likelihood (ML) phylogenetic trees constructed using both MEGA and BEAST software tools revealed that BtaDV is most closely related to homotelomeric ambidensoviruses currently classified within the genus *Diciambidensovirus.* This genus includes Diaphorina citri densovirus (DcDNV) and Bactericera trigonica densovirus (DtDNV), which infect the hemipteran insects *D. citri* and *B. trigonica* (Psylloidea), as well as SAfia-400D ambidensovirus, which was identified in the blood virome of Tanzanian children (Figure 3 and Figure S2).

BtaDV is also, although more distantly, related to homotelomeric ambidensoviruses classified within two additional genera of the subfamily *Densovirinae*, which form a neighboring clade in the ML phylogenetic tree (Figure 3): the genus *Muscodensovirus*, which includes Broome densovirus 1 and Hematobia irritans densovirus, found in the viromes of dipteran insects *Culex annulirostris* (mosquito) and *Hematobia irritans* (horn fly), respectively, and the genus *Tetuambidensovirus*, which includes an acarian mite *Tetranychus urticae*-associated densovirus and two densoviruses detected in mammalian feces: Bat-associated densovirus 4 and Lupin feces-associated densovirus 2.

Comparative pairwise sequence analysis of the complete NS1/Rep proteins of BtaDV and two psyllid-infecting diciambidensoviruses, DcDNV and BtDNV, revealed that the NS1 proteins of diciambidensoviruses share higher identity (42.2% identity, 55.7% similarity, 12.3% gaps) with each other than with the BtaDV NS1, which shows comparably low identity with both BtDNV and DcDNV (26.7% identity, 41.8% similarity, 26.5% gaps; Dataset S1D). Moreover, we found that the genome organization of BtaDV differs substantially from that of diciambidensoviruses. Unlike BtaDV, both BtDNV and DcDNV encode the VP domains PLA2 and Denso_VP4 on two separate ORFs (Dataset S1D) and both initiate the NS1 ORF at non-AUG start codons: UUG in DcDNV [9,10] and CUG in BtDNV [11] (Dataset S1D). Similar to NS1 proteins, the VP proteins of DcDNV and BtDNV were found to be much more similar to each other than to the VP of BtaDV (Dataset S1D).

Taken together, we propose to classify BtaDV as the founding member of a new genus, which we suggest naming *Betaambidensovirus* (where *Be* stands for *Bemisia* and *ta* for *tabaci*). Following binomial nomenclature, we propose naming the BtaDV species *Betaambidensovirus hemiptreran*1.

Interestingly, an unclassified parvovirus identified in an anal swab of the bird *Luscinia sibilans* (accession MT138307) appeared between BtaDV and members of the genus *Diciambidensovirus* in the ML tree based on the Parvo NS1 domain (Figure 3), suggesting that it could be a potential member of either *Diciambidensovirus* or *Betaambidensovirus* (Figure S2A). However, the complete NS1/Rep protein encoded by this virus shares a very low sequence identity with the complete NS1/Rep protein of BtaDV (25.0% identity, 38.4% similarity, and 29.1% gaps) (Dataset S1D).

### 2.2. Presence of BtaDV in Different Whitefly Field Populations and Evidence for Integration of Its Partial Sequences in the B. tabaci Genome

#### 2.2.1. RCA Illumina Sequencing Analysis of the Pooled Whiteflies from Crop Fields Provides Evidence for BtaDV and Its Genetic Variants Circulating in Morocco

Mapping of Illumina-seq reads to the BtaDV reference sequence revealed that, in addition to the library ALYU-390, from which the complete BtaDV genome was de novo assembled, BtaDV-specific reads were present in other libraries (Table S1). In the library ALYU-381, the BtaDV genome sequence was fully covered by reads (ranging from 2 to 25 reads per nucleotide), with single-nucleotide polymorphisms (SNPs) distributed along the genome (Figure S3B), suggesting the presence of distinct genetic variants of BtaDV. In contrast, the libraries ALYU-380 and ALYU-388 contained significantly fewer reads, covering approximately two-thirds and one-third of the genome, respectively, and also contained SNPs (Figure S3B). Other libraries contained either no virus-specific reads (ALYU-382, 385) or only a few (ALYU-379, 383, 384, 386, 387, and 389), indicating that BtaDV was either absent or present at levels below (or close to) those expected from cross-contamination (Figure S3B).

A consensus genome sequence reconstructed from the ALYU-381 reads, which represented a major but not the only genetic variant of BtaDV in the ALYU-381 pool of whiteflies (Figure S3C), differed from the ALYU-390 genome sequence by two one-nucleotide indels located between the 5′-hairpin sequence and the NS1 ORF, and several single-nucleotide substitutions in both non-coding and coding sequences (Dataset S1B). These alterations did not affect any of the cis-elements or protein domains/motifs described above.

Taken together, these findings suggest that BtaDV and its genetic variants are present in different whitefly populations in the Berkane province of Morocco.

#### 2.2.2. Diagnostic PCR Analysis of BtaDV in Different Field Populations of *B. tabaci* Whiteflies Supports Its Prevalence in Morocco

Since BtaDV was identified by Illumina sequencing of the pooled RCA products obtained from pooled whitefly samples collected from different crop fields, we designed a diagnostic PCR using primers specific to the BtaDV VP gene to determine which fields were infested with BtaDV-carrying whiteflies.

**Figure 4:**
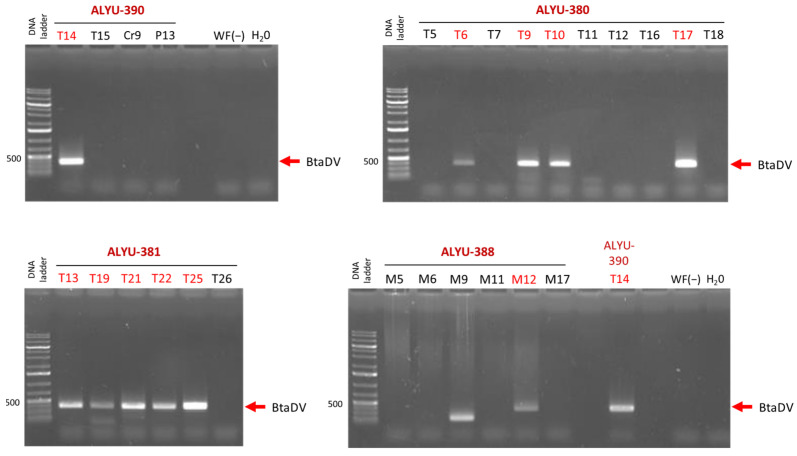
Diagnostic PCR analysis for the presence of BtaDV in individual RCA products was pooled into ALYU pools for Illumina sequencing. The individual RCA products corresponding to each of the four pools positive for virus-specific Illumina reads (ALYU-390, ALYU-380, ALYU-381, and ALYU-388) were used as templates for PCR with BtaDV VP gene-specific primers. The PCR products were analyzed by 1% agarose gel electrophoresis, followed by staining with ethidium bromide. Images of the four stained gels corresponding to each of the ALYU pools, composed of RCA products obtained from whiteflies collected from different crop fields, are shown, with field names indicated (T for tomato, Cr for pumpkin, P for watermelon, and M for melon). Water (H_2_O) and total DNA extracted from BtaDV-free whiteflies collected in France (WF(−)) were used as the negative controls. The position of the BtaDV-specific PCR product of the expected size (372 bp) is indicated by red arrows. The 1 Kb Plus DNA ladder was used as a size marker.

For the ALYU-390 pool, PCR analysis of the individual RCA products revealed that only the tomato field T14 tested positive for BtaDV, while other fields (tomato T15, zucchini Cr9, and watermelon P13) were PCR-negative (Figure 4; Table S1). For the ALYU-381 and ALYU-380 pools, which were fully composed of whitefly samples from tomato fields, five of the six fields and four of the ten fields, respectively, were positive for BtaDV. For the ALYU-388 pool, composed of whitefly samples from melon fields, one of the six fields (M12) was positive for BtaDV, while the other fields were either PCR negative or yielded a PCR product of smaller-than-expected size (M9) (Figure 4; Table S1).

Taken together, our findings indicate that BtaDV is prevalent in the whitefly populations collected from tomato fields and is also present in at least one of the melon fields. We found no clear correlation between the detection of BtaDV and the presence of plant geminiviruses in whitefly pools (Table S1). Nonetheless, the effects of BtaDV on whitefly development and transmission of plant viruses remain to be further investigated. Likewise, further in-depth analysis of the whitefly samples that tested positive for BtaDV should shed more light on the stability of its genome sequence.

#### 2.2.3. Evidence for Integration and Neofunctionalization of Partial BtaDV Sequences in the Genome of *B. tabaci*

BlastX analysis of BtaDV revealed that the N-terminal half of its NS1 protein (amino acids 1–338) shares 67% identity and 85% similarity with the N-terminal region (amino acids 1–338) of an unnamed 486 amino-acid protein encoded by the genome of the *B. tabaci* Q-type from Almeria, Spain, a biotype well distributed in the Mediterranean and elsewhere in the world (Dataset S1E).

**Figure 5:**
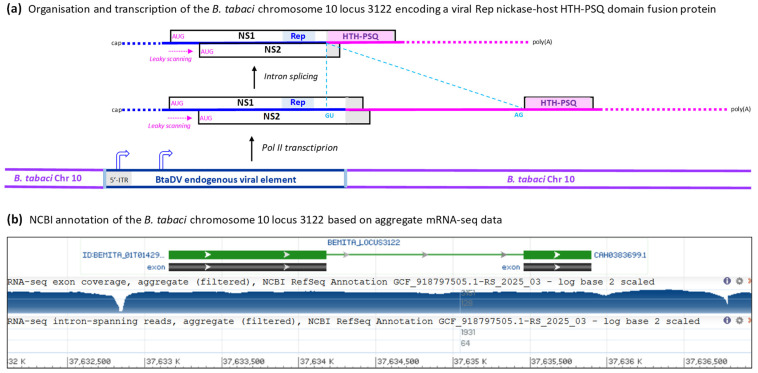
Organization and transcription of the genomic locus of the *B. tabaci* Q-type Almeria chromosome 10, which encodes a fusion protein containing the viral NS1/Rep endonuclease domain and the host DNA-binding HTH-PSQ domain. (**a**) Scheme of the genomic locus 3122 of the *B. tabaci* chromosome 10, which contains the partial BtaDV genome sequence, spanning a nearly complete 5′-ITR (lacking the first 41 nts) and half of the NS1/NS2 transcription unit (ending 34 nts upstream of the NS2 stop codon), flanked by the host genome sequences. The spliced and unspliced mRNAs transcribed by Pol II from this locus are depicted as solid color-coded lines (blue for the viral sequence, pink for the host sequence), with the main ORFs boxed, positions of the splice donor (GU) and acceptor (AG) indicated, and the functional protein domains (viral Rep and host HTH-PSQ) highlighted within ORFs. Ribosome leaky scanning, which allows translation initiation at both NS1 and NS2 AUG start codons, is indicated by pink dotted arrows. The positions of the viral promoters potentially driving Pol II transcription are indicated by bent arrows. Dotted termini of the mRNAs indicate potential transcription initiation and termination events both at the most distal TATA-like promoter and terminator elements (supported by the aggregate mRNA-seq data shown in panel B) and at the TATA-like promoter located just upstream of the NS1 ORF and the terminator located just downstream of the HTH-PSQ ORF. (**b**) Screenshot image of the NCBI genome browser showing features of the genomic locus 3122 of the *B. tabaci* chromosome 10, annotated based on the aggregate RNA-seq data, with the exons corresponding to the viral NS1/Rep and the host HTH-PSQ coding sequences boxed and the intron shown as a line connecting the exon boxes. A log-scale map of the aggregate mRNA-seq reads is shown below, with nucleotide positions of the chromosome 10 scaffold (NC_092802) indicated.

Analysis of the genomic locus on the *B. tabaci* Q-type Almeria chromosome 10 (NC_092802; BioProject: PRJEB47898), which encodes this protein, revealed a 1.6 Kb sequence showing 75% pairwise nucleotide identity with the 5′-portion of the BtaDV genome (positions 41–1597). This sequence spans a nearly complete 5′-ITR (lacking the first 40 nts) and approximately half of the NS1/NS2 transcription unit, ending 34 nts upstream of the NS2 ORF stop codon (Figure 5; Dataset S1E). Interestingly, the incomplete 5′-ITR sequence begins with a near-complete flip-flop palindrome of 41 nts, corresponding to the respective sequence within the 55 nt flip-flop palindrome in the BtaDV 5′-ITR and differing by only a single-nucleotide substitution in the hairpin loop (Dataset S1E). This finding was consistent with our Illumina-seq assembly of the BtaDV 5′-ITR and further enabled the reconstruction of a complete 5′-hairpin sequence of the putative BtaDV genetic variant, whose partial sequence was integrated into the *B. tabaci* genome. The reconstructed sequence folded into a secondary structure nearly identical to the 5′-hairpin of episomal BtaDV (Dataset S1E). Furthermore, the integrated BtaDV sequence retained the AUG start codons of both NS1 and NS2 ORFs and the intact TATA-like promoter element, while its 5′-ITR-based TATA-box sequence was altered to resemble the downstream TATA-like sequence (Figure 5a; Dataset S1E).

The potential expression of the unnamed protein homologous to BtaDV NS1/Rep, encoded within the *B. tabaci* Q-type Almeria genome, is supported by aggregate Illumina mRNA-seq data [12], showing transcripts spanning the genomic locus, with a major transcription start site located within the viral 5′-ITR and a canonical GU-AG intron of 1271 nts spliced out (Figure 5a,b). The 5′-splice site (GU) is located within the integrated viral sequence at positions corresponding to 1467–1468 of episomal BtaDV (171 nt upstream of the NS2 stop codon), while the 3′-splice site (AG) lies within the downstream host sequence and is followed by an in-frame stop codon, resulting in a C-terminal extension of the viral protein sequence (Dataset S1E). The resulting NS1-host fusion protein contains the conserved HuH and YuxK motifs of the NS1/Rep endonuclease/nickase domain and may therefore retain DNA-nicking activity.

InterPro analysis of the 486-amino-acid fusion protein revealed that its host sequence-derived C-terminal region (amino acids 341–484) represented a helix-turn-helix (HTH) PSQ-type domain (Figure 5a; Dataset S1E). Proteins containing the HTH PSQ-type domain constitute a family of eukaryotic DNA-binding proteins, best characterized in Drosophila as the Pipsqueak (PSQ) family, which is implicated in transcriptional regulation, chromatin remodeling, and other pathways [13]. Thus, the fusion protein combining the viral DNA nickase domain and the host DNA-binding domain exemplifies the neofunctionalization of endogenous viral elements.

In addition to the 1.4 Kb endogenous viral element of BtaDV in chromosome 10 of the *B. tabaci* Q-type Almeria genome, its chromosomes 2, 4, 6, and 8 contain much shorter sequences sharing 70–72% identity with the NS1 protein-coding sequences of BtaDV. The longest of these on chromosome 6 spans positions 1125–1498 of the BtaDV genome, encoding the Rep nickase domain, while shorter sequences on other chromosomes map within the SF3 helicase domain (Dataset S1E).

Our further BlastN analysis of the BtaDV genome against the genome resources available in the NCBI for other representatives of the *B. tabaci* cryptic species complex from China, the USA, India, and Sub-Saharan Africa (a total of 15 genome assemblies) revealed that BtaDV-related endogenous viral elements (EVEs) were present in all of them (Dataset S1E). Most strikingly, an unannotated genome assembly of the *B. tabaci* MED cryptic species (male and female individuals sampled in China; BioProject: PRJNA553778) contains within its scaffold 10 (VMOE01000001.1) a 3.8 Kb locus nearly identical (99.7% identity, 0.2% gaps) to the locus of the *B. tabaci* Q-type Almeria chromosome 10, which encodes the viral Rep-host HTH-PSQ fusion protein. Two single-nucleotide substitutions and three short deletions (1 nt, 3 nts, and 4 nts) found in the non-coding sequences of this locus (Dataset S1E) are not expected to affect the potential transcription, splicing, or translation of the fusion protein. Because Q-type Almeria from Spain and MED from China likely belong to the same *B. tabaci* cryptic species MED (formerly named the Q biotype), we assumed that the fusion protein could potentially be expressed in both whitefly populations. In addition, a BtaDV-related EVE of 641 nts, located in the same scaffold 10 of MED, encodes a 215 amino-acid protein with a SF3 helicase/Parvo NS1 domain homologous to the respective domain of the BtaDV NS1 protein (Dataset S1E).

The genomes of other *B. tabaci* representatives from the USA (MEAM1), China (Q1, Asia1, AsiaII1, AsiaII6, and AsiaII7), India (AsiaII-5), and sub-Saharan African countries (SSA1 from Tanzania, SSA1-SG1, SSA2, and SSA3 from Nigeria, SSA1-SG1 and Sweetpotato from Uganda) also contain EVEs sharing homology with the NS1 protein-coding sequences of BtaDV. However, unlike Q-type Almeria and MED, they lack BtaDV-related 5′-ITRs. In further similarity to the genomes of Q-type Almeria from Spain and MED from China, no sequences related to the BtaDV VP gene were found in other genomes, with the notable exception of SSA1 from Tanzania and SSA1-SG, SSA2, and SSA3 from Nigeria. The genomes of these four representatives of *B. tabaci* contained short EVE sequences of up to 322 nts with less than 70% identity to positions 2830–3152 of BtaDV (Dataset S1E).

Remarkably, the SSA2 Nigeria genome contains a 33.5 Kb EVE with an array of NS1 Rep nickase domain-encoding sequences (29 complete head-to-tail copies of ca. 1.1 Kb) sharing ca. 75% pairwise identity with the respective 1.1 Kb region of BtaDV (Dataset S1E).

Taken together, these findings suggest that past infections of *B. tabaci* whitefly populations with BtaDV have resulted in the integration of partial viral sequences in the host genome, followed by an apparent adaptation of the viral NS1/Rep gene sequence in at least one lineage to enable expression of the N-terminal portion of the NS1/Rep protein, containing the HuH endonuclease domain, fused to the host HTH PSQ-type DNA-binding domain. Whether this fusion protein is indeed translated from the hybrid viral-host mRNA and, if so, whether it performs any function within the nuclei of whitefly cells remains to be investigated. Interestingly, endogenous viral elements with partial low homology to Myzus persicae densovirus (MpDV, genus *Hemiambidensovirus*) have been identified in the genome of *Myzus persicae* (Aphidoidea) and have been implicated in antiviral defenses that lead to persistent (rather than acute) infections of *M. persicae* aphids with episomal MpDV [14,15].

## 3. Conclusions

This study reports the discovery and complete genome characterization of a novel insect DNA virus, Bemisia tabaci ambidensovirus (BtaDV), isolated from *B. tabaci* whiteflies sampled in Morocco. BtaDV is the first ssDNA virus associated with any member of the Aleyrodoidea superfamily. Based on genomic organization, phylogenetic placement, and distinctive structural characteristics like homotelomeric ITRs and ambisense gene orientation, BtaDV represents a novel species within a tentative new genus of the subfamily *Densovirinae*. Phylogenetic and comparative sequence analyses showed that, although BtaDV is most closely related to diciambidensoviruses associated with other hemipterans (psyllids), it is genetically different, especially in its NS1/Rep protein sequence and genome architecture. These differences support its classification into a new genus, which we tentatively named *Betaambidensovirus*.

In addition, this study provides evidence for genetic variants of BtaDV circulating in field whitefly populations and identifies partial viral sequences integrated into the genomes of different representatives of the *B. tabaci* cryptic species complex. Most of these endogenous viral elements represent partial NS1 gene sequences that encode the Rep endonuclease/nickase domain. One of the BtaDV sequences integrated into the genome of the Mediterranean biotype (Q/MED) of *B. tabaci* appears to be adapted to enable the expression of the NS1/Rep nickase domain fused to the host DNA-binding domain. These findings suggest a long-term evolutionary relationship between BtaDV and its host, leading to neofunctionalization of endogenous viral elements and potentially impacting virus persistence, whitefly biology, and antiviral defenses. Future research should address these issues.

The identification of BtaDV paves the way for further understanding of insect-parvovirus co-evolution and raises the possibility of exploiting such exogenous or endogenous parvoviruses as biological control agents to control whiteflies, a major global agricultural pest. Further studies should aim to determine the prevalence, genetic diversity, functional impact, and transmission dynamics of BtaDV across *B. tabaci* populations and to explore its biocontrol potential.

## 4. Materials and Methods

### 4.1. Whitefly Sampling

During a survey for invasive geminiviruses in the northeastern Berkane province of Morocco in 2020, adult whiteflies (*B. tabaci*) were collected from several fields of cultivated tomatoes and cucurbits (zucchini, melon, watermelon, cucumber, and pumpkin), in which plants exhibiting geminivirus-like symptoms were observed. Whiteflies collected from each field or plot of a larger field (Table S1) were pooled in a single tube with 70% ethanol and stored until total DNA extraction.

### 4.2. Total Whitefly DNA Extraction

From each pool of whiteflies, ten individuals were taken using a pincette from the collection tube to a new tube containing 150 μL TNES buffer (50 mM Tris-HCl pH 7.5, 400 mM NaCl, 20 mM EDTA, 0.5% SDS) and were ground with a pestle. The resulting crude extract was mixed with 45 μL of cold 5 M NaCl, followed by centrifugation at 6500 rpm for 10 min at 4 °C. The supernatant was then mixed with 500 μL of cold absolute ethanol and incubated for 20 min at −80 °C, followed by centrifugation at 14,000 rpm for 20 min at 4 °C. The pellet was washed with 250 μL of 70% ethanol, followed by centrifugation at 14,000 rpm for 10 min at 4 °C. After discarding the supernatant, the pellet was dried, resuspended in 50 μL H_2_O, and stored at −20 °C. The concentration and purity of the total insect DNA were measured using a Nanodrop spectrophotometer.

### 4.3. Rolling Circle Amplification (RCA) of Viral DNA

Circular viral DNA components of total insect DNA were enriched by rolling circle amplification (RCA) using a TempliPhi RCA kit (GE Healthcare, Singapore) according to the manufacturer’s protocol. Briefly, 5 μL of sample buffer and 1.5 μL of total DNA extracted from the whiteflies were mixed and heated at 95 °C for 3 min. The samples were cooled and mixed with 5 μL reaction buffer and 0.2 μL enzyme mix, followed by incubation at 30 °C for 30 h. The enzyme was inactivated by heating at 65 °C for 10 min.

### 4.4. Restriction Analysis of RCA Products

The RCA products were digested with the restriction enzymes NcoI or XbaI, which were selected based on their unique cutting sites in the genomes of two geminiviruses infecting vegetable crops in Morocco and potentially present in the whitefly vector (tomato yellow leaf curl virus and tomato leaf curl New Delhi virus, respectively). Five microliters of each RCA product was mixed with 2 μL of rCutSmart™ NEBuffer (10×) and 1 μL (1 U/μL) of NcoI or XbaI in a final volume of 20 μL and incubated at 37 °C for 1 h. The digested and undigested RCA products were analyzed on 1% agarose gel.

### 4.5. Illumina Sequencing of RCA Products and De Novo Assembly

Based on the results of the restriction analysis, undigested RCA products were pooled into 12 pools (3–10 products per pool). The DNA of each pool was purified using the NucleoSpin Gel and PCR Clean-up kit (Macherey-Nagel, Allentown, PA, USA) following the manufacturer’s protocol. Fifty nanograms of the purified DNA were subjected to Illumina sequencing at Fasteris AG (www.fasteris.com (accessed on 1 April 2025)). Libraries were prepared using the Twist DNA-Seq method with enzymatic shearing, and the resulting DNA libraries (ALYU-379 to ALYU-391) were multiplexed and sequenced in a single S1 flowcell of the Illumina NovaSeq 6000, using a 2 × 150 bp paired-end run, yielding high-quality data (Q30 > 91%). Illumina reads were demultiplexed based on dual indexing.

### 4.6. De Novo Assembly of Illumina Sequencing Reads

To filter out host DNA, Illumina reads of each library were mapped with Burrows-Wheeler Aligner (BWA) [16] to a combined set of reference genomes, including *B. tabaci* (MEAM1), Candidatus *Hamiltonella defensa* (*B. tabaci* MED), Candidatus *Portiera aleyrodidarum* (*B. tabaci* MED), *Cardinium* endosymbiont (*B. tabaci*), *Rickettsia* sp. MEAM1 (*B. tabaci*), *Citrullus lanatus*, *Cucumis melo*, *Cucumis Sativus*, *Cucurbita moschata*, *Cucurbita pepo*, and *Solanum lycopersicum*, all retrieved from the NCBI Genome database. The unmapped reads, presumed to contain viral sequences, were extracted using SAMtools [17] and used for downstream de novo assembly. All steps in this pipeline were executed in a Bash environment using command-line tools and shell scripts.

Additionally, custom in-house scripts were employed to enrich highly redundant sequences (i.e., unique sequences represented by ≥10, ≥30, or ≥50 reads) to improve the assembly of small circular viral genomes.

De novo assemblies were performed using Velvet v. 1.2.10 assembler [18], in combination with VelvetOptimiser (https://github.com/tseemann/VelvetOptimiser (accessed on 1 May 2025). Multiple k-mer lengths in the range from 117 to 141, incremented by 4, were assessed, with N50 statistics used to identify the optimal assembly. Assemblies were run in parallel, with the expected coverage set to 10, coverage cutoff to 5, and minimum contig length threshold to 200 nts.

For each library, the Velvet contigs generated with all k-mers were combined and scaffolded using SeqMan Pro v. 7.1.0 (DNASTAR Lasergene, Madison, WI, USA). SeqMan contigs were analyzed using BlastN, and consensus viral genomes were verified by mapping raw reads back to the contigs using BWA and Minimap2 [19], followed by visualization using Integrative Genomics Viewer (IGV) v. 2.18.2 [20] and MISIS-2 [21], and manual curation when necessary.

### 4.7. Detection and Complete Consensus Genomic Reconstruction of the Novel Parvovirus

The novel parvovirus was identified as a single contig assembled from the library ALYU-390, which included four RCA products derived from whiteflies collected in two tomato fields, one pumpkin field, and one watermelon field (Table S1), one of which (T14) was PCR-positive for BtaDV. As described in the Results and Discussion section, the contig represented an incomplete viral genome, lacking the 5′- and 3′-terminal sequences of the ITRs. To reconstruct the full-length viral genome, raw Illumina reads were mapped to the contig sequence using Minimap2, followed by visualization using IGV. Reads aligned to the 5′- and 3′- ends of the contig with terminal overhangs were retrieved using a custom Bash script that combined SAMtools and AWK to filter reads by position and orientation. These reads were then used to manually extend the contig at both ends. The resulting complete genome was validated by re-mapping the raw reads with Minimap2 and verifying the consensus sequence using the IGV. The imperfect palindromic terminal repeats were supported by 10 to 189 reads per consensus nucleotide, with the lowest coverage observed at the extreme 5′- and 3′- ends of the reference genome. Reads mapping to the 107 nt hairpin-forming regions of the 5′- and 3′-ITRs were of lower quality than those aligned to the rest of the genome (Figure S3A).

Secondary structures of the 5′- and 3′- ITRs were predicted using the RNAfold Web Server ([22]; http://rna.tbi.univie.ac.at/cgi-bin/RNAWebSuite/RNAfold.cgi; accessed on 23 May 2025), applying DNA-specific parameters (Matthews model, 2004) at 25 °C. Potential G-quadruplex structures within the ITRs were predicted using the Quadruplex forming G-Rich Sequences (QGRS) Mapper Web Server ([23]; https://bioinformatics.ramapo.edu/QGRS/analyze.php; accessed on 27 April 2025).

### 4.8. PCR Screening of Whitefly DNA-Derived RCA Products

To identify the presence of the novel parvovirus BtaDV in the individual RCA products pooled for Illumina sequencing, diagnostic PCR primers were designed targeting the coding region of the VP gene: 5′-GCAGAAAGTTCTTTGGCGGG and 5′-CCTGTGTCCTTGGGTACGAC (amplicon size = 372 bp). Sequences derived from this VP gene region were not detected in any of the 15 genomes of *B. tabaci* biotypes/populations available in the NCBI Genome database (Dataset S1E).

PCR was performed in a 25 μL reaction containing 1 μL of the RCA product, 2 μL of primer mix (10 μM each), 1 μL of 2.5 mM dNTPs, 5 μL of 5× GoTaq buffer (Promega, Singapore), and 1 U homemade Taq DNA polymerase. The cycling conditions included an initial denaturation step at 94 °C for 5 min, followed by 30 cycles at 94 °C for 30 s, 60 °C for 30 s, and 72 °C for 30 s, with a final extension step at 72 °C for 10 min.

### 4.9. Phylogenetic Analysis Based on NS1 SF3 Helicase Domains of Parvoviridae

The evolutionary placement of the newly-identified parvovirus was determined through phylogenetic analysis of the most conserved SF3 helicase/Parvo NS1 domain of its NS1 protein following the current ICTV classification criteria ([3]; ICTV *Parvoviridae* taxonomic proposal 2022: https://ictv.global/taxonomy/taxondetails?taxnode_id=202404215&taxon_name=Parvoviridae; accessed on 1 April 2025). A reference dataset containing the most recent alignment of Parvo NS1 domains from *Parvoviridae* members was kindly provided by Dr. Pénzes and her colleagues from the ICTV *Parvoviridae* Study Group. The Parvo NS1 domain of BtaDV (amino acids 500–637 of the NS1 protein) was selected via MUSCLE multiple alignments and integrated into the reference dataset. Phylogenetic analyses were conducted using MEGA v. 12.0.11 [24], SDT v. 1.2 [25], and BEAST v. 1.10.4 [26].

For MEGA and SDT analyses, multiple sequence alignments were performed using MUSCLE with the default parameters. A maximum likelihood phylogenetic tree was constructed using MEGA with default parameters. The portion of the tree corresponding to the subfamily *Densovirinae* was cropped from the full tree image and manually annotated based on the ICTV *Parvoviridae* Taxonomic Proposal (2022) (Figure 3). The other portions of the tree are shown with manual annotations in Figure S2B. The SDT similarity matrix for the subfamily *Densovirinae* was manually annotated (Figure S2C).

For the BEAST analysis, multiple sequence alignment was performed using MAFFT v. 7 with default parameters and the --reorder option to cluster similar sequences in the output alignment. The alignment was converted to FASTA format and imported into BEAUti v. 1.10.4 (part of the BEAST package) to generate the XML configuration file required for Bayesian phylogenetic inference. Bayesian analysis was conducted via Markov Chain Monte Carlo (MCMC) sampling to explore the space of possible evolutionary trees and estimate the posterior probability of each clade based on the sequence data [26]. A maximum clade credibility (MCC) tree was generated using TreeAnnotator v. 1.10.4 (part of the BEAST package) and visualized using FigTree v. 1.4.4 ([27]; http://tree.bio.ed.ac.uk/software/figtree/; accessed on 27 April 2025).

## Data Availability

The complete genome sequence of *Bemisia tabaci* ambidensovirus (BtaDV) reported in this study was deposited in the NCBI GenBank under accession number PV695490.

## Supplementary Materials

The following supporting information can be downloaded at: https://www.mdpi.com/article/10.3390/pathogens14070714/s1, Figure S1: InterPro protein domain analysis for Bemisia tabaci ambidensovirus (BtaDV) NS and VP proteins; Figure S2A: BEAST-generated phylogenetic tree of the NS1 protein SF3 helicase/Parvo NS1 domain of Bemisia tabaci ambidensovirus (BtaDV) and all current members of the family *Parvoviridae*; Figure S2B: MEGA-generated Maximum Likelihood phylogenetic tree of the NS1 protein SF3 helicase/Parvo NS1 domain of Bemisia tabaci ambidensovirus (BtaDV) and all current members of the family *Parvoviridae*; Figure S2C: Sequence Demarcation Tool (SDT) analysis of the NS1 protein SF3 helicase/Parvo NS1 domain of Bemisia tabaci ambidensovirus (BtaDV) and all current members of the subfamily *Densovirinae*; Figure S3A: IGV visualization of the Illumina 150 nt reads from the ALYU-390 library mapped to the complete genome sequence of Bemisia tabaci ambidensovirus (BtaDV); Figure S3B: IGV visualization of the Illumina 150 nt reads from other ALYU-libraries mapped to the complete genome sequence of Bemisia tabaci ambidensovirus (BtaDV); Figure S3C: IGV visualization of the Illumina 150 nt reads from ALYU-381 library mapped to a consensus genome sequence of the ALYU-381 variants of Bemisia tabaci ambidensovirus (BtaDV) reconstructed by mapping the ALYU-381 reads to the BtaDV ALYU-390 reference sequence; Table S1: Whitefly samples collected in 2020 from crop fields in the Berkane region of Morocco; Dataset S1: Nucleotide and protein sequence analysis of the novel parvovirus Bemisia tabaci ambidensovirus (BtaDV), its relatives from the subfamily *Densovirinae* and BtaDV-related endogenous viral elements integrated into the genomes of different *B. tabaci* biotypes.
