# Supplementary material for: A Novel Parvovirus Associated with the Whitefly Bemisia tabaci
Source: Pathogens. 2025 Jul 19;14(7):714. doi: 10.3390/pathogens14070714 (PMC12297876; doi:10.3390/pathogens14070714)
Supplement: Supplementary file 1 [file pathogens-14-00714-s001.zip › Dataset S1A-E final.pdf]

Dataset S1. Nucleotide and protein sequence analysis of the novel parvovirus Bemisia tabaci ambidensovirus (BtaDV), its relatives from the subfamily *Densovirinae* and BtaDV-related endogenous viral elements integrated into the genomes of different *B. tabaci* biotypes.

(A) ALYU-390 contig representing the novel parvovirus and its BlastX analysis (page 1)
(B) Complete genome sequence of BtaDV with cis-acting elements annotated (pages 1-2)
(C) Sequence analysis of viral NS and VP proteins (pages 2-5)
(D) Sequence analysis of the ambidensoviruses most related to BtaDV (pages 6-13)
(E) Analysis of BtaDV-related endogenous viral elements (EVEs) integrated into the genomes of different *B. tabaci* biotypes (pages 13-44)

(A) ALYU-390 contig representing the novel parvovirus and its BlastX analysis

```
>Alyu390_Contig_12 = novel parvovirus reverse-complement sequence with inverted repeats underlined
GATCATATAAAACGGAGGAAAACCCCCCCTTCCCCCCTCCCCCGTCCCCTCCCCGACCGCCCGTGCGAGCGCACGCCGGAGGGCCCGTGGGAGGGATGGGTTCTTTTTATTTTAATCGTGTAGTTCAAAATGCCGCTGTGGCCTAAGCATAATTATCTTGGTCCGGGAAACGATTTAATTAACGGTGA
CCCTGAAGATGAAGACGATGAGGTTGCATTGCAACATGATATAAGTTACGAATTTGCGAAAAGTGAGGAAGAGATTTTTGAATCAGATAAGAGAGCAATTAGTGATTTTGCAAATATTGCTGTGAACGATTTGAAAAATTGGACATCAATACCGGAGCCATAGCAGGGACGGGACTTTTGTTTTTAA
AACATAACCTAGAAAGTGCAATCGGAACGACGCTTTACCCGTGGCATCCTGACGGTGGTGATGTAAGTGACATGAATAAAACTACGTACGCTCTACGTGAAAAAACCAAAAGTTTAGTGTATCAAAATTTAAAACGTCTGGGGAAAGTGCATACTTCTTATCGTGAATTTTGGCGGTCTGATTCTGGT
GCGAATTTAGCGCAAACAATTTATAGTGATTTTGCTAATCACGGTGAACATTGGCAGTCCTTAATTGCAGACGGGACTTTAGACATTGACAGGGTTTCGAATGGACTGGTTTCTCAGTACGTATCTACGTATTCTCGTAATTCTACTCCATCAATTGCCACATCCAGTGATACTCCTCCCCAATCACC
TGTTCCAGGACCCTCCACCTCCGGAGCCCCACGACAAGAAGCTGGAGATTTTGGAAATTCTGAAGGAGGAAAACGAAAAGCAGAAGAATCTTCTATACTTGATAATTCAAGTAATAAGCGTGTTGCTACTTCATCAAGTGGCAGTTCATCATCTTCACTTTCTGCTTCGTCTACTAACCAAAGTATGG
AGGTGGATGGTGTCACGGACCCGAAGGGAAAGCGAGGAACCGGTGCTGGAATCTCCAAGGTTACCCCCCCCGTCAGTTTAAATAAATCATCTACTCATTCTGGTAATAAGATTGTAATAACAAAGTCTAGAATGTTATTTTGTCATGCTATTGCTCCAGCTCAATTGTCTGTAAACGCCGAATTAAAC
ATTGGAAGAAATGTTAGTGCCAAAGTTTTAGGCTTAGCTTACATTCCAGTCGACTATTTGCCGTTTTATTTAACAGAGCAAGAGTACAACGCAATACCAGATAATTCCAGAGTGTTGCGTGTCGGGTGCAAAATAACACCTGTTGGCTGTCGCACCGCTTTTGATACAGGTACTTCAACCTCTGGCGT
AGCTACTAATGAGTGGATCGCTGTCGGCAGGAGTGTTGTCGGTTTAAATTTAAAAATACCGATTTACAATGTTCATTTATCAACAAAAGCCGATGAACCTATGATCCCAACATCAATTTCTAGATTGGATCATACTACTATGCAAAATAGCTGGTATTTAAATAATGATATTTGCAATGTTCTAGGTT
ATCCAACTCAAATAAACGAGTATGCAGCTTATATTTTTAATACTAATTCTGAAAAAAACACTACTTATAAGAAGGATGTTGATTATAGTTATCATTGCGGTGGTAATCCTAGATTAGATAAAGTTATTGAAAGATTCATGTTCAATGCTCACATTGGAAGCCCTATTATAGATTATGAGTATTCACCG
CAAAATGCTATGATTAAGAAACACAAAATAGTAAATTTGCCCTATCCTACATGCTCGCAAGTTATCTTAAACGGTGAAAATCCTGGCCCTTGGAATATAAATATTGATGGTTGGGCTTCCGATGTTGATCATGATAAAAATAAAGGAAGAGCATATTATAAATCTGTGAATTCGGCAACGTCTGATAT
GTACGTTCCATTTGAGTGCAAATATAATCGCATGTTAGAGTCAGGTCCTTACTCTCACAATTTCAAGGCCGCAGGTGGTGAAAATATTCAACCTCAAGTACATATAGGCCTCATGCCGACGCCAAACATTAATCCTGCAAACTCAAGTGTTTCATTTCAGAATTCAGCGGCATACTTTGAAGTGGAGT
GTTATATTGAGATAAGCAGTAATGTTGACTCTGCGTTTACTGGTGGTACTGACGCTGTTATGTGGAATGACGATATCACTATCTACTACCACGACACGGTGCCTTTGCATGACGGACTCTCAGTTGGCAAGAAGCGCGTCAAACCTTCCTCGGCCAGTGTTTATGCGGGAGAGACTTCGAAACATTCG
AAGATGCCGAGATCCCGCTTGCAAGCTGAAGCGTCCGAGAAGCAAAGTGCCGTCGAAAATACTCAGTACTACAAAGATATAATGAAAATTAAGGCATTGGACGTGGAAAATAAAAAGAAATAATATATGCTGAATATTATATATGTATTTAAATAAAATATAAATGATTATAATGTTTATTGAGTTAT
ATATCTTTCATAAAAATATCCATTATCCTTGAAATATTATCTTCATCATGGTGAATTGCTTCTGTTGACCGGATTGAAGAATCTATATCATGAGTCGCTTCCTGTATGAAAGTGGTGACACTTAAGTCCTGCAACAAACTAGAGTCGAAATTATCCAATTCCAACGTATCAATAAGGTTATACTTTGC
CAAAACATAAAAAAGTGCTAAAGGGTGAGGTTTTTTATGAATTTCTGCCAAGAAAGGTGACTGTCGCCATGTGTATTTAATCATTCTACTATTAAATGGCGGGTCATTAGGGAAAGGATTGTTGTTCGTTAGAACTATTACGGGAGTGCGCATGATTGTCGCATCATTTTTGTATTTAATTTTTGCGG
GGCAAGGGTCCCCAGCGAAAAGCATTTTTATAGTTTCCACCGCGGATGGTTCGCAAACCGGTTCATTCCATAATAAGATCCTTTTCATGTTGCACTCTTGTAATGGAAAGTTACAAAATCTGTTAAAATTTCCGACGTGACCACAATTTATCATAGCTTGTGTCACTGCATCAAAAAAAAAATTTTT
CCTGAATTAGCAGGACCAGCAATTAACAAAGTATTAACTTTTGGAATTCGCTTATCAAGAATATTTAACAAATTTTCGAGAAAATTTACGAATCCGAAACTGCCAACCTGGTGTTTCAATAAAGTTTCAATGCAAAGGATGCTAGCAGATAAATTCAAAAAATAATCCTCAGGATTATCGCAATTATA
AAACAATTTTGAGAAAGGAATATTTAACTGGTACTGAAATATTTGTTGCACAGTCATCTTGTTAATCGTATGTGTGTGCTGTAAAATGGCAAGCTGACAAGACATAGATTCAGGTGTAATAAAATATAAAGATTTATCGAATAAGTACAATTGGGATTGAAAAATTCGAGAAAAAGGAGTTATTACGC
ACGACTGAAGAAATTGAATTATTTTCTCCCTTTTTGTTGGGGCAGGTACTCCACCTTCCTCTCCGGAATTTTCGCTATCTTCTTCACTTGATCTTTCAGGATTTTTTTGATGCTGTTTGCGGCAGGTTCCGCGTAAGAAAGAAGAAATGTGCTCCGGTAATCGGCCTCCTTCCACCAGTTCTCCTTTG
CCACTCTGCGAAGATTGCGTGTCTTGTAAAAGTCCAGCTTGACGAGATAATCGCCTTTCTCGCCCGGCGAGGAAAGCTTGAAGGAGCCTGCGTCGCCCTTTTTGTATATAATTTGCGAGATGTTCGAGGTGGCCTTCTGTGATCCTACTGCCTCGTAAAGTATGTCTTCCAATTCGTCGTCCGAATCG
CTCTTCCGCTTTCTTCGTGAGTGCGCACGAGCACCACGACCTTTCGTATCGGCACTTGTGGATGACGTGGACATGCTGGCCGTGCTGGAAGACGACGAATGTCCATGCCGATGATGAGTTGCCTGTGTCCTTGGGTACGACTCCCCTATCATTTTCCCGAAAGCGCCCGGAAATTGTATTTCGGTAAA
TGTCGTGTAATATGTACCTTCGGTGATTATCCAGTCGGCCAAATTCTGCACAGAATAAGCTTTTAAATTTTTCGAAAGAAACGGTTTCAAAACTTTCTTGGTAGCTATTTGAGCTTCCCGCCAAAGAACTTTCTGCTCCTGAGAAATATTCGTCAATCCGTTTGCTTCGATTACGCCTACGAACTCGG
ACATCAGTCGAGGCAACGGAAGAAGGCCGAGGGGGTGTTCCATGGAGTAATCCTGTATCGCTGTCAAAAGGGGAGATCGATCGATAAAGTCCTGAATTTCCTGCTTCCTTGTTTCCGTCATCTCGAGGGATGGACCCGCTTGAGGCTCCGGAGGATCCTTCTCCAAGATATGGACTTTTTTCTGTTTG
TCTACTTCCGACAAATTCGAAAGCATTTCCGTCTCCGGAGATGTTTGACGGGTTTTCCGGCTTTTTTTTGAGGACATCTTCTGCACAAACTATCGGGATACACTTTGTTACTCCGTCCCTGACTAAATAAACAGGCTGGTTTGTTGCGATACAATGTTCAACGTACCTTTCTTCACTAGGGGATAATT
GTTCAACTTGCACGAATTCACTACTATCTGTATCTATCACTGAAAATGAATCTTGAGAACTGTTATCGTCGCTTAAATGAGCAGACATACCGGCAAAAAAACTGAACTGCGACGAATCCCGCACTCGAGCCACCTTTTATTTACCTACCTTGTTTAAACTCTTATCAACTTCAAAATGACAATAACAA
AATATTTGGTTCCCGCACGCGATTCCCGACCGCCGATAAAAAACAAATTACACATTCCCTCGTACGCACGTATCAGCGATAAAAACATAGTTGCCGCTTATTACGTAATTACTCTTTCAATTTCAATTTCACGATTAAAATAAAAAGAACCCATCCCTCCCACGGGCCCTCCGGCGTGCGCTCGCACG
GGCGGTCGGGGAGGGGACGGGGGAGGGGGGAAGGGGGGGTTTCCTCCGTTTTATATGATCCTTATCATGACGTAACGAC
```

BlastX analysis of the contig

Distribution of the top 100 Blast Hits on 100 subject sequences

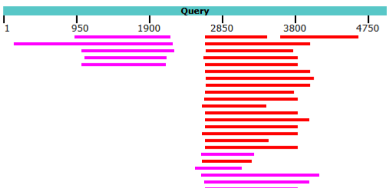

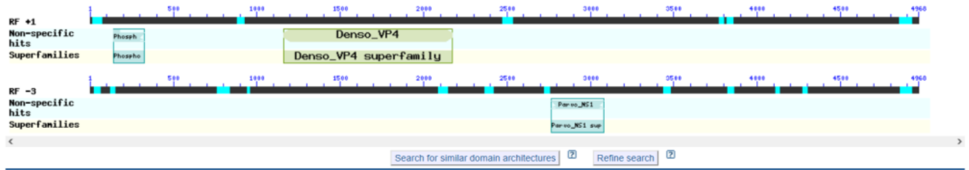

List of domain hits

Name	Accession	Description	Interval	E-value
Denso_VP4	pfam02336	Capsid protein VP4; Four different translation initiation sites of the densovirus capsid ...	1162-2172	4.73e-15
Phospholip_A2_4	pfam08398	Phospholipase A2-like domain; This entry represents a domain that is likely to be a ...	142-327	1.84e-05
Parvo_NS1	pfam01057	Parvovirus non-structural protein NS1; This family also contains the NS2 protein. Parvoviruses ...	2765-3079	7.60e-07

(B) Complete genome sequence of the novel parvovirus BtaDV with cis-acting elements annotated

Inverted terminal repeats (ITRs) with imperfect palindromes forming 5'- and 3'-hairpins with an internal flip-flop palindrome sequences and **G-quadruplex** elements; TATA-box and TATA-like promoter elements and AATAAA poly(A) signals of the NS and VP transcription units; **ATG** start and **TAA** stop codons of ORFs for NS1, NS2, VP and VP-short proteins; GT splice donor site active in the endogenous copy of the virus

```
>BtaDV_Alyu390 complete genome
TTCCTCCGTTTTATATGATCCTTATCATGACGTAACGACTATAAAATCGATCGTAGATCATGATTTTAGGGTCGTTACGTCGATAAGGATCATATAAAACGGAGGAAAACCCCCCCTTCCCCCCTCCCCCGTCCCCTCCCCGACCGCCCGTGCGAGCGCACGCCGGAGGGCCCGTGGGAGGGATGGGT
TCTTTTTATTTTAATCGTGAAATTGAAATTGAAAGAGTAATTACGTAATAAGCGGCAACTATGTTTTTATCGCTGATACGTGCGTACGAGGGAATGTGTAATTTGTTTTTTATCGGCGGTCGGGAATCGCGTGCGGGAACCAAATATTTTGTTATTGTCATTTTGAAGTTGATAAGAGTTTAAACAAG
GTAGGTAAATAAAAGGTGGCTCGAGTGCGGGATTCGTCGCAGTTCAGTTTTTTGCCGGTATGTCTGCTCATTTAAGCGACGATAACAGTTCTCAAGATTCATTTTCAGTGATAGATACAGATAGTAGTGAATTCGTGCAAGTTGAACAATTATCCCCTAGTGAAGAAAGGTACGTTGAACATTGTAT
CGCAACAAACCAGCCTGTTTATTTAGTCAGGGACGGAGTAACAAAGTGTATCCCGATAGTTTGTGCAGAAGATGTCCTCAAAAAAAAGCCGGAAAACCCGTCAAACATCTCCGGAGACGGAAATGCTTTCGAATTTGTCGGAAGTAGACAAACAGAAAAAAGTCCATATCTTGGAGAAGGATCCTCCG
GAGCCTCAAGCGGGTCCATCCCTCGAGATGACGGAAACAAGGAAGCAGGAAATTCAGGACTTTATCGATCGATCTCCCCTTTTGACAGCGATACAGGATTACTCCATGGAACACCCCCTCGGCCTTCTTCCGTTGCCTCGACTGATGTCCGAGTTCGTAGGCGTAATCGAAGCAAACGGATTGACGAA
TATTTCTCAGGAGCAGAAAGTTCTTTGGCGGGAAGCTCAAATAGCTACCAAGAAAGTTTGAAACCGTTTCTTTCGAAAAATTTAAAAGCTTATTCTGTGCAGAATTTGGCCGACTGGATAATCACCGAAGGTACATATTACACGACATTTACCGAAATACAATTTCCGGGCGCTTTCGGGAAAATGA
TAGGGGAGTCGTACCCAAGGACACAGGCAACTCATCATCGGCATGGACATTCGTCGTCTTCCAGCACGGCCAGCATGTCCACGTCATCCACAAGTGCCGATACGAAAGGTCGTGGTGCTCGTGCGCACTCACGAAGAAAGCGGAAGAGCGATTCGGACGACGAATTGGAAGACATACTTTACGAGGCA
GTAGGATCACAGAAGGCCACCTCGAACATCTCGCAAATTATATACAAAAAGGGCGACGCAGGCTCCTTCAAGCTTTCCTCGCCGGGCGAGAAAGGCGATTATCTCGTCAAGCTGGACTTTTACAAGACACGCAATCTTCGCAGAGTGGCAAAGGAGAACTGGTGGAAGGAGGCCGATTACCGGAGCAC
ATTTCTTCTTTCTTACGCGGAACCTGCCGCAAACAGCATCAAAAAAATCCTGAAAGATCAAGTGAAGAAGATAGCGAAAATTCCGGAGAGGAAGGTGGAGTACCTGCCCCAACAAAAAGGGAGAAAATAATTCAATTTCTTCAGTCGTGCGTAATAACTCCTTTTTCTCGAATTTTTCAATCCCAATT
GTACTTATTCGATAAATCTTTATATTTTATTACACCTGAATCTATGTCTTGTCAGCTTGCCATTTTACAGCACACACATACGATTAACAAGATGACTGTGCAACAAATATTTCAGTACCAGTTAAATATTCCTTTCTCAAAATTGTTTTATAATTGCGATAATCCTGAGGATTATTTTTTGAATTTAT
CTGCTAGCATCCTTTGCATTGAAACTTTATTGAAACACCAGGTTGGCAGTTTCGGATTCGTAAATTTTCTCGAAAATTTGTTAAATATTCTTGATAAGCGAATTCCAAAAGTTAATACTTTGTTAATTGCTGGTCCTGCTAATTCAGGAAAAAATTTTTTTTTGATGCAGTGACACAAGCTATGATA
AATTGTGGTCACGTCGGAAATTTTAACAGATTTTGTAACTTTCCATTACAAGAGTGCAACATGAAAAGGATCTTATTATGGAATGAACCGGTTTGCGAACCATCCGCGGTGGAAACTATAAAAATGCTTTTCGCTGGGGACCCTTGCCCCGCAAAAATTAAATACAAAAATGATGCGACAATCATGCG
CACTCCCGTAATAGTTCTAACGAACAACAATCCTTTCCCTAATGACCCGCCATTTAATAGTAGAATGATTAAATACACATGGCGACAGTCACCTTTCTTGGCAGAAATTCATAAAAAACCTCACCCTTTAGCACTTTTTTATGTTTTGGCAAAGTATAACCTTATTGATACGTTGGAATTGGATAATT
TCGACTCTAGTTTGTTGCAGGACTTAAGTGTCACCACTTTCATACAGGAAGCGACTCATGATATAGATTCTTCAATCCGGTCAACAGAAGCAATTCACCATGATGAAGATAATATTTCAAGGATAATGGATATTTTTATGAAAGATATATAACTCAATAAACATTATAATCATTTATATTTTATTTAA
ATACATATATAATATTCAGCATATATTATTTCTTTTTATTTTCCACGTCCAATGCCTTAATTTTCATTATATCTTTGTAGTACTGAGTATTTTCGACGGCACTTTGCTTCTCGGACGCTTCAGCTTGCAAGCGGGATCTCGGCATCTTCGAATGTTTCGAAGTCTCTCCCGCATAAACACTGGCCGAG
GAAGGTTTGACGCGCTTCTTGCCAACTGAGAGTCCGTCATGCAAAGGCACCGTGTCGTGGTAGTAGATAGTGATATCGTCATTCCACATAACAGCGTCAGTACCACCAGTAAACGCAGAGTCAACATTACTGCTTATCTCAATATAACACTCCACTTCAAAGTATGCCGCTGAATTCTGAAATGAAAC
ACTTGAGTTTGCAGGATTAATGTTTGGCGTCGGCATGAGGCCTATATGTACTTGAGGTTGAATATTTTCACCACCTGCGGCCTTGAAATTGTGAGAGTAAGGACCTGACTCTAACATGCGATTATATTTGCACTCAAATGGAACGTACATATCAGACGTTGCCGAATTCACAGATTTATAATATGCTC
TTCCTTTATTTTTATCATGATCAACATCGGAAGCCCAACCATCAATATTTATATTCCAAGGGCCAGGATTTTCACCGTTTAAGATAACTTGCGAGCATGTAGGATAGGGCAAATTTACTATTTTGTGTTTCTTAATCATAGCATTTTGCGGTGAATACTCATAATCTATAATAGGGCTTCCAATGTGA
GCATTGAACATGAATCTTTCAATAACTTTATCTAATCTAGGATTACCACCGCAATGATAACTATAATCAACATCCTTCTTATAAGTAGTGTTTTTTTCAGAATTAGTATTAAAAATATAAGCTGCATACTCGTTTATTTGAGTTGGATAACCTAGAACATTGCAAATATCATTATTTAAATACCAGCT
ATTTTGCATAGTAGTATGATCCAATCTAGAAATTGATGTTGGGATCATAGGTTCATCGGCTTTTGTTGATAAATGAACATTGTAAATCGGTATTTTTAAATTTAAACCGACAACACTCCTGCCGACAGCGATCCACTCATTAGTAGCTACGCCAGAGGTTGAAGTACCTGTATCAAAAGCGGTGCGAC
AGCCAACAGGTGTTATTTTGCACCCGACACGCAACACTCTGGAATTATCTGGTATTGCGTTGTACTCTTGCTCTGTTAAATAAAACGGCAAATAGTCGACTGGAATGTAAGCTAAGCCTAAAACTTTGGCACTAACATTTCTTCCAATGTTTAATTCGGCGTTTACAGACAATTGAGCTGGAGCAATA
GCATGACAAAATAACATTCTAGACTTTGTTATTACAATCTTATTACCAGAATGAGTAGATGATTTATTTAAACTGACGGGGGGGGTAACCTTGGAGATTCCAGCACCGGTTCCTCGCTTTCCCTTCGGGTCCGTGACACCATCCACCTCCATACTTTGGTTAGTAGACGAAGCAGAAAGTGAAGATGA
TGAACTGCCACTTGATGAAGTAGCAACACGCTTATTACTTGAATTATCAAGTATAGAAGATTCTTCTGCTTTTCGTTTTCCTCCTTCAGAATTTCCAAAATCTCCAGCTTCTTGTCGTGGGGCTCCGGAGGTGGAGGGTCCTGGAACAGGTGATTGGGGAGGAGTATCACTGGATGTGGCAATTGATG
GAGTAGAATTACGAGAATACGTAGATACGTACTGAGAAACCAGTCCATTCGAAACCCTGTCAATGTCTAAAGTCCCGTCTGCAATTAAGGACTGCCAATGTTCACCGTGATTAGCAAAATCACTATAAATTGTTTGCGCTAAATTCGCACCAGAATCAGACCGCCAAAATTCACGATAAGAAGTATGC
ACTTTCCCCAGACGTTTTAAATTTTGATACACTAAACTTTTGGTTTTTTCACGTAGAGCGTACGTAGTTTTATTCATGTCACTTACATCACCACCGTCAGGATGCCACGGGTAAAGCGTCGTTCCGATTGCACTTTCTAGGTTATGTTTTAAAAACAAAAGTCCCGTCCCTGCTATGGCTCCGGTATT
GATGTCCCAATTTTTCAAATCGTTCACAGCAATATTTGCAAAATCACTAATTGCTCTCTTATCTGATTCAAAAATCTCTTCCTCACTTTTCGCAAATTCGTAACTTATATCATGTTGCAATGCAACCTCATCGTCTTCATCTTCAGGGTCACCGTTAATTAAATCGTTTCCCGGACCAAGATAATTAT
GCTTAGGCCACAGCGGCATTTTGAACTACACGATTAAAATAAAAAGAACCCATCCCTCCCACGGGCCCTCCGGCGTGCGCTCGCACGGGCGGTCGGGGAGGGGACGGGGAGGGGGGAAGGGGGGTTTTCCTCCGTTTTATATGATCCTTATCGACGTAACGACCCTAAAATCATGATCTACGATCG
ATTTTATAGTCGTTACGTCATGATAAGGATCATATAAAACGGAGGAA
```

Telomere hairpin sequences with a 55 nt flip-flop palindrome

```
5'-hairpin         1 TTCCTCCGTTTTATATGATCCTTATCATGACGTAACGACTATAAAATC--GATCGTA-GATCATGATTTTAGGGTCGTTACGTC--GATAAGGATCATATAAAACGGAGGAA     107
                     ||||||||||||||||||||||||||  |||||||||||..|||||||  ||||  ||  ||||  |||||||..||||||||||||  ||||||||||||||||||||||||||
3'-hairpin      5017 TTCCTCCGTTTTATATGATCCTTATC--GACGTAACGACCCTAAAATCATGATC-TACGATC--GATTTTATAGTCGTTACGTCATGATAAGGATCATATAAAACGGAGGAA     5123

5'-flip-flop                          1 ATGACGTAACGACTATAAAATCGATCGTAGATCATGATTTTAGGGTCGTTACGTC 55

5'-flip-flop reverse complement       1 GACGTAACGACCCTAAAATCATGATCTACGATCGATTTTATAGTCGTTACGTCAT 55
                                          |||||||||||||||||||||||||||||||||||||||||||||||||||||||
3'-flip-flop                          1 GACGTAACGACCCTAAAATCATGATCTACGATCGATTTTATAGTCGTTACGTCAT 55
```

Consensus genome sequence reconstructed from another whitefly pool (ALYU-381)

```
>BtaDV_Alyu381 consensus sequence
TTCCTCCGTTTTATATGATCCTTATCATGACGTAACGACTATAAAATCGATCGTAGATCATGATTTTAGGGTCGTTACGTCGATAAGGATCATATAAAACGGAGGAAAACCCCCCCTTCCCCCCTCCCCCGTCCCCTCCCCGACCGCCCGTGCGAGCGCACGCCGGAGGGCCCGTGGGAGGGATGGGT
TCtTTTTTATTTTAATCGTGAAATTGAAATTGAAAGAGTAATTACGTAATAAGCGGCAACTATGTTTTTATCGCTGATACGTGCGTACGAGGGAATGTGTAATTTGTTTTTTATCGGCGGTCGGGAATCGCGTGCGGGAACgAAATATTTTGTTATTGTCATTTTGAAGTTGATAAGAGTTTAAACAA
```

```
GGTAGGTAAATAAAAGGTGGCTCGAGTGCGGGATTCGTCGCAGTTCAGTTTTTTTCCGGTATGTCTGCTCATTTAAGCGACGATAACAGTTCTCAAGATTCATTTTCAGTGATAGATACAGATAGTAGTGAATTCGTGCAAGTTGAACAATTATCCCCTAGTGAAGAgAGGTACGTTGAACATTGTAT
CGCAACAAACCAGCCTGTTTATTTAGTCAGGGACGGAGTAACAAAGTGTATCCCGATAGTTTGTGCAGAAGATGTCCTCAAAAAAAAGCCGGAAAACCCGTCAAACATCTCCGGAGACGGAAATGCTTTCGAATTTGTCGGAAGTAGACAAACAGAAAAAAGTCCATATCTTGGAGAAGGATCCTCCG
GAGCCTCAAGCGGGTCCATCCCTCGAGATGACGGAAACAAGGAAGCAGGAAATTCAGGACTTTATCGATCGATCaCCCCTTcTGACAGCGATACAGGATTACTCCATGGAACACCCCCTCGGCCTTCTTCCGTTGCCTCGACTGATGTCCGAGTTCGTAGGCGTAATCGAAGCAAACGGATTGACGAA
TATTTCTCAGGAGCAGAAAGTTCTTTGGCGGGAAGCTCAAATAGCTACCAAGAAAGTTTTGAAACCGTTTCTTTCGAAAAATTTAAAAGCTTATTCTGTGCAGAATTTGGCCGACTGGATAATCACCGAAGGTACATATTACACGACATTTACCGAAATACAATTTCCGGGCGCTTTCGGGAAAATGA
TAGGGGAGTCGTACCCAAGGACACAGGCAACTCATCATCGGCATGGACATTCGTCGTCTTCCAGCACGGCCAGCATGTCCACGTCATCCACAAGTGCCGATACGAAAGGTCGTGGTGCTCGTGCGCACTCACGAAGAAAGCGGAAGAGCGATTCGGACGACGAATTGGAAGACATACTTTACGAGGCA
GTAGGATCACAGAAGGCCACCTCGAACATCTCGCAAATTATATACAAAAAGGGCGACGCAGGCTCCTTCAAGCTTTCCTCGCCGGGCGAGAAAGGCGATTATCTCGTCAAGCTGGACTTTTACAAGACACGCAATCTTCGCAGAGTGGCAAAGGAGAACTGGTGGAAGGAGGCCGATTACCGGAGCAC
ATTTCTTCTTTCTTACGCGGAACCTGCCGCAAACAGCATCAAAAAAATCCTGAAAGATCAAGTGAAGAAGATAGCGAAAATTCCGGAGAGGAAGGTGGAGTACCTGCCCCAACAAAAAGGGAGAAAATAATTCAATTTCTTCAGTCGTGCGTAATAACTCCTTTTTCTCGAATTTTTCAATCCCAATT
GTACTTATTCGATAAATCTTTATATTTTATTACACCTGAATCTATGTCTTGTCAGCTTGCCATTTTACAGCACACACATACGATTAACAAGATGACTGTGCAACAAtTATTTCAGTACCAGTTAAATATTCCTTTCTCAAAATTGTTTTATAATTGCGATAATCCTGAGGATTATTTTTTGAATTTAT
CTGCTAGCATCCTTTGCATTGAAACTTTATTGAAACACCAGGTTGGCAGTTTCGGATTtGTAAATTTTCTCGAAAATTTGTTAAATATTCTTGATAAGCGAATTCCAAAAGTTAATACTTTGTTAATTGCTGGTCCTGCTAATTCAGGAAAAAATTTTTTTTTGATGCAGTGACACAAGCTATGATA
AATTGTGGTCACGTCGGAAATTTTAACAGATTTTGTAACTTTCCATTACAAGAGTGCAACATGAAAAGGATCTTATTATGKAATGAACCGGTTTGCGAACCATCCGCGGTGGAAACTATAAAAATGCTTTTCGCTGGGGACCCTTGCCCCGCAAAAATTAAATACAAAAATGATGCGACAATCATGCG
CACTCCCGTAATAGTTCTAACGAACAACAATCCTTTCCCTAATGACCCGCCATTTAATAGTAGAATGATTAAATACACATGGCGACAGTCACCTTTCTTGGCAGAAATTCATAAAAAACCTCACCCTTTAGCACTTTTTTATGTTTTGGCAAAGTATAACCTTATTGATACGTTGGAATTGGATAATT
TCGACTCTAGTTTGTTGCAGGACTTAAGTGTCACCACaTTCATACAGGAAGCGACTCATGATATAGATTCTTCAATCCGGTCAACAGAAGCAATTCACCATGATGAAGATAATATTTCAAGGATAATGGATATTTTTATGAAAGATATATAAgTCAATAAACATTATAATCATTTATATTTTATTTAA
ATACATATATAATATTCAGCATATATTATTTCTTTTTATTTTCCACGTCCAAcGCCTTAATTTTCATTATATCTTTGTAGTACTGAGTATTTTCGACGGCACTTTGCTTCTCGGACGCcTCAGCTTGCAAGCGGGATCTCGGCATCTTCGAATGTTTCGAAGTCTCTCCCGCATAAACACTGGCCGAG
GAAGGTTTGACGCGCTTCTTGCCAACTGAGAGTCCGTCATGCAAAGGCACCGTGTCGTGGTAGTAGATAGTGATATCGTCATTCCACATAACAGCGTCAGTACCACCAGTAAACGCAGAGTCAACATTACTGCTTATCTCAATATAACACTCCACTTCAAAGTATGCCGCTGAATTCTGAAATGAAAC
ACTTGAGTTTGCAGGATTAATGTTTGGCGTCGGCATGAGGCCTATATGTACTTGAGGTTGAATATTTTCACCACCTGCGGCCTTGAAATTGTGAGAGTAAGGACCTGACTCTAACATGCGATTATATTTGCACTCAAATGGAACGTACATATCAGACGTTGCCGAATTCACAGATTTATAATATGCTC
TTCCTTTATTTTTATCATGATCAACATCGGAAGCCCAACCATCAATATTTATATTCCAAGGGCCAGGATTTTCACCGTTTAAaATAACTTGCGAGCATGTAGGATAGGGCAAATTTACTATTTTGTGTTTCTTAATCATAGCATTTTGCGGTGAATAtTCATAATCTATAATAGGGCTTCCAATGTGA
GCATTGAACATGAATCTTTCAATAACTTTATCTAATCTAGGATTACCACCGCAATGATAACTATAATCAACATCCTTCTTATAAGTAGTGTTTTTTCAGAATTAGTATTAAAAATATAAGCTGCATACTCGTTTATTTGAGTTGGATAACCTAGAACATTGCAAATATCATTATTTAAATACCAGCT
ATTTTGCATAGTAGTATGATCCAATCTAGAAATTGATGTaGGGATCATAGGTTCATCGGCTTTTGTTGATAAATGAACATTGTAAATCGGTATTTTTAAATTTAAACCGACAACACTCCTGCCaACAGCaATCCACTCATTAGTAGCTACGCCAGAGGTTGAAGTACCTGTATCAAAAGCGGTGCGAC
AGCCAACAGGTGTTATTTTGCACCCGACACGCAACACTCTGGAATTATCTGGTATTGCGTTGTACTCTTGCTCTGTTAAATAAAACGGCAAATAGTCGACTGGAATGTAAGCTAAGCCTAAAACTTTGGCACTAACATTTCTTCCAATGTTTAATTCGGCGTTTACAGACAATTGAGCTGGAGCAATA
GCATGACAAAATAACATTCTAGACTTTGTTATTACAATCTTATTACCAGAATGAGTAGATGATTTATTTAAACTGACGGGGGGGTAACCTTGGAGATTCCAGCACCGGTTCCTCGCTTTCCCTTCGGGTCCGTGACACCATCCACCTCCATACTTTGGTTAGTAGACGAAGCAGAAAGTGAAGATGA
TGAACTGCCACTTGATGAAGTAGCAACACGCTTATTACTTGAATTATCAAGTATAGAAGATTCTTCTGCTTTTCGTTTTCCTCCTTCAGAATTTCCAAAATCTCCAGCTTCTTGTCGTGGGGCTCCGGAGGTGGAGGGTCCTGGAACAGGTGATTGGGGAGGAGTATCACTGGATGTGGCAATTGATG
GAGTAGAATTACGAGAATACGTAGATACGTACTGAGAAACCAGTCCATTCGAAACCCTGTCAATGTCTAAAGTCCCGTCTGCAATTAAGGACTGCCAATGTTCACCGTGATTAGCAAAATCACTATAAATTGTTTGCGCTAAATTCGCACCAGAATCAGACCGCCAAAATTCACGATAAGAAGTATGC
ACTTTCCCCAGACGTTTTAAATTTTGATACACTAAACTTTTGGTTTTTTCACGTAGAGCGTACGTAGTTTTATTCATGTCACTTACATCACCACCGTCAGGATGCCACGGGTAAAGCGTCGTTCCGATTGCACTTTCTAGGTTATGTTTTAAAAACAAAAGTCCCGTCCCTGCTATGGCTCCGGTATT
GATGTCCCAATTTTTCAAATCGTTCACAGCAATATTTGCAAAATCACTAATTGCTCTCTTATCTGATTCAAAAATCTCTTCCTCACTTTTCGCAAATTCGTAACTTATATCATGTTGCAATGCAACCTCATCGTCTTCATCTTCAGGGTCACCGTTAATTAAATCGTTTCCCGGACCAAGATAATTAT
GCTTAGGCCACAGCGGCATTTTGAACTACACGATTAAAATAAAAAGAACCCATCCCTCCCACGGGCCCTCCGGCGTGCGCTCGCACGGGCGGTCGGGGAGGGGACGGGGGAGGGGGGAAGGGGGGGTTTTCCTCCGTTTTATATGATCCTTATCGACGTAACGACCCTAAAATCATGATCTACGATCG
ATTTTATAGTCGTTACGTCATGATAAGGATCATATAAAACGGAGGAA
```

Positions of single-nucleotide indels are highlighted in cyan and SNPs in yellow

(C) Sequence analysis of viral NS and VP proteins encoded in the BtaDV_ALYU-390 genome

>BtaDV_NS1 719 amino acids

```
MSAHLSDDNSSQDSFSVIDTDSSEFVQVEQLSPSEERYVEHCIATNQPVYLVRDGVTKCIPIVCAEDVLKKKPENPSNISGDGNAFEFVGSRQTEKSPYLGEGSSGASSGSIPRDDGNKEAGNSGLYRSISPFDSDTGLLHGTPPRPSSVASTDVRVRRRNRSKRIDEYFSGAESSLAGSSNSYQESF
ETVSFEKFKSLFCAEFGRLDNHRRYILHDIYRNTISGRFRENDRGVVPKDTGNSSSAWTFVVFQHGQHVHVIHKCRYERSWCSCALTKKAEERFGRRIGRHTLRGSRITEGHLEHLANYIQKGRRRLLQAFLAGRERRLSRQAGLLQDTQSSQSGKGELVEGGRLPEHISSFLRGTCRKQHQKNPERS
SEEDSENSGEEGGVPAPTKREKIIQFLQSCVITPFSRIFQSQLYLFDKSLYFITPESMSCQLAILQHTHTINKMTVQQIFQYQLNIPFSKLFYNCDNPEDYFLNLSASILCIETLLKHQVGSFGFVNFLENLLNILDKRIPKVNTLLIAGPANSGKNFFFDAVTQAMINCGHVGNFNRFCNFPLQECN
MKRILLWNEPVCEPSAVETIKMLFAGDPCPAKIKYKNDATIMRTPVIVLTNNNPFPNDPPFNSRMIKYTWRQSPFLAEIHKKPHPLALFYVLAKYNLIDTLELDNFDSSLLQDLSVTTFIQEATHDIDSSIRSTEAIHHDEDNISRIMDIFMKDI
```

The NS1 Rep endonuclease motifs_HuH and YxxK and the most conserved Parvo NS1 domain sequence used for phylogenetic analysis are highlighted in yellow and green

BlastN matches

nonstructural protein Bactericera trigonica densovirus: 81% coverage; 30.67% identity

```
Sequence ID: YP_010802686.1 Length: 676
Identities     Positives      Gaps
192/626(31%)   296/626(47%)   69/626(11%)

Query  83    GNAFEFVGSRQTEKSPYLGEGSSGASSGSIPRDDGNKEAGNSGLYRSISPFDSDTGLLHG  142
             G A    G RQTE+S    E      S       G++  G S     I   D   G
Sbjct  76    GRAQHIDGRRQTEESLLEREEGDRDHSLEWQNASGDEGEGESDSEADIGAADKSLGSTGQ  135

Query  143   TPP--RPSSVASTDVRVRRRNRSKRIDEY--FSGAESSLAGSSNSYQESFETVSFEKFKS  198
               P   PS V   D      N      EY  + GA+  L    +        V  ++++
Sbjct  136   HFPCRTPSHVERNDPS--GSNSLHESYEYGGYEGAQPELQKEDHGLASGAPAVEKKRWRD  193

Query  199   LFCAEFGRLDN--HRRYILHDIYRNTISGR----FRENDRGVVPKDTGNSSSAWTFVVFQ  252
             L+      L N   +  ILH +YR   +GR    F +  R  +P+ TGN+      ++ +
Sbjct  194   LYQFTVSCLQNAPSKHVILHSVYRLCAAGRVDRQFHQRVR-RLPQFTGNTIQ----LISE  248

Query  253   HGQHVHVIHKCRYERSWCSCALTKKAEERFGR---RIGRHTLRGSRITEGHLEHLANYIQ  309
             HG H+HV+H C++  S C C   +     FG    R  R  +R  + +  HL     Y+
Sbjct  249   HGDHIHVLHDCQWTSSTCRCGFIRAFGSDFGTTHWRAKRWVVRRYKFSTAHLTSALLYLS  308

Query  310   KGRRRLLQAFLAGRERRLSRQAGLLQDTQSSQSG--------KGELVEGGRLPEH---IS  358
                  L    L     R   Q GL   TQ+            +  LVE G   +H   +
Sbjct  309   TRGHELGHVQLG----RQVFQRGLGSGTQTGHCPIQACFSHPEERLVECGLSEDHSYILG  364

Query  359   SFLRGTCRKQHQKNPERSSEEDSENSGEEGGVPAPTKRE---------KIIQFLQSCVIT  409
              F  G C  Q+    +R+S  +   SG+     AP +R+         +++ +L+
Sbjct  365   EFGVGEC-GQNSGGSDRASFPN-RASGQ-----APKRRKVSFEVQLGIELLGWLRQFPSA  417

Query  410   PFSRIFQSQLYLFDKSLYFITPESMSCQLAILQHTHTIN--KMTVQQIF-QYQLNIPFSK  466
             P   I Q++ +   +S + +     S  L  + + + +    M++ ++F ++Q   P
Sbjct  418   PLVSILQTRFW--TESEFGLQVHRGSKLLCTVLNNYALELCDMSMPELFARFQSVDPVHL  475

Query  467   LFYN-CDNPEDYFLNLSASILCIETLLKHQ-------VGSFGFVNFLENLLNILDKRIPK  518
             LF     N    + ++  S+L +E LL +Q       VG F     LE+L++IL+KR  K
Sbjct  476   LFAAPMGNVAATYYDIEESVLILEDLLMYQFDNDEEIVGVF-----LEDLVDILEKRKAK  530

Query  519   VNTLLIAGPANSGKNFFFDAVTQAMINCGHVGNFNRFCNFPLQECNMKRILLWNEPVCEP  578
             +N++ +  P+N+GKNFFFD V    +N G +GNFN++  FPLQE   KRILLWNEP  EP
Sbjct  531   INSIFVLSPSNAGKNFFFDCVVHFFLNFGLIGNFNKYVGFPLQETVHKRILLWNEPNAEP  590

Query  579   SAVETIKMLFAGDPCPAKIKYKNDATIMRTPVIVLTNNNPFPNDPPFNSRMIKYTWRQSP  638
             +A ET+KML  GD C  ++K+++DAT+ RTPVI+L+NN+ FP D  F +RMIKY W+ +
Sbjct  591   AAFETLKMLLGGDQCVVRVKFQSDATVGRTPVIILSNNDIFPRDAAFRNRMIKYEWKTAG  650

Query  639   FLAEIHKKPHPLALFYVLAKYNLIDT  664
             +L + +KKPH +A + +L KYN+ +T
Sbjct  651   YLEKYNKKPHLIAFYKLLLKYNIFNT  676
```

putative nonstructural protein 1 [Ambidensovirus sp.]: 63% coverage, 36.09% identity

```
Sequence ID: XBS25898.1 Length: 513
Identities     Positives      Gaps
166/460(36%)   245/460(53%)   15/460(3%)

Query  211   RRYILHDIYRNTISGRFRE-NDRGVVPKDTGNSSSAWTFVVFQHGQHVHVIHKCRYERSW  269
             RR ILHD+Y  T    F     RGV  K+   +     F V  HG H HV+H C Y
Sbjct  60    RRRILHDVYEKTNEAGFSGLVGRGVYQKEHRGA----IFCVCSHGNHWHVVHDCTYTNHT  115

Query  270   CSCALTKKAEER--FGRRIGRHTLRGSRITEGHLEHLANYIQKGRRRLLQAFLAGRERRL  327
             C CA     + R   GRR  R  +  +  T  H  +LA Y +K  R + Q  L+
Sbjct  116   CRCACVDIWKSRGGVGRRYSRRVVASNEYTIDHWVNLAIYFEKEGRSISQLDLSSVRCIP  175

Query  328   SRQAGLLQDTQSSQSGKGELVEGGRLPEHISSFLRGTCRKQHQKNPERSSEEDSENSGEE  387
             S +A  +Q  +  ++GK +LVEG R   +      G       +    +SE  S ++ E
Sbjct  176   SSEARYIQLLEDLRAGKEKLVEGSRSSLYFHYEQPGG---PDSRFASEASEAGSYSNFES  232

Query  388   GGVPAPTKREKIIQFLQSCVITPFSRIFQSQLYLFDKSLYFITPESMSCQLAILQHTHTI  447
                    K ++++ FL+   I+P ++IF   +++  K  +F    S+  Q  +  H
Sbjct  233   CRGEKRGKGDQLVLFLRQFAISPITQIFNLSVWMTSKYKFFNRNSSLM-QNCMKIHGSFY  291

Query  448   NKMTVQQIFQYQLNIPFSKLFYNC--DNPEDYFLNLSASILCIETLLKHQV--GSFGFVN  503
             N++T++++  +  ++    + +N    N  +Y+  +  S+  ++ L+K Q        +
Sbjct  292   NELTIEEMHTHFKSLEAQNVMFNAPHGNALEYYYTVEESVFILDELIKFQFRNDEARIME  351

Query  504   FLENLLNILDKRIPKVNTLLIAGPANSGKNFFFDAVTQAMINCGHVGNFNRFCNFPLQEC  563
             F   L +++DKR+PK NTL +  P NSGKNFFFD V   +IN G +GNFNR+C+FPL EC
Sbjct  352   FYTALFDVVDKRVPKKNTLFVLSPPNSGKNFFFDCVIHYLINFGIMGNFNRYCSFPLMEC  411

Query  564   NMKRILLWNEPVCEPSAVETIKMLFAGDPCPAKIKYKNDATIMRTPVIVLTNNNPFPNDP  623
               KRI++WNEP+ EPSA ET+K +F GD C AKIK++ DA I RTPVIVL+NN+ FP D
Sbjct  412   VDKRIIMWNEPLMEPSATETLKCIFGGDTCNAKIKFQGDAVIPRTPVIVLSNNDVFPKDE  471

Query  624   PFNSRMIKYTWRQSPFLAEIHKKPHPLALFYVLAKYNLID  663
              F SRM  + WR + FL    KKPHPLA +Y+   YN ++
Sbjct  472   AFRSRMFNFEWRPAIFLKNYKKKPHPLAFYYLYVYYNKVN  511
```

non-structural protein 1 [Diaphorina citri densovirus]: 59% coverage, 34.93% identity

```
Sequence ID: YP_009256211.1 Length: 679
Identities     Positives      Gaps
164/468(35%)   245/468(52%)   30/468(6%)

Query  214   ILHDIYRNTISGRFREN-DRGVVPK----DTGNSSSAWTFVVFQHGQHVHVIHKCRYERS  268
             ILHDIY    S R  E+ DR +        +GN  +A  FV F H  H+HV+H C Y  S
Sbjct  219   ILHDIYLLPDSQRNMEHLDRAIRGACRNYTSGNRQAALQFVCF-HKTHLHVVHDCTYTSS  277

Query  269   WCSCALTKKAEERFG-RRIGRHTLRGSRITEGHLEHLANYIQKGRRRLLQAFLAGRERRL  327
              C CA T+  ++  G R   R  +R  R     + +   Y QK  R+ L   + GR  R
Sbjct  278   TCRCAFTRNIQDELGVRYTKRWVIRQYRFGSERVYNTLLYFQKTGRQALYIQIGGRIWRH  337
```

```
Query  328  SRQAG---LLQDTQSSQSG---KGELVEGGRLPEHISSFLRGTC-RKQHQKNPERSSEED  380
              + G   L       Q G   +GE  + G L    + F  G C R  H+ N     E
Sbjct  338  GCEIGPNPLFGGGDKVQEGLVERGEFADNGDLS---AEFGIGDCDRGLHESNHGPLPEST  394

Query  381  SENSGEEGGVPAPTKREKIIQFLQSCVITPFSRIFQSQLYLFDKSLYFITPESMSCQLAI  440
            S  + E   +P      + I+ +L+      P + I Q++ +   +S +        +  L
Sbjct  395  SGTTVERYSLP-----KAILVWLRQHPAAPLTSILQTRHW--SESKFGTKVLRTAPLLTN  447

Query  441  LQHTHTINKM--TVQQIFQYQLNIPFSKLFYNC--DNPEDYFLNLSASILCIETLLKHQV  496
            L + + +  M  ++ ++F+   ++  + L +      N ++ +  L  S++ +E LL +Q
Sbjct  448  LFNNYALELMDASMVELFERYSSLEHTNLIFAAPLGNIQETYYTLDESVVILEKLLLYQY  507

Query  497  GSFGFVN--FLENLLNILDKRIPKVNTLLIAGPANSGKNFFFDAVTQAMINCGHVGNFNR  554
                +   FL++L ++L+KRI K NT  +  P+N+GKNFFFD +    +N G +GNFN+
Sbjct  508  RDDTEIVEIFLQDLYDVLEKRIAKKNTFFVLSPSNAGKNFFFDCIVHFYLNFGLIGNFNK  567

Query  555  FCNFPLQECNMKRILLWNEPVCEPSAVETIKMLFAGDPCPAKIKYKNDATIMRTPVIVLT  614
            + NFPLQEC  KRILLWNEP  EPSA ET+K L  GD    ++KY+ DAT+ RTPVI+L+
Sbjct  568  YQNFPLQECVHKRILLWNEPQAEPSAFETLKTLLGGDQTVVRVKYQGDATVGRTPVIILS  627

Query  615  NNNPFPNDPPFNSRMIKYTWRQSPFLAEIHKKPHPLALFYVLAKYNLI  662
            N+N FPND  F +RM  Y WR +P+LA+  KKPHP+A + +  KY +I
Sbjct  628  NHNIFPNDAAFVNRMFVYEWRTAPYLAQYRKKPHPMATYLLFKKYKII  675
```

>BtaDV_NS2 332 amino acids

MSSKKSRKTRQTSPETEMLSNLSEVDKQKKVHILEKDPPEPQAGPSLEMTETRKQEIQDFIDRSPLLTAIQDYSMEHPLGLLPLPRLMSEFVGVIEANGLTNISQEQKVLWREAQIATKKVLKPFLSKNLKAYSVQNLADWIITEGTYYTTFTEIQFPGAFGKMIGESYPRTQATHHR**H**G**H**SSSSSTA
SMSTSSTSADTKGRGARAHSRRKRKSDSDDELEDILYEAVGSQKATSNISQIIYKKGDAGSFKLSSPGEKGDYLVKLDFYKTRNLRRVAKENWWKEADYRSTFLLSYAEPAANSIKKILKDQVKKIAKIPERKVEYLPQQKGRK

MAG: hypothetical protein 1 [Periparus ater ambidensovirus]

```
Sequence ID: QTE03816.1 Length: 435 Number of Matches: 1
Identities     Positives      Gaps
54/174(31%) 79/174(45%) 22/174(12%)

Query  155  IQFPGAFGKMIGESYPRTQATHHRHGHSSSSSTASMSTSSTSADTKGRGARAHSRRKRKS  214
            +QF G  G ++ E            H    S +T   STS ++      R        + + S
Sbjct  270  VQFQGHPGSILKE-----------HLTLKSKTTLKNSTSGSAK----RPKVVIEEKAQTS  314

Query  215  DSDDELEDILYEAVGSQK---ATSNISQIIYKKGDAGSFKLSSPGEKGDYLVKLDFYKTR  271
            DSDD LED+L   V  Q    +   ISQ I  K        L SPGE+G   VKL+ +
Sbjct  315  DSDD-LEDMLDGVVQQQCLRPSIGCISQNICLKHQEQLITLKSPGERGTKTVKLEIFPYS  373

Query  272  NLRRVAKENWWKEADYRSTFLLSYAE--PAANSIKKILKDQVKKIAKIPERKVE  323
            +  R  K  WWK A  RS F L+Y++  P A  + ++ K+  K+I +    ++E
Sbjct  374  DCLRKPKNEWWKTALLRSQF-LTYSDDSPVAKLVDQLTKEIFKQIRQTKNVEIE  426
```

MAG: nonstructural protein [Phylloscopus inornatus ambidensovirus]

```
Sequence ID: QVW56838.1 Length: 360 Number of Matches: 1
Identities     Positives      Gaps
53/180(29%)    79/180(43%)    5/180(2%)

Query  115  QIATKKVLKPFLSKNLKAYSVQNLADWIITEGTYYTTFTEIQFPGAFGKMIGESYPRTQA  174
            Q+A K++LK    K +            +  E  YYT FT           ++ +++   T
Sbjct  140  QMAVKEMLKIMYKKEISMTCSFIRFKTLCREEKYYTIFTHETLTADETDLLVDAWESTT-  198

Query  175  THHRHGHSSSSSTASMSTSSTSADTKGRGARAHSRRKRKSDSDDELEDILYEAVGSQKAT  234
                      SS   T ++ T ST+A T    A         K+ S D  ED L+    S   T
Sbjct  199  ---EEISSSLPRTETIGTLSTTATTAAAPADVQESTTSKTISQDSGED-LFTRANSPCDT  254

Query  235  SNISQIIYKKGDAGSFKLSSPGEKGDYLVKLDFYKTRNLRRVAKENWWKEADYRSTFLLS  294
              ISQ I K+ +       +SPGE+G  ++KLD    ++    + K+NWW+ A  RS FL S
Sbjct  255  GTISQHISKRVEDSWISFTSPGERGCNIIKLDVIPFKDAVTLDKQNWWRVATMRSQFLTS  314
```

putative nonstructural protein [Diaphorina citri densovirus]

```
Sequence ID: YP_009256210.1 Length: 432
Identities     Positives      Gaps
49/149(33%)    65/149(43%)    9/149(6%)

Query  149  YTTFTEIQFPGAFGKMIGESY----PRTQATHHRHGHSSSSSTASMSTSSTSADTKGRGA  204
            YTTF     P     ++ E Y       TQA    +   S S ST   STS T+  T    A
Sbjct  233  YTTFICYPIPKEIWSILIERYEELAATTQAVTDKQLFSLSVSTKPTSTSCTTVPTLLPRA  292

Query  205  RAHSRRKRKSDSDDELEDI-LYEAVGSQKATSNISQIIYKK-GD-AGSFKLSSPGEKGDY  261
                S    K+ S      +  L++ + S +  S I   I KK GD   +FK  S G  GD
Sbjct  293  DVPSPGTSKTSSACATPNGGLFDNIDSDQNGSTIPCSISKKLGDKLCTFK--SEGAFGDT  350

Query  262  LVKLDFYKTRNLRRVAKENWWKEADYRST  290
            +VKLD            +K++WW EA  R+T
Sbjct  351  VVKLDLIPYSAAATKSKKDWWSEASLRTT  379
```

NS2 [Myzus persicae densovirus 2]

```
Sequence ID: WDS 38637.1 Length: 366
Identities     Positives      Gaps
37/165(22%)    73/165(44%)    4/165(2%)

Query  157  FPGAFGKMIG--ESYPRTQATHHRHGHSSSSSTASMSTSSTSADT--KGRGARAHSRRKR  212
            +P   G+++    +Y     +  ++   +SS++TA+ STS T   T         ++S
Sbjct  168  YPEEIGQLMELLRTYAGVSQSECQNACTSSANTATTSTSYTPVTTPRAAVDVVSYSTPLL  227

Query  213  KSDSDDELEDILYEAVGSQKATSNISQIIYKKGDAGSFKLSSPGEKGDYLVKLDFYKTRN  272
             +   DD+   + + E+   QK T  +S    Y       S K ++      DY+V +  Y+
Sbjct  228  SNYVDDDAFERMLESSNYQKETGPVSFNTYVHRPGMSKKWAASTLMEDYVVDIHIYQKDQ  287

Query  273  LRRVAKENWWKEADYRSTFLLSYAEPAANSIKKILKDQVKKIAKI  317
            ++ V   +WWK A  R+     +    P     K I ++  +++ K+
Sbjct  288  IQNVDPNDWWKHAFCRARGSFNLNSPYVKMAKTIQQEVFRRMTKM  332
```

unnamed protein product [Bemisia tabaci]

```
Sequence ID: CAH0395645.1 Length: 352 Number of Matches: 1
Identities     Positives      Gaps
71/272(26%)    125/272(45%)   20/272(7%)

Query  61   IDRSPLLTAIQDYSMEHPLGLLPLPRLMSEFVGVIEA----NGLTNISQEQKV-LWREAQ  115
            ++  PL   +Q+       GL PLPR++ E  G+I       + L +  Q+  + + R  +
Sbjct  70   VETHPLAQLLQELISFADEGLAPLPRVLDELRGMIMHPMFYSDLEDPQQKDLLKIMRAFE  129

Query  116  IATKKVLKPFLSKNLKAYSVQNLADWIITEGTYYTTFTEIQFPGAFGKMIGESYPRTQAT  175
            +  KK+ +    K L+A   ++L + ++ +  +YTTFT I           + +S+   +
Sbjct  130  LKMKKLKQQ--GKQLRACGWRDL-EQLMEKEPFYTTFTRIVLEEPVKTYVCDSFLNLE--  184

Query  176  HHRHGHSSSSSTASMSTSSTSADTKGRGARAHSRRKRKSDSDDELEDILYEAVGSQKATS  235
                     S   T +  T  T A T    A A SRR   ++S+    D L     S
Sbjct  185  ---RELISPPDTETTCTYVTHATTSTAHADATSRRSSGANSESGYLDHLLAHETSASKDG  241

Query  236  NISQIIYKKGDAGSFKLSSPGEKGDYLVKLDFYKTRNLRRVAKENWWKEADYRSTFLLSY  295
             ISQ I  +    S +L++PGE G  +VK++ Y    +     + NWW+ A  R+  L+ +
Sbjct  242  VISQFICHQDRGTSLRLTTPGEIGYEIVKIEVYPFSEVVNEDRMNWWRSARTRA--LIHF  299

Query  296  AEP---AANSIKKILKDQVKKIAKI--PERKV  322
              P      +IK+I+  + K+I+++   E+KV
Sbjct  300  NSPLDTELQTIKRIIDRKAKEISELEGTEKKV  331
```

>BtaDV_VP 749 amino acids

MPLWPKHNYLGPGNDLINGDPEDEDDEVALQHDISYEFAKSEEEIFESDKRAISDFANIAVNDLKNWDINTGAIAGTGLLFLKHNLESAIGTTLYPWHPDGGDVSDMNKTTYALREKTKSLVYQNLKRLGKVHTSYREFWRSDSGANLAQTIYSDFANHGEHWQSLIADGTLDIDRVSNGLVSQYVST
YSRNSTPSIATSSDTPPQSPVPGPSTSGAPRQEAGDFGNSEGGKRKAEESSILDNSSNKRVATSSSGSSSSSLSASSTNQSMEVDGVTDPKGKRGTGAGISKVTPPVSLNKSSTHSGNKIVITKSRMLFCHAIAPAQLSVNAELNIGRNVSAKVLGLAYIPVDYLPFYLTEQEYNAIPDNSRVLRVGC
KITPVGCRTAFDTGTSTSGVATNEWIAVGRSVVGLNLKIPIYNVHLSTKADEPMIPTSISRLDHTTMQNSWYLNNDICNVLGYPTQINEYAAYIFNTNSEKNTTYKKDVDYSYHCGGNPRLDKVIERFMFNAHIGSPIIDYEYSPQNAMIKKHKIVNLPYPTCSQVILNGENPGPWNINIDGWASDVD
HDKNKGRAYYKSVNSATSDMYVPFECKYNRMLESGPYSHNFKAAGGENIQPQVHIGLMPTPNINPANSSVSFQNSAAYFEVECYIEISSNVDSAFTGGTDAVMWNDDITIYYHDTVPLHDGLSVGKKRVKPSSASVYAGETSKHSKMPRSRLQAEASEKQSAVENTQYYKDIMKIKALDVENKKK

MAG: VP4 [Phylloscopus inornatus ambidensovirus]

```
Sequence ID: QVW56836.1 Length: 559 Number of Matches: 1
Identities     Positives      Gaps
141/430(33%)   213/430(49%)   45/430(10%)

Query  267  NQSMEVD-----GVTDPKGKRGTGAGISKVTPPVSLNKSSTHSGNKIVITKSRMLFCHAI  321
            NQ+M++D       +    G RG      S  +P V L+KS+    G  +   TKSR  F +A
Sbjct  106  NQNMDIDPPLEGEASGSSGPRGAHGASSSGSPLVPLSKSTASDGWSMNFTKSRTAFAYAY  165

Query  322  APAQLSVNAELNIGRNVSAKVLGLAYIPVDYLPFYLTEQEYNAIPDNSRVLRVGCKITPV  381
              AQL++ + L  G   S      +AY+PVD+LPFYL+ QE++++P ++R+  V CKI  +
Sbjct  166  CNAQLNLQSGLFDGVCTS-----MAYVPVDWLPFYLSPQEFSSLPPHARITDVWCKIKVI  220

Query  382  GCRTAFDTGTSTSGVATNEWIAVGRSVVGLNLKIPIYNVHLSTKADEPMIPTSISRLDHT  441
```

```
            G R+AFDTG++ S  AT+E+  +  + VGLN K  I NV  +T A  PM+PT    +  T
Sbjct  221  GVRSAFDTGSTLSATATSEYCPILHTCVGLNNKFNISNVKYTTAAAAPMVPTGYGAISGT  280

Query  442  TMQNSWYLNNDICNVLGYPTQINEYAAYIFNTNSEKNTT--YKKDVDYSYHCGGNPRLDK  499
            +M   +Y N+   +V+  P     YA +++N     NT   Y+K   Y  H G   R+DK
Sbjct  281  SMAEKYY-NHATSSVMCVPRSATGYAMFMWNKEVYANTVENYEK---YQPHLGKMARIDK  336

Query  500  VIERFMFNAHIGSPIIDYEYSPQNAMIKKHKIVNLPYPTCSQVILNGENPGP------WN  553
             +  ++ N+ IG  +IDY Y  ++  I   KI         Q+    +N G       WN
Sbjct  337  YVNAYLMNSAIGEDVIDYRYRVKSGYINNPKITYQYGMGARQIPF--QNRGSTLREVWWN  394

Query  554  INIDGWASDVDHDKNKGRAYYKSVNSATSDMYVPFECKYNRMLESGPYSHNFKAAGG--E  611
             N   W       +  G AY++          Y      Y++ LE    S +F A G
Sbjct  395  NNDKRW-------EVSGTAYHEK-------HYQQATFSYSQSLEK---SRSFTADGAALH  437

Query  612  NI--QPQVHIGLMPTPNINPANSSVSFQNSAAYFEVECYIEISSNVDSAFTGGTDAVMWN  669
            N   QPQVHIG+   P +NPA  S  F N+  Y+ + C   +  +++S +T GT A   +
Sbjct  438  NTPNQPQVHIGMQAIPQLNPATESTGFMNACVYYHITCGATVVGDINSDWTHGTAACHPS  497

Query  670  DDITIYYHDT  679
            + + I Y  T
Sbjct  498  NAVFINYQST  507
```

MAG: putative structural protein VP1 [Ambidensovirus sp.]

Sequence ID: XBS25899.1Length: 678 Number of Matches: 1

```
Identities     Positives      Gaps
185/683(27%)   304/683(44%)   68/683(9%)

Query  1    MPLWPKHNYLGPGNDLINGDPEDEDDEVALQHDISYEFAKSEEEIFESDKRAISDFANIA  60
            M   P +NYLGPGN L +G+P DE D +A  HD  Y      + I  +D+  +   + +
Sbjct  1    MATVPGYNYLGPGNKLNSGEPVDELDLIAQIHDYDYA-TLPVDRIHRADQIFLERVSKLE  59

Query  61   VNDLKNWDINTGAIAGTGLLFLKHNLESAIGTTLYPWHPDGGDVSDMNKTTYALREKTKS  120
             N L       G   G   +  K  LE+  G  LY    G D  DM K TY     +
Sbjct  60   NNSL-------GKYIGEAGIEAKQELENYTGV-LY----GGIDPEDM-KRTYNFGNELYK  106

Query  121  LVYQNLKRLGKVHTSYREFWRSDSGANLAQTIYSDFANHGEHWQSLIADGTLDIDRVSNG  180
            L  Q +         YR+F  +     + +  YSD    G++  S    G L+  R  +
Sbjct  107  LRQQEI---------YRKFREA-----VNRGEYSDL---GDYRSSAEYKGILENFRKGSD  149

Query  181  LVSQYVSTYSRNSTPSIATSSDTPPQSPVPGPSTSGAPRQEAG-----DFGNSEGGKRKA  235
               +  + +      S A S    P  P PGPS++  P    G     D  + +
Sbjct  150  NYKKLKTDFDARRGNSDAASR---PVDPQPGPSSAVDPDPNQGNEIADDLPDFDWDNFLT  206

Query  236  EESSILDNSSNKRVATSSSGSSSSSLSASSTNQSMEVDGVTDPKGKRGTGAGISKVTPPV  295
            E  + +      ++  +  G  S +    +T          T P    G   G +  +P +
Sbjct  207  EFDAQIGGVGEAQLPGAMEGIQSGTDRGPNTGSRS-----TAPTETMGAATGGAVSSPWI  261

Query  296  SLNKSSTHSGNKIV-ITKSRMLFCHAIAPAQLSVNAELNIGRNVSAKVLG-LAYIPVDYL  353
              ++     G+ +V  +KSR+L+ +  A          NI + ++  V    +A IPVDY+
Sbjct  262  PRSRPD---GSVVVSFSKSRILYSYGFATK--------NIQKTLTDHVTTPMALIPVDYV  310

Query  354  PFYLTEQEYNAIPDNSRVLRVGCKITPVGCRTAFDTGTSTSGVATNEWIAVGRSVVGLNL  413
            PFYL+  E++ +P  +++ +V C++ P+G RTAFD GT+ SG AT+E+I +G S VG+N+
Sbjct  311  PFYLSRAEFDQLPVGAKITQVSCRVYPIGTRTAFDVGTTLSGSATSEYIPIGLSCVGMNI  370

Query  414  KIPIYNVHLSTKADEPMIPTSISRLDHTTMQNSWYLNNDICNVLGYPTQINEYAAYIFNT  473
                 N+   T A EPM PT  + +  + +   +Y +   C+ L   P  I+EY  + +N
Sbjct  371  DFYGKNMKYETGATEPMKPTGTTDIVPSEITGKYYDHVASCS-LNVPRSISEYFVHEWNR  429

Query  474  NSEKNTTYKKDVDYSYHCGGNPRLDKVIERFMFNAHIGSPIIDYEYSPQNAMIKKHKIVN  533
            +   + T  K   Y  H  G  R D+ ++ F+ NA +  P+++Y Y P+N ++K  K
Sbjct  430  SGNPDVT--KYPQYQVHNAGVVRSDEKVKAFLLNALVNQPVVEYYYIPKNGIVKSGKDHY  487

Query  534  LPYPTCSQVILNGE--NPGPWNINIDGWASDVDHDKNKGRAYYKSVNSATSDMYVPFECK  591
            +PY +    +L  E        +       S         G    K  +   ++ + V +E +
Sbjct  488  VPY-SRKDFLLESEYYRARSHTLQFFQTGSGTPDLGVVGLGREKQPSVMSNQVAVSYERQ  546

Query  592  YNRMLESGPYSHNFKAAGGENIQPQVHIGLMPTPNINPANSSVSFQNSAAYFEVECYIEI  651
                    P+S       G + QPQVH+G+  TP +NPA    +F NS AY+ VEC   +
Sbjct  547  IENYGSFSPHS----GTTGFHAQPQVHVGIQATPQLNPATEGENFLNSCAYWRVECSCTV  602

Query  652  SSNVDSAFTGGTDAVMWNDDITI  674
              +++S+FT G   + W  +++
Sbjct  603  KFSLNSSFTKGF-PISWPREVSF  624
```

MAG: capsid protein [Emberiza pusilla parvoviridae sp.]

Sequence ID: QTE03973.1 Length: 639 Number of Matches: 1

```
Identities     Positives      Gaps
194/695(28%)   298/695(42%)   98/695(14%)

Query  3    LWPKHNYLGPGNDLINGDPEDEDDEVALQHDISYEFAKSEEEIFESDKRAISDFANIAVN  62
            L P H Y+GPGN L +G P D DD +A  HD +YE+      + ESD+    DF     N
Sbjct  2    LLPFHKYVGPGNKLESGKPFDSDDVIARLHDYAYEYGI----VHESDRHFAVDFF---WN  54

Query  63   DLKNWDINTGAIAGTGLLFLKHNLESAIGTTLYPWHPDGGDVSDMNKTTYALREKTKSLV  122
             + N++I++  + G+  L+ K+ +E   G  +YP        S   +TT+A   ++
Sbjct  55   SVYNYNIHS--VIGSLGLYFKYYVEDLYGQ-IYPASVMAPGTSTTYRTTHAGHQR-----  106

Query  123  YQNLKRLGKVHTSYREFWRSDSGANLAQTIYSDFANHGEHWQSL--IADGTLDIDRVSNG  180
            Y NL++       Y  F R + G +               WQ    +  G   I
Sbjct  107  YANLQKYWA--ERYHNFKRRNPGVS--------------WQEYKKLVRGQTPIPEAMEA  149

Query  181  LVSQYVSTYSRNSTPSIATSSDTPPQSPVPGPSTSGAPRQEAGDFGNSEGGKRKAEESSI  240
            +      +    +   TS+  P      G S              S G KR
Sbjct  150  VAGSRRPSTRSSRGSTTTTSNAPPYHQDFMGES--------------SRGSKRSFHPDDY  195

Query  241  LDNSSNKRVATSSSGSSSSSLSASSTNQSMEVDGVTDPKGKRGTGAGIS--------KVT  292
            +D S +     S  GSSS +          ME+D     +G  G  A  +        K+T
Sbjct  196  VDESFD----VSQLGSSSVA--------PMELDIENVNRGGGGAQATSTNFDSGSNRKLT  243

Query  293  PPVSLNKSSTHSGNKIVITKSRMLFCHAIAPAQLSVNAELNIGRNVSAKVLGLAYIPVDY  352
             P S+ +   H     +  TKSR+ +C+  +      +      ++G++      LA +PV+
Sbjct  244  IPRSVQQLVFH----MNFTKSRIFYCYGFNFTDIPKKNTSDLGKSYVQFCTPLANLPVEM  299

Query  353  LPFYLTEQEYNAIPDN--SRVLRVGCKITPVGCRTAFDTGTSTSGVATNEWIAVGRSVVG  410
            L FY+   EYN I     S +    C + P G RTAFDTG++ SG AT+E  A+G S +G
Sbjct  300  LGFYVDNYEYNLISHRGTSVIKHCSCTVVPQGLRTAFDTGSTLSGTATSEHCAIGISAIG  359

Query  411  LNLKIP-IYNVHLSTKADEPMIPTSISRLDHTTMQNSWYL-----NNDICNVLGYPTQIN  464
            +N     I +    T AD PM+PTS+    +    + + +Y       N +I  ++G P  IN
Sbjct  360  INRAYDNIVHGQYDTNADAPMVPTSVKNTNEEKLLDIFYGQKKMDNANIPMIVGLPRHIN  419

Query  465  EYAAYIFNTNSEKNTTYKKDVDYSYHCGGNPRLDKVIERFMFNAHIGSPIIDYEYSPQNA  524
             YA + FN  +   TT       Y+ H  G P LD+ +ERF F   +G+ I D+ YSP+N
Sbjct  420  NYAVFAFNMETTPTTT----GGYAIHDEGYPVLDRHLERFNFVPTVGNVIADWNYSPKNG  475

Query  525  MIKKHKIVNLPYPTCSQVILNGENPGPWNINIDGWASD---VDHDKNKGRAYYKSVNSAT  581
            +I+  K  +  Y        ++G       N    A +  V+   N        + +
Sbjct  476  IIQC-KAYDSNYTQLQSDTVSGFFNNNLVTNTTIAAKEDGVVNLATNVKAQSFSNKLVHL  534

Query  582  SDMYVPFECKYNRMLESGPYSHNFKAAGGENIQPQVHIGLMPTPNINPANSSVSFQNSAA  641
              MY+  E   ++ L  GP +   K        PQ+HIG++P P +NP+    SFQN+AA
Sbjct  535  QKMYI--ENTRSKFLFGGPTA--LKTV------PQLHIGILPVPQLNPSKDEKSFQNTAA  584

Query  642  YFEVECYIEISSNVDSAFTGGTDAVMWNDDITIYY  676
            YF V C I +  N++S +          D+I I Y
Sbjct  585  YFSVVCNISVEINLNSIYALSNTPHAGVDEIVINY  619
```

VP4 [Bactericera trigonica densovirus]

Sequence ID: YP_010802687.1Length: 573 Number of Matches: 1

```
Identities     Positives      Gaps
136/466(29%)   221/466(47%)   47/466(10%)

Query  211  GPSTSGAPRQEAGDFGNSEGGKRKAEESSILDNSSNKRVATSSSGSSSSSLSASSTNQSM  270
            G STSG+    AG F  +     +  + + LD         T  S ++ S  S ++ ++ M
Sbjct  76   GASTSGS----AGRFDATGDEHSEQSKRARLDGDDTIDSDTLPSDAADSLQSGTTMSEPM  131

Query  271  EVDGV---TDPKGKRGTGAGISKVTPPVSLNKSSTHSGNKIVITKSRMLFCHAIAPAQLS  327
            + D +   T   G RG  +G +     V L  SS   +  +   +KSR+ + +A A  +L
Sbjct  132  DEDAILQDTSGSGGRGPTSGRTTGGATVPLGLSSGDATFQATYSKSRIWYSYAFANKKL-  190
```

```
Query  328   VNAELNIGRNVSAKVLGLAYIPVDYLPFYLTEQEYNAIPD-NSRVLRVGCKITPVGCRTA  386
                +L+  + +   V  LA+IPVD+LPFY++  E+ +IPD N  + +V CK+  +G R+
Sbjct  191   ---DLDSTKTLEGIVTPLAFIPVDFLPFYMSPAEFASIPDRNVWIDKVTCKVRVLGVRSG  247

Query  387   FDTGTSTSGVATNEWIAVGRSVVGLNLKIPIYNVHLSTKADEPMIPTSISRLDHTTMQNS  446
             FD G + +G AT+E+  +  + VGLN +I I N  +     +PMIPT +  +D    +
Sbjct  248   FDIGATLTGTATSEYCPILHTCVGLNNEIHIMNKSVEANTTQPMIPTGVRDIDLAEYKKK  307

Query  447   WYLNNDICNVLGYPTQINEYAAYIFNTNSEKNTTYKKDVDYSYHCGGNPRLDKVIERFMF  506
             WY   +  NV+G P   + YA   +N+++   T  +K      H  GNPRLD+ ++  M
Sbjct  308   WY-TGETSNVIGTPRSNSVYAIPYWNSSTTSATEVQK------HTWGNPRLDRFVKTHML  360

Query  507   NAHIGSPIIDYEYSPQNAMIKKHKIVNLPYPTCSQVILNGENPGPWNINIDGWASDVDHD  566
              + IG  I DY YSP+ A I+    + +P                N  ++  ++    D
Sbjct  361   TSTIGEEIFDYSYSPRFAPIRPQDQLVIP----------------NHRVNEASTFQSRD  403

Query  567   KNKGRAYYKSVNSATSDM----YVPFECK----YNRMLES-GPYSHNFKAAGGEN-IQPQ  616
               + +  Y S      D      VP  C     Y +++E    Y     + GGE   QPQ
Sbjct  404   LWQAKCAYGSGKFTMKDYSWPEMVPL-CDTAFVYAQLIEKYDMYMPGTGSVGGEQRAQPQ  462

Query  617   VHIGLMPTPNINPANSSVSFQNSAAYFEVECYIEISSNVDSAFTGG  662
             +HIG+   P +NP     SF N+  Y+ VEC I ++   ++ +T G
Sbjct  463   LHIGMSAIPQLNPTTDLTSFVNACVYYSVECSIHLTGKREAYWTEG  508
```

putative structural protein [Diaphorina citri densovirus]

Sequence ID: YP_009256212.1 Length: 588 Number of Matches: 1

```
Identities     Positives      Gaps
136/455(30%)   221/455(48%)   47/455(10%)

Query  257   SSSSLSASSTN-QSMEVDGVTD-------PKGKRGTGAGISKVTPPVSLNKSSTHSGNKI  308
             S+S L  S TN ++M  D  D        P G  G     S+   P ++  +++H   +
Sbjct  115   SASKLPRSDTNSETMPADERMDTTQYGDPPTGGSGQQPRTSRGGGPSAVPLNNSHGSQQW  174

Query  309   V--ITKSRMLFCHAIAPAQLSVNAELNIGRNVSAKVLGLAYIPVDYLPFYLTEQEYNAIP  366
                 +KSR+ + +  +     V + L+  +++   V  L++IPVDYLPFYL   EY  +P
Sbjct  175   TASFSKSRIWYSYGYS----QVTSYLDTKQSLLGTVTPLSFIPVDYLPFYLDSAEYATVP  230

Query  367   DNSRVLRVGCKITPVGCRTAFDTGTSTSGVATNEWIAVGRSVVGLNLKIPIYNVHLSTKA  426
              N+ V RV C++  +G R+ FD G + +GVAT+E+  +  + +GLN  + I N  + +++
Sbjct  231   PNTWVKRVTCQVRVLGVRSGFDIGATVTGVATSEYCPILLTAIGLNNNVHIMNKKIKSES  290

Query  427   DEPMIPTSISRLDHTTMQNSWYLNNDICNVLGYPTQINEYAAYIFNTNSEK---NTTYKK  483
               PMIPT +  +  T   + WY+ +    V   P   + YA    NT+  K   +   KK
Sbjct  291   TAPMIPTGLEDISLTDYMDKWYV-DPASGVTCVPRSNSVYAVPYRNTSMAKPDWDDATKK  349

Query  484   DVDYSYHCGGNPRLDKVIERFMFNAHIGSPIIDYEYSPQNAMIKKHKIVNLPYPTCSQ-V  542
             DV    H  GN RLDK ++  + ++ +G  ++DYEY P   +IK+   + +PY   S  +
Sbjct  350   DV--LLHDAGNFRLDKFVKSHLLHSTVGETVVDYEYEPHMGVIKEPDPLCIPYHRASDGL  407

Query  543   ILNGENPGPWNINIDGWASDVDHDKNKGRAYYKSV--NSATSDMYVPFECKYNRMLES-G  599
             ++N        +NI     DV   + K        V  + A S  +       Y+  +E  G
Sbjct  408   MMNA-------MNIVDATCDVSTKEYKLTQKEPLVKHHMAKSKFF------YHSQIEKYG  454

Query  600   PYSHNFKAAG-GENIQPQVHIGLMPTPNINPANSSVSFQNSAAYFEVECYIEISSNVDSA  658
              ++  + A    +N+QPQVHIGL   P +NPA     +F N   Y+ V+C IE+ S   +
Sbjct  455   IFAPRYGALNPRQNVQPQVHIGLSAIPQLNPATEHKNFVNCCVYYAVDCSIELESRRTTE  514

Query  659   FTGG-----TDAVMWNDDITIYY---HDTVPLHDG  685
             +TG      TDA +++      YY   H    + DG
Sbjct  515   WTGKPTQPETDA-LFSSHANYYYEQPHSVFGMWDG  548
```

uncharacterized protein LOC118740163 [Rhagoletis pomonella]

Sequence ID: XP_036327552.1 Length: 673 Number of Matches: 1

```
Identities     Positives      Gaps
195/697(28%)   319/697(45%)   44/697(6%)

Query  5     PKHNYLGPGNDLINGDPEDEDDEVALQHDISYEFAKSEEEIFESDKRAISDFANIAVNDL  64
             P + YLGPGN L  G P +E D +A  HDI Y  AK  ++I E+D  AI  F  I
Sbjct  5     PGYKYLGPGNKLNKGVPINEVDLIAQIHDIDYALAKCGKDIREADLEAIRQFHKI-----  59

Query  65    KNWDINTGAIAGTGLLFLKHNLESAIGTTLYP---WHPDGGDVSDMNKTTYALREKTKSL  121
                  + G I G   L  K+ +ES  G  LY     +   G   ++ +  Y  R++
Sbjct  60    -ENPFDFGRILGKFGLIAKYGIESITGV-LYGGGNMNKQRGKKRNLGQLLYGERQRQVHR  117

Query  122   VYQNLKRLGKVHTSYREFWRSDSGANLAQTIYSDFANHGEHWQSLIADGTLDIDRVSNGL  181
              +  LK  GK + S +EF++S+      + I  D++  G  ++ +    T  +D + +G
Sbjct  118   KFNELKSTGK-YKSLQEFYKSEE----YKEIIKDYSTGGSAYKRI---KTYILDDIPSG-  168

Query  182   VSQYVSTYSRNSTPSIATSSDTPPQSPVPGPS--TSGAPRQEAGDFGNSEGGKRKAEESS  239
                       S+N  P+   ++D P   P  G     S  P          + +         +
Sbjct  169   --------SKNP-PTEEPTTDLPSTDPGSGSHDPRSSTPVDFEDRDDDFDWDNFLDNIGN  219

Query  240   ILDNSSNKRVATSSSGSSSSSLSASSTNQSMEVDGVTDPKGKRGTGAGISKVTPPVSLNK  299
              L+++    +  +SS   S + S    N     +       G    +G G +   +P   L++
Sbjct  220   SLNSADLADLPEASSMDISQAGSGRGPNTGARGEATAPSLGASSSGNGGNNSSP--WLSR  277

Query  300   SSTHSGNKIVITKSRMLFCHAIAPAQLSVNAELNIGRNVSAKVLGLAYIPVDYLPFYLTE  359
             S     + +   KSR+++ + +A  +L      N  + +       +A IPVD+LPFY++++
Sbjct  278   SVPKRVSNLEFKKSRIMYSYGLATERLE-----NTEKTIDIFSTSMALIPVDFLPFYISQ  332

Query  360   QEYNAIPDNSRVLRVGCKITPVGCRTAFDTGTSTSGVATNEWIAVGRSVVGLNLKIPIYN  419
              E+   +P  S++   V CK+ P+G RTAFDTGT+ SG AT+E++ +G + +GLNL      N
Sbjct  333   AEFANLPFASKIESVWCKVYPLGTRTAFDTGTTLSGTATSEYVPIGMTGIGLNLDFYGQN  392

Query  420   VHLSTKADEPMIPTSISRLDHTTMQNSWYLNNDICNVLGYPTQINEYAAYIFNTNSEKNT  479
             V LS ++ +PMIPTSI ++  + +   +Y  +D C  +G P  I+EY  +  + N + N
Sbjct  393   VELSGESTKPMIPTSIKQMSASKIVRKFY-EDDACCAMGVPRSISEY--FTIHRNKDANP  449

Query  480   TYKKDVDYSYHCGGNPRLDKVIERFMFNAHIGSPIIDYEYSPQNAMIKKHKIVNLPYPTC  539
               +K   Y  +  G    D  I +F+ N  I   P+IDY+Y  +N  +   K   +P+
Sbjct  450   DPEKYPQYQVNTHGVVNFDNKINKFLVNGLINQPVIDYKYDVKNGYVMNAKYHIIPHNRR  509

Query  540   SQVILNGENPGPWNINIDGWASDVDHDKNKGRAYYKSVNSATSDMYVPFECKYNRMLESG  599
             S V        G  N  ++ +   +    KG+  +K        S +        Y R LE
Sbjct  510   SDVYCTHHTRGSKN-KLEFY--HLGDGPAKGQVGFKGKRGEVSPLNNMIANAYLRQLEIY  566

Query  600   PYSHNFKAAGGENIQPQVHIGLMPTPNINPANSSVSFQNSAAYFEVECYIEISSNVDSAF  659
               + +          QPQVH+GL  TP +NP+N     F NS  Y++V+C I IS+N  SA
Sbjct  567   GCFNIYTGTTPYYAQPQVHVGLCATPQLNPSNEDKIFLNSCCYWKVDCGITISTNSHSAM  626

Query  660   TGGTDAVMWNDDITIYYHDTVPLHDGLSVGKKRVKPS  696
             T G + + W  ++                DG ++    V P+
Sbjct  627   TKG-EQISWPREVLFKETSDYKYTDGKTLFGSSVLPT  662
```

(D) Sequence analysis of the ambidensoviruses most related to BtaDV

>KX165268_Diaphorina_citri_densovirus_DcDNV

```
AGTACTTTTGGAGTGGTTCTTTCACTCCAAACTCTTTCTAGAATGCAGCACCGACAACAGCAGCATGAGACCTAGATTTTCCTTGATTTTCCCGTGCACCGGCCGAAGGCCTAGGTAAGCACCTGGGAGGAAAATCTAGGTCTCATGCTGCTGTTGTCGGTGCTGCATTCTAGAAAGAGTTTGGAGTG
AAAGAACCACTCCAAAAGTACTTTGCGTGTTTATCTCTGATGTCATTGGTGATAAAGTCTCTAACGATGCGAACTATAAATAGAGGCGTTTCTGTTGTGTAGATCTTCAGTATGGATTGTTCAGCGGACGGAAAATTTAAACGTTTGGATTGTTTGTGTTACTTTTGTTTACTTCCAAAATTGCCAAC
ACCAGTAGTGAGTATAGTTCCGACGATGAGTTCGAACGAGGATTCGTGTCAATCTCTAAACGGCGAGGATCCGTTGAGTTCGGCTCCAGCGAGTCCGGCGACGAGTCAACATGGGCACAATTTAAGCGATTCCGAGGAGACGAATTGTACCGTCGTATTGGCACAGAATCCCCTGCCCGATACACAAG
AAGTCGAGTCAATGTTCCAAGAGGATCACCGGAACGTGTCAGTACATTTCGCGGAAGAGACAGTGACTCCGGCTCGGAAGAAGAAATCTATACTGAAACGGACGATGAAAAAGAAAACAACTCCCAAAGTGACCACGGCGAAGAAGATGTTGACACTGACTCATCAGCAACAGATATCAGTGTGGGAA
CGGCAAACCAACAACTTTATGGCGTTCCTGTTTCTCGTCTGGACTCCTATGCAGACCGTTCACCTTCTCCAGGACCTTCTACTGCATATCGCTATTGTAGCGAGGAAAACCCAAAATGTGGACCTAAAAAAGCAGTGCATCCGTTGGGGGGAAGGCTTGGCGATCGTCAAGCAAAAGCTAGGAGCCAA
TTACGAAACAGCATCGTGGGCCTCCTTCAGCGAGTGCCTCCGAAGTCTCGCATCTTACACGACATTTATCTGCTACCCGATTCCCAAAGAAATATGGAGCATCTTGATCGAGCGATACGAGGAGCTTGCCGCAACTACACAAGCGGTAACCGACAAGCAGCTCTTCAGTTTGTCTGTTTCCACAAAAC
CCACCTCCACGTCGTGCACGACTGTACCTACACTTCTTCCACGTGCAGATGTGCCTTCACCCGGAACATCCAAGACGAGCTCGGCGTGCGCTACACCAAACGGTGGGTTATTCGACAATATAGATTCGGATCAGAACGGGTCTACAATACCTTGCTCTATTTCCAAAAAACTGGGCGACAAGCTCTGT
ACATTCAAATCGGAGGGCGCATTTGGAGACACGGTTGTGAAATTGGACCTAATCCCCTATTCGGCGGCGGCGACAAAGTCCAAGAAGGATTGGTGGAGCGAGGCGAGTTTGCGGACAACGGTGACCTTTCAGCAGAATTCGGCATTGGCGATTGCGACCGAGGCCTACACGAAAGCAATCATGGACCA
CTTCCAGAGTCAACAAGTGGGACCACTGTCGAAAGATACTCACTACCAAAGGCAATCCTCGTTTGGTTACGGCAACATCCAGCCGCGCCGCTGACATCCATACTCCAAACTCGTCACTGGTCGGAGTCGAAATTCGGTACGAAAGTACTACGGACCGCGCCCCTACTCACCAATCTCTTCAACAATTA
CGCCCTTGAACTCATGGATGCTTCCATGGTCGAACTTTTCGAACGCTATTCATCACTCGAGCATACGAATTTAATTTTTGCCGCTCCACTCGGTAACATTCAGGAAACCTACTATACGTTGGACGAGTCCGTTGTAATTTTGGAAAAATTGCTACTCTATCAGTATCGTGATGATACGGAAATTGTGG
AAATTTTTCTCCAGGATCTATATGACGTACTCGAAAAACGTATAGCTAAAAAAAATACCTTTTTTGTTCTCTCCCCATCGAATGCTGGCAAAAATTTTTTCTTCGATTGCATTGTACATTTTTATTTGAATTTCGGTCTGATCGGTAATTTTAATAAGTACCAAAATTTCCCTTTACAAGAGTGTGTT
CACAAACGTATTCTTCTGTGGAACGAACCACAAGCTGAGCCTAGTGCTTTTGAAACTCTGAAAACGTTACTCGGAGGCGATCAGACCGTAGTTCGAGTGAAATATCAAGGTGATGCAACGGTGGGTCGAACACCGGTTATTATTCTCTCAAATCACAACATTTTTCCCAACGACGCTGCATTCGTCAA
TCGTATGTTTGTATACGAATGGCGAACTGCTCCGTATTTGGCACAATATCGCAAAAAACCACACCCTATGGCTACATACTTGCTTTTTAAAAAATATAAAATAATCAAACCAGAAATGTAATCAATACAATATCAAATTTATTGTTTACAATACTTCGAATTCTCTAATTAGTTCTGCTTCTTTAGTT
CTCTCTGCCAATTTTTTTTCCAGCCTTGCAGAAGTACTCGAAGACTTCTTATTTGTGGTAACCTTAATAGAACCCCCATCTCCATCCCACATACCGAACACTGAGTGCGGTTGTTCGTAATAGTAGTTGGCATGAGAAGAAAACAGAGCATCTGTTTCAGGCTGGGTAGGTTTTCCGGTCCATTCCGT
CGTTCGACGAGACTCTAATTCGATGGAACAATCTACAGCATAGTAGACACAACAATTAACAAAATTCTTATGTTCGGTAGCTGGATTCAATTGGGGTATAGCGCTTAAACCTATGTGCACTTGGGGTTGGACATTTTGACGGGGATTCAATGCCCCATAACGTGGAGCAAAAATACCGTATTTTTCGA
TTTGAGAATGATAGAAAAATTTACTCTTCGCCATATGATGTTTAACAAGGGGCTCTTTTTGCGTTAGTTTATATTCTTTGGTGCTGACGTCACATGTGGCATCTACAATATTCATAGCGTTCATCATTAGACCGTCCGAGGCGCGATGATAGGGAATACACAGAGGATCCGGTTCTTTAATAACGCCC
ATGTGAGGTTCATATTCATAGTCCACAACAGTTTCACCAACTGTGGAATGTAGAAGATGACTTTTGACGAATTTGTCGAGTCGGAAATTACCCGCGTCGTGTAGTAATACATCTTTCTTTGTGGCATCATCCCAGTCGGGTTTCGCCATGGAGGTGTTCCTATAGGGTACAGCGTACACCGAATTCGA
ACGGGGAACACACGTCACACCGGACGCAGGGTCCACGTACCATTTGTCCATGTAGTCGGTGAGACTGATATCTTCGAGACCGGTGGGTATCATGGGCGCCGTCGACTCCGATTTGATTTTTTTGTTCATAATGTGCACGTTGTTGTTCAAACCAATAGCGGTTAACAATATGGGACAATATTCCGAGG
TGGCCACTCCAGTTACAGTGGCCCCAATATCGAACCCTGAACGTACACCTAGCACACGAACCTGACATGTTACACGCTTAACCCAAGTGTTCGGTGGCACAGTGGCGTATTCCGCAGAGTCCAAATAGAACGGGAGATAGTCGACGGGAATAAAAGATAGAGGTGTAACTGTGCCCAGGAGACTTTGC
TTGGTGTCCAAATACGATGTTACTTGACTGTAACCATACGAATACCAAATACGCGATTTGCTAAACGACGCCGTCCATTGTTGAGAACCGTGGGAATTATTCAAGGGAACAGCACTTGGTCCTCCTCCTCGGCTAGTTCGGGGTTGTTGTCCACTGCCTCCCGTAGGCGGGTCCCCATACTGGGTAGT
ATCCATACGCTCATCCGCAGGCATAGTTTCACTGTTTGTATCGCTGCGAGGTAACTTACTAGCAGAAGGATTATCCAAATTAGGGTCGTTATTTTCCGAATTTGAAACTCCTCGCTTGCGTGTAGCTGATTCAGAAGCTGGGTTTGCTGTGCTTGATTCTGTAAGATTTCCAGGAGCGGATCGTCCAT
ACTCCGTGTCGTGCAAACGGTCACATTCGGCGCTTGTATTTTCACGAACGCGAACAGCGTCTTCGGAAGCACACCAGAGAGCGTACGGCAATGTAGACCCGGAGCGTTTGTATTCGGCAGATAAAGCACGTTGCACAACACTGTACCGGTATTGATAGTATTCCTTCGGACGTTTAACTGTAGGCTTT
GGACCCGGGGGTTTACGCTTCCTCGGTGCCATCGTTGTTCTTCTTACGTTTTATACCGGCCATGGCCGAACGTTGCGCAGTACTAGCACGTAATTTGGAAGGCGCTGGATACACAGTTCCCGTAACGGATTCGAGTAACGATTTCAAACCGAGTCCTGTGTAACCGACAAGCGAACTAAACTCCGTAG
GATGCTCGAGAAACTGATGACGAGCCTCTGCATCTGCTTCTTTTATATGATCCTCTGTCCAGGCATGCGCATAAGCATAATCGTGCTGTTGCGCAACTTTATCGGCGTTCGTTGTAGGTTCTCCGTTGTCGAGAGGATTGCCAGGCCCCATATAATTATGCCCGGGCCACAAAAAACCGCCACGTCGA
```

CGTCGTTGTTGTTGCGTGCTAGCACGATGTAATCCCATTAAAACATATGGTCCTTTATTCGTTTGCCACGTTTTGTATCGCTTATCCGGAACACTATACGGATTCACCAGAGACGATGTTGTAGACATTGTTGTAACACTCCCGAATGCCACTGGTTTTTCTACGCGTTTTACATTTGTTTTTTTCGG
TTTGGCCTTGCCGCTGGAGGGTTTTTTTATCGCTTTTATCTGAGCACTATTATTTACAGATGACATGTTTAAGCACACAAGCCAACTCACTAATGAGTCCTCGAGGCTAACAAATATATTGCGTTATCACACGCCACGACCTTGAGATGACAAATACATGGAGTACTTTTGGAGTGGTTCTTTCACTC
CAAACTCTTTCTAGAATGCAGCACCGACAACAGCAGCATGAGACCTAGATTTTCCTCCCAGGTGCTTACCTAGGCCTTCGGCCGGTGCACGGGAAAATCAAGGAAAATCTAGGTCTCATGCTGCTGTTGTCGGTGCTGCATTCTAGAAAGAGTTTGGAGTGAAAGAACCACTCCAAAAGTACT

5´-hairpin 1 AGTACTTTTGGAGTGGTTCTTTCACTCCAAACTCTTTCTAGAATGCAGCACCGACAACAGCAGCATGAGACCTAGATTTTCCTTGATTTTCCC--GTGC--ACC--GGCCGAAGGCCTAGGTAAGCACCTGGGA-------GGAAAATCTAGGTCTCATGCTGCTGTTGTCGGTGCTGCATTCTAGAAAGAGTTTGGAGTGAAAGAACCACTCCAAAAGTACT 210

3´-hairpin 1 AGTACTTTTGGAGTGGTTCTTTCACTCCAAACTCTTTCTAGAATGCAGCACCGACAACAGCAGCATGAGACCTAGATTTTCC-------TCCCAGGTGCTTACCTAGGCCTTCGGCC--GGT--GCAC--GGGAAAATCAAGGAAAATCTAGGTCTCATGCTGCTGTTGTCGGTGCTGCATTCTAGAAAGAGTTTGGAGTGAAAGAACCACTCCAAAAGTACT 210

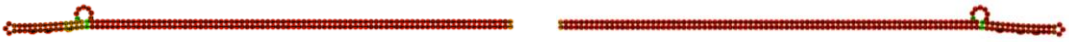

Genome organization and transcription map of DcDNV

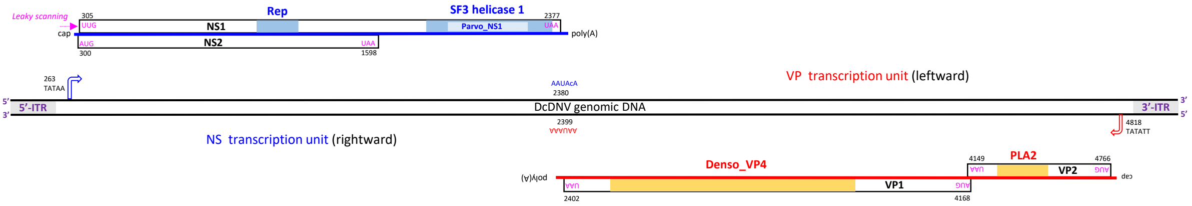

>MN364694_Bactericera_trigonica_densovirus BtDNV

[illegible]

Incomplete ITRs, lacking a flip-flop palindrome

Genome organization and putative transcription map of BtDNV

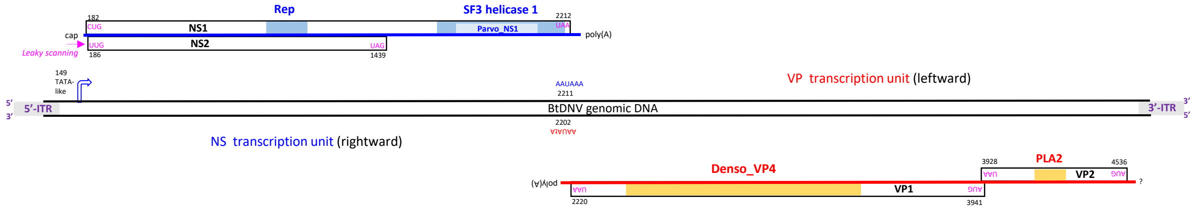

>MN765189.1 MAG: **SAfia-400D ambidensovirus**, complete genome reverse complement

[illegible]

TTTTGTTGGTATCATCGGTTTATCGGCTGCACTCTCGTATGATACATGAGTACCATAAGTACTAACATTTAAATTAGTTGCAACATAACCTATCGGAACGTGCTCCGTATTAGCATTTCCACTTAATGTTTCGCCTACACCAAATGTTGTACGACATCCTAATAATGTAACTTTACAACGTACTTCTA
CCGCTCGACTGACTCTTGGCAAATTTAACCATTCTGCTTCAGTTAGATATGATCCAACATAATCTACTAATGTATTCATAAATGGTGTACAAACACCTTCATCCTCTCCAAATTTTATTGCTTTCATAGCGTATCCATAAGAGTTCCATATTCGGGATTTCTTGAATGTGTATATCTGTGCCTTCGGC
ATAATACTGCGAGGAATCCGTATTGGTGTACGAGAGTATGCTGAATTTCCACCCATAGCATTACCGCCTCTACCTTCGTGAGAAACCATTCCCGATGGAGCGCCACTAATATTGCCAATGTCGATATCCATAGCAGCAAGAAAACTATCATCCAATTCTGGTACGTCGTCTCCCTCTTGAGGGGCACC
GGTTTTGGTTGGGGTTCCGGGTACGGTGGCTCCTCCGGTCTCTCTGTCGGGAGTCCTGGAGGTAGTGTTAATCCGATCACTTGGACCTGGTTGATTATTATCCAATAGATTACTTTTACCACGTTTTGCTGGAAGACGTCTAATAGCTTTCGCTGCGTCACTACGAATGAACTCATGCCATGTACCGG
CATTTGCACCGTGCAATAATTTATGTTGTTTCCAAGCACGAGATAATTCTTTTTGTATATTAGCATATTGTTCTTGGCCAACGTGCGGTCGTTTCGACATGTCAGGATATACTGGTGAACCTTTTAATTTTTCAAATATATATTTTATACCTAATCCAGTTGCTCCTAATAACGATGCAACATCTGCG
CTTTCGAAAAACTCTGCACCAAATTGTAATATTGCTTCTTCATCTGCACGCACTATGTCTTTTTCATTTCTAGCCGACCAATACATTGCATCATGCCTTGCTGCTATCCAATCTGCATCATTAACAGGTTCCCCTTTATCTATATCATTTCCTGGACCTAAATATTTATAACCTGGTAAAGTAAAATC
ACCTTTAAAATACTTTACAAATTCAAAATCGTCCATTGCGCTCGGTAATATAAGAAGAACTGGGCAGCGCCCAGTTTCCCTACCTCGTGAGCGACGATCGCGGGGCGGCGGGGGGTAC

Incomplete 5'-ITR and 3'-ITR, with a putative 33 nt flip-flop palindrome followed by 22 nt sequence, both repeated twice at the 5'-end

Reconstructed 3'-hairpin sequence:

GCTCGGTAATATAAGAAGAACTGGGCAGCGCCCAGTTTCCCTACCTCGTGAGCGACGATCGCGGGGCGGCGGGGGGTACAATCGATCGATCGCGCGAAGCGCATGATCATGATTCGCGGGGCGGCGGGGGGTACAatcgatcgatcgcgcgaagcgcatgatcatgattgtacccccgccgccccgc
gaatcatgatcatgcgcttcgcgcgatcgatcgattgtacccccgccgccccgcgatcgtcgctcacgaggtagggaaactgggcgctgcccagttcttcttatattaccgagc

Predicted secondary structure of the putative 3'-flip-flop palindrome:

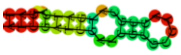

Genome organization and putative transcription map of SAfia-400D ambidensovirus

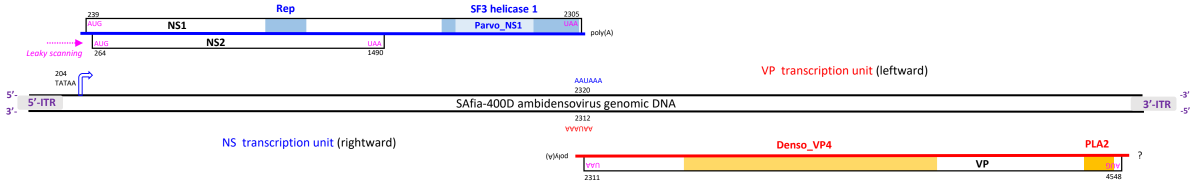


```
>MT138307_Parvoviridae_sp_MAG_rtr168par1
ATTTTACACTGGCGTGACTGGAGGGAGAGGTTTATAAGAGCAGGTACGGCGCAGCGCAGTTGCATTTCTTTTTTGAACAAGATATGGAACGGATGTTGACCTTGTACGAGTGCGCGAAGTGGGTCTTTCTCAGAGACAGAATAGTTTTGCACCAGGAGGACATAGAGGACTTGCCGGTGGATCTACAG
CGTGACCTTTTTTGCACTTGGAGTCGCATCCTTGGAGCTGCCACTGCTACCGCTCTGCGCTCTGCTCCCGCCATTCGCCGGCTTCGCCGGCGTGGGTGTAGGGCGGGAGCAGAGCGCAGAGCGGTAGCAGTGGCGAAGCGTTAGTAATAATTTTACACTGGCGTGACTGGAGGGAGAGGTTTATAAGA
GCAGGTACGGCGCAGCGCAGGTACACTATGAGTTCTGAAACCGACAGTGACTGTTCGTGGACGTATGTGGAAGAAGACGAAGAGGACAAAGACGGTCCTAATCAAGAGCAGTCGTTGGCATTTTACGAGAGGAGACGAAGAGAATTGAGCAAAGATGAGCAGCAAATTTATCAGTTTGTTCAAGAAAT
CCTGGGTGAACATCGACGAGACACCGCAGGCGTGGTTGAAGGCCGTCGAGATCTGCAGCAAGAGAGAAGATGTGTGCGTGGAGGATCAGGAGAAGTTCGCGGAATTGGGCTTTACGATGAGGGAGGTGTTCGACAAGATACTGTTACAGAATCAGAACTTCGAAAAGTGCTCACTACCGAATTTCGTC
GGTCAAATCTACGACACCGAATTGTACATTCGGTATATGAAAGGAAACTTTTTAGAGGAGAGACAATTGAAAATTTTACTCGACACCTACAACGAGCTTTCCATGGATCAATCTTTATCATTGGAGAACACGGAAACCACTACCACGTTGTCCATGACTGCAACTGGTCCTCTTCAACGTGTCGCTGC
AGTAGAATTAGAGGCATCGAATTCGACTCAGAGCACGGTAGATCACGATTTAGATACAATCGGAAAATTACTAGCACCGCAGCTTGGGACATCGACCATTGGATCAATCTGCTCATCTATCTCTCTACAGCAGAACGGCGGCTTAACTATGTTGAAATATCCGGAAAATGCTGGCTACGCGATCATCG
AGGTAAATCTAGCACGCTTTTCCAAGGTGGCTCATTTAGACTCGAACGAAGCCTGGAAAGCGTACGACTATCGGACGAAAATTGTAGTGCAAAACAACTTGGACCACATTCATATCCTGGCGACGAAATTGATCAAGAACATTACGGAACGGCTGAGACATCGGGACGATTTCAAGAAGACAGTGTTT
CGGAAGCGGGCAGCAGCACGTCATTCGGATCCAGATTCGGACGAGGAGCTACAGCAGCGCAAAAAGAAAAGAAATCGCTATTCCAATACCTTCAAGGAGTAATATTCTCACCGCCGAAGGCTCTCTTCGCATCACGGTTGTGGCTCAAGTCTAACTTTAGATATACGGATAGACGTAAGAATTACTTT
CAAGTAACCTTCGAGGAGTTAAAGCTTTTTTACGTAGAACGGTCAATAAGCGATATATGGGCACATGTTTTGGATATAGATAGTACTAAAATATTCTACGCGAGTGAGAATAAAGACGAATATTATTACGGGATGCGCGATTCGGTTAAATATTTAGAAGCGCTATTGTTCTTTCAATTTCACACGGC
CGATCGAGTTCGCACTTTCCTGTTGAATCTGAAATGCATATTGGATAAAGAGCGCCCGAAGGTTAATACTTTATTTATATACGGAGCACCGAATGCCGGTAAGAATTTTTTCTTCGACGCCGTAGCGCATTATTGTGTTAATTTCGGAATCATAAGTAATTGGACGAAGTATAATGCATTTCCGCTTC
AAGATTGCGTAAACAGACGTCTGTTAATGTGGAACGAGCCGAATTTCGAGGACGGCGTAGAGGAAACTTTAAAGATGTTATTCGGAGGTGATCAATGCGGCGCGCGTATTAAATACGAAGGTGACGGAATTATACAACGTACGCCTATTATCGTGCTATCTAACGAAGATCCGTTTCCAACTAATGTA
GCCTTTCGCACGCGCATGATAAAGTATACGTGGCAACCGTGTAAACACTTACGTAAGCTTAAGAAAAAACCGTTTCCTCTGGCGATCGCGTACTTATTAGCTCGTTGGGAAATAATTGATTATAAATCATTATCTTACGAGTTTTCCGACGACGAATTATGTATATTCAACGATGAATCTTTGGACTC
GGCGAGTGAGAGCGAGGTCGACGAGGAAGGCTACGACACTGTATTCTAATCATATGCATTTATTAAATGGTTGTGGGTTATTGCAATACACGATGTGTTTTTGTGTAATCCCGTTGATCATACGTTTCCCGTGTCAATACTCGTTTGTCCGGCATCATCGATATCAGCACTGGGACAATTGGCAATCG
TTCCGGTTTCGACTGTGTCTGTTGTGAGATTCTTGAATTCCCAGGTGCAATTGCTAAGGTACTTAGACGCTGTTGAATCTGTAAGTACGTTTAATACTCATATTGGATACCACAATAGTGCCAATAATCAATTATTAAGCGGACTTACTGGATGCATTATTCACGGAACCTTGTGGCTGTGCGTTGAC
GTGTGCTAACAGGCACATACTGAGAGCCAGGAGCAGACATCGGTCGAATAAACGGATCCGCGTCTCCCTCTGTCGCTTGAAGCAAGTCCAGATAAGAGTTGACATTGGCGTGATGGTTAATGGCCAAACCGGTTTGGTAGGTTTTCGTTCCTTGCGAGCCGATTTGGTTGCTGTAATACTTGTTGGCG
TATGTCGCGATTGGTCCATAATGGAAGCACGAGTTTTGATAATGATGAACGACAGCTTCGACCTGTACACTCCAATATATCGAAGTATTTTGAAAGTCGACGGCATCGTTGGCAGGATTTATAGCCGGTACTGCGGTGAGACCTACGTGAACTTGCGGCTGTACATTGCCGTGCGGTGTACCGCTCGA
CCAATCGTACATCTCGTACGTTTCGATGGTCTGATATCGTATACTCTTAAGATTATTTTGATACTTAACGGATAAGGCGTCTGTTTTATCACTCGCGCCTCCTTGTACAGCCGCGGTACTTTCTTGATTCAATTGCGGAGATAATATAGAACTACTACCTTCACTCGCGGTAATATACGCGGGCCATT
TCCAATCGGTGCCGTTCGGTGTATTAGGTTCTACGTTAGGAGCTAATGTATTATACGTATCGGTGATATAACCTTGTTTCGGTTTATAAGAATATTCTAAGACGGGATTACCAACGCAGGAATTGACTAGCCAACGCTCTACATATTGATCGAATTTAGGAGCACCGTATATATCGTTGAATCTTTTA
TTTACCAAATTACCAGTTTCGGAATTGTCCGACGAGGCTATAATAGGTGTCGCATAAATATTCAAATGACGCGGAATCATTTGTGCCATACCCGTACCATTTCTACCACCGTAAAGTTTATCATCAAGAGTAGTCGTGCTAATTTCCGAGCTAGCTATAGGTTGCATAGGTTTAGTGCTGTCCGGTGT
ATACGAGCGATTTTCCAACGGCATGGCAAGGTTCAATCCAACAGTACTTTGTCCGATAGCGACAAATTCCGAAGTTGCCCATCCTGCTTTGGTCGCGTTTACTTGGAAGTTCATACGACAACCGAGCGGTACGACAGTAGCCCTTACTTCCACCACAACTGCACGCCCGTGTAATAAACTGAATTCCG
CATCGTCCATATAAAACGCAAGACAGTCTACCGGCACAAGACATAAAGGAGTGCAAAAATAGTTATTATTATTAGCGGTGAGAATAGTATGTTTATATCCATAACTAAAAAATACCCACGACTTGCGGTAACTCTTAGTATAATATTCGGTCTTCGGTGTACGTGGAATGCCGATGATTCCTCCTTGC
GCTACAGCCTTCCCGCTCCGTCCACCAGCAGCGGCCCCTGAACGTCCAGATAGGCCGCCTCCGGCAGAAGCACCTTCGCTAGGATTAGCAATCTCCTCAACGCCATCCAGGTCCATTGCAGACATTGCGGGATCAAAAGTATCGTCAGCGAGATCAGAAGCCAGTGGATCGTCGGGTAGTCCAGCGTC
GTCCGCAGTTCGTTTCTGAGCACCGCTATCTCTCGCGCCAGAAGGTCCAGCTTCTCCCCGTTTTTCAGGAGGAGGTCGTTCGACTTGTTGAGGAGCCGATGTACTTCGTAGTCCAGACTCATTGGTCAATTGCCAGTGTGTTTTCCAATTAGATTTAAGATACTGACTCCACGGTAATACTCCTCCGG
TGTTTAACTTATCGACTTTATATTGTGTGCTTAGCGACTGGAGAGTCTTACGATACTGGTCGTGTCCCGCGTGTGTCTTCGGTTTATCTACTTTACCTCTAGCGCCGCGACGAGTAGGAGCCATCCTGCGCGCCGGATACTTCTGTCCGAAGATTCCTTCCCATGATTTCTTAGCTTGTAAACCGACA
GCTCCCACTAGCGAATGTACGTTGAGATTACGCTGAAAATCCTCCGCAAACTTATCGATAGCTTGTGTATCAGCCGCAGCAGCCTCATTCGGATTGCGTTCGTATGCGATGTCGTGCTCTCTCGCTATCTCGTCGTCCCTGTCGACTGGCTCGCCCGCGTCCAACTGATTGCCCGGTCCCAAGTACCG
ATGTCCCGGCCAGTTCCATCCTGCGCGCCGGATACTTCTGTCCGAAGATTCCTTCCCATGATTTCTTAGCTTGTAAACCGACAGCTCCCACTAGCGAATGTACGTTGAGATTACGCTGAAAATCCTCCGCAAACTTATCGATAGCTTGTGTATCAGCCGCAGCAGCCTCATTCGGATTGCGTTCGTAT
GCGATGTCGTGCTCTCTCGCTATCTCGTCGTCCCTGTCGACTGGCTCGCCCGCGTCCAACTGATTGCCCGGTCCCAAGTACCGATGTCCCGGCCAGTTCCAGCCTGCGCGCTTTGACATTCTCCCGCTCTACTGACTGCACGCCTTCCTCGAGCACCTTCCTCTTTCTGCTGGGATCCGCGTAAAAGC
GCTCCATGCAACGTAGCCGAACTCCAAGGATGCGACTCCAAGTGCAAAAAAGGTCACGCTGTAGATCCACCGGCAAGTCCTCTATGTCCTCCTGGTGCAAAACTATTCTGTCTCTGAGAAAGACCCACTTCGCGCACTCGTACAAGGTCAACATCCGTTCCATATCTTGTTCAAAAAAGAAATGCAAC
TGCGCTGCGCCGTACCTGCTCTTATAAACCTCTCCCTCCAGTCACGCCAGTGTAAAATTATTACTAACGCTTCGCCACTGCTACCGCTCTGCGCTCTGCTCCCG
```

Direct repeat underlined => ITRs are incomplete or 5'-ITR was misassembled (no hairpin structure)

Genome organization and putative transcription map of MT138307_Parvo

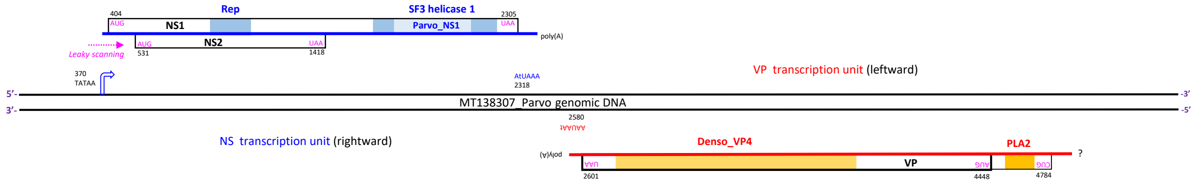

EMBOSS Needle pairwise alignments of complete NS1 and VP proteins of diciambidensoviruses (BtDNV, DcDNV, SAfia-400D ambidensovirus), MT138307_Parvo and BtaDV

NS1/Rep proteins

```
# 1: BtDNV_NS1
# 2: DcDNV_NS1
# Matrix: EBLOSUM62
# Gap_penalty: 10.0
# Extend_penalty: 0.5
# Length: 722
# Identity:     305/722 (42.2%)
# Similarity:   402/722 (55.7%)
# Gaps:          89/722 (12.3%)

BtDNV_NS1          1 -----LFAAKIT------SSDSEFEIIEYDVRHRPILATQLAPG----GP     35
                          ||.:||.      |||.|||        |..::.....|    |.
DcDNV_NS1          1 LFVLLLFTSKIANTSSEYSSDDEFE--------RGFVSISKRRGSVEFGS     42

BtDNV_NS1         36 AESHKRGEHTSHTQL-------FGRRVGHCSGRTAAGEYT--------GG     70
                     :||   |:.::..|.       ..||:    |..:...||        |.
DcDNV_NS1         43 SES---GDESTWAQFKRFRGDELYRRI----GTESPARYTRSRVNVPRGS     85

BtDNV_NS1         71 SGAVS---GRAQHIDGRRQTEESLLEREEGDRDHSLEWQNASGDEGEGES    117
                     ...||   ||    |....:||.:....:.::::     |:..|  .||.
DcDNV_NS1         86 PERVSTFRGR----DSDSGSEEEIYTETDDEKEN-----NSQSD--HGEE    124

BtDNV_NS1        118 DSEADIGAADKSLGSTGQHF----PCRTPSHVERNDPSGSNSLHESYEYG    163
                     |.:.|..|.|.|:|:..|..    ..|..|:.:|:...|.::   :|.|.
DcDNV_NS1        125 DVDTDSSATDISVGTANQQLYGVPVSRLDSYADRSPSPGPST---AYRYC    171

BtDNV_NS1        164 GYEGAQPELQKEDHGLASGAPAVEKKRWRDLYQFTVSCLQNAPSKHVILH    213
                     ..|..:...:|..|.|.......:.|....|....|..||..|.|..|||
DcDNV_NS1        172 SEENPKCGPKKAVHPLGGRLGDRQAKARSQLRNSIVGLLQRVPPKSRILH    221

BtDNV_NS1        214 SVYRLCAAGR----VDRQFHQRVRRLPQFTGN---TIQLISEHGDHIHVL    256
                     .:|.|..:.|    :||......|...  :||   .:|.:..|..|:||:
DcDNV_NS1        222 DIYLLPDSQRNMEHLDRAIRGACRNYT--SGNRQAALQFVCFHKTHLHVV    269

BtDNV_NS1        257 HDCQWTSSTCRCGFIRAFGSDFGTTHWRAKRWVVRRYKFSTAHLTSALLY    306
                     |||.:|||||||.|.|....:.|..:  .||||:|:|:|.:..:.:.|||
DcDNV_NS1        270 HDCTYTSSTCRCAFTRNIQDELGVRY--TKRWVIRQYRFGSERVYNTLLY    317

BtDNV_NS1        307 LSTRGHELGHVQLGRQVFQRGLGSGTQTGHCPIQACFSHPEERLVECGLS    356
                     ....|.:..::|:|.::::.    |.:.|..|:.......:|.|||.|..
DcDNV_NS1        318 FQKTGRQALYIQIGGRIWRH----GCEIGPNPLFGGGDKVQEGLVERGEF    363

BtDNV_NS1        357 EDHSYILGEFGVGECGQNSGGSDRASFPNRASGQAPKRRKVSFEVQLGIE    406
                     .|:..:..|||:|:|.:....|:....|...||...:|      ..|...
DcDNV_NS1        364 ADNGDLSAEFGIGDCDRGLHESNHGPLPESTSGTTVER------YSLPKA    407

BtDNV_NS1        407 LLGWLRQFPSAPLVSILQTRFWTESEFGLQVHRGSKLLCTVLNNYALELC    456
                     :|.||||.|:|||.||||||.|:||:||.:|.|.:.||..:.|||||||.
DcDNV_NS1        408 ILVWLRQHPAAPLTSILQTRHWSESKFGTKVLRTAPLLTNLFNNYALELM    457

BtDNV_NS1        457 DMSMPELFARFQSVDPVHLLFAAPMGNVAATYYDIEESVLILEDLLMYQF    506
                     |.||.|||.|:.|::..:|:||||:||:..|||.::|||:|||.||:||:
DcDNV_NS1        458 DASMVELFERYSSLEHTNLIFAAPLGNIQETYYTLDESVVILEKLLLYQY    507

BtDNV_NS1        507 DNDEEIVGVFLEDLVDILEKRKAKINSIFVLSPSNAGKNFFFDCVVHFFL    556
                     .:|.|||.:||:||.|:||||.||.|:.||||||||||||||||:||||:|
DcDNV_NS1        508 RDDTEIVEIFLQDLYDVLEKRIAKKNTFFVLSPSNAGKNFFFDCIVHFYL    557

BtDNV_NS1        557 NFGLIGNFNKYVGFPLQETVHKRILLWNEPNAEPAAFETLKMLLGGDQCV    606
                     |||||||||||..|||||.|||||||||||.|||:||||||.|||||||.|
DcDNV_NS1        558 NFGLIGNFNKYQNFPLQECVHKRILLWNEPQAEPSAFETLKTLLGGDQTV    607

BtDNV_NS1        607 VRVKFQSDATVGRTPVIILSNNDIFPRDAAFRNRMIKYEWKTAGYLEKYN    656
                     ||||:|.||||||||||||||::|||.||||.|||..|||:||.||.:|.
DcDNV_NS1        608 VRVKYQGDATVGRTPVIILSNHNIFPNDAAFVNRMFVYEWRTAPYLAQYR    657

BtDNV_NS1        657 KKPHLIAFYKLLLKYNIFNT--    676
                     ||||.:|.|.|..||.|...
DcDNV_NS1        658 KKPHPMATYLLFKKYKIIKPEM    679
```

```
# 1: DcDNV_NS1
# 2: BtaDV_NS1
# Matrix: EBLOSUM62
# Gap_penalty: 10.0
# Extend_penalty: 0.5
# Length: 804
# Identity:     215/804 (26.7%)
# Similarity:   336/804 (41.8%)
# Gaps:         213/804 (26.5%)

DcDNV_NS1          1 LFAAKIT---SSDSEFEIIEYD-----------------VRH-----RPI     25
                      .:|.::   ||...|.:|:.|                 |.|     :|:
BtaDV_NS1          1 -MSAHLSDDNSSQDSFSVIDTDSSEFVQVEQLSPSEERYVEHCIATNQPV     49

DcDNV_NS1         26 L-----ATQLAPGGPAES--HKRGEHTSHTQLFGRR---VGHCSGRTAAG     65
                     .     .|:..|...||.  .|:.|:.|:....|..   ||  |.:|...
BtaDV_NS1         50 YLVRDGVTKCIPIVCAEDVLKKKPENPSNISGDGNAFEFVG--SRQTEKS     97

DcDNV_NS1         66 EY--TGGSGAVSGRAQHIDGRRQTEESLLEREEG--DRDHSLEWQNASGD    111
                     .|  .|.|||.||.....||.::...|.|.|...  |.|..|    ..|.
BtaDV_NS1         98 PYLGEGSSGASSGSIPRDDGNKEAGNSGLYRSISPFDSDTGL----LHGT    143

DcDNV_NS1        112 EGEGESDSEADIGAADKSLGS-TGQHFPCRTPSHVERNDPSGSNSLHESY    160
                     .....|.:..|:....::... ..::|     |..|.:....|||..||:
BtaDV_NS1        144 PPRPSSVASTDVRVRRRNRSKRIDEYF-----SGAESSLAGSSNSYQESF    188

DcDNV_NS1        161 EYGGYEGAQPELQKEDHGLASGAPAVEKKRWRDLYQFTVSCLQNAPSKHV    210
                     |...:|                       :::.|:......|.|  .:..
BtaDV_NS1        189 ETVSFE-----------------------KFKSLFCAEFGRLDN--HRRY    213

DcDNV_NS1        211 ILHSVYRLCAAGRVDRQFHQRVRR-LPQFTGNTIQ----LISEHGDHIHV    255
                     |||.:||...:||    |.:..|. :|:.|||:..    ::.:||.|:||
BtaDV_NS1        214 ILHDIYRNTISGR----FRENDRGVVPKDTGNSSSAWTFVVFQHGQHVHV    259

DcDNV_NS1        256 LHDCQWTSSTCRCGFIRAFGSDFGTTHWRAKRWVVRRYKFSTAHLTSALL    305
                     :|.|::..|.|.|...:.....||.   |..|..:|..:.:..||.
BtaDV_NS1        260 IHKCRYERSWCSCALTKKAEERFGR---RIGRHTLRGSRITEGHLE----    302

DcDNV_NS1        306 YLSTRGHELGHVQLGR----QVF----------QRGLGSGTQTGHCPIQA    341
                           |...::|.||    |.|          |.||...||:.
BtaDV_NS1        303 ------HLANYIQKGRRRLLQAFLAGRERRLSRQAGLLQDTQSS------    340

DcDNV_NS1        342 CFSHPEERLVECGLSEDHSYILGEFGVGEC-GQNSGGSDRAS-FPNRASG    389
                       ...:..|||.|...:|   :..|..|.| .|:....:|:| ..:..||
BtaDV_NS1        341 --QSGKGELVEGGRLPEH---ISSFLRGTCRKQHQKNPERSSEEDSENSG    385

DcDNV_NS1        390 Q-----APKRRKVSFEVQLGIELLGWLRQFPSAPLVSILQTRFW--TESE    432
                     :     ||.:|:         :::.:|:.....|...|.|::.:  .:|.
BtaDV_NS1        386 EEGGVPAPTKRE---------KIIQFLQSCVITPFSRIFQSQLYLFDKSL    426

DcDNV_NS1        433 FGLQVHRGSKLLCTVLNNYALELCDMSMPELFARFQSVDPVHLLFAAPMG    482
                     :.:.....|..|..:.:.:.:.  .|::.::| ::|...|...|| ....
BtaDV_NS1        427 YFITPESMSCQLAILQHTHTIN--KMTVQQIF-QYQLNIPFSKLF-YNCD    472

DcDNV_NS1        483 NVAATYYDIEESVLILEDLLMYQ-----FDNDEEIVGVFLEDLVDILEKR    527
                     |....:.::..|:|.:|.||.:|     |.|       |||:|::||:||
BtaDV_NS1        473 NPEDYFLNLSASILCIETLLKHQVGSFGFVN-------FLENLLNILDKR    515

DcDNV_NS1        528 KAKINSIFVLSPSNAGKNFFFDCVVHFFLNFGLIGNFNKYVGFPLQETVH    577
                     ..|:|::.:..|:|:|||||||.|....:|.|.:||||::..|||||...
BtaDV_NS1        516 IPKVNTLLIAGPANSGKNFFFDAVTQAMINCGHVGNFNRFCNFPLQECNM    565

DcDNV_NS1        578 KRILLWNEPNAEPAAFETLKMLLGGDQCVVRVKFQSDATVGRTPVIILSN    627
                     |||||||||..||:|.||:|||..||.|..::|:::|||:.|||||:|:|
BtaDV_NS1        566 KRILLWNEPVCEPSAVETIKMLFAGDPCPAKIKYKNDATIMRTPVIVLTN    615

DcDNV_NS1        628 NDIFPRDAAFRNRMIKYEWKTAGYLEKYNKKPHLIAFYKLLLKYNIFNT-    676
                     |:.||.|..|.:|||||.|:.:.:|.:.:||||.:|.:.:|.|||:.:|
BtaDV_NS1        616 NNPFPNDPPFNSRMIKYTWRQSPFLAEIHKKPHPLALFYVLAKYNLIDTL    665

DcDNV_NS1        677 --------------------------------------------------    676

BtaDV_NS1        666 ELDNFDSSLLQDLSVTTFIQEATHDIDSSIRSTEAIHHDEDNISRIMDIF    715

DcDNV_NS1        677 ----    676

BtaDV_NS1        716 MKDI    719

# 1: BtaDV_NS1
# 2: BtDNV_NS1
# Matrix: EBLOSUM62
# Gap_penalty: 10.0
# Extend_penalty: 0.5
# Length: 804
# Identity:     215/804 (26.7%)
# Similarity:   336/804 (41.8%)
# Gaps:         213/804 (26.5%)

BtaDV_NS1          1 -MSAHLSDDNSSQDSFSVIDTDSSEFVQVEQLSPSEERYVEHCIATNQPV     49
                      .:|.::   ||...|.:|:.|                 |.|     :|:
BtDNV_NS1          1 LFAAKIT---SSDSEFEIIEYD-----------------VRH-----RPI     25

BtaDV_NS1         50 YLVRDGVTKCIPIVCAEDVLKKKPENPSNISGDGNAFEFVG--SRQTEKS     97
                     .     .|:..|...||.  .|:.|:.|:....|..   ||  |.:|...
BtDNV_NS1         26 L-----ATQLAPGGPAES--HKRGEHTSHTQLFGRR---VGHCSGRTAAG     65

BtaDV_NS1         98 PYLGEGSSGASSGSIPRDDGNKEAGNSGLYRSISPFDSDTGL----LHGT    143
                     .|  .|.|||.||.....||.::...|.|.|...  |.|..|    ..|.
BtDNV_NS1         66 EY--TGGSGAVSGRAQHIDGRRQTEESLLEREEG--DRDHSLEWQNASGD    111

BtaDV_NS1        144 PPRPSSVASTDVRVRRRNRSKRIDEYF-----SGAESSLAGSSNSYQESF    188
                     .....|.:..|:....::... ..::|     |..|.:....|||..||:
BtDNV_NS1        112 EGEGESDSEADIGAADKSLGS-TGQHFPCRTPSHVERNDPSGSNSLHESY    160

BtaDV_NS1        189 ETVSFE-----------------------KFKSLFCAEFGRLDN--HRRY    213
                     |...:|                       :::.|:......|.|  .:..
BtDNV_NS1        161 EYGGYEGAQPELQKEDHGLASGAPAVEKKRWRDLYQFTVSCLQNAPSKHV    210

BtaDV_NS1        214 ILHDIYRNTISGR----FRENDRGVVPKDTGNSSSAWTFVVFQHGQHVHV    259
                     |||.:||...:||    |.:..|. :|:.|||:..    ::.:||.|:||
BtDNV_NS1        211 ILHSVYRLCAAGRVDRQFHQRVRR-LPQFTGNTIQ----LISEHGDHIHV    255

BtaDV_NS1        260 IHKCRYERSWCSCALTKKAEERFGR---RIGRHTLRGSRITEGHLE----    302
                     :|.|::..|.|.|...:.....||.   |..|..:|..:.:..||.
BtDNV_NS1        256 LHDCQWTSSTCRCGFIRAFGSDFGTTHWRAKRWVVRRYKFSTAHLTSALL    305

BtaDV_NS1        303 ------HLANYIQKGRRRLLQAFLAGRERRLSRQAGLLQDTQSS------    340
                           |...::|.||    |.|          |.||...||:.
BtDNV_NS1        306 YLSTRGHELGHVQLGR----QVF----------QRGLGSGTQTGHCPIQA    341

BtaDV_NS1        341 --QSGKGELVEGGRLPEH---ISSFLRGTCRKQHQKNPERSSEEDSENSG    385
                       ...:..|||.|...:|   :..|..|.| .|:....:|:| ..:..||
BtDNV_NS1        342 CFSHPEERLVECGLSEDHSYILGEFGVGEC-GQNSGGSDRAS-FPNRASG    389

BtaDV_NS1        386 EEGGVPAPTKRE---------KIIQFLQSCVITPFSRIFQSQLYLFDKSL    426
                     :     ||.:|:         :::.:|:.....|...|.|::.:  .:|.
BtDNV_NS1        390 Q-----APKRRKVSFEVQLGIELLGWLRQFPSAPLVSILQTRFW--TESE    432

BtaDV_NS1        427 YFITPESMSCQLAILQHTHTIN--KMTVQQIF-QYQLNIPFSKLF-YNCD    472
                     :.:.....|..|..:.:.:.:.  .|::.::| ::|...|...|| ....
BtDNV_NS1        433 FGLQVHRGSKLLCTVLNNYALELCDMSMPELFARFQSVDPVHLLFAAPMG    482

BtaDV_NS1        473 NPEDYFLNLSASILCIETLLKHQVGSFGFVN-------FLENLLNILDKR    515
                     |....:.::..|:|.:|.||.:|     |.|       |||:|::||:||
BtDNV_NS1        483 NVAATYYDIEESVLILEDLLMYQ-----FDNDEEIVGVFLEDLVDILEKR    527

BtaDV_NS1        516 IPKVNTLLIAGPANSGKNFFFDAVTQAMINCGHVGNFNRFCNFPLQECNM    565
                     ..|:|::.:..|:|:|||||||.|....:|.|.:||||::..|||||...
BtDNV_NS1        528 KAKINSIFVLSPSNAGKNFFFDCVVHFFLNFGLIGNFNKYVGFPLQETVH    577

BtaDV_NS1        566 KRILLWNEPVCEPSAVETIKMLFAGDPCPAKIKYKNDATIMRTPVIVLTN    615
```

```
                    |||||||||..||:|.||:|||..||.|..::|:::|||:.|||||:|:|
BtDNV_NS1        578 KRILLWNEPNAEPAAFETLKMLLGGDQCVVRVKFQSDATVGRTPVIILSN    627

BtaDV_NS1        616 NNPFPNDPPFNSRMIKYTWRQSPFLAEIHKKPHPLALFYVLAKYNLIDTL    665
                     |:.||.|..|.:|||||.|:.:.:|.:.:||||.:|.:.:|.|||:.:|
BtDNV_NS1        628 NDIFPRDAAFRNRMIKYEWKTAGYLEKYNKKPHLIAFYKLLLKYNIFNT-    676

BtaDV_NS1        666 ELDNFDSSLLQDLSVTTFIQEATHDIDSSIRSTEAIHHDEDNISRIMDIF    715

BtDNV_NS1        677 --------------------------------------------------    676

BtaDV_NS1        716 MKDI    719

BtDNV_NS1        677 ----    676

# 1: SAfia-400D_NS1
# 2: BtaDV_NS1
# Matrix: EBLOSUM62
# Gap_penalty: 10.0
# Extend_penalty: 0.5
# Length: 819
# Identity:     221/819 (27.0%)
# Similarity:   335/819 (40.9%)
# Gaps:         231/819 (28.2%)

SAfia-400D_NS      1 MSDDSSDKHEDSDQFSSGIDFERTEREEFISSVSDYINASEFKYRRIRSR     50
                     ||...||.:...|.||. ||   |:..||:
BtaDV_NS1          1 MSAHLSDDNSSQDSFSV-ID---TDSSEFV--------------------     26

SAfia-400D_NS     51 TRERFKRVSPNEKQFTTTIECNDQ------FGGTRTMHGFGS--------     86
                        :.:::||:|::........:|      .|.|:.:....:
BtaDV_NS1         27 ---QVEQLSPSEERYVEHCIATNQPVYLVRDGVTKCIPIVCAEDVLKKKP     73

SAfia-400D_NS     87 EYPSTSRGNGS-----GSRKISTSKNYKKKKKTIEQHEEISSSDEESSQS    131
                     |.||...|:|:     |||                |.|:.....|.||.:
BtaDV_NS1         74 ENPSNISGDGNAFEFVGSR----------------QTEKSPYLGEGSSGA    107

SAfia-400D_NS    132 LSGS-PSRQFFRYFGANVVYEYVS---SD---------------NEEIKI    162
                     .||| |.....:..|.:.:|..:|   ||               :.::::
BtaDV_NS1        108 SSGSIPRDDGNKEAGNSGLYRSISPFDSDTGLLHGTPPRPSSVASTDVRV    157

SAfia-400D_NS    163 RRTD---------GNAKEGFASEEGVQQFDMFMEDNFENNNPQTEREKGR    203
                     ||.:         ..|:...|......| :.|...:||........|.||
BtaDV_NS1        158 RRRNRSKRIDEYFSGAESSLAGSSNSYQ-ESFETVSFEKFKSLFCAEFGR    206

SAfia-400D_NS    204 RFILECFQNYPPRFRIIHDVF--TIQNERYSDTNISRICESSGNSGRSFK    251
                        |:..:.|     |:||::  ||.. |:.:.:...:.:.:|||..::.
BtaDV_NS1        207 ---LDNHRRY-----ILHDIYRNTISG-RFRENDRGVVPKDTGNSSSAWT    247

SAfia-400D_NS    252 GAIFWVCNHANDHLHVIHDCSYNGNTCRCQRIKEIRGECGQPRLNRRLLR    301
                     ..:|   .| ..|:||||.|.|..:.|.|...|:.....|: |:.|..||
BtaDV_NS1        248 FVVF---QH-GQHVHVIHKCRYERSWCSCALTKKAEERFGR-RIGRHTLR    292

SAfia-400D_NS    302 SADISSWHWINLAIYFESDERKAIHFEIAGRMWLQCGKTRNLSIRKNL--    349
                     .:.|:..|..:||.|.:...|:.:...:|||       .|.||.:..|
BtaDV_NS1        293 GSRITEGHLEHLANYIQKGRRRLLQAFLAGR-------ERRLSRQAGLLQ    335

SAfia-400D_NS    350 -----QFGKEVLVETCGNEDDIPHEFQ------CRSHSTDSEQNPESSNA    388
                          |.||..|||  |..  :|....      ||.   ..::|||.|:.
BtaDV_NS1        336 DTQSSQSGKGELVE--GGR--LPEHISSFLRGTCRK---QHQKNPERSSE    378

SAfia-400D_NS    389 TNNQNRRSS----KRNKGDKLLEFFWRFPTAPINNI-------FNNSVWF    427
                     .:::|....    ...|.:|:::|.......|.:.|       |:.|::|
BtaDV_NS1        379 EDSENSGEEGGVPAPTKREKIIQFLQSCVITPFSRIFQSQLYLFDKSLYF    428

SAfia-400D_NS    428 TSP----WKYLPLNNTHI---RAVQRIYQRQLCDMNFMDFVNRYKYSEVL    470
                     .:|    .:...|.:||.   ..||:|:|.||          ...:|: |
BtaDV_NS1        429 ITPESMSCQLAILQHTHTINKMTVQQIFQYQL----------NIPFSK-L    467

SAfia-400D_NS    471 YFNCGVRDVNDYYYTTDQSLRIATDLL-----LFQFNNNLDRVQQFLKDL    515
                     ::||.  :..||:.....|:.....||     .|.|.|       ||::|
BtaDV_NS1        468 FYNCD--NPEDYFLNLSASILCIETLLKHQVGSFGFVN-------FLENL    508

SAfia-400D_NS    516 FNLLDRITPKKNCMFILSPPNAGKNYFFDAVIHFFLNFGQIGNFNRYNNF    565
                     .|:||:..||.|.:.|..|.|:|||:|||||....:|.|.:|||||:.||
BtaDV_NS1        509 LNILDKRIPKVNTLLIAGPANSGKNFFFDAVTQAMINCGHVGNFNRFCNF    558

SAfia-400D_NS    566 PLMECVNRRIILWNEPHCETSAFETIKMLFGGDTLNVKVKFQDDAIVNRT    615
                     ||.||..:||:|||||.||.||.|||||||.||....|:|:::||.:.||
BtaDV_NS1        559 PLQECNMKRILLWNEPVCEPSAVETIKMLFAGDPCPAKIKYKNDATIMRT    608

SAfia-400D_NS    616 PCIVLSNNDIFPKDTAFRTRMYNYVWRSCDALKRCKKKLYPLCIPKLFIQ    665
                     |.|||:||:.||.|..|.:||..|.||....|....||.:||.:..:..:
BtaDV_NS1        609 PVIVLTNNNPFPNDPPFNSRMIKYTWRQSPFLAEIHKKPHPLALFYVLAK    658

SAfia-400D_NS    666 FNIINDIGYIIEENNTSSNSDSE---------------------------    688
                     :|:|:.:        ...|.||.
BtaDV_NS1        659 YNLIDTL--------ELDNFDSSLLQDLSVTTFIQEATHDIDSSIRSTEA    700

SAfia-400D_NS    689 -------------------    688

BtaDV_NS1        701 IHHDEDNISRIMDIFMKDI    719

# 1: MT138307_Parvo_NS1
# 2: BtaDV_NS1
# Matrix: EBLOSUM62
# Gap_penalty: 10.0
# Extend_penalty: 0.5
# Length: 791
# Identity:     198/791 (25.0%)
# Similarity:   304/791 (38.4%)
# Gaps:         230/791 (29.1%)

MT138307_Parv      1 --------------------------------------------------      0

BtaDV_NS1          1 MSAHLSDDNSSQDSFSVIDTDSSEFVQVEQLSPSEERYVEHCIATNQPVY     50

MT138307_Parv      1 ------------------------------------------------MS      2
                                                                     ..
BtaDV_NS1         51 LVRDGVTKCIPIVCAEDVLKKKPENPSNISGDGNAFEFVGSRQTEKSPYL    100

MT138307_Parv      3 SETDSDCSWTYVEEDEEDKDGPNQ--EQSLAFYER---------------     35
                     .|..|..|...:..|:.:|:..|.  .:|::.::.
BtaDV_NS1        101 GEGSSGASSGSIPRDDGNKEAGNSGLYRSISPFDSDTGLLHGTPPRPSSV    150

MT138307_Parv     36 -------RRRELSKDEQQIYQFVQEILGEHRRDTAGVVEGRRDLQQERRC     78
                            |||..||...:.:...:..|.
BtaDV_NS1        151 ASTDVRVRRRNRSKRIDEYFSGAESSLA----------------------    178

MT138307_Parv     79 VRGGSGEVRGIGLYDEGGVRQDTVTESELRKVLTTEF-RRSNLRHRIVHS    127
                       |.|..      |.|.   .:||:..:.:.:...|| |..|.|..|:|.
BtaDV_NS1        179 --GSSNS------YQES---FETVSFEKFKSLFCAEFGRLDNHRRYILHD    217

MT138307_Parv    128 VYERKLFRGETIEN----FTRHLQRAFHGSIFIIGEHGNHYHVVHDCNWS    173
                     :| |....|...||    ..:....:.....|::.:||.|.||:|.|.:.
BtaDV_NS1        218 IY-RNTISGRFRENDRGVVPKDTGNSSSAWTFVVFQHGQHVHVIHKCRYE    266

MT138307_Parv    174 SSTCRCSRIRGIE--FDSEHGRSRFRYNRKITSTAAWDIDHWINLLIYLS    221
                     .|.|.|:..:..|  |....||...|.:| ||.       .|..:|..|:.
BtaDV_NS1        267 RSWCSCALTKKAEERFGRRIGRHTLRGSR-ITE------GHLEHLANYIQ    309

MT138307_Parv    222 TAERRLNYVEISGKCWLRDHRGKSSTLFQGGSFRLERSLESVRLSDENCS    271
                     ...|||....::|    |:.|...    |.|..:..:|.:|.:  .|...
BtaDV_NS1        310 KGRRRLLQAFLAG----RERRLSR----QAGLLQDTQSSQSGK--GELVE    349

MT138307_Parv    272 AKQLGPH--SYPGDEIDQEHYGTAETSGRFQEDSVSEAGSSTSFGSRFGR    319
                     ..:|..|  |:......::|....|.|.....:..|.|
BtaDV_NS1        350 GGRLPEHISSFLRGTCRKQHQKNPERSSEEDSENSGEEG-----------    388

MT138307_Parv    320 GATAAQKEKKSLFQYLQGVIFSPPKALFASRLWLKSNFRYTDRRKNYFQV    369
                     |..|..|.:| :.|:||..:.:|...:|.|:|:|..        |:.:.:
BtaDV_NS1        389 GVPAPTKREK-IIQFLQSCVITPFSRIFQSQLYLFD--------KSLYFI    429
```

```
MT138307_Parv    370 TFEEL--KLFYVER-------SISDIWAHVLDIDSTKIFYASENKDEYYY    410
                     |.|.:  :|..::.       ::..|:.:.|:|..:|:||..:|.::|:.
BtaDV_NS1        430 TPESMSCQLAILQHTHTINKMTVQQIFQYQLNIPFSKLFYNCDNPEDYFL    479

MT138307_Parv    411 GMRDSVKYLEALLFFQFHTADRVRTFLLNLKCILDKERPKVNTLFIYGAP    460
                     .:..|:..:|.||..|..:...| .||.||..||||..||||||.|.|..
BtaDV_NS1        480 NLSASILCIETLLKHQVGSFGFV-NFLENLLNILDKRIPKVNTLLIAGPA    528

MT138307_Parv    461 NAGKNFFFDAVAHYCVNFGIISNWTKYNAFPLQDCVNRRLLMWNEPNFED    510
                     |:|||||||||....:|.|.:.|:.::..||||:|..:|:|:||||..|.
BtaDV_NS1        529 NSGKNFFFDAVTQAMINCGHVGNFNRFCNFPLQECNMKRILLWNEPVCEP    578

MT138307_Parv    511 GVEETLKMLFGGDQCGARIKYEGDGIIQRTPIIVLSNEDPFPTNVAFRTR    560
                     ...||:||||.||.|.|:|||:.|..|.|||:|||:|.:|||.:..|.:|
BtaDV_NS1        579 SAVETIKMLFAGDPCPAKIKYKNDATIMRTPVIVLTNNNPFPNDPPFNSR    628

MT138307_Parv    561 MIKYTWQPCKHLRKLKKKPFPLAIAYLLARWEIIDYKSL----SYEFSDD    606
                     ||||||:....|.::.|||.|||:.|:||::.:||...|    |....|.
BtaDV_NS1        629 MIKYTWRQSPFLAEIHKKPHPLALFYVLAKYNLIDTLELDNFDSSLLQDL    678

MT138307_Parv    607 ELCIFNDES---LDSASES----EVDEEGYDTVF-------    633
                     .:..|..|:   :||:..|    ..||:....:.
BtaDV_NS1        679 SVTTFIQEATHDIDSSIRSTEAIHHDEDNISRIMDIFMKDI    719
-------------------------------------------------------------------------------
```

VP proteins

```
# 1: BtDNV_VP2
# 2: BcDNV_VP2
# Matrix: EBLOSUM62
# Gap_penalty: 10.0
# Extend_penalty: 0.5
# Length: 603
# Identity:     264/603 (43.8%)
# Similarity:   355/603 (58.9%)
# Gaps:          45/603 ( 7.5%)

BtDNV_VP2          1 MAPTSRRLKKPRKNLVRRTLAHNRNVYATVQRAQSAEWNKRGRPVPWKEW     50
                     |||..|:...| |..|:|...:.:..|:.||||.|||:.:.|..:|:..|
BcDNV_VP2          1 MAPRKRKPPGP-KPTVKRPKEYYQYRYSVVQRALSAEYKRSGSTLPYALW     49

BtDNV_VP2         51 VRSDLARQIRENTLAECNRLYDEQYGASTSGSAGRFDATGDEHSEQSKRA    100
                     ..|:.|.::||||.|||:||:|.:||.|..|:... .:|.:..||.:.|.
BcDNV_VP2         50 CASEDAVRVRENTSAECDRLHDTEYGRSAPGNLTE-SSTANPASESATRK     98

BtDNV_VP2        101 RLDGDDTIDSDTLPSDAADSLQSGTTMSEPMDEDAILQDT------SGSG    144
                     |...:...:...|.:.:|..|....|.||.|..|..:..|      :|..
BcDNV_VP2         99 RGVSNSENNDPNLDNPSASKLPRSDTNSETMPADERMDTTQYGDPPTGGS    148

BtDNV_VP2        145 GRGPTSGRTTGGATVPLGLSSGDATFQATYSKSRIWYSYAFANKKLDLDS    194
                     |:.|.:.|..|.:.|||..|.|...:.|::|||||||||.::.....||:
BcDNV_VP2        149 GQQPRTSRGGGPSAVPLNNSHGSQQWTASFSKSRIWYSYGYSQVTSYLDT    198

BtDNV_VP2        195 TKTLEGIVTPLAFIPVDFLPFYMSPAEFASIPDRNVWIDKVTCKVRVLGV    244
                     .::|.|.||||:|||||:||||:..||:|::|. |.|:.:|||:||||||
BcDNV_VP2        199 KQSLLGTVTPLSFIPVDYLPFYLDSAEYATVPP-NTWVKRVTCQVRVLGV    247

BtDNV_VP2        245 RSGFDIGATLTGTATSEYCPILHTCVGLNNEIHIMNKSVEANTTQPMIPT    294
                     |||||||||:||.||||||||||.|.:||||.:|||||.:::.:|.||||
BcDNV_VP2        248 RSGFDIGATVTGVATSEYCPILLTAIGLNNNVHIMNKKIKSESTAPMIPT    297

BtDNV_VP2        295 GVRDIDLAEYKKKWYTGETSNVIGTPRSNSVYAIPYWNSSTT-----SAT    339
                     |:.||.|.:|..|||....|.|...||||||||:||.|:|..     .||
BcDNV_VP2        298 GLEDISLTDYMDKWYVDPASGVTCVPRSNSVYAVPYRNTSMAKPDWDDAT    347

BtDNV_VP2        340 --EVQKHTWGNPRLDRFVKTHMLTSTIGEEIFDYSYSPRFAPIRPQDQLV    387
                       :|..|..||.|||:|||:|:|.||:||.:.||.|.|....|:..|.|.
BcDNV_VP2        348 KKDVLLHDAGNFRLDKFVKSHLLHSTVGETVVDYEYEPHMGVIKEPDPLC    397

BtDNV_VP2        388 IPNHRVNEASTFQSRDLWQAKCAYGSGKFTMKDYSWPEMVPL-----CDT    432
                     ||.||.::.....:.::..|.|     ..:.|:|...:..||     ..:
BcDNV_VP2        398 IPYHRASDGLMMNAMNIVDATC-----DVSTKEYKLTQKEPLVKHHMAKS    442

BtDNV_VP2        433 AFVYAQLIEKYDMYMPGTGSVGGEQRAQPQLHIGMSAIPQLNPTTDLTSF    482
                     .|.|...||||.::.|..|::...|..|||:|||:||||||||.|:..:|
BcDNV_VP2        443 KFFYHSQIEKYGIFAPRYGALNPRQNVQPQVHIGLSAIPQLNPATEHKNF    492

BtDNV_VP2        483 VNACVYYSVECSIHLTGKREAYWTEGGPTCREENTIFSRHKAFYYENPHT    532
                     ||.||||:|:|||.|..:|...|| |.||..|.:.:||.|..:|||.||:
BcDNV_VP2        493 VNCCVYYAVDCSIELESRRTTEWT-GKPTQPETDALFSSHANYYYEQPHS    541

BtDNV_VP2        533 AFGMWDGDGGTIKM--KSKREFESPRME----------ELHRRKEITTKI    570
                     .|||||||||:||:  ..|....|.:|           ||.|..|:.
BcDNV_VP2        542 VFGMWDGDGGSIKVTTNKKSSSTSARLEKKLAERTKEAELIREFEVL---    588

BtDNV_VP2        571 QHL    573

BcDNV_VP2        589 ---    588

# 1: BtaDV_VP
# 2: BcDNV_VP2
# Matrix: EBLOSUM62
# Gap_penalty: 10.0
# Extend_penalty: 0.5
# Length: 796
# Identity:     178/796 (22.4%)
# Similarity:   286/796 (35.9%)
# Gaps:         255/796 (32.0%)

BtaDV_VP           1 MPLWPKHNYLGPGNDLINGDPEDEDDEVALQHDISYEFAKSEEEIFESDK     50

BcDNV_VP2          1 --------------------------------------------------      0

BtaDV_VP          51 RAISDFANIAVNDLKNWDINTGAIAGTGLLFLKHNLESAIGTTLYPWHPD    100

BcDNV_VP2          1 --------------------------------------------------      0

BtaDV_VP         101 GGDVSDMNKTTYALREKTKSLVYQNLKRLGKVHTSYR----------EFW    140
                                .|.|::........:|| .|.:..||          |:.
BcDNV_VP2          1 -----------MAPRKRKPPGPKPTVKR-PKEYYQYRYSVVQRALSAEYK     38

BtaDV_VP         141 RSDSGANLAQTIYSDFANHGEHWQSLIADGT-LDIDRVSNGLVSQYVSTY    189
                     ||.|....|....|:.|..       :.:.| .:.||:.:       :.|
BcDNV_VP2         39 RSGSTLPYALWCASEDAVR-------VRENTSAECDRLHD-------TEY     74

BtaDV_VP         190 SRNSTPSIATSSDTPPQSPVPGPSTSGAPRQEAGDFGNSEGGKRKAEESS    239
                     .| |.|...|.|.|      ..|::..|.|:..  ..|||.....
BcDNV_VP2         75 GR-SAPGNLTESST------ANPASESATRKRG--VSNSENNDPN-----    110

BtaDV_VP         240 ILDNSSNKRVATSSSGSSSSSLSASSTN-QSMEVDGVTD-------PKGK    281
                      |||            .|:|.|..|.|| ::|..|...|       |.|.
BcDNV_VP2        111 -LDN------------PSASKLPRSDTNSETMPADERMDTTQYGDPPTGG    147

BtaDV_VP         282 RGTGAGISKVTPPVSLNKSSTHSGNK--IVITKSRMLFCHAIAPAQLSVN    329
                     .|.....|:...|.::..:::|...:  ...:|||:.:.:..:    .|.
BcDNV_VP2        148 SGQQPRTSRGGGPSAVPLNNSHGSQQWTASFSKSRIWYSYGYS----QVT    193

BtaDV_VP         330 AELNIGRNVSAKVLGLAYIPVDYLPFYLTEQEYNAIPDNSRVLRVGCKIT    379
                     :.|:..:::...|..|::||||||||||...||..:|.|:.|.||.|::.
BcDNV_VP2        194 SYLDTKQSLLGTVTPLSFIPVDYLPFYLDSAEYATVPPNTWVKRVTCQVR    243

BtaDV_VP         380 PVGCRTAFDTGTSTSGVATNEWIAVGRSVVGLNLKIPIYNVHLSTKADEP    429
                     .:|.|:.||.|.:.:||||:|:..:..:.:|||..:.|.|..:.:::..|
BcDNV_VP2        244 VLGVRSGFDIGATVTGVATSEYCPILLTAIGLNNNVHIMNKKIKSESTAP    293

BtaDV_VP         430 MIPTSISRLDHTTMQNSWYLNNDICNVLGYPTQINEYAAYIFNTNSEK--    477
                     ||||.:..:..|...:.||: :....|...|...:.||....||:..|
BcDNV_VP2        294 MIPTGLEDISLTDYMDKWYV-DPASGVTCVPRSNSVYAVPYRNTSMAKPD    342

BtaDV_VP         478 -NTTYKKDVDYSYHCGGNPRLDKVIERFMFNAHIGSPIIDYEYSPQNAMI    526
                      :...||||  ..|..||.||||.::..:.::.:|..::||||.|...:|
```

```
BcDNV_VP2        343 WDDATKKDV--LLHDAGNFRLDKFVKSHLLHSTVGETVVDYEYEPHMGVI    390

BtaDV_VP         527 KKHKIVNLPYPTCSQ-VILNGENPGPWNINIDGWASDVD-----------    564
                     |:...:.:||...|. :::|.       :||.....||.
BcDNV_VP2        391 KEPDPLCIPYHRASDGLMMNA-------MNIVDATCDVSTKEYKLTQKEP    433

BtaDV_VP         565 ---HDKNKGRAYYKSVNSATSDMYVPFECKYNRMLESGPYSHNFKAAGGE    611
                        |...|.:.:|.|    ..:.|..|..:|..:   .|         .:
BcDNV_VP2        434 LVKHHMAKSKFFYHS----QIEKYGIFAPRYGAL---NP---------RQ    467

BtaDV_VP         612 NIQPQVHIGLMPTPNINPANSSVSFQNSAAYFEVECYIEISSNVDSAFTG    661
                     |:||||||||...|.:|||....:|.|...|:.|:|.||:.|...:.:||
BcDNV_VP2        468 NVQPQVHIGLSAIPQLNPATEHKNFVNCCVYYAVDCSIELESRRTTEWTG    517

BtaDV_VP         662 -----GTDAVMWNDDITIYY---HDTVPLHDGLSVGKKRVKPSSASVYAG    703
                          .||| :::.....||   |....:.|| ..|..:|
BcDNV_VP2        518 KPTQPETDA-LFSSHANYYYEQPHSVFGMWDG-DGGSIKV----------    555

BtaDV_VP         704 ETSKHSKMPRSRLQAEASEKQSAVENTQYYKDIMKIKALDVENKKK    749
                     .|:|.|....:||:.:.:|:....|..:.::.:
BcDNV_VP2        556 TTNKKSSSTSARLEKKLAERTKEAELIREFEVL-------------    588

# 1: BtaDV_VP
# 2: BtDNV_VP2
# Matrix: EBLOSUM62
# Gap_penalty: 10.0
# Extend_penalty: 0.5
# Length: 778
# Identity:     168/778 (21.6%)
# Similarity:   282/778 (36.2%)
# Gaps:         234/778 (30.1%)

BtaDV_VP           1 MPLWPKHNYLGPGNDLINGDPEDEDDEVALQHDISYEFAKSEEEIFESDK     50

BtDNV_VP2          1 --------------------------------------------------      0

BtaDV_VP          51 RAISDFANIAVNDLKNWDINTGAIAGTGLLFLKHNLESAIGTTLYPWHPD    100

BtDNV_VP2          1 --------------------------------------------------      0

BtaDV_VP         101 GGDVSDMNKTTYALREKTKSLVYQNLKRLGKVHTSYR-----EFWRSDSG    145
                           |..|:..|::..|:||.:.|.....|:.:.:     |:.:....
BtDNV_VP2          1 ------MAPTSRRLKKPRKNLVRRTLAHNRNVYATVQRAQSAEWNKRGRP     44

BtaDV_VP         146 ANLAQTIYSDFANHGEHWQSLIADGTL-DIDRVSNGLVSQYVSTYSRNST    194
                     ....:.:.||.|..       |.:.|| :.:|:.:   .||
BtDNV_VP2         45 VPWKEWVRSDLARQ-------IRENTLAECNRLYD---EQY---------     75

BtaDV_VP         195 PSIATSSDTPPQSPVPGPSTSG-APRQEAGDFGNSEGGKRKAEESSILDN    243
                                     |.|||| |.|.:|....:||..||     :.||.
BtDNV_VP2         76 ----------------GASTSGSAGRFDATGDEHSEQSKR-----ARLDG    104

BtaDV_VP         244 SSNKRVATSSSGSSSSSLSASSTNQSMEVDGV---TDPKGKRGTGAGISK    290
                     .......|..|.::.|..|.::.::.|:.|.:   |...|.||..:|.:.
BtDNV_VP2        105 DDTIDSDTLPSDAADSLQSGTTMSEPMDEDAILQDTSGSGGRGPTSGRTT    154

BtaDV_VP         291 VTPPVSLNKSSTHSGNKIVITKSRMLFCHAIAPAQLSVNAELNIGRNVSA    340
                     ....|.|..||..:.:...:|||:.:.:|.|..:|    :|:..:.:..
BtDNV_VP2        155 GGATVPLGLSSGDATFQATYSKSRIWYSYAFANKKL----DLDSTKTLEG    200

BtaDV_VP         341 KVLGLAYIPVDYLPFYLTEQEYNAIPD-NSRVLRVGCKITPVGCRTAFDT    389
                     .|..||:||||:||||::..|:.:||| |..:.:|.||:..:|.|:.||.
BtDNV_VP2        201 IVTPLAFIPVDFLPFYMSPAEFASIPDRNVWIDKVTCKVRVLGVRSGFDI    250

BtaDV_VP         390 GTSTSGVATNEWIAVGRSVVGLNLKIPIYNVHLSTKADEPMIPTSISRLD    439
                     |.:.:|.||:|:..:..:.||||.:|.|.|..:.....:|||||.:..:|
BtDNV_VP2        251 GATLTGTATSEYCPILHTCVGLNNEIHIMNKSVEANTTQPMIPTGVRDID    300

BtaDV_VP         440 HTTMQNSWYLNNDICNVLGYPTQINEYAAYIFNTNSEKNTTYKKDVDYSY    489
                     ....:..|| ..:..||:|.|...:.||...:|:::...|..:|
BtDNV_VP2        301 LAEYKKKWY-TGETSNVIGTPRSNSVYAIPYWNSSTTSATEVQK------    343

BtaDV_VP         490 HCGGNPRLDKVIERFMFNAHIGSPIIDYEYSPQNAMIKKHKIVNLPYPTC    539
                     |..||||||:.::..|..:.||..|.||.|||:.|.|:....:.:|
BtDNV_VP2        344 HTWGNPRLDRFVKTHMLTSTIGEEIFDYSYSPRFAPIRPQDQLVIP----    389

BtaDV_VP         540 SQVILNGENPGPWNINIDGWASDVDHDKNKGRAYYKSVNSATSDM----Y    585
                                  |..::..::....|..:.:..|.|......|.    .
BtDNV_VP2        390 -------------NHRVNEASTFQSRDLWQAKCAYGSGKFTMKDYSWPEM    426

BtaDV_VP         586 VPFECK----YNRMLES-GPYSHNFKAAGGE-NIQPQVHIGLMPTPNINP    629
                     ||. |.    |.:::|. ..|.....:.||| ..|||:|||:...|.:||
BtDNV_VP2        427 VPL-CDTAFVYAQLIEKYDMYMPGTGSVGGEQRAQPQLHIGMSAIPQLNP    475

BtaDV_VP         630 ANSSVSFQNSAAYFEVECYIEISSNVDSAFTGG-----TDAVMWNDDITI    674
                     .....||.|:..|:.|||.|.::...::.:|.|     .:..:::.....
BtDNV_VP2        476 TTDLTSFVNACVYYSVECSIHLTGKREAYWTEGGPTCREENTIFSRHKAF    525

BtaDV_VP         675 YY---HDTVPLHDGLSVGKKRVKPSSASVYAGETSKHSKMPRSRLQAEAS    721
                     ||   |....:.|| ..|..::|          :.:..:.||........
BtDNV_VP2        526 YYENPHTAFGMWDG-DGGTIKMK----------SKREFESPRMEELHRRK    564

BtaDV_VP         722 EKQSAVENTQYYKDIMKIKALDVENKKK    749
                     |..:.:::.
BtDNV_VP2        565 EITTKIQHL-------------------    573

# 1: SAfia-400D_VP
# 2: BtaDV_VP
# Matrix: EBLOSUM62
# Gap_penalty: 10.0
# Extend_penalty: 0.5
# Length: 862
# Identity:     195/862 (22.6%)
# Similarity:   318/862 (36.9%)
# Gaps:         230/862 (26.7%)

SAfia-400D_VP      1 MDDFEFVKYFKGDFTL-PGYKYLGPGNDIDKGEPVNDADWIAARHDAMYW     49
                                  ..| |.:.|||||||:..|:|.::.|.:|.:||..|.
BtaDV_VP           1 -------------MPLWPKHNYLGPGNDLINGDPEDEDDEVALQHDISYE     37

SAfia-400D_VP     50 SARNEKDIVRADEEAILQF---GAEFFESADV-ASLLGATG-LGIKYIFE     94
                     .|::|::|..:|:.||..|   .....::.|: ...:..|| |.:|:..|
BtaDV_VP          38 FAKSEEEIFESDKRAISDFANIAVNDLKNWDINTGAIAGTGLLFLKHNLE     87

SAfia-400D_VP     95 KLKGSPVYP---------DMSKRPHVGQEQYAN-IQKELSRAWKQHKLLH    134
                     ...|:.:||         ||:|..:..:|:..: :.:.|.|..|.|.
BtaDV_VP          88 SAIGTTLYPWHPDGGDVSDMNKTTYALREKTKSLVYQNLKRLGKVHT---    134

SAfia-400D_VP    135 GANAGTWHEFIRSDA----AKAIRRLPAKRGK-------SNLLDNNQPG-    172
                          ::.||.|||:    |:.|....|..|:       ...||.::..
BtaDV_VP         135 -----SYREFWRSDSGANLAQTIYSDFANHGEHWQSLIADGTLDIDRVSN    179

SAfia-400D_VP    173 --PSDRINTTSR--TPDRETGGAT-----VPGTPTKTGAP-QEGDDVPE-    211
                       .|..::|.||  ||...|...|     ||| |:.:||| ||..|...
BtaDV_VP         180 GLVSQYVSTYSRNSTPSIATSSDTPPQSPVPG-PSTSGAPRQEAGDFGNS    228

SAfia-400D_VP    212 ------------LDD------------SFLAAMDIDIGNISGAPSGMVSH    237
                                 ||:            |..:::.....|.|....|:...
BtaDV_VP         229 EGGKRKAEESSILDNSSNKRVATSSSGSSSSSLSASSTNQSMEVDGVTDP    278

SAfia-400D_VP    238 EGRGGNAMGGNSAYSRTPIRIPRSIMPKAQIYTFKKSRIWNSYGYAMKAI    287
                     :|:.|...|.:..  ..|:.:.:|...........|||:...:..|...:
BtaDV_VP         279 KGKRGTGAGISKV--TPPVSLNKSSTHSGNKIVITKSRMLFCHAIAPAQL    326

SAfia-400D_VP    288 KFGED----EGVCTPFMNTL---VDYVGSYLTEAEWLNLPRVSRAVEVRC    330
                     ....:    ..|....:...   |||:..||||.|:..:|..||.:.|.|
BtaDV_VP         327 SVNAELNIGRNVSAKVLGLAYIPVDYLPFYLTEQEYNAIPDNSRVLRVGC    376

SAfia-400D_VP    331 KVTLLGCRTTFGVGETLSGNANTEHVPIG--YVATNLNVSTYGTHVSYES    378
                     |:|.:||||.|..|.:.||.|..|.:.:|  .|..||.:..|..|:|  :
BtaDV_VP         377 KITPVGCRTAFDTGTSTSGVATNEWIAVGRSVVGLNLKIPIYNVHLS--T    424
```

```
SAfia-400D_VP    379 AADKPMIPTKCVLPSIESTV--KKMYL--DVPCQSLGVP----------    413
                     .||:|||||.  :..::.|.  ...||  |: |..||.|
BtaDV_VP         425 KADEPMIPTS--ISRLDHTTMQNSWYLNNDI-CNVLGYPTQINEYAAYIF    471

SAfia-400D_VP    414 ----RKNSIYWWYRDDTHAVTGVGNMMLDCICDRFLINSMIGQTVAEYKY    459
                         .||:.|  .:|..::....||..||.:.:||:.|:.||..:.:|:|
BtaDV_VP         472 NTNSEKNTTY--KKDVDYSYHCGGNPRLDKVIERFMFNAHIGSPIIDYEY    519

SAfia-400D_VP    460 NPKNGILTPERIHVTPKAYHNKGMYMNTAAFISKRVLNKSGMTPDKLSTL    509
                     :|:|.::...:|...|  |..          .|:.:||.....|..::..
BtaDV_VP         520 SPQNAMIKKHKIVNLP--YPT----------CSQVILNGENPGPWNINID    557

SAfia-400D_VP    510 G---------DKGDMFGKGLGDQLGVTYGSTIEYSAVFNPRDPVYNATMI    550
                     |         :||..:.|.:....          |.::.|.:..||..:.
BtaDV_VP         558 GWASDVDHDKNKGRAYYKSVNSAT----------SDMYVPFECKYNRMLE    597

SAfia-400D_VP    551 P---------------QPQLHVGIGAIPQINPATEFENYQTTMVYWLCEY    585
                     .               |||:|:|:...|.||||....::|.:..|:..|.
BtaDV_VP         598 SGPYSHNFKAAGGENIQPQVHIGLMPTPNINPANSSVSFQNSAAYFEVEC    647

SAfia-400D_VP    586 EMDVSVDLHSYYTYGTISNM--PQKVKYYYVHSPYYTTGETFAGIEQNLE    633
                     .:::|.::.|.:|.||.:.|  .....||:...|          :...|.
BtaDV_VP         648 YIEISSNVDSAFTGGTDAVMWNDDITIYYHDTVP----------LHDGLS    687

SAfia-400D_VP    634 RSVKLINTENESTEPEEEPDYCPITGKDLQQSVYTNRHNIYKKPFNYDSP    683
                     ...|.:...:.|....|...:..:....||......:..:....:..|..
BtaDV_VP         688 VGKKRVKPSSASVYAGETSKHSKMPRSRLQAEASEKQSAVENTQYYKDIM    737

SAfia-400D_VP    684 AFRTRSKEKSKNIKSRGTSKRSNAGGGCGVVNANEITSNEEDFEILSDSS    733
                     ..:....|..|.
BtaDV_VP         738 KIKALDVENKKK--------------------------------------    749

SAfia-400D_VP    734 SISNINKRFIKM    745

BtaDV_VP         750 ------------    749

# 1: BtaDV_VP
# 2: MT138307_Parvo_VP2
# Matrix: EBLOSUM62
# Gap_penalty: 10.0
# Extend_penalty: 0.5
# Identity:     164/830 (19.8%)
# Similarity:   268/830 (32.3%)
# Gaps:         296/830 (35.7%)

BtaDV_VP           1 MPLWPKHNYLGPGNDLINGDPEDEDDEVALQHDISYEFAKSEEEIFESDK     50

MT138307_Parv      1 --------------------------------------------------      0

BtaDV_VP          51 RAISDFANIAVNDLKNWDINTGAIAGTGLLFLKHNLESAIGTTLYPWHPD    100

MT138307_Parv      1 --------------------------------------------------      0

BtaDV_VP         101 GGDVSDMNKTTYALREKTKSLVYQNLKRLGKVHTSYREFWRSDSGANLAQ    150
                                                                    :|.
MT138307_Parv      1 -----------------------------------------------MAP      3

BtaDV_VP         151 TIY---------SDFANHGEHWQSLIADGT-LDIDRVSNGLV---SQYV-    186
                     |..         ...|.|.::.::|.:..| ..:|:::.|.|   |||:
MT138307_Parv      4 TRRGARGKVDKPKTHAGHDQYRKTLQSLSTQYKVDKLNTGGVLPWSQYLK     53

BtaDV_VP         187 ---STYSRNSTPSIATSSDTPPQSPVPGPSTSGAPRQEAGDFGNSEGG--    231
                        .|:.:.:..|...|:..|.|...|.|...|    |||..|..:.|
MT138307_Parv     54 SNWKTHWQLTNESGLRSTSAPQQVERPPPEKRG----EAGPSGARDSGAQ     99

BtaDV_VP         232 KRKAEESSILDNSSNKRVATSSSGSSSSSLSASSTNQSMEVDGV---TDP    278
                     ||.|:::.:.|:.....:|..:...:.|         :|::|||   .:|
MT138307_Parv    100 KRTADDAGLPDDPLASDLADDTFDPAMS---------AMDLDGVEEIANP    140

BtaDV_VP         279 KGKRGTGAGISKVTPPVSLNKS--STHSGNKIVI-------------TKS    313
                     ......|.|:|..:...:..:|  :...|..|.|             .||
MT138307_Parv    141 SEGASAGGGLSGRSGAAAGGRSGKAVAQGGIIGIPRTPKTEYYTKSYRKS    190

BtaDV_VP         314 RMLFCHAIAPAQLSVNAELNIGRNVSAKVLGLAYIPVDYLPFYLTEQEYN    363
                     .:.|.:......|:.|       |.:.....|..:|||.|.||:.:.|::
MT138307_Parv    191 WVFFSYGYKHTILTAN-------NNNYFCTPLCLVPVDCLAFYMDDAEFS    233

BtaDV_VP         364 AIPDNSRVLRVGCKITPVGCRTAFDTGTSTSGVATNEWIAVGRSVVGLNL    413
                     .:...:.|:.|...:.|:|||..|....:.:|.||:|::|:|:|.|||||
MT138307_Parv    234 LLHGRAVVVEVRATVVPLGCRMNFQVNATKAGWATSEFVAIGQSTVGLNL    283

BtaDV_VP         414 KIPIYNVHLSTKADEPMIPTSISRLDHTTMQNSWYLNNDICNVLG----Y    459
                     .:|:.|...:.:.:||.|.:.|.:..||:.:..|...   |..|    .
MT138307_Parv    284 AMPLENRSYTPDSTKPMQPIASSEISTTTLDDKLYGGR---NGTGMAQMI    330

BtaDV_VP         460 PTQINEYAAYIF--NTNSEKNTTYKKDVDYSYHCGGNPRLDKVIERFMFN    507
                     |..:|.||..|.  :.|||......|..:..|   |.|:.|:.:||::.|
MT138307_Parv    331 PRHLNIYATPIIASSDNSETGNLVNKRFNDIY---GAPKFDQYVERWLVN    377

BtaDV_VP         508 AHIGSPIIDYEYSPQNAMIKKHKIVNLPYPTCSQVILNGENPGPWNINI-    556
                     :.:|:|:::|.|.|:...|.  ...|...|.......||.: ..|...|
MT138307_Parv    378 SCVGNPVLEYSYKPKQGYIT--DTYNTLAPNVEPNTPNGTD-WKWPAYIT    424

BtaDV_VP         557 --DGWASDVDHDKNKGRAYYKSVNSATSDMYVPFECKYNRMLESGPYS--    602
                       :|.:|.:....|:...  .:|....||.......||...|:|..|.
MT138307_Parv    425 ASEGSSSILSPQLNQEST--AAVQGGASDKTDALSVKYQNNLKSIRYQTI    472

BtaDV_VP         603 ---HNFKAAGGE---NIQPQVHIGLMPTPNINPANSSVSFQNSAAYFEVE    646
                        ..:..:.|.   |:|||||:||...|.|||||.:|.|||::.|:.|:
MT138307_Parv    473 ETYEMYDWSSGTPHGNVQPQVHVGLTAVPAINPANDAVDFQNTSIYWSVQ    522

BtaDV_VP         647 CYIEISSNVDSAFTGGTDAVMWNDDITIYYHD------TVPLHDGLSVGK    690
                     ....:....:|.|..|..|...|.    ||.:      |.....||::..
MT138307_Parv    523 VEAVVHHYQNSCFHYGPIATYANK----YYSNQIGSQGTKTYQTGLAINH    568

BtaDV_VP         691 KR-------------------VKPSSA--SVYAGETSKHSKMPRSRLQAE    719
                     ..                   ::|.||  |.|...:::.....|.|.
MT138307_Parv    569 HANVNSYLDLLQATEGDADPFIRPMSAPGSQYVPVSTRQRTATRFRE---    615

BtaDV_VP         720 ASEKQSAVENTQYYKDIMKIKALDVENKKK    749

MT138307_Parv    616 ------------------------------    615

# 1: BtDNV_VP2
# 2: MT138307_Parvo_VP2
# Matrix: EBLOSUM62
# Gap_penalty: 10.0
# Extend_penalty: 0.5
# Length: 693
# Identity:     171/693 (24.7%)
# Similarity:   258/693 (37.2%)
# Gaps:         198/693 (28.6%)

BtDNV_VP2          1 MAPTSR----RLKKPRKNLVRRTLAHNRNVYATVQRAQSAEWN----KRG     42
                     ||||.|    ::.||:.:     ..|::  |....::.|.::.    ..|
MT138307_Parv      1 MAPTRRGARGKVDKPKTH-----AGHDQ--YRKTLQSLSTQYKVDKLNTG     43

BtDNV_VP2         43 RPVPWKEWVRSDLARQIRENTLAECNRLYDEQYGASTS------------     80
                     ..:||.::::|:.....         :|.:|....|||
MT138307_Parv     44 GVLPWSQYLKSNWKTHW---------QLTNESGLRSTSAPQQVERPPPEK     84

BtDNV_VP2         81 -GSAGRFDATGDEHSEQSKRARLDG---DDTIDSDTLPSDAADSLQSGTT    126
                      |.||   .:|...|...||...|.   ||.:.|| |..|..|...|.
MT138307_Parv     85 RGEAG---PSGARDSGAQKRTADDAGLPDDPLASD-LADDTFDPAMSA--    128

BtDNV_VP2        127 MSEPMDEDA---ILQDTSGSGGRGPTSGRTTGGATVPLGLSSGDATFQA-    172
                         ||.|.   |...:.|:...|..|||:...|    |..||.|..|.
MT138307_Parv    129 ----MDLDGVEEIANPSEGASAGGGLSGRSGAAA----GGRSGKAVAQGG    170

BtDNV_VP2        173 ---------------TYSKSRIWYSYAFANKKLDLDSTKTLEGIVTPLAF    207
                                    :|.||.:::||.:.:..|..::...   ..|||..
MT138307_Parv    171 IIGIPRTPKTEYYTKSYRKSWVFFSYGYKHTILTANNNNY---FCTPLCL    217
```

```
BtDNV_VP2        208 IPVDFLPFYMSPAEFASIPDRNVWIDKVTCKVRVLGVRSGFDIGATLTGT    257
                     :|||.|.|||..|||:.:..|.|.:: |...|..||.|..|.:.||..|.
MT138307_Parv    218 VPVDCLAFYMDDAEFSLLHGRAVVVE-VRATVVPLGCRMNFQVNATKAGW    266

BtDNV_VP2        258 ATSEYCPILHTCVGLNNEIHIMNKSVEANTTQPMIPTGVRDIDLAEYKKK    307
                     ||||:..|..:.||||..:.:.|:|...::|:||.|....:|.......|
MT138307_Parv    267 ATSEFVAIGQSTVGLNLAMPLENRSYTPDSTKPMQPIASSEISTTTLDDK    316

BtDNV_VP2        308 WYTGETSNVIGT------PRSNSVYAIPYWNSSTTSATE--VQKH---TW    346
                     .|.|..    ||      ||..::||.|...||..|.|.  |.|.   .:
MT138307_Parv    317 LYGGRN----GTGMAQMIPRHLNIYATPIIASSDNSETGNLVNKRFNDIY    362

BtDNV_VP2        347 GNPRLDRFVKTHMLTSTIGEEIFDYSYSPRFAPIRPQDQLVIPNHRVNEA    396
                     |.|:.|::|:..::.|.:|..:.:|||.|:...|......:.||...|..
MT138307_Parv    363 GAPKFDQYVERWLVNSCVGNPVLEYSYKPKQGYITDTYNTLAPNVEPNTP    412

BtDNV_VP2        397 STFQSRDLWQAKCAYGSGKFTMKDYSWPEMVPLCD--------------T    432
                     :              |:      |:.||..:...:              |
MT138307_Parv    413 N--------------GT------DWKWPAYITASEGSSSILSPQLNQEST    442

BtDNV_VP2        433 AFVYA----------------------QLIEKYDMYMPGTGSVGGEQRAQ    460
                     |.|..                      |.||.|:||...:|:..|  ..|
MT138307_Parv    443 AAVQGGASDKTDALSVKYQNNLKSIRYQTIETYEMYDWSSGTPHG--NVQ    490

BtDNV_VP2        461 PQLHIGMSAIPQLNPTTDLTSFVNACVYYSVECSI---HLTGKREAYWTE    507
                     ||:|:|::|:|.:||..|...|.|..:|:||:...   |.......|   
MT138307_Parv    491 PQVHVGLTAVPAINPANDAVDFQNTSIYWSVQVEAVVHHYQNSCFHY---    537

BtDNV_VP2        508 GGPTCREENTIFSRH------KAF-------YYENPHTAFGM---WDGD-    540
                      ||.....|..:|..      |.:       ::.|.::...:   .:|| 
MT138307_Parv    538 -GPIATYANKYYSNQIGSQGTKTYQTGLAINHHANVNSYLDLLQATEGDA    586

BtDNV_VP2        541 ----------GGTIKMKSKREFESPRMEELHRRKEITTKIQHL    573
                               |......|.|:..:.|..|
MT138307_Parv    587 DPFIRPMSAPGSQYVPVSTRQRTATRFRE--------------    615

# 1: BcDNV_VP2
# 2: MT138307_Parvo_VP2
# Matrix: EBLOSUM62
# Gap_penalty: 10.0
# Extend_penalty: 0.5
# Length: 690
# Identity:     175/690 (25.4%)
# Similarity:   280/690 (40.6%)
# Gaps:         177/690 (25.7%)

BcDNV_VP2          1 MAPRKRKPPGPKPTVKRPKEYY---QYRYSVVQRALSAEYK----RSGST     43
                     |||.:|   |.:..|.:||.:.   |||.::  ::||.:||    .:|..
MT138307_Parv      1 MAPTRR---GARGKVDKPKTHAGHDQYRKTL--QSLSTQYKVDKLNTGGV     45

BcDNV_VP2         44 LPYALWCAS----------EDAVRVRENTSA--ECDRLHDTEYGRSAPGN     81
                     ||::.:..|          |..:|   :|||  :.:|....:.|.:.|  
MT138307_Parv     46 LPWSQYLKSNWKTHWQLTNESGLR---STSAPQQVERPPPEKRGEAGP--     90

BcDNV_VP2         82 LTESSTANPASESATRKRGVSNSENNDPNL-DNPSASKLPRSDTNSETMP    130
                                 |..|..|......:|..| |:|.||.| ..||....|.
MT138307_Parv     91 ------------SGARDSGAQKRTADDAGLPDDPLASDL-ADDTFDPAMS    127

BcDNV_VP2        131 ADERMDTTQYGDPPTGGS--------------GQQPRTSRGGGPSAVPLN    166
                     |.:.....:..:|..|.|              |:..:....||...:|  
MT138307_Parv    128 AMDLDGVEEIANPSEGASAGGGLSGRSGAAAGGRSGKAVAQGGIIGIP--    175

BcDNV_VP2        167 NSHGSQQWTASFSKSRIWYSYGYSQVTSYLDTKQSLLGT------VTPLS    210
                     .:..::.:|.|:.||.:::||||         |.::|..      .|||.
MT138307_Parv    176 RTPKTEYYTKSYRKSWVFFSYGY---------KHTILTANNNNYFCTPLC    216

BcDNV_VP2        211 FIPVDYLPFYLDSAEYATVPPNTWVKRVTCQVRVLGVRSGFDIGATVTGV    260
                     .:|||.|.||:|.||::.:.....|..|...|..||.|..|.:.||..|.
MT138307_Parv    217 LVPVDCLAFYMDDAEFSLLHGRAVVVEVRATVVPLGCRMNFQVNATKAGW    266

BcDNV_VP2        261 ATSEYCPILLTAIGLNNNVHIMNKKIKSESTAPMIPTGLEDISLTDYMDK    310
                     ||||:..|..:.:|||..:.:.|:....:||.||.|....:||.|...||
MT138307_Parv    267 ATSEFVAIGQSTVGLNLAMPLENRSYTPDSTKPMQPIASSEISTTTLDDK    316

BcDNV_VP2        311 WYVD-PASGVT-CVPRSNSVYAVPYRNTSMAKPDWDDATKKDVLLHDAGN    358
                     .|.. ..:|:. .:||..::||.|...:|.....:...|:...::.|..
MT138307_Parv    317 LYGGRNGTGMAQMIPRHLNIYATPIIASSDNSETGNLVNKRFNDIYGAPK    366

BcDNV_VP2        359 FRLDKFVKSHLLHSTVGETVVDYEYEPHMGVIK----------EPD----    394
                     |  |::|:..|::|.||..|::|.|:|..|.|.          ||:    
MT138307_Parv    367 F--DQYVERWLVNSCVGNPVLEYSYKPKQGYITDTYNTLAPNVEPNTPNG    414

BcDNV_VP2        395 -----PLCIPYHRASDGLMMNAMNIVDATCDV----STKEYKLTQKEPLV    435
                          |..|.....|..::...:| .::|..|    |.|...|:.|    
MT138307_Parv    415 TDWKWPAYITASEGSSSILSPQLN-QESTAAVQGGASDKTDALSVK----    459

BcDNV_VP2        436 KHHMAKSKFFYHSQIEKYGIFAPRYGALNPRQNVQPQVHIGLSAIPQLNP    485
                       :....|...:..||.|.::  .:.:..|..|||||||:||:|:|.:||
MT138307_Parv    460 --YQNNLKSIRYQTIETYEMY--DWSSGTPHGNVQPQVHVGLTAVPAINP    505

BcDNV_VP2        486 ATEHKNFVNCCVYYAV-----------DC--------------SIELESR    510
                     |.:..:|.|..:|::|           .|              |.::.|:
MT138307_Parv    506 ANDAVDFQNTSIYWSVQVEAVVHHYQNSCFHYGPIATYANKYYSNQIGSQ    555

BcDNV_VP2        511 RTTEWTGKPTQPETDALFSSHANY--YYEQPHSVFGMWDGD--------G    550
                     .|..:       :|....:.|||.  |.:...:..|  |.|        .
MT138307_Parv    556 GTKTY-------QTGLAINHHANVNSYLDLLQATEG--DADPFIRPMSAP    596

BcDNV_VP2        551 GS--IKVTTNKKSSSTSARLEKKLAERTKEAELIREFEVL    588
                     ||  :.|:|.::   |:.|..:
MT138307_Parv    597 GSQYVPVSTRQR---TATRFRE------------------    615
```

(E) Analysis of BtaDV-related endogenous viral elements (EVEs) integrated into the genomes of different *B. tabaci* biotypes

BlastN match for the BtaDV NS1 protein (Rep endonuclease motifs)

unnamed protein product [Bemisia tabaci]: 47% coverage, 67.16% identity
Sequence ID: CAH0383699.1 **Length: 486**

```
Identities      Positives      Gaps
227/338(67%)    289/338(85%)   0/338(0%)

Query  1    MSAHLSDDNSSQDSFSVIDTDSSEFVQVEQLSPSEERYVEHCIATNQPVYLVRDGVTKCI  60
            MS HLSDD+SSQ+SFS+IDTDSS+ VQVEQLSPSEERYVEHCI TNQPVYLVRDGVTKCI
Sbjct  1    MSVHLSDDSSSQNSFSIIDTDSSDLVQVEQLSPSEERYVEHCIQTNQPVYLVRDGVTKCI  60

Query  61   PIVCAEDVLKKKPENPSNISGDGNAFEFVGSRQTEKSPYLGEGSSGASSGSIPRDDGNKE  120
            PIVCAEDV +K  E+ S ++G+G+AF+FV S+Q E SP+LGEG+SG SS S+   DG+ E
Sbjct  61   PIVCAEDVFQKGKESTSGVAGNGDAFQFVSSQQNEDSPFLGEGTSGTSSWSVHGTDGSAE  120

Query  121  AGNSGLYRSISPFDSDTGLLHGTPPRPSSVASTDVRVRRRNRSKRIDEYFSGAESSLAGS  180
             GN+  +RS++P  S +GL  G+  R SS AS++V +R  NR +RID+YFSG+E S+AG
Sbjct  121  EGNTRFHRSVTPTVSHSGLHDGSSSRASSPASSNVGIRGNNRKRRIDQYFSGSEGSVAGG  180

Query  181  SNSYQESFETVSFEKFKSLFCAEFGRLDNHRRYILHDIYRNTISGRFRENDRGVVPKDTG  240
            ++ Y+ESFE +SF+KFK LFC+EF R+D++RRY+LHDIYRNT+SGRFRE+DRG++PK TG
Sbjct  181  TDCYEESFEAISFQKFKDLFCSEFSRMDHNRRYMLHDIYRNTLSGRFREDDRGIIPKSTG  240

Query  241  NSSSAWTFVVFQHGQHVHVIHKCRYERSWCSCALTKKAEERFGRRIGRHTLRGSRITEGH  300
            NSS++W FVVF+HG+H+HVIH CRYERSWCSCALTKKAE+RFGRR+GR++LR SR+++ H
Sbjct  241  NSSASWAFVVFEHGKHIHVIHLCRYERSWCSCALTKKAEKRFGRRVGRYSLRSSRVSKSH  300

Query  301  LEHLANYIQKGRRRLLQAFLAGRERRLSRQAGLLQDTQ  338
            L +LA+YIQKG+RRLLQAFLA RERR+  +AGLLQ+ +
Sbjct  301  LSNLADYIQKGKRRLLQAFLARRERRILGEAGLLQNKE  338
```

InterPro analysis of domains of the unnamed protein of *B. tabaci*

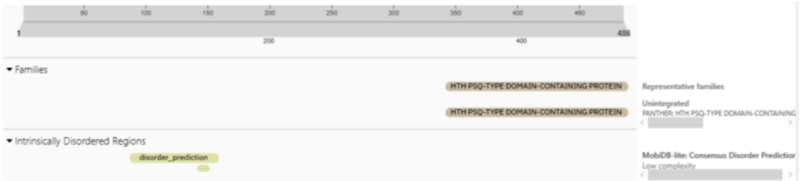

The *B. tabaci* Q-type Almeria genome locus encoding the protein homologous to BtaDV NS1

```
gene            37633155..37635896
                     /locus_tag="BEMITA_LOCUS3122"
     mRNA            join(37633155..37634178,37635460..37635896)
                     /locus_tag="BEMITA_LOCUS3122"
                     /note="ID:BEMITA_01T014299-RA;
                     source:maker"
     CDS             join(37633155..37634178,37635460..37635896)
                     /locus_tag="BEMITA_LOCUS3122"
                     /note="unnamed protein product; source:maker;
                     ID:cds.BEMITA_01T014299-RA"
                     /codon_start=1
                     /protein_id="CAH0383699.1"
                     /translation="MSVHLSDDSSSQNSFSIIDTDSSDLVQVEQLSPSEERYVEHCIQ
                     TNQPVYLVRDGVTKCIPIVCAEDVFQKGKESTSGVAGNGDAFQFVSSQQNEDSPFLGE
                     GTSGTSSWSVHGTDGSAEEGNTRFHRSVTPTVSHSGLHDGSSSRASSPASSNVGIRGN
                     NRKRRIDQYFSGSEGSVAGGTDCYEESFEAISFQKFKDLFCSEFSRMDHNRRYMLHDI
                     YRNTLSGRFREDDRGIIPKSTGNSSASWAFVVFEHGKHIHVIHLCRYERSWCSCALTK
                     KAEKRFGRRVGRYSLRSSRVSKSHLSNLADYIQKGKRRLLQAFLARRERRILGEAGLL
                     QNKEPKKKNILLAMLNDALQDVKQLAVQKIMNARRTKSSTVRKFKVPLLNFNAETYSE
                     MIFWNEVTVTQPPILQYFSEEDLENLAREKDLQNYSFYKYPCHTQSVERCVKIVTEAS
                     ALVCGGEERDGLIRSKLKARLLMPAFEPKSQFKFVK"
```

BlastN analysis of the complete BtaDV genome against the annotated genome assembly of *B. tabaci* Q-type Almeria (unknown sex multi-individual) population originating from Spain (BioProject: PRJEB47898)

Descriptions | Graphic Summary | Alignments | Taxonomy

Sequences producing significant alignments Download Select columns Show 100

select all *5 sequences selected* GenBank Graphics Distance tree of results MSA Viewer

Description	Scientific Name	Max Score	Total Score	Query Cover	E value	Per. Ident	Acc. Len	Accession
Bemisia tabaci chromosome 10, PGI_BMITA_v3	Bemisia tabaci	1044	1369	35%	0.0	75.06%	42753141	NC_092802.1
Bemisia tabaci chromosome 6, PGI_BMITA_v3	Bemisia tabaci	182	292	10%	7e-43	71.54%	54143761	NC_092798.1
Bemisia tabaci chromosome 4, PGI_BMITA_v3	Bemisia tabaci	79.7	126	5%	6e-12	69.26%	62539693	NC_092796.1
Bemisia tabaci chromosome 2, PGI_BMITA_v3	Bemisia tabaci	54.5	54.5	2%	2e-04	71.03%	79695644	NC_092794.1
Bemisia tabaci chromosome 8, PGI_BMITA_v3	Bemisia tabaci	53.6	53.6	2%	2e-04	71.15%	47528266	NC_092800.1

Distribution of the top 11 Blast Hits on 5 subject sequences

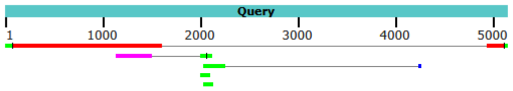

BtaDV-related EVEs in the chromosome 10 of the *B. tabaci* Q-type Almeria genome

Bemisia tabaci chromosome 10, PGI_BMITA_v3

Sequence ID: NC_092802.1 Length: 42753141 **Number of Matches: 4**
Range 1: 37632757 to 37634315

```
Identities      Gaps          Strand
1180/1572(75%)  28/1572(1%)   Plus/Plus
Features:
12978 bp at 5' side: protein deadpan isoform x1
6754 bp at 3' side: brahma-associated protein of 60 kda

Query  41        ATAAAATCGATCGTAGATCATGATTTTAGGGTCGTTACGTCGATAAGGATCATATAAAAC  100        TATA-box
                 ||||||||||||| |||||||||||||||||||||||||||||||||||| | |||||| 
Sbjct  37632757  ATAAAATCGATCGAAGATCATGATTTTAGGGTCGTTACGTCGATAAGGATTAGATAAAAA  37632816   TATA-like promoter element

Query  101       GGAGGAAAAcccccccttcccccctc-ccccgtcccct----------ccccGACCGCCC  149
                  |||||||||||||||| |||||| | |||| |||||           ||||||||||||
Sbjct  37632817  CGAGGAAAACCCCCCCTACCCCCCCCTCCCCCTCCCCCAAAGCACCCGCCCCGACCGCCC  37632876

Query  150       GTGCGAGCGCACGCCGGAGGGCCCGTGGGAGGGATGGGTTCTTTTTATTTTAATCGTGAA  209
                 ||||||||||||| | ||||||||| |||||||||||||| ||||| |||||||||||||
Sbjct  37632877  GTGCGAGCGCACGTCCGAGGGCCCGCGGGAGGGATGGGTTTTTTTTCTTTTAATCGTGAA  37632936

Query  210       ATTGAAATTGAAAGAGTAATTACGTAATAAGCGGCAACTATGTTTTT--ATCGCTGATAC  267
                 |||    | |   |||  |||  ||||||||||||||| |||| ||   |||  ||   |
Sbjct  37632937  ATTCTTCTGGTG-GAGCGATTGTGTAATAAGCGGCAACAATGTATTGTAATCATTG---C  37632992

Query  268       GTGCGTACGAGGGAATGTGTAATTTGTTTTTTATCGGCGGTCGGGAATCGCGTGCGGGAA  327
                 |||||||||||||||||    |   | |||||  || ||| |||||||   |  ||||||
Sbjct  37632993  GTGCGTACGAGGGAATG----ACGCGGTTTTTTCCGCCGG-CGGGAATGAAGCACGGGAA  37633047

Query  328       CCAA-ATATTTTGTTATTGTCATTTTGAAGTTGATAAGAGTTTAAACAAGGTAGGTAAAT  386        TATA-like promoter element
                   |  ||| |||   ||||||||| |     |||||| |   ||||||||||  ||||||
Sbjct  37633048  GGACGATAATTTTCAATTGTCATTATATCTCTGATAAAAAGGTAAACAAGGTGTGTAAAT  37633107

Query  387       AAAAGGTGGCTCGAGTGCGGGATTCGTCGCAGT-TCAGtttttttGCCGGTATGTCTGCT  445        NS1 ORF ATG start codon
                 ||||   |  | ||||| ||||    ||||||| |  |||||||   ||  ||||| | |
Sbjct  37633108  AAAACCCGCGT-GAGTGAGGGAACGATCGCAGTATTCGTTTTTT---CGTGATGTCAGTT  37633163

Query  446       CATTTAAGCGACGATAACAGTTCTCAAGATTCATTTTCAGTGATAGATACAGATAGTAGT  505
                 ||  ||  |||||||   ||||||||| |||| |||||  | || ||||| |||   |||
Sbjct  37633164  CACCTATCCGACGATTCTAGTTCTCAAAATTCTTTTTCGATTATTGATACTGATTCAAGT  37633223

Query  506       GAATTCGTGCAAGTTGAACAATTATCCCCTAGTGAAGAAAGGTACGTTGAACATTGTATC  565
                 ||  | |||||||| ||||| ||||||||    || ||||| ||||| ||||||||||| 
Sbjct  37633224  GATCTTGTGCAAGTGGAACAGTTATCCCCGTCGGAGGAAAGATACGTGGAACATTGTATT  37633283

Query  566       GCAACAAACCAGCCTGTTTATTTAGTCAGGGACGGAGTAACAAAGTGTATCCCGATAGTT  625
                   ||| || || || ||||||||  | ||||| ||||| |||||||| || || |||
Sbjct  37633284  CAAACTAATCAACCAGTCTATTTAGTTCGCGACGGTGTAACTAAGTGTATTCCCATTGTT  37633343

Query  626       TGTGCAGAAGATGTCCTCaaaaaaaaGCCGGAAAACCCGTCAAACATCTCCGGAGACGGA  685        NS2 ORF ATG start codon
                 ||||| ||||||||| || |||||  |   ||||   |||||  | || ||||| |||||
Sbjct  37633344  TGTGCGGAAGATGTCTTCCAAAAAGGGAAAGAAAGTACGTCAGGCGTCGCCGGAAACGGA  37633403

Query  686       AATGCTTTCGAATTTGTCGGAAGTAGACAAACAGAAAAAAGTCCATATCTTGGAGAAGGA  745
                  |||||||| | ||||||  |||   |||||  ||| | || || |  || |||||||||
Sbjct  37633404  GATGCTTTCCAGTTTGTCTCAAGCCAACAAAACGAAGATAGCCCCTTCCTCGGAGAAGGA  37633463

Query  746       TCCTCCGGAGCCTCAAGCGGGTCCATCCCTCGAGATGACGGAAACAAGGAAGCAGGAAAT  805
                  | |||||| ||   ||| ||  | |||   ||  |||||||| |   |||| ||| |||
Sbjct  37633464  ACATCCGGAACCAGTAGCTGGAGCGTCCACGGAACTGACGGAAGCGCAGAAGAAGGTAAT  37633523

Query  806       TCAGGACTTTATCGATCGATCTCCCCTTTTGACAGCGATACAGGATTACTCCATGGAACA  865
                  || || || || ||||  || |||||  || |||| || ||||| | | | ||||| ||
```

```
Sbjct  37633524  ACAAGATTTCATAGATCAGTCACCCCTACTGTCAGCCATTCAGGACTTCACGATGGATCA  37633583

Query  866       CCCCCTCGGCCTTCTTCCGTTGCCTCGACTGATGTCCGAGTTCGTAGGCGTAATCGAAGC  925
                  || || || ||||||||  ||||||| || ||||| || ||||| ||   |||   |||
Sbjct  37633584  TCCTCTAGGGCTTCTTCCCCTGCCTCGTCTAATGTCGGAATTCGTGGGAATAACAGAAAG  37633643

Query  926       AAACGGATTGACGAATATTTCTCAGGAGCAGAAAGTTCTTTGGCGGGAAGCTCAAATAGC  985
                   |||||||||| ||||||||    ||| ||||| | ||| |||||||| || || || ||
Sbjct  37633644  CGACGGATTGACCAATATTTCAGCGGATCAGAAGGCTCTGTGGCGGGAGGCACAGATTGC  37633703

Query  986       TACCAAGAAAGTTTTGAAACCGTTTCTTTCGAAAAATTTAAAAGCTTATTCTGTGCAGAA  1045
                 ||  |||| ||||||||| || | |||||| | |||||||||   | |||||||| |||||
Sbjct  37633704  TATGAAGAGAGTTTTGAAGCCATATCTTTCCAGAAATTTAAAGACCTATTCTGTTCAGAA  37633763

Query  1046      TTTGGCCGACTGGATAATCACCGAAGGTACATATTACACGACATTTACCGAAATACAATT  1105
                 ||| || || ||||| || || || |||||||   | ||||| |||||| |||||||  ||
Sbjct  37633764  TTTAGCAGAATGGATCATAACAGACGGTACATGCTCCACGATATTTACAGAAATACGCTT  37633823

Query  1106      TCCGGGCGCTTTCGGGAAAATGATAGGGGAGTCGTACCCAAGGACACAGGCAACTCATCA  1165
                 ||||| |||||||||||| ||||| ||||| || ||||||||    ||||| ||||| ||
Sbjct  37633824  TCCGGCCGCTTTCGGGAAGATGATCGGGGAATCATACCCAAGAGTACAGGGAACTCGTCG  37633883

Query  1166      TCGGCATGGACATTCGTCGTCTTCCAGCACGGCCAGCATGTCCACGTCATCCACAAGTGC  1225
                  || | ||| | |||||||||||| |||||||   ||||   ||||||||||||||   ||||
Sbjct  37633884  GCGTCGTGGGCGTTCGTCGTCTTCGAGCACGGGAAGCACATCCACGTCATCCACCTGTGC  37633943

Query  1226      CGATACGAAAGGTCGTGGTGCTCGTGCGCACTCACGAAGAAAGCGGAAGAGCGATTCGGA  1285
                  |||| ||   | || ||||| || ||||| ||||| || ||||||||   | || ||||||
Sbjct  37633944  AGATATGAGCGATCATGGTGTTCATGCGCGCTCACAAAAAAAGCGGAGAAACGGTTCGGA  37634003

Query  1286      CGACGAATTGGAAGACATACTTTACGAGGCAGTAGGATCACAGAAGGCCACCTCGAACAT  1345
                 ||| || |||||||| || || | ||| ||||| || || || || ||||||||     ||
Sbjct  37634004  CGAAGAGTTGGAAGATATTCTCTTCGAAGCAGTCGGGTCTCAAAAAGCCACCTCAGCAAT  37634063

Query  1346      CTCGCAAATTATATACAAAAAGGGCGACGCAGGCTCCTTCAAGCTTTCCTCGCCGGGCGA  1405
                 |||||| | ||||||||||| |||    | |   || | ||||||||||| |||||| || ||
Sbjct  37634064  CTCGCAGACTATATACAAAAGGGGAAAAGACGGTTGCTTCAAGCTTTTCTCGCCAGGAGA  37634123

Query  1406      GAAAGGCGATTATCTCGTCAAGCTGGACTTTTACAAGACACGCAATCTTCGCAGAGTGGC  1465
                 |||||| || ||   | || ||||||||||| ||||| ||| | || ||   | ||||| ||
Sbjct  37634124  GAAAGGAGAATACTTGGTGAAGCTGGACTTCTACAAAACAAGGAACCTAAGAAGAGTTGC  37634183   5'-splice site of the B. tabaci transcript

Query  1466      AAAGGAGAACTGGTGGAAGGAGGCCGATTACCGGAGCACATTTCTTCTTTCTTACGCGGA  1525
                  || || || ||||||| ||| || || ||||||| ||||||   | ||||| ||||||||
Sbjct  37634184  GAAAGAAAATTGGTGGAGGGAAGCGGACTACCGGACCACATTCGTGCTTTCCTACGCGGA  37634243

Query  1526      ACCTGCCGCAAACAGCATCaaaaaaaTCCTGAAAGATCAAGTGAAGAAGATAGCGAAAAT  1585
                  ||||| |||||    || || || || || || || |||||||||||||||||   | ||
Sbjct  37634244  TCCTGCGGCAAATCAAATAAAGAAGATACTCAAGGAACAAGTGAAGAAGATAGCTCAGAT  37634303

Query  1586      TCCGGAGAGGAA  1597
                 ||||||  ||||
Sbjct  37634304  TCCGGACCGGAA  37634315
```

Range 2: 37632757 to 37632940

```
Identities     Gaps          Strand
157/184(85%)   11/184(5%)    Plus/Minus

Query  4911      GAACTACACGATTAAAATAAAAAGAACCCATCCCTCCCACGGGCCCTCCGGCGTGCGCTC  4970
                 ||| | ||||||||||| ||||| |||||||||||||| ||||||||| | |||||||||
Sbjct  37632940  GAATTTCACGATTAAAAGAAAAAAAACCCATCCCTCCCGCGGGCCCTCGGACGTGCGCTC  37632881

Query  4971      GCACGGGCGGTCggggaggggac---------gggggaggggggga--agggggggTTTTC  5019
                 |||||||||||||||| |||   |          ||||||||||||   |||||||||||||
Sbjct  37632880  GCACGGGCGGTCGGGGCGGGTGCTTTGGGGGAGGGGGAGGGGGGGGTAGGGGGGGTTTTC  37632821

Query  5020      CTCCGTTTTATATGATCCTTATCGACGTAACGACCCTAAAATCATGATCTACGATCGATT  5079
                 |||  |||||| | ||||||||||||||||||||||||||||||||||||| |||||||||
Sbjct  37632820  CTCGTTTTTATCTAATCCTTATCGACGTAACGACCCTAAAATCATGATCTTCGATCGATT  37632761

Query  5080      TTAT  5083
                 ||||
Sbjct  37632760  TTAT  37632757
```

Range 3: 37632758 to 37632823

```
Identities     Gaps          Strand
56/70(80%)     6/70(8%)      Plus/Plus

Query  5056      TAAAATCATGATCTACGATC--GATTTTATAGTCGTTACGTCATGATAAGGATCATATAA  5113
                 |||||||  |||| | ||||  |||||||  |||||||||||  ||||||||| | ||||
Sbjct  37632758  TAAAATC--GATCGAAGATCATGATTTTAGGGTCGTTACGTC--GATAAGGATTAGATAA  37632813

Query  5114      AACGGAGGAA  5123
                 ||  ||||||
Sbjct  37632814  AAACGAGGAA  37632823
```

Range 4: 37632758 to 37632823

```
Identities     Gaps          Strand
56/70(80%)     6/70(8%)      Plus/Minus

Query  1         TTCCTCCGTTTTATATGATCCTTATCATGACGTAACGACTATAAAATC--GATCGTAGAT  58
                 ||||||  |||||| | |||||||||  |||||||||||  |||||||   |||| | |||
Sbjct  37632823  TTCCTCGTTTTTATCTAATCCTTATC--GACGTAACGACCCTAAAATCATGATCTTCGAT  37632766

Query  59        CATGATTTTA  68
                 |  |||||||
Sbjct  37632765  C--GATTTTA  37632758
```

The genomic locus of the *B. tabaci* Q-type Almeria chromosome 10 carrying the 1.4 Kb sequence homologous to BtaDV

spanning an incomplete 5′-ITR starting with the incomplete flip-flop palindrome sequence, followed by the downstream hairpin-forming sequence, ITR sequence and half of the NS1 transcription unit

```
>OU963871.1:37632155-37636914 Bemisia tabaci genome assembly, chromosome: 10
ATATTTTATTGAGAGCTGCTTAGATTAAAGCAATTATGGAAAATTCTACCTCTTCTGCTGGGGTTATTGCCAAAAATTTCAAGAAAGCTGTAAATGCTAGGTTTCCTGATTACAAAACTGCTGTGGTACGTGCACTTTACATGTTATTGGATCCAAAATTCAAAGCATCTTCACTGCCTGAAACCCTC
AAAAACTTGGGTTGGAAGGCTGAAGACAAAAGCCACAGCGAAATTTGTAGACACGAATGAAGAATATTGGAAAAGTGTAAAGTTTCCAGTCTCAATAATTGCTAGGAAGGCAGAGGGCGCAAGCTTGAAAGATAAGGATAAAAAAGAAAAGAAAACATTCAATGCAATTTGCCAAGGTCAGTATGCAA
TGCATCGATTGCATATTTCTTTTGTCCTCAACTTAGTTGGTTCAAGGTCGATGTACCAGCCAGAATGAAAGCCTTTGACCACCAGTGATGAGTGAAAGACTAATAAATATTTTCATTCTTTCATAGGAACGTGCCTAAAGTTTTTTTTTTTTTTTTTTTTTTTTGAGTTTTTTTTTGGCTTCTATTCC
TTTATATAGATAAGATAGGAATCTACAGCCATCTATAGATAAAATCGATCGAAGATCATGATTTTAGGGTCGTTACGTCGATAAGGATTAGATAAAAACGAGGAAAACCCCCCCTACCCCCCCCTCCCCCTCCCCCAAAGCACCCGCCCCGACCGCCCGTGCGAGCGCACGTCCGAGGGCCCGCGGGA
GGATGGGTTTTTTTCTTTTAATCGTGAAATTCTTCTGGTGGAGCGATTGTGTAATAAGCGGCAACAATGTATTGTAATCATTGCGTGCGTACGAGGGAATGACGCGGTTTTTTCCGCCGGCGGGAATGAAGCACGGGAAGGACGATAATTTTCAATTGTCATTATATCTCTGATAAAAAGGTAAAC
AAGGTGTGTAAATAAAACCCGCGTGAGTGAGGGAACGATCGCAGTATTCGTTTTTTCGTGATGTCAGTTCACCTATCCGACGATTCTAGTTCTCAAAATTCTTTTTCGATTATTGATACTGATTCAAGTGATCTTGTGCAAGTGGAACAGTTATCCCCGTCGGAGGAAAGATACGTGGAACATTGTAT
TCAAACTAATCAACCAGTCTATTTAGTTCGCGACGGTGTAACTAAGTGTATTCCCATTGTTTGTGCGGAAGATGTCTTCCAAAAAGGGAAAGAAAGTACGTCAGGCGTCGCCGGAAACGGAGATGCTTTCCAGTTTGTCTCAAGCCAACAAAACGAAGATAGCCCCTTCCTCGGAGAAGGAACATCCG
GAACCAGTAGCTGGAGCGTCCACGGAACTGACGGAAGCGCAGAAGAAGGTAATACAAGATTTCATAGATCAGTCACCCCTACTGTCAGCCATTCAGGACTTCACGATGGATCATCCTCTAGGGCTTCTTCCCCTGCCTCGTCTAATGTCGGAATTCGTGGGAATAACAGAAAGCGACGGATTGACCAA
TATTTCAGCGGATCAGAAGGCTCTGTGGCGGGAGGCACAGATTGCTATGAAGAGAGTTTTGAAGCCATATCTTTCCAGAAATTTAAAGACCTATTCTGTTCAGAATTTAGCAGAATGGATCATAACAGACGGTACATGCTCCACGATATTTACAGAAATACGCTTTCCGGCCGCTTTCGGGAAGATGA
TCGGGGAATCATACCCAAGAGTACAGGGAACTCGTCGGCGTCGTGGGCGTTCGTCGTCTTCGAGCACGGGAAGCACATCCACGTCATCCACCTGTGCAGATATGAGCGATCATGGTGTTCATGCGCGCTCACAAAAAAAGCGGAGAAACGGTTCGGACGAAGAGTTGGAAGATATTCTCTTCGAAGCA
GTCGGGTCTCAAAAAGCCACCTCAGCAATCTCGCAGACTATATACAAAAGGGGAAAAGACGGTTGCTTCAAGCTTTTCTCGCCAGGAGAGAAAGGAGAATACTTGGTGAAGCTGGACTTCTACAAAACAAGGAACCTAAGAAGAGTTGCGAAAGAAAATTGGTGGAGGGAAGCGGACTACCGGACCAC
ATTCGTGCTTTCCTACGCGGATCCTGCGGCAAATCAAATAAAGAAGATACTCAAGGAACAAGTGAAGAAGATAGCTCAGATTCCGGACCGGAAAGAAAAGAAAAGAAAAGAAAACATTCAATGCAATTTGCCAAGGTCAGTATGCAATGCATCGATTGCATATTTCTTTTGTCCTCAACTTAGTTGGT
TCAAGGTCGATGTACCAGCCAGAATGAAAGCCTTTGACCACCAGTGATGAGTGAAAGACTAATAAATATTTTCATTCTTTCATAGGAACGTGCCTAAAGTTCATAAATTCAACGTAGAAGATAAAAGAACCGATGCCCCACGAGAAAAAGAGGAGGGACAACCCCCTTTCTGGTCCAGTCTAGATGAA
TTTCCTACACAAAAATCTGGAGAATAAGACTTGTTGAAAGCAAAACTGATTTGCAATTTGCAAATTACTTAATTGCATAGCTGATGAGAAGCTTAATGCTTTTGAATTTTTGAAAGAAACTGGGAGAAACACCCATAAAACCCTTCGCCTGGTAGCAAAGGAATATTTCGCTAAACAAGGAGTTGCAT
GTTGAATAAATCTGCAGCTTCAATGCTCAAAGATAGGAAAGGAATTTTGATGCCTGAAGCTATCTCATGAGACAAAATGCAACATTGCCTGGCGCTGACTTTAATAGTGTAGTAAATAATTATGATGATTATGATATATATAAAAGCATAATAGTAAGTAAGAGATAAGATAAGAGATAAGTTGTTAG
ATAAGTTCGCTCTCATAAGCATGAAATTGTCTAGACGGGTGCAAATGATGTAAATAGGAATGAAGCCACATTTTTCTTAAAATCGTTACTAACTTTTCTTAATACTTCAGCCACTCTTGATGCACGGGAAATTTCCAAGAGGCACCAAATTGTAACTCTGCCCTCAAAATTAAAAAAATCAAAATCTG
GATTTTGACCTTGCAAATCGTGGTCCTGGCAATATTATGCACATGAGATGGCTCACGACGGCCAATCGAGCACTGAGACTGTATGTAGGAACCAAAAATCCCGATTCATCCCTGGTGACCATCGCTACGTACATAATGAAAGTATACGCGCCGAGCTGGTTTGCAATTAAAACCCATTCATCATGCAC
TGCAGGGGCCAAACATCTGTTCAAAATAATTGAATTGACTAGAGTAGCACAGAAATCAGAGACATTGTCCACCCTGTCATTCAAAGAAATGCTTTTTTCGCCCATCCAGAAAACATCCTGCTTGCTATGCTCAACGATGCATTACAAGATGTTAAACAATTGGCCGTGCAAAAAATCATGAACGCTCG
GAGGACTAAAAGCTCAACAGTACGAAAATTCAAAGTGCCCCTTCTCAATTTCAACGCGGAAACCTACAGTGAAATGATTTTCTGGAACGAAGTCACAGTAACCCAGCCGCCTATCTTACAGTATTTCTCCGAAGAAGATCTAGAAAATTTGGCAAGAGAAAAGGACCTCCAGAATTACAGTTTTTACA
AATATCCTTGTCACACGCAAAGTGTCGAAAGATGCGTCAAGATTGTCACCGAGGCATCCGCATTAGTCTGTGGAGGAGAGGAAAGAGATGGGCTGATTCGATCCAAGTTGAAGGCGAGATTGTTGATGCCTGCTTTTGAACCCAAAAGCCAATTTAAATTCGTTAAATGAAGGTGAGCCAAATTATTT
GTAGCTAGCTACAAATGCCCCATACTCTGAGGAAGAATTCATCAACCTATAGACCCTGTTTCGGATTACCGTATAGCTCCGCAGATAAGACGCACTTTTTTCCCCATTTAAGCGCCCAAAATCGGGGGTGCGTCTTATACGCGAAACTCGTGCAAATTGCATACCTCACAGGCGCGCCTAAGCAGAT
AATCGTTCCCCCTGGCCCCTCCTTGCTATATCTCACAACCCCAACGCGTACCATCCTCTTATCTCTATAAACACACTGGTCTGAGTCTAACTGAACGATAGTGACCCATTCCTGCCAACCGACACTCTTTTTTTGCTTTTGAAGGAGTGCAAACAAAGCATAAATTGCCCAACGCATGCCTTCTTTGC
AAAACCGCGAACGTAAACACCTTTTTCGACTCGCAATCTGACCAGAGCTAGAATTGAATCTGGCCCATGGTGGGGGGAAGCGTCAGGAAATAGGCCGCGTCTCCTTCGATTCATCGCCATCGAACAATCGAAAGTGGCCACCTGTTGACAAGGTCAATGGCTCACTGCATTTTCGGCACGCGCCGCAT
GCAGCGCTGGCGCGGCGCAGCGGAAGACATGTCAATCGCCCACTCAATCCCCGCTGTTATCGCAATTTTAATCTCCCTTTCTTAAAGATTTGAGCTTCCTTTGAGGAAAGATCCTTTTTGCTTTAATTCTTTTTTCGTATCAATGGCTCTGCCTTTCCCTCTTTTCAAACTCGACGCTGCTGCTTTGA
GAAAGGGTACAGTAGGGGGTGGGGTTGGGACGATGACCGCGGGTCTTAATTCAAACAATCGGCGTGATTTCACGCCTCATTAGGTGTTGACACCCTCCCTTTCTTTTATTAACCTATGTTTCCTATTTTTATTGTATCTTGTGCCTACTACTATCATGTACCTATCTAGCATGTATAACCTGATCTGT
ATGATGGCCATGTACACAGCTCATTTACCAATTGTAAAAATAAAATAAAATGAGCATGTA
```

```
>Spliced mRNA
CAAAGCACCCGCCCCGACCGCCCGTGCGAGCGCACGTCCGAGGGCCCGCGGGAGGGATGGGTTTTTTTCTTTTAATCGTGAAATTCTTCTGGTGGAGCGATTGTGTAATAAGCGGCAACAATGTATTGTAATCATTGCGTGCGTACGAGGGAATGACGCGGTTTTTTCCGCCGGCGGGAATGAAGCA
CGGGAAGGACGATAATTTTCAATTGTCATTATATCTCTGATAAAAAGGTAAACAAGGTGTGTAAATAAAACCCGCGTGAGTGAGGGAACGATCGCAGTATTCGTTTTTTCGTGATGTCAGTTCACCTATCCGACGATTCTAGTTCTCAAAATTCTTTTTCGATTATTGATACTGATTCAAGTGATCTT
GTGCAAGTGGAACAGTTATCCCCGTCGGAGGAAAGATACGTGGAACATTGTATTCAAACTAATCAACCAGTCTATTTAGTTCGCGACGGTGTAACTAAGTGTATTCCCATTGTTTGTGCGGAAGATGTCTTCCAAAAAGGGAAAGAAAGTACGTCAGGCGTCGCCGGAAACGGAGATGCTTTCCAGTT
TGTCTCAAGCCAACAAAACGAAGATAGCCCCTTCCTCGGAGAAGGAACATCCGGAACCAGTAGCTGGAGCGTCCACGGAACTGACGGAAGCGCAGAAGAAGGTAATACAAGATTTCATAGATCAGTCACCCCTACTGTCAGCCATTCAGGACTTCACGATGGATCATCCTCTAGGGCTTCTTCCCCTG
CCTCGTCTAATGTCGGAATTCGTGGGAATAACAGAAAGCGACGGATTGACCAATATTTCAGCGGATCAGAAGGCTCTGTGGCGGGAGGCACAGATTGCTATGAAGAGAGTTTTGAAGCCATATCTTTCCAGAAATTTAAAGACCTATTCTGTTCAGAATTTAGCAGAATGGATCATAACAGACGGTAC
ATGCTCCACGATATTTACAGAAATACGCTTTCCGGCCGCTTTCGGGAAGATGATCGGGGAATCATACCCAAGAGTACAGGGAACTCGTCGGCGTCGTGGGCGTTCGTCGTCTTCGAGCACGGGAAGCACATCCACGTCATCCACCTGTGCAGATATGAGCGATCATGGTGTTCATGCGCGCTCACAAA
AAAAGCGGAGAAACGGTTCGGACGAAGAGTTGGAAGATATTCTCTTCGAAGCAGTCGGGTCTCAAAAAGCCACCTCAGCAATCTCGCAGACTATATACAAAAGGGGAAAAGACGGTTGCTTCAAGCTTTTCTCGCCAGGAGAGAAAGGAGAATACTTGGTGAAGCTGGACTTCTACAAAACAAGGAAC
CTAAGAAGAAAAACATCCTGCTTGCTATGCTCAACGATGCATTACAAGATGTTAAACAATTGGCCGTGCAAAAAATCATGAACGCTCGGAGGACTAAAAGCTCAACAGTACGAAAATTCAAAGTGCCCCTTCTCAATTTCAACGCGGAAACCTACAGTGAAATGATTTTCTGGAACGAAGTCACAGTA
ACCCAGCCGCCTATCTTACAGTATTTCTCCGAAGAAGATCTAGAAAATTTGGCAAGAGAAAAGGACCTCCAGAATTACAGTTTTTACAAATATCCTTGTCACACGCAAAGTGTCGAAAGATGCGTCAAGATTGTCACCGAGGCATCCGCATTAGTCTGTGGAGGAGAGGAAAGAGATGGGCTGATTCG
ATCCAAGTTGAAGGCGAGATTGTTGATGCCTGCTTTTGAACCCAAAAGCCAATTTAAATTCGTTAAATGAAGGTGAGCCAAATTATTTGTAGCTAGCTACAAATGCCCCATACTCTGAGGAAGAATTCATCAACCTATAGACCCTGTTTCGGATTACCG
```

>NS1 coding sequence C-terminally extended with the host coding sequence, following splicing of the GT-AG intron

ATGTCAGTTCACCTATCCGACGATTCTAGTTCTCAAAATTCTTTTTCGATTATTGATACTGATTCAAGTGATCTTGTGCAAGTGGAACAGTTATCCCCGTCGGAGGAAAGATACGTGGAACATTGTATTCAAACTAATCAACCAGTCTATTTAGTTCGCGACGGTGTAACTAAGTGTATTCCCATTGT
TTGTGCGGAAGATGTCTTCCAAAAAGGGAAAGAAAGTACGTCAGGCGTCGCCGGAAACGGAGATGCTTTCCAGTTTGTCTCAAGCCAACAAAACGAAGATAGCCCCTTCCTCGGAGAAGGAACATCCGGAACCAGTAGCTGGAGCGTCCACGGAACTGACGGAAGCGCAGAAGAAGGTAATACAAGAT
TTCATAGATCAGTCACCCCTACTGTCAGCCATTCAGGACTTCACGATGGATCATCCTCTAGGGCTTCTTCCCCTGCCTCGTCTAATGTCGGAATTCGTGGGAATAACAGAAAGCGACGGATTGACCAATATTTCAGCGGATCAGAAGGCTCTGTGGCGGGAGGCACAGATTGCTATGAAGAGAGTTTT
GAAGCCATATCTTTCCAGAAATTTAAAGACCTATTCTGTTCAGAATTTAGCAGAATGGATCATAACAGACGGTACATGCTCCACGATATTTACAGAAATACGCTTTCCGGCCGCTTTCGGGAAGATGATCGGGGAATCATACCCAAGAGTACAGGGAACTCGTCGGCGTCGTGGGCGTTCGTCGTCTT
CGAGCACGGGAAGCACATCCACGTCATCCACCTGTGCAGATATGAGCGATCATGGTGTTCATGCGCGCTCACAAAAAAAGCGGAGAAACGGTTCGGACGAAGAGTTGGAAGATATTCTCTTCGAAGCAGTCGGGTCTCAAAAAGCCACCTCAGCAATCTCGCAGACTATATACAAAAGGGGAAAAGAC
GGTTGCTTCAAGCTTTTCTCGCCAGGAGAGAAAGGAGAATACTTGGTGAAGCTGGACTTCTACAAAACAAGGAACCTAAGAAGAAAAACATCCTGCTTGCTATGCTCAACGATGCATTACAAGATGTTAAACAATTGGCCGTGCAAAAAATCATGAACGCTCGGAGGACTAAAAGCTCAACAGTACGA
AAATTCAAAGTGCCCCTTCTCAATTTCAACGCGGAAACCTACAGTGAAATGATTTTCTGGAACGAAGTCACAGTAACCCAGCCGCCTATCTTACAGTATTTCTCCGAAGAAGATCTAGAAAATTTGGCAAGAGAAAAGGACCTCCAGAATTACAGTTTTTACAAATATCCTTGTCACACGCAAAGTGT
CGAAAGATGCGTCAAGATTGTCACCGAGGCATCCGCATTAGTCTGTGGAGGAGAGGAAAGAGATGGGCTGATTCGATCCAAGTTGAAGGCGAGATTGTTGATGCCTGCTTTTGAACCCAAAAGCCAATTTAAATTCGTTAAATGA

>incomplete hairpin of the integrant with the upstream host sequence

CTTTATATAGATAAGATAGGAATCTACAGCCATCTATAGATAAAATCGATCGAAGATCATGATTTTAGGGTCGTTACGTCGATAGGATTAGATAAAAACGAGGAA

>Incomplete 5'-hairpin of the integrant (with a missing part of the flip-flop palindrome)

ATAAAATCGATCGAAGATCATGATTTTAGGGTCGTTACGTCGATAGGATTAGATAAAAACGAGGAA

```
                       >>>>>>>> reconstructed sequence >>>>>>>>>                 <<<<<<<<<<<<<<<<<<<<<<<<<<<<<<<<<<<<<<<<<<<

Complete 5'-hairpin  ttcctc-gttttatctaatcc-tatcatgacgtaacgactATAAAATC--GATCGAAGATCATGATTTTAGGGTCGTTACGTC--GATA-GGATTAGATAAAAACG-AGGAA
                     |||||| ||||| || | |||| |||||||||||||||||||||||||  ||||| ||||||||||||||||||||||||||  |||| |||| | || |||||| |||||
BtaDV 5'-hairpin   1 TTCCTCCGTTTT-ATATGATCCTTATCATGACGTAACGACTATAAAATC--GATCGTAGATCATGATTTTAGGGTCGTTACGTC--GATAAGGATCATAT-AAAACGGAGGAA    107
                     |||||||||||| ||||||||||||||  |||||||||||..|||||||  ||||| | |||  ||||||||..||||||||||  |||||||||||||| ||||||||||||
BtaDV 3'-hairpin   1 TTCCTCCGTTTT-ATATGATCCTTATC--GACGTAACGACCCTAAAATCATGATCTACGATC--GATTTTATAGTCGTTACGTCATGATAAGGATCATAT-AAAACGGAGGAA    107

                     >>>>> inverted repeat >>>>>  >>>>>>>>>>>>>>>> flip-flop palindrome <<<<<<<<<<<<<<<<<<<<<<<< inverted repeat <<<<<
```

>complete reconstructed 5'hairpin

ttcctcgttttatctaatcctatcatgacgtaacgactaTAAAATCGATCGAAGATCATGATTTTAGGGTCGTTACGTCGATAGGATTAGATAAAAACGAGGAA

Complete (reconstructed) 5'-hairpin of the integrated BtaDV

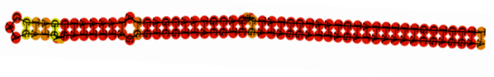

5'-hairpin of the episomal BtaDV

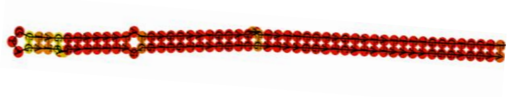

BtaDV-related EVEs in other chromosomes of the *B. tabaci* Q-type Almeria genome

Bemisia tabaci chromosome 6, PGI_BMITA_v3

```
Sequence ID: NC_092798.1 Length: 54143761 Number of Matches: 3
Identities     Gaps           Strand
269/376(72%)   4/376(1%)      Plus/Plus
Features:
31136 bp at 5' side: uncharacterized protein
8404 bp at 3' side: facilitated trehalose transporter tret1 isoform x2

Query  1125      ATGATAGGGGAGTCGTACCCAAGGACACAGGCAACTCATCATCGGCATGGACATTCGTCG  1184
                 ||||| | ||| || |||||||   |||||| ||| |   | ||   | ||| || ||||||
Sbjct  36735104  ATGATGGCGGAATCTTACCCAAAAGCACAGGGAACGCGGCGTCAACGTGGTCAATCGTCG  36735163

Query  1185      TCTTCCAGCACGGCCAGCATGTCCACGTCATCCACAAGTGCCGATACGAAAGGTCGTGGT  1244
                 | | | | |||||    || ||| |  || |||||    || |||||||| || || ||||
Sbjct  36735164  TGTGCGAACACGGAAGCCACGTCTATCTCCTCCACCTATGACGATACGACAGATCCTGGT  36735223

Query  1245      GCTCGTGCGCACTCACGAAGAAAGCGGAA-GAGCGATTCGGACGACGAATTGGAAGACAT  1303
                 ||||||| ||  |||  || || |||||| ||| | | ||||||||||||||| || ||
Sbjct  36735224  GCTCGTGTGCGGTCAAAAA-AAGGCGGAAAGAGTGCT-CGGACGACGAATTGGACGATAT  36735281

Query  1304      ACTTTACGAGGCAGTAGGATCACAGAAGGCCACCTCGAACATCTCGCAAATTATATACAA  1363
                 ||| | ||| ||| | || || |  |||  ||| || || ||||| ||| |||| |||||
Sbjct  36735282  ACTATTCGAAGCATTCGGCTCTCTAAAGAGCACATCAAAAATCTCTCAAGTTATCTACAA  36735341

Query  1364      AAAGGGCGACGCAGGCTCCTTCAAGCTTTCCTCGCCGGGCGAGAAAGGCGATTATCTCGT  1423
                 |||||||  || ||| || ||||||||||  || || |||| ||| || |||||   |  |
Sbjct  36735342  AAAGGGCAGCGAAGGGTCATTCAAGCTTTTTCTCCAGGCGGGAATGGAGATTACATGAT  36735401

Query  1424      CAA-GCTGGACTTTTACAAGACACGCAATCTTCGCAGAGTGGCAAAGGAGAACTGGTGGA  1482
                 ||| | | ||| | |||   |||||  | ||  | ||||    | ||||| ||||||||||
Sbjct  36735402  CAAGGTTAGACCTGTACCGCACACGAGAACTGAGGAGAGCACCGAAGGAAAACTGGTGGA  36735461

Query  1483      AGGAGGCCGATTACCG  1498
                 |  | || || |||||
Sbjct  36735462  AACAAGCGGACTACCG  36735477

Range 2: 34231435 to 34231522
Identities     Gaps           Strand
67/90(74%)     2/90(2%)       Plus/Minus

Query  2025      CAGGAAAAAAtttttttttGATGCAGTGACACAAGCTATGATAAATTGTGGTCACGTCG  2084
                 ||||||||||  |||||||  ||   ||  | ||||||||||| ||| | ||   |  | |
Sbjct  34231522  CAGGAAAAAACGTTTTTTT--ATTGTGTCGCGCAAGCTATGATTAATAGCGGGTATATTG  34231465

Query  2085      GAAATTTTAACAGATTTTGTAACTTTCCAT  2114
                   |||||||||||||||| |  ||||||||
Sbjct  34231464  ATAATTTTAACAGATTTTCTTCCTTTCCAT  34231435


Range 3: 34225338 to 34225399
Identities     Gaps           Strand
51/64(80%)     4/64(6%)       Plus/Minus

Query  1985      CCAAAAGTTAATACTTTGTTA--ATTGCTGGTCCTGCTAATTCAGGAAAAAAttttttt  2042
                 || ||||| |||||| ||||   ||||  ||| || | || ||||||||||||| ||||
Sbjct  34225399  CCCAAAGTAAATACTCTGTTCGTATTG--GGTGCTCCAAACTCAGGAAAAAAATTTCTTTT  34225342

Query  2043      ttGA  2046
                 ||||
Sbjct  34225341  TTGA  34225338
```

Bemisia tabaci chromosome 4, PGI_BMITA_v3

```
Sequence ID: NC_092796.1 Length: 62539693 Number of Matches: 2
Identities     Gaps           Strand
160/231(69%)   10/231(4%)     Plus/Plus
Features:
10979 bp at 5' side: uncharacterized protein
32256 bp at 3' side: uncharacterized protein

Query  2019      CTAATTCAGGAAAAAAtttttttttGATGCAGTGACACAAGCTATGATAAATTGTGGTC  2078
                 ||||| | ||||||||||||||||||||   ||  ||||   |||| |||||||||| |
Sbjct  45896860  CTAATGCTGGAAAAAAATTTTTTTTTGATTGTGTTGCACAGTTTATGTTAAATTGTGGAC  45896919

Query  2079      ACGTCGGAAATTTTAACAGATTTTGTAACTTTCCATTACAAGA--GTGCAACATGAAAAG  2136
                 | |  ||||| |  || | |  ||||   |||||||||   ||  ||| || |   |||
Sbjct  45896920  ATGCTGGAAACTCAAATAAAAATTGTGCTTTTCCATTAATGGAATGTGTAAAA---AAAC  45896976

Query  2137      GATC--TTATTATGGAATGAACCGGTTTGCGAACCATCCGCGGTGGAA-ACTATAAAAAT  2193
                 |||   ||||| |||||||||||   ||| ||| |         | ||| | | ||||||
Sbjct  45896977  GATTAATTATT-TGGAATGAACCTTCTTGTGAATCTGATTTTTTTGAAGATTGTAAAAA-  45897034

Query  2194      GCTTTTCGCTGGGGACCCTTGCCCCGCAAAAATTAAATACAAAAATGATGC  2244
                 | | |||||||| || || |    || |||| | ||||| |||||||||||
Sbjct  45897035  GTTATTCGCTGGAGATCCGTTAAACGTAAAAGTAAAATATAAAAATGATGC  45897085

Range 2: 22232840 to 22232870
Identities     Gaps           Strand
30/32(94%)     1/32(3%)       Plus/Minus
```

```
Query  4207      TTTCGTTTTCCTCCTTCAGAATTTCCAAAATC  4238
                 |||| ||||||||||||||||||| |||||||
Sbjct  22232870  TTTCTTTTTCCTCCTTCAGAATTT-CAAAATC  22232840
```

Bemisia tabaci chromosome 2, PGI_BMITA_v3

```
Sequence ID: NC_092794.1 Length: 79695644 Number of Matches: 1
Identities      Gaps            Strand
76/107(71%)     0/107(0%)       Plus/Minus
Features:
172954 bp at 5' side: uncharacterized protein
72642 bp at 3' side: serine/threonine-protein kinase gl21140

Query  1985      CCAAAAGTTAATACTTTGTTAATTGCTGGTCCTGCTAATTCAGGAAAAAAtttttttttt  2044
                 |||||||| || ||| | || ||    ||| |  | ||||| || |||||||||||||||
Sbjct  32060661  CCAAAAGTAAACACTCTCTTCATTTTAGGTGCCCCGAATTCGGGTAAAAATTTTTTTTTC  32060602

Query  2045      GATGCAGTGACACAAGCTATGATAAATTGTGGTCACGTCGGAAATTT  2091
                 |||    | | |||   |||||||||||  || ||  | ||||| ||
Sbjct  32060601  GATAGTATAATACACCTTATGATAAATTACGGACAGATGGGAAACTT  32060555
```

BtaDV-related EVEs in the unannotated genome assembly of *B. tabaci* MED pooled male and female from China (BioProject: PRJNA553778)

Descriptions | Graphic Summary | Alignments

Sequences producing significant alignments — Download — Manage columns — Show 100

select all *7 sequences selected* — GenBank — Graphics — Distance tree of results — MSA Viewer

	Description	Max Score	Total Score	Query Cover	E value	Per. Ident	Acc. Len	Accession
☑	Bemisia tabaci isolate Med HIC_SCAFFOLD_10_PILON_PILON_PI...	1049	1486	48%	0.0	75.21%	43320628	VMOE01000001.1
☑	Bemisia tabaci isolate Med HIC_SCAFFOLD_1_PILON_PILON_PIL...	181	300	10%	7e-43	71.28%	52489030	VMOE01000720.1
☑	Bemisia tabaci isolate Med HIC_SCAFFOLD_7_PILON_PILON_PIL...	100	247	10%	2e-18	77.60%	47292472	VMOE01001335.1
☑	Bemisia tabaci isolate Med HIC_SCAFFOLD_920_PILON_PILON_P...	59.9	59.9	2%	5e-06	75.82%	9147	VMOE01001451.1
☑	Bemisia tabaci isolate Med HIC_SCAFFOLD_2_PILON_PILON_PIL...	54.5	54.5	2%	2e-04	71.03%	78263488	VMOE01000002.1
☑	Bemisia tabaci isolate Med HIC_SCAFFOLD_603_PILON_PILON_P...	51.8	103	2%	8e-04	70.16%	11440	VMOE01001129.1
☑	Bemisia tabaci isolate Med HIC_SCAFFOLD_4_PILON_PILON_PIL...	51.8	103	2%	8e-04	70.16%	43304008	VMOE01001022.1

Distribution of the top 17 Blast Hits on 7 subject sequences

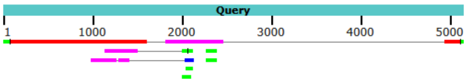

Bemisia tabaci isolate Med HIC_SCAFFOLD_10_PILON_PILON_PILON, whole genome shotgun sequence

```
Sequence ID: VMOE01000001.1 Length: 43320628 Number of Matches: 5
Range 1: 4838504 to 4840059
Identities      Gaps            Strand
1180/1569(75%)  25/1569(1%)     Plus/Minus

Query  41        ATAAAATCGATCGTAGATCATGATTTTAGGGTCGTTACGTCGATAAGGATCATATAAAAC  100
                 ||||||||||||| |||||||||||||||||||||||||||||||||||| | ||||||
Sbjct  4840059   ATAAAATCGATCGAAGATCATGATTTTAGGGTCGTTACGTCGATAAGGATTAGATAAAAA  4840000

Query  101       GGAGGAAAAcccccccttcccccc-----tccccc---gtcccctccccGACCGCCCGTG  152
                  |||||||||||||||| ||||||     ||||||   |  ||| |||||||||||||||
Sbjct  4839999   CGAGGAAAACCCCCCCTACCCCCCCCCCCTCCCCCAAAGCACCCGCCCCGACCGCCCGTG  4839940

Query  153       CGAGCGCACGCCGGAGGGCCCGTGGGAGGGATGGGTTCTTTTTATTTTAATCGTGAAATT  212
                 |||||||||| | ||||||||| |||||||||||||| ||||| ||||||||||||||||
Sbjct  4839939   CGAGCGCACGTCCGAGGGCCCGCGGGAGGGATGGGTTTTTTTTCTTTTAATCGTGAAATT  4839880

Query  213       GAAATTGAAAGAGTAATTACGTAATAAGCGGCAACTATGTTTTT--ATCGCTGATACGTG  270
                     | |   |||  |||  ||||||||||||||| |||| ||   |||  ||   ||||
Sbjct  4839879   CTTCTGGTG-GAGCGATTGTGTAATAAGCGGCAACAATGTATTGTAATCATTG---CGTG  4839824

Query  271       CGTACGAGGGAATGTGTAATTTGTTTTTTATCGGCGGTCGGGAATCGCGTGCGGGAACCA  330
                 ||||||||||||||    |   | |||||  || ||| |||||||   |  ||||||  |
Sbjct  4839823   CGTACGAGGGAATG----ACGCGGTTTTTTCCGCCGG-CGGGAATGAAGCACGGGAAGGA  4839769

Query  331       A-ATATTTTGTTATTGTCATTTTGAAGTTGATAAGAGTTTAAACAAGGTAGGTAAATAAA  389
                   ||| |||   ||||||||| |     |||||| |   ||||||||||  |||||||||
Sbjct  4839768   CGATAATTTTCAATTGTCATTATATCTCTGATAAAAAGGTAAACAAGGTGTGTAAATAAA  4839709

Query  390       AGGTGGCTCGAGTGCGGGATTCGTCGCAGT-TCAGtttttttGCCGGTATGTCTGCTCAT  448
                 |   |  | ||||| ||||    ||||||| |  |||||||   ||  ||||| | |||
Sbjct  4839708   ACCCGCGT-GAGTGAGGGAACGATCGCAGTATTCGTTTTTT---CGTGATGTCAGTTCAC  4839653

Query  449       TTAAGCGACGATAACAGTTCTCAAGATTCATTTTCAGTGATAGATACAGATAGTAGTGAA  508
                  ||  |||||||   ||||||||| |||| |||||  | || ||||| |||   |||||
Sbjct  4839652   CTATCCGACGATTCTAGTTCTCAAAATTCTTTTTCGATTATTGATACTGATTCAAGTGAT  4839593

Query  509       TTCGTGCAAGTTGAACAATTATCCCCTAGTGAAGAAAGGTACGTTGAACATTGTATCGCA  568
                  | |||||||| ||||| ||||||||    || ||||| ||||| |||||||||||   |
Sbjct  4839592   CTTGTGCAAGTGGAACAGTTATCCCCGTCGGAGGAAAGATACGTGGAACATTGTATTCAA  4839533

Query  569       ACAAACCAGCCTGTTTATTTAGTCAGGGACGGAGTAACAAAGTGTATCCCGATAGTTTGT  628
                 || || || || || ||||||||  | ||||| ||||| |||||||| || || ||||||
Sbjct  4839532   ACTAATCAACCAGTCTATTTAGTTCGCGACGGTGTAACTAAGTGTATTCCCATTGTTTGT  4839473

Query  629       GCAGAAGATGTCCTCaaaaaaaaGCCGGAAAACCCGTCAAACATCTCCGGAGACGGAAAT  688
                 || ||||||||| || |||||  |   ||||   |||||  | || ||||| ||||| ||
Sbjct  4839472   GCGGAAGATGTCTTCCAAAAAGGGAAAGAAAGTACGTCAGGCGTCGCCGGAAACGGAGAT  4839413

Query  689       GCTTTCGAATTTGTCGGAAGTAGACAAACAGAAAAAAGTCCATATCTTGGAGAAGGATCC  748
                 |||||| | ||||||  |||   |||||  ||| | || || |  || ||||||||| |
Sbjct  4839412   GCTTTCCAGTTTGTCTCAAGCCAACAAAACGAAGATAGCCCCTTCCTCGGAGAAGGAACA  4839353

Query  749       TCCGGAGCCTCAAGCGGGTCCATCCCTCGAGATGACGGAAACAAGGAAGCAGGAAATTCA  808
                 |||||| ||   ||| ||  | |||   ||  |||||||| |   |||| ||| ||| ||
Sbjct  4839352   TCCGGAACCAGTAGCTGGAGCGTCCACGGAACTGACGGAAGCGCAGAAGAAGGTAATACA  4839293

Query  809       GGACTTTATCGATCGATCTCCCCTTTTGACAGCGATACAGGATTACTCCATGGAACACCC  868
                  || || || ||||  || |||||  || |||| || ||||| | | | ||||| || ||
Sbjct  4839292   AGATTTCATAGATCAGTCACCCCTACTGTCAGCCATTCAGGACTTCACGATGGATCATCC  4839233

Query  869       CCTCGGCCTTCTTCCGTTGCCTCGACTGATGTCCGAGTTCGTAGGCGTAATCGAAGCAAA  928
                  || || ||||||||  ||||||| || ||||| || ||||| ||  |||  |||    |
Sbjct  4839232   TCTAGGGCTTCTTCCCCTGCCTCGTCTAATGTCGGAATTCGTGGGAATAACAGAAAGCGA  4839173

Query  929       CGGATTGACGAATATTTCTCAGGAGCAGAAAGTTCTTTGGCGGGAAGCTCAAATAGCTAC  988
                 ||||||||| ||||||||   ||| ||||| | ||| |||||||| || || || ||||
Sbjct  4839172   CGGATTGACCAATATTTCAGCGGATCAGAAGGCTCTGTGGCGGGAGGCACAGATTGCTAT  4839113

Query  989       CAAGAAAGTTTTGAAACCGTTTCTTTCGAAAAATTTAAAAGCTTATTCTGTGCAGAATTT  1048
                  |||| ||||||||| || | |||||| | |||||||||  | |||||||| ||||||||
Sbjct  4839112   GAAGAGAGTTTTGAAGCCATATCTTTCCAGAAATTTAAAGACCTATTCTGTTCAGAATTT  4839053

Query  1049      GGCCGACTGGATAATCACCGAAGGTACATATTACACGACATTTACCGAAATACAATTTCC  1108
                  || || ||||| || || || |||||||   | ||||| |||||| |||||||  |||||
Sbjct  4839052   AGCAGAATGGATCATAACAGACGGTACATGCTCCACGATATTTACAGAAATACGCTTTCC  4838993

Query  1109      GGGCGCTTTCGGGAAAATGATAGGGGAGTCGTACCCAAGGACACAGGCAACTCATCATCG  1168
                 || |||||||||||| ||||| ||||| || ||||||||   ||||| ||||| ||  ||
Sbjct  4838992   GGCCGCTTTCGGGAAGATGATCGGGGAATCATACCCAAGAGTACAGGGAACTCGTCGGCG  4838933
```

```
Query  1169     GCATGGACATTCGTCGTCTTCCAGCACGGCCAGCATGTCCACGTCATCCACAAGTGCCGA  1228
                 | ||| | |||||||||||| |||||||  ||||  ||||||||||||||  |||| ||
Sbjct  4838932  TCGTGGGCGTTCGTCGTCTTCGAGCACGGGAAGCACATCCACGTCATCCACCTGTGCAGA  4838873

Query  1229     TACGAAAGGTCGTGGTGCTCGTGCGCACTCACGAAGAAAGCGGAAGAGCGATTCGGACGA  1288
                || ||  | || ||||| || ||||| ||||| || ||||||||  | || |||||||||
Sbjct  4838872  TATGAGCGATCATGGTGTTCATGCGCGCTCACAAAAAAAGCGGAGAAACGGTTCGGACGA  4838813

Query  1289     CGAATTGGAAGACATACTTTACGAGGCAGTAGGATCACAGAAGGCCACCTCGAACATCTC  1348
                 || |||||||| || || | ||| ||||| || || || || ||||||||     |||||
Sbjct  4838812  AGAGTTGGAAGATATTCTCTTCGAAGCAGTCGGGTCTCAAAAAGCCACCTCAGCAATCTC  4838753

Query  1349     GCAAATTATATACAAAAAGGGCGACGCAGGCTCCTTCAAGCTTTCCTCGCCGGGCGAGAA  1408
                ||| | ||||||||||| |||  | |  || | ||||||||||| |||||| || |||||
Sbjct  4838752  GCAGACTATATACAAAAGGGGAAAAGACGGTTGCTTCAAGCTTTTCTCGCCAGGAGAGAA  4838693

Query  1409     AGGCGATTATCTCGTCAAGCTGGACTTTTACAAGACACGCAATCTTCGCAGAGTGGCAAA  1468
                ||| || ||  | || ||||||||||| ||||| ||| | || ||  | ||||| || ||
Sbjct  4838692  AGGAGAATACTTGGTGAAGCTGGACTTCTACAAAACAAGGAACCTAAGAAGAGTTGCGAA  4838633

Query  1469     GGAGAACTGGTGGAAGGAGGCCGATTACCGGAGCACATTTCTTCTTTCTTACGCGGAACC  1528
                 || || ||||||| ||| || || ||||||| ||||||  | ||||| |||||||| ||
Sbjct  4838632  AGAAAATTGGTGGAGGGAAGCGGACTACCGGACCACATTCGTGCTTTCCTACGCGGATCC  4838573

Query  1529     TGCCGCAAACAGCATCaaaaaaaTCCTGAAAGATCAAGTGAAGAAGATAGCGAAAATTCC  1588
                ||| |||||    || || || || || || || ||||||||||||||||  | |||||
Sbjct  4838572  TGCGGCAAATCAAATAAAGAAGATACTCAAGGAACAAGTGAAGAAGATAGCTCAGATTCC  4838513

Query  1589     GGAGAGGAA  1597
                |||  ||||
Sbjct  4838512  GGACCGGAA  4838504

Range 2: 4839879 to 4840059
Identities     Gaps           Strand
157/181(87%)   8/181(4%)      Plus/Plus

Query  4911     GAACTACACGATTAAAATAAAAAGAACCCATCCCTCCCACGGGCCCTCCGGCGTGCGCTC  4970
                ||| | ||||||||||| ||||| |||||||||||||| ||||||||| | |||||||||
Sbjct  4839879  GAATTTCACGATTAAAAGAAAAAAAACCCATCCCTCCCGCGGGCCCTCGGACGTGCGCTC  4839938

Query  4971     GCACGGGCGGTCggggaggggac---gggggaggggga-----aggggggTTTTCCTC  5022
                |||||||||||||||| |||  |   ||||||||||||      ||||||||||||||||
Sbjct  4839939  GCACGGGCGGTCGGGGCGGGTGCTTTGGGGGAGGGGGGGGGGGTAGGGGGGGTTTTCCTC  4839998

Query  5023     CGTTTTATATGATCCTTATCGACGTAACGACCCTAAAATCATGATCTACGATCGATTTTA  5082
                  |||||| | |||||||||||||||||||||||||||||||||||| ||||||||||||
Sbjct  4839999  GTTTTTATCTAATCCTTATCGACGTAACGACCCTAAAATCATGATCTTCGATCGATTTTA  4840058

Query  5083     T  5083
                |
Sbjct  4840059  T  4840059

Range 3: 33884290 to 33884938
Identities     Gaps           Strand
424/660(64%)   22/660(3%)     Plus/Plus

Query  1800      TATTTCAGTACCAGTTAAATATTCCTTTCTCAAAATTGTTTTATAATTGCGATAATCCTG  1859
                 ||||||| |  ||| | ||||||||| || |||||| | |||||| |||    || |||||
Sbjct  33884290  TATTTCAATTGCAGCTTAATATTCCATTTTCAAAACTATTTTATGATTCATATGATCCTT  33884349

Query  1860      AGGATTATTTTTTGAATTTATCTGCTAGCATCCTTTGCATTGAAACTTTATTGAAACACC  1919
                       | |||||  ||||   | | |||||  |     | |||| |||||| |  || |
Sbjct  33884350  CCTCCCAATTTTTAGATTTGATTTCCAGCATAATAATATTAGAAAGTTTATTAATCCATC  33884409

Query  1920      AGGTTGGCAGTTTCGGATTCGTAA---ATTTTCTCGAAAATTT--GTTAAATATTCTTGA  1974
                 | ||||| ||   ||     || |   ||||| |  | |||||  |||  || ||    ||
Sbjct  33884410  AAGTTGGTAG---CGATAATGTTATGGATTTTTTATATAATTTAAGTTGCATTTTA--GA  33884464

Query  1975      TAAGCGAATTCCAAAAGTTAATACTTTGTTAATTGCTGGTCCTGCTAATTCAGGAAAAAA  2034
                 ||||  |||||| ||    ||||| || || |||       ||  ||||| | || |||||
Sbjct  33884465  TAAGAAAATTCCCAAGAAAAATACATTATTTATTTATCGCCGCCTAATGCTGGTAAAAA  33884524

Query  2035      ttttttttttGATGCAGTGACACAAGCTATGATAAATTGTGGTCACGTCGGAAATTTTAA  2094
                 |||||||||||||   ||  |||| || ||||| |||    || ||| | |||||||||||
Sbjct  33884525  TTTTTTTTTTGATTGCGTAGCACAGGCAATGATTAATGCAGGCCACATAGGAAATTTTAA  33884584

Query  2095      CAGATTTTGTAACTTTCCATTACAAGAGTGCAACA-TGAAAAGGATCTTATTATGGAATG  2153
                 ||| ||||      |||||  ||    ||| |||  |    || || || ||| |||||||||
Sbjct  33884585  CAGGTTTTCATCATTTCCGCTAATGGAG-GCAGTAGACAAGAGAATTTTAATATGGAATG  33884643

Query  2154      AACCGGTTTGCGAACCATCCGCGGTGGAAACTATAAAAATGCTTTTCGCTGGGGACCCTT  2213
                 |||||  |    || || || ||   | |||   | ||||||    | || |  || || ||||
Sbjct  33884644  AACCGAATGCAGAGCCTTCAGCCTTTGAAGATTTAAAAAAATTATTTGGAGGTGATCCTT  33884703

Query  2214      GCCCCGCAAAAATTAAATACAAAAATGATGCGACAATCATGCGCACTCCCGTAATAGTTC  2273
                 |   |    ||| ||||||  ||    ||||    | | | ||| | ||||| || || |||
Sbjct  33884704  GTAACTGTAAAGTTAAATTTAAGGGTGATAACATAGTTATGAGAACTCCAGTTATTGTTT  33884763

Query  2274      TAACGAACAACAATCCTTTCCCTAATGACCCGCCA----TTTAATAG-TAGAATGATTAA  2328
                 || | ||  |  ||  ||| ||      | |||  |     ||| | ||   |||||||| ||
Sbjct  33884764  TATCAAATGAAGATATTTTTCC-----ATCCGAAAAAGCTTTCAGAGCAAGAATGATAAA  33884818

Query  2329      ATACACATGGCGACAGTCACCTTTCTTGGCAGAAATTCATAAAAAACCTCACCCTTTAGC  2388
                 |   | ||||  |     | ||||| ||   ||| ||| | || ||||| || || |||||
Sbjct  33884819  AGTAAAATGGAAAGCTGCTCCTTTTTTAAAAGATATTAAAAAGAAACCGCATCCATTAGC  33884878

Query  2389      ACTTTTTTATGTTTTGGCAAAGTATAACCTTATTGATACGTTGGAATTGGATAATTTCGA  2448
                   |||||||| | |||||||| || ||| |  | ||||| |  || || || ||||| ||
Sbjct  33884879  TTTTTTTTATTTATTGGCAAAATACAACATCTTAGATACATATGATTTAGAAAATTTTGA  33884938

Range 4: 4839993 to 4840058
Identities     Gaps           Strand
56/70(80%)     6/70(8%)       Plus/Plus

Query  1        TTCCTCCGTTTTATATGATCCTTATCATGACGTAACGACTATAAAATC--GATCGTAGAT  58
                ||||||  |||||| | |||||||||  |||||||||||  |||||||  |||| | |||
Sbjct  4839993  TTCCTCGTTTTTATCTAATCCTTATC--GACGTAACGACCCTAAAATCATGATCTTCGAT  4840050

Query  59       CATGATTTTA  68
                |  |||||||
Sbjct  4840051  C--GATTTTA  4840058


Range 5: 4839993 to 4840058
Identities     Gaps           Strand
56/70(80%)     6/70(8%)       Plus/Minus

Query  5056     TAAAATCATGATCTACGATC--GATTTTATAGTCGTTACGTCATGATAAGGATCATATAA  5113
                |||||||  |||| | ||||  |||||||  |||||||||||  ||||||||| | ||||
Sbjct  4840058  TAAAATC--GATCGAAGATCATGATTTTAGGGTCGTTACGTC--GATAAGGATTAGATAA  4840003

Query  5114     AACGGAGGAA  5123
                ||  ||||||
Sbjct  4840002  AAACGAGGAA  4839993
```

The genomic locus containing the BtaDV-related EVE, nearly identical to the locus of B. tabaci Q-type Almeria chromosome 10

EVE begins with the incomplete flip-flop palindrome. SNPs and the sequence that differs from that of Q-type Almeria are highlighted in yellow

```
>VMOE01000001.1:4836504-4840656 Bemisia tabaci isolate Med HIC_SCAFFOLD_10_PILON_PILON_PILON, whole genome shotgun sequence_REVERSE COMPLEMENT
ATATTTTATTGAGAGCTGCTTAGATTAAAGCAATTATGGAAAATTCTACCTCTTCTGCTGGGGTTATTGCCAAAAATTTCAAGAAAGCTGTAAATGCTAGGTTTCCTGATTACAAAACTGCTGTGGTACGTGCACTTTACATGTTATTGGATCCAAAATTCAAAGCATCTTCACTGCCTGAAACCCTC
AAAAACTTGGGTTGGAAGGCTGAAGACAAAAGCCACAGCGAAATTTGTAGACACGAATGAAGAATATTGGAAAAGTGTAAAGTTTCCAGTCTCAATAATTGCTAGGAAGGCAGAGGGCGCAAGCTTGAAAGATAAGGATAAAAAAGAAAAGAAAACATTCAATGCAATTTGCCAAGGTCAGTATGCAA
TGCATCGATTGCATATTTCTTTTGTCCTCAACTTAGTTGGTTCAAGGTCGATGTACCAGCCAGAATGAAAGCCTTTGACCACCAGTGATGAGTGAAAGACTAATAAATATTTTCATTCTTTCATAGGAACGTGCCTAAAGTTTTTTTTTTTTTTTTTTTTGAGTTTTTTTTGGCTTCTATTCCTTTAT
ATAGATAAGATAGGAATCTACAGCCATCTATAGATAAAATCGATCGAAGATCATGATTTTAGGGTCGTTACGTCGATAAGGATTAGATAAAAACGAGGAAAACCCCCCTACCCCCCCCCCCTCCCCCAAAGCACCCGCCCCGACCGCCCGTGCGAGCGCACGTCCGAGGGCCCGCGGGAGGGATGGG
TTTTTTTTCTTTTAATCGTGAAATTCTTCTGGTGGAGCGATTGTGTAATAAGCGGCAACAATGTATTGTAATCATTGCGTGCGTACGAGGGAATGACGCGGTTTTTTCCGCCGGCGGGAATGAAGCACGGGAAGGACGATAATTTTCAATTGTCATTATATCTCTGATAAAAAGGTAAACAAGGTGTG
TAAATAAAACCCGCGTGAGTGAGGGAACGATCGCAGTATTCGTTTTTTCGTGATGTCAGTTCACCTATCCGACGATTCTAGTTCTCAAAATTCTTTTTCGATTATTGATACTGATTCAAGTGATCTTGTGCAAGTGGAACAGTTATCCCCGTCGGAGGAAAGATACGTGGAACATTGTATTCAAACTA
ATCAACCAGTCTATTTAGTTCGCGACGGTGTAACTAAGTGTATTCCCATTGTTTGTGCGGAAGATGTCTTCCAAAAAGGGAAAGAAAGTACGTCAGGCGTCGCCGGAAACGGAGATGCTTTCCAGTTTGTCTCAAGCCAACAAAACGAAGATAGCCCCTTCCTCGGAGAAGGAACATCCGGAACCAGT
AGCTGGAGCGTCCACGGAACTGACGGAAGCGCAGAAGAAGGTAATACAAGATTTCATAGATCAGTCACCCCTACTGTCAGCCATTCAGGACTTCACGATGGATCATCCTCTAGGGCTTCTTCCCCTGCCTCGTCTAATGTCGGAATTCGTGGGAATAACAGAAAGCGACGGATTGACCAATATTTCAG
CGGATCAGAAGGCTCTGTGGCGGGAGGCACAGATTGCTATGAAGAGAGTTTTGAAGCCATATCTTTCCAGAAATTTAAAGACCTATTCTGTTCAGAATTTAGCAGAATGGATCATAACAGACGGTACATGCTCCACGATATTTACAGAAATACGCTTTCCGGCCGCTTTCGGGAAGATGATCGGGGAA
TCATACCCAAGAGTACAGGGAACTCGTCGGCGTCGTGGGCGTTCGTCGTCTTCGAGCACGGGAAGCACATCCACGTCATCCACCTGTGCAGATATGAGCGATCATGGTGTTCATGCGCGCTCACAAAAAAAGCGGAGAAACGGTTCGGACGAAGAGTTGGAAGATATTCTCTTCGAAGCAGTCGGGTC
TCAAAAAGCCACCTCAGCAATCTCGCAGACTATATACAAAAGGGGAAAAGACGGTTGCTTCAAGCTTTTCTCGCCAGGAGAGAAAGGAGAATACTTGGTGAAGCTGGACTTCTACAAAACAAGGAACCTAAGAAGAGTTGCGAAAGAAAATTGGTGGAGGGAAGCGGACTACCGGACCACATTCGTGC
TTTCCTACGCGGATCCTGCGGCAAATCAAATAAAGAAGATACTCAAGGAACAAGTGAAGAAGATAGCTCAGATTCCGGACCGGAAAGAAAAGAAAAGAAAAGAAAACATTCAATGCAATTTGCCAAGGTCAGTATGCAATGCATCGATTGCATATTTCTTTTGTCCTCAACTTAGTTGGTTCAAGGTC
```

```
GATGTACCAGCCAGAATGAAAGCCTTTGACCACCAGTGATGAGTGAAAGACTAATAAATATTTTCATTCTTTCATAGGAACGTGCCTAAAGTTCATAAATTCAACGTAGAAGATAAAAGAACCGATGCCCCACAAGAAAAAGAGGAGGGACAACCCCCTTTCTGGTCCAGTCTAGATGAATTTCCTAC
ACAAAAATCTGGAGAATAAGACTTGTTGAAAGCAAAACTGATTTGCAATTTGCAAATTACTTAATTGCATAGCTGATGAGAAGCTTAATGCTTTTGAATTTTTGAAAGAAACTGGGAGAAACACCCATAAAACCCTTCGCCTGGTAGCAAAGGAATATTTCGCTAAACAAGGAGTTGCATGTTGAATA
AATCTGCAGCTTCAATGCTCAAAGATAGGAAAGGAATTTTGATGCCTGAAGCTATCTCATGAGACAAAATGCAACATTGCCTGGCGCTGACTTTAATAGTGTAGTAAATAATTATGATGATTATGATATATATAAAAGCATAATAGTAAGTAAGAGATAAGATAAGAGATAAGTTGTTAGATAAGTTC
GCTCTCATAAGCATGAAATTGTCTAGACGGGTGCAAATGATGTAAATAGGAATGAAGCCACATTTTTCTTAAAATCGTTACTAACTTTTCTTAATACTTCAGCCACTCTTGATGCACGGGAAATTTCCAAGAGGCACCAAATTGTAACTCTGCCCTCAAAATTAAAAAAATCAAAATCTGGATTTTGA
CCTTGCAAATCGTGGTCCTGGCAATATTATGCACATGAGATGGCTCACGACGGCCAATCGAGCACTGAGACTGTATGTAGGAACCAAAAATCCCGATTCATCCCTGGTGACCATCGCTACGTACATAATGAAAGTATACGCGCCGAGCTGGTTTGCAATTAAAACCCATTCATCATGCACTGCAGGGG
CCAAACATCTGTTCAAAATAATTGAATTGACTAGAGTAGCACAGAAATCAGAGACATTGTCCACCCTGTCATTCAAAGAAATGCTTTTTCGCCCATCCAGAAAACATCCTGCTTGCTATGCTCAACGATGCATTACAAGATGTTAAACAATTGGCCGTGCAAAAAATCATGAACGCTCGGAGGACTA
AAAGCTCAACAGTACGAAAATTCAAAGTGCCCCTTCTCAATTTCAACGCGGAAACCTACAGTGAAATGATTTTCTGGAACGAAGTCACAGTAACCCAGCCGCCTATCTTACAGTATTTCTCCGAAGAAGATCTAGAAAATTTGGCAAGAGAAAAGGACCTCCAGAATTACAGTTTTACAAATATCCT
TGTCACACGCAAAGTGTCGAAAGATGCGTCAAGATTGTCACCGAGGCATCCGCATTAGTCTGTGGAGGAGAGGAAAGAGATGGGCTGATTCGATCCAAGTTGAAGGCGAGATTGTTGATGCCTGCTTTTGAACCCAAAAGCCAATTTAAATTCGTTAAATGAAGGTGAGCCAAATTATTTGTAGCTAG
CTACAAATGCCCCATACTCTGAGGAAGAATTCATCAACCTATAGACCCTGTTTTGGATTAC
TTTCCTATTTTTATTGTATCTTGTGCCTACTACTATCATGTACCTATCTAGCATGTATAACCTGATCTGTATGATGGCCATGTACACAGCTCATTTACCAATTGTAAAAATAAAATAAAATGAGCATGTACAGAAACAAGTTTTTCTACACGACAACAGACACGTAGTAGTTTGAGAAGCAGTTCTCC
```

```
# 1: 4836504-4840656     MED China
# 2: 37632155-37636914   Q-type Almeria Spain
# Matrix: EDNAFULL
# Gap_penalty: 10.0
# Extend_penalty: 0.5
# Length: 3829
# Identity:    3819/3829 (99.7%)
# Similarity:  3819/3829 (99.7%)
# Gaps:           8/3829 ( 0.2%)

4836504-48406       1 ATATTTTATTGAGAGCTGCTTAGATTAAAGCAATTATGGAAAATTCTACC     50
                      ||||||||||||||||||||||||||||||||||||||||||||||||||
37632155-3763       1 ATATTTTATTGAGAGCTGCTTAGATTAAAGCAATTATGGAAAATTCTACC     50

4836504-48406      51 TCTTCTGCTGGGGTTATTGCCAAAAATTTCAAGAAAGCTGTAAATGCTAG    100
                      ||||||||||||||||||||||||||||||||||||||||||||||||||
37632155-3763      51 TCTTCTGCTGGGGTTATTGCCAAAAATTTCAAGAAAGCTGTAAATGCTAG    100

4836504-48406     101 GTTTCCTGATTACAAAACTGCTGTGGTACGTGCACTTTACATGTTATTGG    150
                      ||||||||||||||||||||||||||||||||||||||||||||||||||
37632155-3763     101 GTTTCCTGATTACAAAACTGCTGTGGTACGTGCACTTTACATGTTATTGG    150

4836504-48406     151 ATCCAAAATTCAAAGCATCTTCACTGCCTGAAACCCTCAAAAACTTGGGT    200
                      ||||||||||||||||||||||||||||||||||||||||||||||||||
37632155-3763     151 ATCCAAAATTCAAAGCATCTTCACTGCCTGAAACCCTCAAAAACTTGGGT    200

4836504-48406     201 TGGAAGGCTGAAGACAAAAGCCACAGCGAAATTTGTAGACACGAATGAAG    250
                      ||||||||||||||||||||||||||||||||||||||||||||||||||
37632155-3763     201 TGGAAGGCTGAAGACAAAAGCCACAGCGAAATTTGTAGACACGAATGAAG    250

4836504-48406     251 AATATTGGAAAAGTGTAAAGTTTCCAGTCTCAATAATTGCTAGGAAGGCA    300
                      ||||||||||||||||||||||||||||||||||||||||||||||||||
37632155-3763     251 AATATTGGAAAAGTGTAAAGTTTCCAGTCTCAATAATTGCTAGGAAGGCA    300

4836504-48406     301 GAGGGCGCAAGCTTGAAAGATAAGGATAAAAAAGAAAAGAAAACATTCAA    350
                      ||||||||||||||||||||||||||||||||||||||||||||||||||
37632155-3763     301 GAGGGCGCAAGCTTGAAAGATAAGGATAAAAAAGAAAAGAAAACATTCAA    350

4836504-48406     351 TGCAATTTGCCAAGGTCAGTATGCAATGCATCGATTGCATATTTCTTTTG    400
                      ||||||||||||||||||||||||||||||||||||||||||||||||||
37632155-3763     351 TGCAATTTGCCAAGGTCAGTATGCAATGCATCGATTGCATATTTCTTTTG    400

4836504-48406     401 TCCTCAACTTAGTTGGTTCAAGGTCGATGTACCAGCCAGAATGAAAGCCT    450
                      ||||||||||||||||||||||||||||||||||||||||||||||||||
37632155-3763     401 TCCTCAACTTAGTTGGTTCAAGGTCGATGTACCAGCCAGAATGAAAGCCT    450

4836504-48406     451 TTGACCACCAGTGATGAGTGAAAGACTAATAAATATTTTCATTCTTTCAT    500
                      ||||||||||||||||||||||||||||||||||||||||||||||||||
37632155-3763     451 TTGACCACCAGTGATGAGTGAAAGACTAATAAATATTTTCATTCTTTCAT    500

4836504-48406     501 AGGAACGTGCCTAAAG----TTTTTTTTTTTTTTTTTTTTGAG-TTTTTT    545
                      ||||||||||||||||    ||||||||||||||||||||||| ||||||
37632155-3763     501 AGGAACGTGCCTAAAGTTTTTTTTTTTTTTTTTTTTTTTTGAGTTTTTTT    550

4836504-48406     546 TTGGCTTCTATTCCTTTATATAGATAAGATAGGAATCTACAGCCATCTAT    595
                      ||||||||||||||||||||||||||||||||||||||||||||||||||
37632155-3763     551 TTGGCTTCTATTCCTTTATATAGATAAGATAGGAATCTACAGCCATCTAT    600

4836504-48406     596 AGATAAAATCGATCGAAGATCATGATTTTAGGGTCGTTACGTCGATAAGG    645
                      ||||||||||||||||||||||||||||||||||||||||||||||||||
37632155-3763     601 AGATAAAATCGATCGAAGATCATGATTTTAGGGTCGTTACGTCGATAAGG    650 the viral sequence begins with a partial flip-flop palidrome

4836504-48406     646 ATTAGATAAAAACGAGGAAAACCCCCCCTACCCCCC---CCCCCTCCCCC    692
                      ||||||||||||||||||||||||||||||||||||   |||||||||||
37632155-3763     651 ATTAGATAAAAACGAGGAAAACCCCCCCTACCCCCCCCTCCCCCTCCCCC    700  TATA-like promoter

4836504-48406     693 AAAGCACCCGCCCCGACCGCCCGTGCGAGCGCACGTCCGAGGGCCCGCGG    742
                      ||||||||||||||||||||||||||||||||||||||||||||||||||
37632155-3763     701 AAAGCACCCGCCCCGACCGCCCGTGCGAGCGCACGTCCGAGGGCCCGCGG    750

4836504-48406     743 GAGGGATGGGTTTTTTTTCTTTTAATCGTGAAATTCTTCTGGTGGAGCGA    792
                      ||||||||||||||||||||||||||||||||||||||||||||||||||
37632155-3763     751 GAGGGATGGGTTTTTTTTCTTTTAATCGTGAAATTCTTCTGGTGGAGCGA    800

4836504-48406     793 TTGTGTAATAAGCGGCAACAATGTATTGTAATCATTGCGTGCGTACGAGG    842
                      ||||||||||||||||||||||||||||||||||||||||||||||||||
37632155-3763     801 TTGTGTAATAAGCGGCAACAATGTATTGTAATCATTGCGTGCGTACGAGG    850

4836504-48406     843 GAATGACGCGGTTTTTTCCGCCGGCGGGAATGAAGCACGGGAAGGACGAT    892
                      ||||||||||||||||||||||||||||||||||||||||||||||||||
37632155-3763     851 GAATGACGCGGTTTTTTCCGCCGGCGGGAATGAAGCACGGGAAGGACGAT    900

4836504-48406     893 AATTTTCAATTGTCATTATATCTCTGATAAAAAGGTAAACAAGGTGTGTA    942
                      ||||||||||||||||||||||||||||||||||||||||||||||||||
37632155-3763     901 AATTTTCAATTGTCATTATATCTCTGATAAAAAGGTAAACAAGGTGTGTA    950

4836504-48406     943 AATAAAACCCGCGTGAGTGAGGGAACGATCGCAGTATTCGTTTTTTCGTG    992
                      ||||||||||||||||||||||||||||||||||||||||||||||||||
37632155-3763     951 AATAAAACCCGCGTGAGTGAGGGAACGATCGCAGTATTCGTTTTTTCGTG   1000

4836504-48406     993 ATGTCAGTTCACCTATCCGACGATTCTAGTTCTCAAAATTCTTTTTCGAT   1042
                      ||||||||||||||||||||||||||||||||||||||||||||||||||
37632155-3763    1001 ATGTCAGTTCACCTATCCGACGATTCTAGTTCTCAAAATTCTTTTTCGAT   1050

4836504-48406    1043 TATTGATACTGATTCAAGTGATCTTGTGCAAGTGGAACAGTTATCCCCGT   1092
                      ||||||||||||||||||||||||||||||||||||||||||||||||||
37632155-3763    1051 TATTGATACTGATTCAAGTGATCTTGTGCAAGTGGAACAGTTATCCCCGT   1100

4836504-48406    1093 CGGAGGAAAGATACGTGGAACATTGTATTCAAACTAATCAACCAGTCTAT   1142
                      ||||||||||||||||||||||||||||||||||||||||||||||||||
37632155-3763    1101 CGGAGGAAAGATACGTGGAACATTGTATTCAAACTAATCAACCAGTCTAT   1150

4836504-48406    1143 TTAGTTCGCGACGGTGTAACTAAGTGTATTCCCATTGTTTGTGCGGAAGA   1192
                      ||||||||||||||||||||||||||||||||||||||||||||||||||
37632155-3763    1151 TTAGTTCGCGACGGTGTAACTAAGTGTATTCCCATTGTTTGTGCGGAAGA   1200

4836504-48406    1193 TGTCTTCCAAAAAGGGAAAGAAAGTACGTCAGGCGTCGCCGGAAACGGAG   1242
                      ||||||||||||||||||||||||||||||||||||||||||||||||||
37632155-3763    1201 TGTCTTCCAAAAAGGGAAAGAAAGTACGTCAGGCGTCGCCGGAAACGGAG   1250

4836504-48406    1243 ATGCTTTCCAGTTTGTCTCAAGCCAACAAAACGAAGATAGCCCCTTCCTC   1292
                      ||||||||||||||||||||||||||||||||||||||||||||||||||
37632155-3763    1251 ATGCTTTCCAGTTTGTCTCAAGCCAACAAAACGAAGATAGCCCCTTCCTC   1300

4836504-48406    1293 GGAGAAGGAACATCCGGAACCAGTAGCTGGAGCGTCCACGGAACTGACGG   1342
                      ||||||||||||||||||||||||||||||||||||||||||||||||||
37632155-3763    1301 GGAGAAGGAACATCCGGAACCAGTAGCTGGAGCGTCCACGGAACTGACGG   1350

4836504-48406    1343 AAGCGCAGAAGAAGGTAATACAAGATTTCATAGATCAGTCACCCCTACTG   1392
                      ||||||||||||||||||||||||||||||||||||||||||||||||||
37632155-3763    1351 AAGCGCAGAAGAAGGTAATACAAGATTTCATAGATCAGTCACCCCTACTG   1400

4836504-48406    1393 TCAGCCATTCAGGACTTCACGATGGATCATCCTCTAGGGCTTCTTCCCCT   1442
                      ||||||||||||||||||||||||||||||||||||||||||||||||||
37632155-3763    1401 TCAGCCATTCAGGACTTCACGATGGATCATCCTCTAGGGCTTCTTCCCCT   1450

4836504-48406    1443 GCCTCGTCTAATGTCGGAATTCGTGGGAATAACAGAAAGCGACGGATTGA   1492
                      ||||||||||||||||||||||||||||||||||||||||||||||||||
37632155-3763    1451 GCCTCGTCTAATGTCGGAATTCGTGGGAATAACAGAAAGCGACGGATTGA   1500

4836504-48406    1493 CCAATATTTCAGCGGATCAGAAGGCTCTGTGGCGGGAGGCACAGATTGCT   1542
```

```
                          ||||||||||||||||||||||||||||||||||||||||||||||||||
37632155-3763       1501  CCAATATTTCAGCGGATCAGAAGGCTCTGTGGCGGGAGGCACAGATTGCT  1550

4836504-48406       1543  ATGAAGAGAGTTTTGAAGCCATATCTTTCCAGAAATTTAAAGACCTATTC  1592
                          ||||||||||||||||||||||||||||||||||||||||||||||||||
37632155-3763       1551  ATGAAGAGAGTTTTGAAGCCATATCTTTCCAGAAATTTAAAGACCTATTC  1600

4836504-48406       1593  TGTTCAGAATTTAGCAGAATGGATCATAACAGACGGTACATGCTCCACGA  1642
                          ||||||||||||||||||||||||||||||||||||||||||||||||||
37632155-3763       1601  TGTTCAGAATTTAGCAGAATGGATCATAACAGACGGTACATGCTCCACGA  1650

4836504-48406       1643  TATTTACAGAAATACGCTTTCCGGCCGCTTTCGGGAAGATGATCGGGGAA  1692
                          ||||||||||||||||||||||||||||||||||||||||||||||||||
37632155-3763       1651  TATTTACAGAAATACGCTTTCCGGCCGCTTTCGGGAAGATGATCGGGGAA  1700

4836504-48406       1693  TCATACCCAAGAGTACAGGGAACTCGTCGGCGTCGTGGGCGTTCGTCGTC  1742
                          ||||||||||||||||||||||||||||||||||||||||||||||||||
37632155-3763       1701  TCATACCCAAGAGTACAGGGAACTCGTCGGCGTCGTGGGCGTTCGTCGTC  1750

4836504-48406       1743  TTCGAGCACGGGAAGCACATCCACGTCATCCACCTGTGCAGATATGAGCG  1792
                          ||||||||||||||||||||||||||||||||||||||||||||||||||
37632155-3763       1751  TTCGAGCACGGGAAGCACATCCACGTCATCCACCTGTGCAGATATGAGCG  1800

4836504-48406       1793  ATCATGGTGTTCATGCGCGCTCACAAAAAAAGCGGAGAAACGGTTCGGAC  1842
                          ||||||||||||||||||||||||||||||||||||||||||||||||||
37632155-3763       1801  ATCATGGTGTTCATGCGCGCTCACAAAAAAAGCGGAGAAACGGTTCGGAC  1850

4836504-48406       1843  GAAGAGTTGGAAGATATTCTCTTCGAAGCAGTCGGGTCTCAAAAAGCCAC  1892
                          ||||||||||||||||||||||||||||||||||||||||||||||||||
37632155-3763       1851  GAAGAGTTGGAAGATATTCTCTTCGAAGCAGTCGGGTCTCAAAAAGCCAC  1900

4836504-48406       1893  CTCAGCAATCTCGCAGACTATATACAAAAGGGGAAAAGACGGTTGCTTCA  1942
                          ||||||||||||||||||||||||||||||||||||||||||||||||||
37632155-3763       1901  CTCAGCAATCTCGCAGACTATATACAAAAGGGGAAAAGACGGTTGCTTCA  1950

4836504-48406       1943  AGCTTTTCTCGCCAGGAGAGAAAGGAGAATACTTGGTGAAGCTGGACTTC  1992
                          ||||||||||||||||||||||||||||||||||||||||||||||||||
37632155-3763       1951  AGCTTTTCTCGCCAGGAGAGAAAGGAGAATACTTGGTGAAGCTGGACTTC  2000

4836504-48406       1993  TACAAAACAAGGAACCTAAGAAGAGTTGCGAAAGAAAATTGGTGGAGGGA  2042
                          ||||||||||||||||||||||||||||||||||||||||||||||||||
37632155-3763       2001  TACAAAACAAGGAACCTAAGAAGAGTTGCGAAAGAAAATTGGTGGAGGGA  2050

4836504-48406       2043  AGCGGACTACCGGACCACATTCGTGCTTTCCTACGCGGATCCTGCGGCAA  2092
                          ||||||||||||||||||||||||||||||||||||||||||||||||||
37632155-3763       2051  AGCGGACTACCGGACCACATTCGTGCTTTCCTACGCGGATCCTGCGGCAA  2100

4836504-48406       2093  ATCAAATAAAGAAGATACTCAAGGAACAAGTGAAGAAGATAGCTCAGATT  2142
                          ||||||||||||||||||||||||||||||||||||||||||||||||||
37632155-3763       2101  ATCAAATAAAGAAGATACTCAAGGAACAAGTGAAGAAGATAGCTCAGATT  2150

4836504-48406       2143  CCGGACCGGAAAGAAAAGAAAAGAAAAGAAAACATTCAATGCAATTTGCC  2192
                          ||||||||||||||||||||||||||||||||||||||||||||||||||
37632155-3763       2151  CCGGACCGGAAAGAAAAGAAAAGAAAAGAAAACATTCAATGCAATTTGCC  2200

4836504-48406       2193  AAGGTCAGTATGCAATGCATCGATTGCATATTTCTTTTGTCCTCAACTTA  2242
                          ||||||||||||||||||||||||||||||||||||||||||||||||||
37632155-3763       2201  AAGGTCAGTATGCAATGCATCGATTGCATATTTCTTTTGTCCTCAACTTA  2250

4836504-48406       2243  GTTGGTTCAAGGTCGATGTACCAGCCAGAATGAAAGCCTTTGACCACCAG  2292
                          ||||||||||||||||||||||||||||||||||||||||||||||||||
37632155-3763       2251  GTTGGTTCAAGGTCGATGTACCAGCCAGAATGAAAGCCTTTGACCACCAG  2300

4836504-48406       2293  TGATGAGTGAAAGACTAATAAATATTTTCATTCTTTCATAGGAACGTGCC  2342
                          ||||||||||||||||||||||||||||||||||||||||||||||||||
37632155-3763       2301  TGATGAGTGAAAGACTAATAAATATTTTCATTCTTTCATAGGAACGTGCC  2350

4836504-48406       2343  TAAAGTTCATAAATTCAACGTAGAAGATAAAAGAACCGATGCCCCACAAG  2392
                          |||||||||||||||||||||||||||||||||||||||||||||||.||
37632155-3763       2351  TAAAGTTCATAAATTCAACGTAGAAGATAAAAGAACCGATGCCCCACGAG  2400

4836504-48406       2393  AAAAAGAGGAGGGACAACCCCCTTTCTGGTCCAGTCTAGATGAATTTCCT  2442
                          ||||||||||||||||||||||||||||||||||||||||||||||||||
37632155-3763       2401  AAAAAGAGGAGGGACAACCCCCTTTCTGGTCCAGTCTAGATGAATTTCCT  2450

4836504-48406       2443  ACACAAAAATCTGGAGAATAAGACTTGTTGAAAGCAAAACTGATTTGCAA  2492
                          ||||||||||||||||||||||||||||||||||||||||||||||||||
37632155-3763       2451  ACACAAAAATCTGGAGAATAAGACTTGTTGAAAGCAAAACTGATTTGCAA  2500

4836504-48406       2493  TTTGCAAATTACTTAATTGCATAGCTGATGAGAAGCTTAATGCTTTTGAA  2542
                          ||||||||||||||||||||||||||||||||||||||||||||||||||
37632155-3763       2501  TTTGCAAATTACTTAATTGCATAGCTGATGAGAAGCTTAATGCTTTTGAA  2550

4836504-48406       2543  TTTTTGAAAGAAACTGGGAGAAACACCCATAAAACCCTTCGCCTGGTAGC  2592
                          ||||||||||||||||||||||||||||||||||||||||||||||||||
37632155-3763       2551  TTTTTGAAAGAAACTGGGAGAAACACCCATAAAACCCTTCGCCTGGTAGC  2600

4836504-48406       2593  AAAGGAATATTTCGCTAAACAAGGAGTTGCATGTTGAATAAATCTGCAGC  2642
                          ||||||||||||||||||||||||||||||||||||||||||||||||||
37632155-3763       2601  AAAGGAATATTTCGCTAAACAAGGAGTTGCATGTTGAATAAATCTGCAGC  2650

4836504-48406       2643  TTCAATGCTCAAAGATAGGAAAGGAATTTTGATGCCTGAAGCTATCTCAT  2692
                          ||||||||||||||||||||||||||||||||||||||||||||||||||
37632155-3763       2651  TTCAATGCTCAAAGATAGGAAAGGAATTTTGATGCCTGAAGCTATCTCAT  2700

4836504-48406       2693  GAGACAAAATGCAACATTGCCTGGCGCTGACTTTAATAGTGTAGTAAATA  2742
                          ||||||||||||||||||||||||||||||||||||||||||||||||||
37632155-3763       2701  GAGACAAAATGCAACATTGCCTGGCGCTGACTTTAATAGTGTAGTAAATA  2750

4836504-48406       2743  ATTATGATGATTATGATATATATAAAAGCATAATAGTAAGTAAGAGATAA  2792
                          ||||||||||||||||||||||||||||||||||||||||||||||||||
37632155-3763       2751  ATTATGATGATTATGATATATATAAAAGCATAATAGTAAGTAAGAGATAA  2800

4836504-48406       2793  GATAAGAGATAAGTTGTTAGATAAGTTCGCTCTCATAAGCATGAAATTGT  2842
                          ||||||||||||||||||||||||||||||||||||||||||||||||||
37632155-3763       2801  GATAAGAGATAAGTTGTTAGATAAGTTCGCTCTCATAAGCATGAAATTGT  2850

4836504-48406       2843  CTAGACGGGTGCAAATGATGTAAATAGGAATGAAGCCACATTTTTCTTAA  2892
                          ||||||||||||||||||||||||||||||||||||||||||||||||||
37632155-3763       2851  CTAGACGGGTGCAAATGATGTAAATAGGAATGAAGCCACATTTTTCTTAA  2900

4836504-48406       2893  AATCGTTACTAACTTTTCTTAATACTTCAGCCACTCTTGATGCACGGGAA  2942
                          ||||||||||||||||||||||||||||||||||||||||||||||||||
37632155-3763       2901  AATCGTTACTAACTTTTCTTAATACTTCAGCCACTCTTGATGCACGGGAA  2950

4836504-48406       2943  ATTTCCAAGAGGCACCAAATTGTAACTCTGCCCTCAAAATTAAAAAAATC  2992
                          ||||||||||||||||||||||||||||||||||||||||||||||||||
37632155-3763       2951  ATTTCCAAGAGGCACCAAATTGTAACTCTGCCCTCAAAATTAAAAAAATC  3000

4836504-48406       2993  AAAATCTGGATTTTGACCTTGCAAATCGTGGTCCTGGCAATATTATGCAC  3042
                          ||||||||||||||||||||||||||||||||||||||||||||||||||
37632155-3763       3001  AAAATCTGGATTTTGACCTTGCAAATCGTGGTCCTGGCAATATTATGCAC  3050

4836504-48406       3043  ATGAGATGGCTCACGACGGCCAATCGAGCACTGAGACTGTATGTAGGAAC  3092
                          ||||||||||||||||||||||||||||||||||||||||||||||||||
37632155-3763       3051  ATGAGATGGCTCACGACGGCCAATCGAGCACTGAGACTGTATGTAGGAAC  3100

4836504-48406       3093  CAAAAATCCCGATTCATCCCTGGTGACCATCGCTACGTACATAATGAAAG  3142
                          ||||||||||||||||||||||||||||||||||||||||||||||||||
37632155-3763       3101  CAAAAATCCCGATTCATCCCTGGTGACCATCGCTACGTACATAATGAAAG  3150

4836504-48406       3143  TATACGCGCCGAGCTGGTTTGCAATTAAAACCCATTCATCATGCACTGCA  3192
                          ||||||||||||||||||||||||||||||||||||||||||||||||||
37632155-3763       3151  TATACGCGCCGAGCTGGTTTGCAATTAAAACCCATTCATCATGCACTGCA  3200

4836504-48406       3193  GGGGCCAAACATCTGTTCAAAATAATTGAATTGACTAGAGTAGCACAGAA  3242
                          ||||||||||||||||||||||||||||||||||||||||||||||||||
37632155-3763       3201  GGGGCCAAACATCTGTTCAAAATAATTGAATTGACTAGAGTAGCACAGAA  3250

4836504-48406       3243  ATCAGAGACATTGTCCACCCTGTCATTCAAAGAAATGCTTTTTTCGCCCA  3292
                          ||||||||||||||||||||||||||||||||||||||||||||||||||
37632155-3763       3251  ATCAGAGACATTGTCCACCCTGTCATTCAAAGAAATGCTTTTTTCGCCCA  3300
```

```
4836504-48406   3293 TCCAGAAAACATCCTGCTTGCTATGCTCAACGATGCATTACAAGATGTTA   3342
                     ||||||||||||||||||||||||||||||||||||||||||||||||||
37632155-3763   3301 TCCAGAAAACATCCTGCTTGCTATGCTCAACGATGCATTACAAGATGTTA   3350

4836504-48406   3343 AACAATTGGCCGTGCAAAAAATCATGAACGCTCGGAGGACTAAAAGCTCA   3392
                     ||||||||||||||||||||||||||||||||||||||||||||||||||
37632155-3763   3351 AACAATTGGCCGTGCAAAAAATCATGAACGCTCGGAGGACTAAAAGCTCA   3400

4836504-48406   3393 ACAGTACGAAAATTCAAAGTGCCCCTTCTCAATTTCAACGCGGAAACCTA   3442
                     ||||||||||||||||||||||||||||||||||||||||||||||||||
37632155-3763   3401 ACAGTACGAAAATTCAAAGTGCCCCTTCTCAATTTCAACGCGGAAACCTA   3450

4836504-48406   3443 CAGTGAAATGATTTTCTGGAACGAAGTCACAGTAACCCAGCCGCCTATCT   3492
                     ||||||||||||||||||||||||||||||||||||||||||||||||||
37632155-3763   3451 CAGTGAAATGATTTTCTGGAACGAAGTCACAGTAACCCAGCCGCCTATCT   3500

4836504-48406   3493 TACAGTATTTCTCCGAAGAAGATCTAGAAAATTTGGCAAGAGAAAAGGAC   3542
                     ||||||||||||||||||||||||||||||||||||||||||||||||||
37632155-3763   3501 TACAGTATTTCTCCGAAGAAGATCTAGAAAATTTGGCAAGAGAAAAGGAC   3550

4836504-48406   3543 CTCCAGAATTACAGTTTTTACAAATATCCTTGTCACACGCAAAGTGTCGA   3592
                     ||||||||||||||||||||||||||||||||||||||||||||||||||
37632155-3763   3551 CTCCAGAATTACAGTTTTTACAAATATCCTTGTCACACGCAAAGTGTCGA   3600

4836504-48406   3593 AAGATGCGTCAAGATTGTCACCGAGGCATCCGCATTAGTCTGTGGAGGAG   3642
                     ||||||||||||||||||||||||||||||||||||||||||||||||||
37632155-3763   3601 AAGATGCGTCAAGATTGTCACCGAGGCATCCGCATTAGTCTGTGGAGGAG   3650

4836504-48406   3643 AGGAAAGAGATGGGCTGATTCGATCCAAGTTGAAGGCGAGATTGTTGATG   3692
                     ||||||||||||||||||||||||||||||||||||||||||||||||||
37632155-3763   3651 AGGAAAGAGATGGGCTGATTCGATCCAAGTTGAAGGCGAGATTGTTGATG   3700

4836504-48406   3693 CCTGCTTTTGAACCCAAAAGCCAATTTAAATTCGTTAAATGAAGGTGAGC   3742
                     ||||||||||||||||||||||||||||||||||||||||||||||||||
37632155-3763   3701 CCTGCTTTTGAACCCAAAAGCCAATTTAAATTCGTTAAATGAAGGTGAGC   3750

4836504-48406   3743 CAAATTATTTGTAGCTAGCTACAAATGCCCCATACTCTGAGGAAGAATTC   3792
                     ||||||||||||||||||||||||||||||||||||||||||||||||||
37632155-3763   3751 CAAATTATTTGTAGCTAGCTACAAATGCCCCATACTCTGAGGAAGAATTC   3800

4836504-48406   3793 ATCAACCTATAGACCCTGTTTTGGATTAC   3809
                     |||||||||||||||||||||.|||||||
37632155-3763   3801 ATCAACCTATAGACCCTGTTTCGGATTAC   3821
```

The BtaDV-related EVE encoding the SF3 helicase domain with the internal Parvo_NS1 domain

Located in the same scaffold (VMOE01000001), but far away from the NS1 Rep nickase domain-containing EVE (positions 4836504-4840656)

```
>VMOE01000001.1:33884290-33884938 Bemisia tabaci isolate Med HIC_SCAFFOLD_10_PILON_PILON_PILON, whole genome shotgun sequence
TATTTCAATTGCAGCTTAATATTCCATTTTCAAAACTATTTTATGATTCATATGATCCTTCCTCCCAATTTTTAGATTTGATTTCCAGCATAATAATATTAGAAAGTTTATTAATCCATCAAGTTGGTAGCGATAATGTTATGGATTTTTTATATAATTTAAGTTGCATTTTAGATAAGAAAATTCCC
AAGAAAAATACATTATTTATTTTATCGCCGCCTAATGCTGGTAAAAATTTTTTTTTGATTGCGTAGCACAGGCAATGATTAATGCAGGCCACATAGGAAATTTTAACAGGTTTTCATCATTTCCGCTAATGGAGGCAGTAGACAAGAGAATTTTAATATGGAATGAACCGAATGCAGAGCCTTCAGC
CTTTGAAGATTTAAAAAAATTATTTGGAGGTGATCCTTGTAACTGTAAAGTTAAATTTAAGGGTGATAACATAGTTATGAGAACTCCAGTTATTGTTTTATCAAATGAAGATATTTTTCCATCCGAAAAAGCTTTCAGAGCAAGAATGATAAAAGTAAAATGGAAAGCTGCTCCTTTTTTAAAAGATA
TTAAAAAGAAACCGCATCCATTAGCTTTTTTTTATTTATTGGCAAAATACAACATCTTAGATACATATGATTTAGAAAATTTTGA
```

The 215 amino-acid protein encoded by this EVE is homologuos to the BtaDN NSI SF3 helicase domain:

```
BtaDV_NS1       451 TVQQIFQYQLNIPFSKLFYNCDNPEDYFLNLSASILCIETLLKHQVGSFG    500
                         ||.|||||||||||:..:|...||:|.:||:.:|:||.|||||..
ORF3              1 -----FQLQLNIPFSKLFYDSYDPSSQFLDLISSIIILESLLIHQVGSDN     45

BtaDV_NS1       501 FVNFLENLLNILDKRIPKVNTLLIAGPANSGKNFFFDAVTQAMINCGHVG    550
                    .::||.||..||||:|||.|||.|..|.|:||||||||.|.|||||.||:|
ORF3             46 VMDFLYNLSCILDKKIPKKNTLFILSPPNAGKNFFFDCVAQAMINAGHIG     95

BtaDV_NS1       551 NFNRFCNFPLQECNMKRILLWNEPVCEPSAVETIKMLFAGDPCPAKIKYK    600
                    |||||.:|||.|...||||:||||..||||.|.:|.||.||||..|:|:|
ORF3             96 NFNRFSSFPLMEAVDKRILIWNEPNAEPSAFEDLKKLFGGDPCNCKVKFK    145

BtaDV_NS1       601 NDATIMRTPVIVLTNNNPFPNDPPFNSRMIKYTWRQSPFLAEIHKKPHPL    650
                    .|..:|||||||||:|.:.||::..|.:||||..|:.:|||.:|.||||||
ORF3            146 GDNIVMRTPVIVLSNEDIFPSEKAFRARMIKVKWKAAPFLKDIKKKPHPL    195

BtaDV_NS1       651 ALFYVLAKYNLIDTLELDNFDSSLLQDLSVTTFIQEATHDIDSSIRSTEA    700
                    |.||:||||||::||.:|:||
ORF3            196 AFFYLLAKYNILDTYDLENF------------------------------    215
```

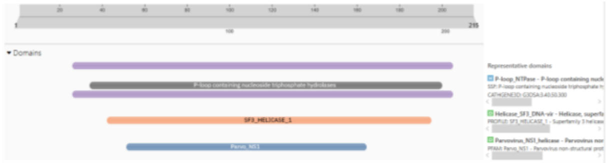

BtaDV-related EVEs in the genome of *B. tabaci* MEAM1 single female from USA (BioProject: PRJNA312470)

Descriptions | Graphic Summary | Alignments | Taxonomy

Sequences producing significant alignments — Download — Select columns — Show 100

select all 8 sequences selected — GenBank — Graphics — Distance tree of results — MSA Viewer

Description	Scientific Name	Max Score	Total Score	Query Cover	E value	Per. Ident	Acc. Len	Accession
Bemisia tabaci isolate MEAM1 unplaced genomic scaffold, ASM185493v1, whole genome shotgun seq...	Bemisia tabaci	1342	1454	35%	0.0	76.37%	8940436	NW_017549038.1
Bemisia tabaci isolate MEAM1 unplaced genomic scaffold, ASM185493v1, whole genome shotgun seq...	Bemisia tabaci	159	378	16%	8e-36	72.03%	2756461	NW_017548293.1
Bemisia tabaci isolate MEAM1 unplaced genomic scaffold, ASM185493v1, whole genome shotgun seq...	Bemisia tabaci	65.3	65.3	2%	1e-07	72.50%	1062	NW_017560287.1
Bemisia tabaci isolate MEAM1 unplaced genomic scaffold, ASM185493v1, whole genome shotgun seq...	Bemisia tabaci	52.7	52.7	2%	8e-04	72.38%	2636620	NW_017547299.1
Bemisia tabaci isolate MEAM1 unplaced genomic scaffold, ASM185493v1, whole genome shotgun seq...	Bemisia tabaci	52.7	52.7	1%	8e-04	79.69%	2790873	NW_017566682.1
Bemisia tabaci isolate MEAM1 unplaced genomic scaffold, ASM185493v1, whole genome shotgun seq...	Bemisia tabaci	50.0	50.0	2%	0.003	75.31%	3617067	NW_017547290.1
Bemisia tabaci isolate MEAM1 unplaced genomic scaffold, ASM185493v1, whole genome shotgun seq...	Bemisia tabaci	50.0	50.0	2%	0.003	75.31%	1080145	NW_017548866.1
Bemisia tabaci isolate MEAM1 unplaced genomic scaffold, ASM185493v1, whole genome shotgun seq...	Bemisia tabaci	47.3	47.3	1%	0.034	84.44%	2348942	NW_017547231.1

Distribution of the top 11 Blast Hits on 8 subject sequences

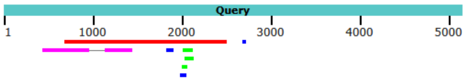

Bemisia tabaci isolate MEAM1 unplaced genomic scaffold, ASM185493v1, whole genome shotgun sequence

```
Sequence ID: NW_017549038.1 Length: 8940436 Number of Matches: 2
Identities      Gaps            Strand
1390/1820(76%)                  12/1820(0%)     Plus/Minus

Query  677      GGAGACGGAAATGCTTTCGAATTTGTCGGAAGTAGACAAACAGAAAAAAGTCCATATCTT  736
                ||| ||||| |||||||| | ||||||  ||| |||||||  ||| | || ||||  ||
Sbjct  8905132  GGAAACGGAGATGCTTTCCAGTTTGTCTCAAGCAGACAAAGCGAAGATAGCCCATTCCTC  8905073

Query  737      GGAGAAGGATCCTCCGGAGCCTCAAGCGGGTCCATCCCTCGAGATGACGGAAACAAGGAA  796
                ||||||||| | |||||| ||   ||| ||  | |||   ||  || ||||| |   |||
Sbjct  8905072  GGAGAAGGAACATCCGGAACCAATAGCTGGAGCGTCCACGGAACTGTCGGAAGCGCAGAA  8905013
```

```
Query  797      GCAGGAAATTCAGGACTTTATCGATCGATCTCCCCTT-TTGACAGCGATACAGGATTACT  855
                | |||  || || || || || |||| ||| |||||  |||||||| |||||||| |||
Sbjct  8905012  GAAGGTGATACAAGATTTCATAGATCAATCACCCCTCCTTGACAGCCATACAGGACTACA  8904953

Query  856      CCATGGAACACCCCCTCGGCCTTCTTCCGTTGCCTCGACTGATGTCCGAGTTCGTAGGCG  915
                | ||||| ||  | || || ||||||||  ||||||| || ||||| || ||||| ||
Sbjct  8904952  CGATGGAGCATACTCTAGGGCTTCTTCCCCTGCCTCGTCTCATGTCGGAATTCGTGGGAA  8904893

Query  916      TAATCGAAGCAAACGGATTGACGAATATTTCTCAGGAGCAGAAAGTTCTTTGGCGGGAAG  975
                |||| |||    |||||||||||||||||||   ||| ||||| | ||| |||||||| |
Sbjct  8904892  TAATAGAAAGCGACGGATTGACGAATATTTCAGCGGATCAGAAGGCTCTGTGGCGGGAGG  8904833

Query  976      CTCAAATAGCTACCAAGAAAGTTTTGAAACCGTTTCTTTCGAAAAATTTAAAAGCTTATT  1035
                | || || ||||  |||| |||||||||||| ||||||||| | |||||||||||  | ||||
Sbjct  8904832  CACAGATTGCTATGAAGAGAGTTTTGAAACCATTTCTTTCCAGAAATTTAAAGACCTATT  8904773

Query  1036     CTGTGCAGAATTTGGCCGACTGGATAATCACCGAAGGTACATATTACACGACATTTACCG  1095
                |||| |||||||||||| || ||||| || || || ||||||||  | ||||| | |||| |
Sbjct  8904772  CTGTTCAGAATTTGGCAGATTGGATCATAACAGACGGTACATGCTCCACGATAATTACAG  8904713

Query  1096     AAATACAATTTCCGGGCGCTTTCGGGAAAATGATAGGGGAGTCGTACCCAAGGACACAGG  1155
                ||||||  ||||||| ||||||||| ||| || || || ||||| || ||||||||| | |||||
Sbjct  8904712  AAATACGCTTTCCGGCCGCTTTCGCGAAGATTATCGGGGAATCATACCCAAGAATACAGG  8904653

Query  1156     CAACTCATCATCGGCATGGACATTCGTCGTCTTCCAGCACGGCCAGCATGTCCACGTCAT  1215
                 ||||| ||  || | ||| |||||||||||||| |||||||  ||||         |||
Sbjct  8904652  GAACTCGTCGGCGTCGTGGGCATTCGTCGTCTTCGAGCACGGGAAGCA---------CAT  8904602

Query  1216     CCACAAGTGCCGATACGAAAGGTCGTGGTGCTCGTGCGCACTCACGAAGAAAGCGGAAGA  1275
                ||||  |||| |||| ||  | || ||||| || ||||| ||||| || ||||||||  |
Sbjct  8904601  CCACCTGTGCAGATATGAGCGATCATGGTGTTCATGCGCGCTCACAAAAAAAGCGGAGAA  8904542

Query  1276     GCGATTCGGACGACGAATTGGAAGACATACTTTACGAGGCAGTAGGATCACAGAAGGCCA  1335
                 || ||||||||| || |||||||| || || | ||| ||||| || || || || ||||
Sbjct  8904541  ACGGTTCGGACGAAGAGTTGGAAGATATTCTCTTCGAAGCAGTCGGGTCTCAAAAAGCCA  8904482

Query  1336     CCTCGAACATCTCGCAAATTATATACAAAAAGGGCGACGCAGGCTCCTTCAAGCTTTCCT  1395
                ||||    |||||||| | ||||||||||| |||  | |  |||| ||||||||||| ||
Sbjct  8904481  CCTCAGCAATCTCGCAGACTATATACAAAAGGGGAAAAGACGGCTGCTTCAAGCTTTTCT  8904422

Query  1396     CGCCGGGCGAGAAAGGCGATTATCTCGTCAAGCTGGACTTTTACAAGACACGCAATCTTC  1455
                |||| || |||||||| || || || || || ||||| || ||||| ||| | || ||
Sbjct  8904421  CGCCAGGAGAGAAAGGAGACTACCTGGTGAAACTGGATTTCTACAAAACAAGGAACCTAA  8904362

Query  1456     GCAGAGTGGCAAAGGAGAACTGGTGGAAGGAGGCCGATTACCGGAGCACATTTCTTCTTT  1515
                | ||||| || || ||||| | ||||| ||| || |||||||||| ||||||  | ||||
Sbjct  8904361  GAAGAGTTGCGAAAGAGAATTCGTGGAGGGAAGCGGATTACCGGACCACATTCGTGCTTT  8904302

Query  1516     CTTACGCGGAACCTGCCGCAAACAGCATCaaaaaaaTCCTGAAAGATCAAGTGAAGAAGA  1575
                | || ||||| ||||| |||||    || || || || || || |||||||||||||
Sbjct  8904301  CCTAAGCGGATCCTGCGGCAAATCAAATAAAGAAGATACTCAAGGAACAAGTGAAGAAGA  8904242

Query  1576     TAGCGAAAATTCCGGAGAGGAAGGTGGAGTACCTGCCCCAACAAAAAGGGAGAAAATAAT  1635
                ||||  | ||||||||  |||| ||||||| ||||||||  |||||||||| ||||||||
Sbjct  8904241  TAGCTCAGATTCCGGACCGGAAAGTGGAGTTCCTGCCCCGTCAAAAAGGGAAAAAATAAT  8904182

Query  1636     TCAATTTCTTCAGTCGTGCGTAATAACTCCTTTTTCTCGAATTTTTCAATCCCAATTGTA  1695
                ||| ||||| || ||||| ||||| || || ||||| || ||||||||||| || || ||
Sbjct  8904181  TCAGTTTCTGCAATCGTGTGTAATTACACCGTTTTCCCGTATTTTTCAATCGCATTTATA  8904122

Query  1696     CTTATTCGATAAATCTTTATATTTTATTACACCTGAATCTATGTCTTGTCAGCTTGCCAT  1755
                  |||| ||||||||||||| ||| || ||||| ||||| |||||||| || || || ||
Sbjct  8904121  TCTATTTGATAAATCTTTATTTTTCATAACACCAGAATCCATGTCTTGCCAACTAGCAAT  8904062

Query  1756     TTTACAGCACACACATACGATTAACAAGATGACTGTGCAACAAATATTTCAGTACCAGTT  1815
                |||||| ||||| || || || || || ||||  || || ||| ||||||| || || ||
Sbjct  8904061  TTTACAACACACTCACACAATAAATAAAATGAGCGTTCAGCAATTATTTCAATATCAATT  8904002

Query  1816     AAATATTCCTTTCTCAAAATTGTTTTATAATTGCGATAATCCTGAGGATTATTTTTTGAA  1875
                |||||| || |  || ||||| ||||| ||||||||||  ||||| ||||||||| | ||
Sbjct  8904001  AAATATACCATATTCCAAATTATTTTACAATTGCGATACGCCTGAAGATTATTTTCTTAA  8903942

Query  1876     TTTATCTGCTAGCATCCTTTGCATTGAAACTTTATTGAAACACCAGGTTGGCAGTTTCGG  1935
                 ||| | || || || ||||| ||||||||||| || || ||||| || || ||||| ||
Sbjct  8903941  CTTACCGGCGAGTATTCTTTGTATTGAAACTTTGTTAAAGCACCAAGTCGGAAGTTTTGG  8903882

Query  1936     ATTCGTAAATTTTCTCGAAAATTTGTTAAATATTCTTGATAAGCGAATTCCAAAAGTTAA  1995
                 || || |||||| | || ||| |  ||| |||  | |||||  | || || ||||||||
Sbjct  8903881  TTTTGTTAATTTTTTAGAGAATCTTGTAAGTATCTTAGATAAAAGGATCCCTAAAGTTAA  8903822

Query  1996     TACTTTGTTAATTGCTGGTCCTGCTAATTCAGGAAAAAAtttttttttt--GATGCAGTG  2053
                |||  |  || | || || || || || ||||| || ||||||||||||  ||||||||
Sbjct  8903821  TACGCTACTAGTAGCAGGCCCGGCAAACTCAGGTAAGAATTTTTTTTTTTCGATGCAGTT  8903762

Query  2054     ACACAAGCTATGATAAATTGTGGTCACGTCGGAAATTTTAACAGATTTTGTAACTTTCCA  2113
                || ||||| ||| ||||||| || || || || ||||| ||||| |||||||| |||||
Sbjct  8903761  ACGCAAGCCATGTTAAATTGCGGACATGTAGGTAATTTCAACAGGTTTTGTAATTTTCCT  8903702

Query  2114     TTACAAGAGTGCAACATGAAAAGGATCTTATTATGGAATGAACCGGTTTGCGAACCATCC  2173
                || ||||||||| ||||||||||| ||  |  | ||||||||||| |||||||| || ||
Sbjct  8903701  TTGCAAGAGTGTAACATGAAAAGAATACTGATGTGGAATGAACCTGTTTGCGAGCCTTCT  8903642

Query  2174     GCGGTGGAAACTATAAAAATGCTTTTCGCTGGGGACCCTTGCCCCGCAAAAATTAAATAC  2233
                || || ||||| ||||||||| | || ||||| || ||||| ||||| ||||| |||||
Sbjct  8903641  GCTGTAGAAACGATAAAAATGTTATTTGCTGGAGATCCTTGTCCCGCTAAAATAAAATAT  8903582

Query  2234     AAAAATGATGCGACAATCATGCGCACTCCCGTAATAGTTCTAACGAACAACAATCCTTTC  2293
                || |  || || || |||||| | ||||| ||||| ||  |||  || || ||||| ||
Sbjct  8903581  AAGAGCGACGCAACTATCATGAGAACTCCTGTAATTGTATTAAAAAATAATAATCCGTTT  8903522

Query  2294     CCTAATGACCCGCCATTTAATAGTAGAATGATTAAATACACATGGCGACAGTCACCTTTC  2353
                |||| ||| || || || ||||| || ||||| ||||| || ||||| ||| | |||||
Sbjct  8903521  CCTATTGATCCTCCGTTCAATAGAAGGATGATAAAATATACTTGGCGCCAGGCTCCTTTT  8903462

Query  2354     TTGGCAGAAATTCATAAAAAACCTCACCCTTTAGCACTTTTTTATGTTTTGGCAAAGTAT  2413
                || ||||| || |||||||||||||| || || ||| ||||||| || || ||||| ||
Sbjct  8903461  TTAGCAGATATACATAAAAAACCTCATCCGTTGGCAATTTTTTACGTATTTGCAAAATAC  8903402

Query  2414     AACCTTATTGATACGTTGGAATTGGATAATTTCGACTCTAGTTTGTTGCAGGACTTAAGT  2473
                ||  | ||||||||  ||||  ||||||| || || || |||||||| |||||  | |||
Sbjct  8903401  AATTTGATTGATACTATGGATATGGATAACTTTGATTCAAGTTTGTTACAGGATCTTAGT  8903342

Query  2474     GTCACCACTTTCATACAGGA  2493
                ||   ||  |||||||||||
Sbjct  8903341  GTTTGCAGGTTCATACAGGA  8903322

Range 2: 8901792 to 8902091
Identities     Gaps          Strand
210/304(69%)   5/304(1%)     Plus/Minus

Query  1942     AAATTTTCTCGAAAATTTGTTAAATATTCTTGATAAGCGAAT-TCCAAAAGTTAATACTT  2000
                || |||| | |||  ||| |||   |||||||||||  |||| |   |||  ||||||||
Sbjct  8902091  AACTTTTTTAGAAGTTTTATTAGCAATTCTTGATAAA-GAATGTATGAAACGTAATACTT  8902033

Query  2001     TGTTAATTGCTGGTCCTGCTAATTCAGGAAAAAAttttttttttGATGCAGTGACACAAG  2060
                | ||||||  ||| ||  ||||||| || ||   ||||||||||  ||| ||  |||||
Sbjct  8902032  TATTAATTCTTGGACCCCCTAATTCTGGTAAT--TTTTTTTTTTATTGC-GTTTCACAAT  8901976

Query  2061     CTATGATAAATTGTGGTCACGTCGGAAATTTTAACAGATTTTGTAACTTTCCATTACAAG  2120
                 |||||||||||||||  |    || ||||  |||||||||   || || || || || |
Sbjct  8901975  TTATGATAAATTGTGGGTATACTGGCAATTCCAACAGATTTCAAAATTTCCCTTTTCAGG  8901916

Query  2121     AGTGCAACATGAAAAGGATCTTATTATGGAATGAACCGGTTTGCGAACCATCCGCGGTGG  2180
                |||||   |  ||| |  |  || ||||||| ||||     || ||| |||| ||  | |
Sbjct  8901915  AGTGCGTTAAAAAACGTTTGATAGTATGGAACGAACAACAATGTGAAACATCAGCATTTG  8901856

Query  2181     AAACTATAAAAATGCTTTTCGCTGGGGACCCTTGCCCCGCAAAAATTAAATACAAAAATG  2240
                ||  | |||| |  ||||| ||||| || || ||    | |||||||||| | ||||  |
Sbjct  8901855  AAGATGTAAAGAAACTTTTTGCTGGTGATCCATGTAATGTAAAAATTAAACATAAAATGG  8901796

Query  2241     ATGC  2244
```

```
                       ||||
Sbjct  8901795  ATGC  8901792
```

B. tabaci MEAM1 genome browser showing the positions of two EVEs related to BtaDV and aggregate RNA-seq data

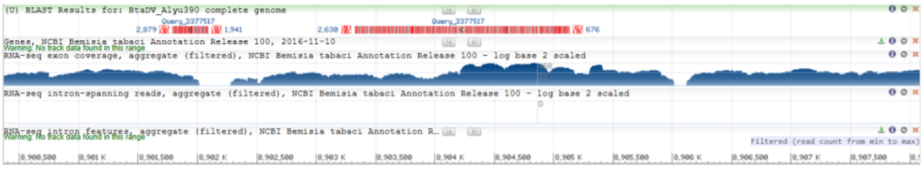

The proteins encoded in the EVEs (Rep endonuclease motifs, and part of SF3 helicase)

```
ORF40             1 --------------------------------------------ETEMLSSL      8
                                                            :||....|
BtaDV_NS1        51 LVRDGVTKCIPIVCAEDVLKKKPENPSNISGDGNAFEFVGSRQTEKSPYL    100

ORF40             9 SQADKAKIAHSSEKEHPEPIAGASTELSEAQKKVIQDFIDQSPLLDSHTG     58
                    .:......:.|..::.....||.|     ...:.|..|       ||.||
BtaDV_NS1       101 GEGSSGASSGSIPRDDGNKEAGNS-----GLYRSISPF-------DSDTG    138

ORF40            59 LHDGAYSRASSPASSHVGIRGNNRKRRIDEYFSGSEGSVAGGTDCYEESF    108
                    |..|...|.||.||:.|.:|..||.:||||||||:|.|:||.::.|:|||
BtaDV_NS1       139 LLHGTPPRPSSVASTDVRVRRRNRSKRIDEYFSGAESSLAGSSNSYQESF    188

ORF40           109 ETISFQKFKDLFCSEFGRLDHNRRYMLHDNYRNTLSGRFREDYRGIIPKN    158
                    ||:||:|||.|||:||||||::|||:|||.||||:|||||||:.||::||:
BtaDV_NS1       189 ETVSFEKFKSLFCAEFGRLDNHRRYILHDIYRNTISGRFRENDRGVVPKD    238

ORF40           159 TGNSSASWAFVVFEHGKHIHL---CRYERSWCSCALTKKAEKRFGRRVGR    205
                    |||||::|.||||:||:|:|:   |||||||||||||||||:|||||:||
BtaDV_NS1       239 TGNSSSAWTFVVFQHGQHVHVIHKCRYERSWCSCALTKKAEERFGRRIGR    288

ORF40           206 YSLRSSRVSKSHLSNLADYIQKGKRRLLQAFLARRERRLPGETGFLQNKE    255
                    ::||.||:::.||.:||:|||||:|||||||||.|||||..:.|.||:.:
BtaDV_NS1       289 HTLRGSRITEGHLEHLANYIQKGRRRLLQAFLAGRERRLSRQAGLLQDTQ    338

ORF40           256 PKKSCEREFVEGSGLPDHIRAFLSGSCGKSNKEDTQGTSEEDSSDSGPES    305
                    ..:|.:.|.|||..||:||.:||.|:|.|.::::.:.:|||||.:||.|.
BtaDV_NS1       339 SSQSGKGELVEGGRLPEHISSFLRGTCRKQHQKNPERSSEEDSENSGEEG    388

ORF40           306 GVPAPSKREKIIQFLQSCVITPFSRIFQSHLYLFDKSLFFITPESMSCQL    355
                    |||||:||||||||||||||||||||||||.||||||||:||||||||||
BtaDV_NS1       389 GVPAPTKREKIIQFLQSCVITPFSRIFQSQLYLFDKSLYFITPESMSCQL    438

ORF40           356 AILQHTHTINKMSVQQLFQYQLNIPYSKLFYNCDTPEDYFLNLPASILCI    405
                    |||||||||||:|||:|||||||||:||||||||.||||||||.||||||
BtaDV_NS1       439 AILQHTHTINKMTVQQIFQYQLNIPFSKLFYNCDNPEDYFLNLSASILCI    488

ORF40           406 ETLLKHQVGSFGFVNFLENLVSILDKRIPKVNTLLVAGPANSGKNFFFS-    454
                    ||||||||||||||||||||::|||||||||||||||:||||||||||||.
BtaDV_NS1       489 ETLLKHQVGSFGFVNFLENLLNILDKRIPKVNTLLIAGPANSGKNFFFDA    538

ORF40           455 -MQLRKPC------------------------------------------    461
                     .|....|
BtaDV_NS1       539 VTQAMINCGHVGNFNRFCNFPLQECNMKRILLWNEPVCEPSAVETIKMLF    588


ORF31             1 ----------------------------------------------NAFF      4
                                                                  ....
BtaDV_NS1       351 GRLPEHISSFLRGTCRKQHQKNPERSSEEDSENSGEEGGVPAPTKREKII    400

ORF31             5 SHAIFCYVPFFPESSSITPSSRLLDTETWHFGP--FKGTPETQYRVQQAL     52
                    .          |.:|..|||.||:..::.:.|..  :..|||:. ..|.|:
BtaDV_NS1       401 Q---------FLQSCVITPFSRIFQSQLYLFDKSLYFITPESM-SCQLAI    440

ORF31            53 TRYKLKINKMSVCDLAKNAFNLKPENIFFNSENPVLEFYSVLQSIQILEL    102
                    .::...||||:|..:.:...|:....:|:|.:||...|.::..||..:|.
BtaDV_NS1       441 LQHTHTINKMTVQQIFQYQLNIPFSKLFYNCDNPEDYFLNLSASILCIET    490

ORF31           103 LLMHQYGN-NVKTFLEVLLAILDKECMKRNTLLILGPPNSG-NFFFYCVS    150
                    ||.||.|: ....|||.||.||||...|.|||||.||.||| ||||..|:
BtaDV_NS1       491 LLKHQVGSFGFVNFLENLLNILDKRIPKVNTLLIAGPANSGKNFFFDAVT    540

ORF31           151 QFMINCGYTGNSNRFQNFPFQECVKKRLIVWNEQQCETSAFEDVKKLFAG    200
                    |.|||||:.||.|||.|||.|||..||:::|||..||.||.|.:|.||||
BtaDV_NS1       541 QAMINCGHVGNFNRFCNFPLQECNMKRILLWNEPVCEPSAVETIKMLFAG    590

ORF31           201 DPCNVKIKHKMDAIVLRTPFIVLTNSDIFRHPLYLHTLHCIRTLPRTAFP    250
                    |||..|||:|.||.::|||.||||::.|.:                   .
BtaDV_NS1       591 DPCPAKIKYKNDATIMRTPVIVLTNNNPFPN------------------D    622

ORF31           251 PPTNRKSS------------------------------------------    258
                    ||.|.:..
BtaDV_NS1       623 PPFNSRMIKYTWRQSPFLAEIHKKPHPLALFYVLAKYNLIDTLELDNFDS    672
```

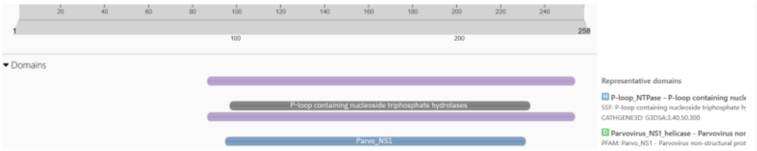

BtaDV-related EVEs in the genome of *B. tabaci* ASIAII-5 population from India (BioProject PRJEB39408)

Descriptions | Graphic Summary | Alignments

Sequences producing significant alignments — Download — Manage columns — Show 100

select all 6 sequences selected — GenBank — Graphics — Distance tree of results — MSA Viewer

Description	Max Score	Total Score	Query Cover	E value	Per. Ident	Acc. Len	Accession
Bemisia tabaci genome assembly, contig: CONTIG127	608	608	17%	5e-171	75.48%	11517917	CAJEWF010000013.1
Bemisia tabaci genome assembly, contig: CONTIG219	114	166	10%	8e-23	66.51%	41757880	CAJEWF010000002.1
Bemisia tabaci genome assembly, contig: CONTIG19	92.4	143	5%	9e-16	70.62%	33883951	CAJEWF010000003.1
Bemisia tabaci genome assembly, contig: CONTIG191	71.6	71.6	2%	9e-10	75.20%	6689163	CAJEWF010000027.1
Bemisia tabaci genome assembly, contig: CONTIG152	53.6	53.6	2%	2e-04	70.54%	20761191	CAJEWF010000007.1
Bemisia tabaci genome assembly, contig: CONTIG133	47.3	47.3	4%	0.034	67.02%	3470409	CAJEWF010000044.1

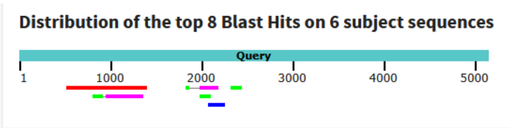

Bemisia tabaci genome assembly, contig: CONTIG127, whole genome shotgun sequence

```
Sequence ID: CAJEWF010000013.1 Length: 11517917 Number of Matches: 1
Identities      Gaps            Strand
671/889(75%)    10/889(1%)      Plus/Plus

Query  512      GTGCAAGTTGAACAATTATCCCCTAGTGAAGAAAGGTACGTTGAACATTGTATCGCAACA  571
                || ||| | ||| | ||| |  |||||||||||| |||||| || ||||| ||   ||||
Sbjct  3219697  GTTCAAATCGAAGATTTAACGTCTAGTGAAGAAAAGTACGTGGAGCATTGCATTCAAACA  3219756

Query  572      AACCAGCCTGTTTATTTAGTCAGGGACGGAGTAACAAAGTGTATCCCGATAGTTTGTGCA  631
                || || ||||| ||  ||||  | ||||| |  || |||||||| ||  ||||||||||
Sbjct  3219757  AATCAACCTGTGTACCTAGTGCGCGACGGTGCTACTAAGTGTATACCCGTAGTTTGTGCT  3219816

Query  632      GAAGATGTCCTCaaaaaaaaGCCGGAAAACCCGTCAAACATCTCCGGAGACGGAAATGCT  691
                |||||| || ||||||| |  ||| |||||||||||| | || ||||||||||| ||||
Sbjct  3219817  GAAGATATCTTCAAAAAGATCCCGCAAAACCCGTCAAGCGTCCCCGGAGACGGAGATGCC  3219876

Query  692      TTCGAATTTGTCGGAAGTAGACAAACAGAAAAAAGTCCATATCTTGGAGAAGGATCCTCC  751
                ||||||||||||| ||| | | |||  || || || |||| ||| |||| || | |  ||
Sbjct  3219877  TTCGAATTTGTCGCAAGCACAGAAAACGACAACAGCCCATTTCTCGGAGCAGCAACGGCC  3219936

Query  752      GGAGCCTCAAGCGGGTCCATCCCTCGAGA----TGACGGAAACAAGGAAGCAGGAAATTC  807
                ||| ||||  |||  || |  | |||||     |||| ||  |   ||   |||  || |
Sbjct  3219937  GGACCCTCCTGCG--TCAA--CTTCGAGGGATCTGACCGACGCTCAGATAAAGGTGATAC  3219992

Query  808      AGGACTTTATCGATCGATCTCCCCTTTTGACAGCGATACAGGATTACTCCATGGAACACC  867
                | || || || || | ||| |||||  |  ||||  | ||||||||| | | ||  || |
Sbjct  3219993  AAGATTTCATAGAGCAATCACCCCTCCTATCAGCTTTGCAGGATTACGCGACGGGTCATC  3220052

Query  868      CCCTCGGCCTTCTTCCGTTGCCTCGACTGATGTCCGAGTTCGTAGGCGTAATCGAAGCAA  927
                | || || ||||||||  ||||||| |||||||| || ||  | ||| |||| |||
Sbjct  3220053  CTCTGGGGCTTCTTCCCCTGCCTCGTCTGATGTCGGAATTTATCGGCCTAATAGAAAGCG  3220112

Query  928      ACGGATTGACGAATATTTCTCAGGAGCAGAAAGTTCTTTGGCGGGAAGCTCAAATAGCTA  987
                |||||||||| ||||||||   ||| ||||| | ||| |||    | |  || |||||||
Sbjct  3220113  ACGGATTGACCAATATTTCAGCGGAACAGAAGGATCTGTGGAAACAGGGACAGATAGCTA  3220172

Query  988      CCAAGAAAGTTTTGAAACCGTTTCTTTCGAAAAATTTAAAAGCTTATTCTGTGCAGAATT  1047
                 ||||| |||||||||||||| ||||||  |||| |||||  |||||||||| | |||||
Sbjct  3220173  TCAAGAGAGTTTTGAAACCGTATCTTTCTCAAAACTTAAAGACTTATTCTGTTCTGAATT  3220232

Query  1048     TGGCCGACTGGATAATCACCGAAGGTACATATTACACGACATTTACCGAAATACAATTTC  1107
                |||    | ||||| ||     || | ||| |  | ||||| |||||||||||| |  ||||
Sbjct  3220233  TGGGAAAGTGGATCATAGAAGACGATACTTGCTCCACGATATTTACCGAAATTCGCTTTC  3220292

Query  1108     CGGGCGCTTTCGGGAAAATGATAGGGGAGTCGTACCCAAGGACACAGGCAACTCATCATC  1167
                ||| |||||||||||| ||||||||||||||||||||||   | ||||| |||||||| ||
Sbjct  3220293  CGGCCGCTTTCGGGAAGATGATAGGGGAGTCGTACCCAAAAATACAGGGAACTCATCGTC  3220352

Query  1168     GGCATGGACATTCGTCGTCTTCCAGCACGGCCAGCATGTCCACGTCATCCACAAGTGCCG  1227
                  | ||| | |||||| ||||||| |||||  |||| |||||| ||||||||| |||| |
Sbjct  3220353  ATCGTGGGCGTTCGTCCTCTTCCAACACGGGAAGCACGTCCACCTCATCCACATGTGCAG  3220412

Query  1228     ATACGAAAGGTCGTGGTGCTCGTGCGCACTCACGAAGAAAGCGGAAGAGCGATTCGGACG  1287
                |||||| || || ||||||||||| ||  |||| || |||| |||  |||| ||||||||
Sbjct  3220413  ATACGACAGATCATGGTGCTCGTGTGCGGTCACCAAAAAAGAGGAGAAGCGGTTCGGACG  3220472

Query  1288     ACGAATTGGAAGACATACTTTACGAGGCAGTAGGATCACAGAAGGCCACCTCGAACATCT  1347
                |||||||||||||||| ||||||||| ||||| ||||| || || ||||||||     ||||
Sbjct  3220473  ACGAATTGGAAGACATTCTTTACGAAGCAGTCGGATCTCAAAAAGCCACCTCAGCAATCT  3220532

Query  1348     CGCAAATTATATACAAAAAGGG-CGACGCAGGCTCCTTCAAGCTTTCCT  1395
                |||| | ||||||||||| ||| ||| |  || | ||||||||||| ||
Sbjct  3220533  CGCAGACTATATACAAAAGGGGACGA-GACGGTTGCTTCAAGCTTTTCT  3220580
```

The protein encoded in the integrated sequence (Rep endonuclease motifs)

```
ORF1              1 ------------------------VQIEDLTSSEEKYVEHCIQTNQPVY     25
                                            ||:|.|:.|||:||||||.||||||
BtaDV_NS1         1 MSAHLSDDNSSQDSFSVIDTDSSEFVQVEQLSPSEERYVEHCIATNQPVY     50

ORF1             26 LVRDGATKCIPVVCAEDIFKKIPQNPSSVPGDGDAFEFVASTENDNSPFL     75
                    |||||.|||||:|||||:.||.|:|||::.|||:|||||.|.:.:.||:|
BtaDV_NS1        51 LVRDGVTKCIPIVCAEDVLKKKPENPSNISGDGNAFEFVGSRQTEKSPYL    100

ORF1             76 GAATAGPSCVNFEGSDRRSDKGDTRFHRAITPPISFAGLRDGSSSGASSP    125
                    |..::|.|..:....|...:.|::..:|:|:|..|..||..|:....||.
BtaDV_NS1       101 GEGSSGASSGSIPRDDGNKEAGNSGLYRSISPFDSDTGLLHGTPPRPSSV    150

ORF1            126 ASSDVGIYRPNRKRRIDQYFSGTEGSVETGTDSYQESFETVSFSKLKDLF    175
                    ||:||.:.|.||.:|||:||||.|.|:...::||||||||||.|.|.||
BtaDV_NS1       151 ASTDVRVRRRNRSKRIDEYFSGAESSLAGSSNSYQESFETVSFEKFKSLF    200

ORF1            176 CSEFGKVDHRRRYLLHDIYRNSLSGRFREDDRGVVPKNTGNSSSSWAFVL    225
                    |:||||::|:.|||:||||||||::||||||:||||||||:||||||:|.||:
BtaDV_NS1       201 CAEFGRLDNHRRYILHDIYRNTISGRFRENDRGVVPKDTGNSSSAWTFVV    250

ORF1            226 FQHGKHVHLIHMCRYDRSWCSCAVTKKEEKRFGRRIGRHSLRSSRISKSH    275
                    ||||:|||:||.|||:||||||||:|||.|:|||||||||:||.|||::.|
BtaDV_NS1       251 FQHGQHVHVIHKCRYERSWCSCALTKKAEERFGRRIGRHTLRGSRITEGH    300

ORF1            276 LSNLADYIQKGTRRLLQAF-------------------------------    294
                    |.:||:|||||.|||||||
BtaDV_NS1       301 LEHLANYIQKGRRRLLQAFLAGRERRLSRQAGLLQDTQSSQSGKGELVEG    350
```

Bemisia tabaci genome assembly, contig: CONTIG219, whole genome shotgun sequence

```
Sequence ID: CAJEWF010000002.1 Length: 41757880 Number of Matches: 2
Range 1: 29654188 to 29654603
Identities      Gaps            Strand
278/418(67%)    5/418(1%)       Plus/Plus

Query  940       ATATTTCTCAGGAGCAGAAAGTTCTTTGGCGGGAAGCTCAAA--TAGCTACCAAGAAAGT  997
                 ||||||| | ||||||| ||  | | |||   ||||| ||||  | | |  ||| ||
Sbjct  29654188  ATATTTCGCTGGAGCAGCAAACTATGTGGAAAGAAGCGCAAAGGTTGGT--CAAAAATTA  29654245

Query  998       TTTGAAACCGTTTCTTTCGAAAAATTTAAAAGCTTATTCTGTGCAGAATTTGGCCGACTG  1057
                 | ||| ||| ||||||||  | ||||||||  | ||||   |   | | ||| | |   |
Sbjct  29654246  TATGAGACCATTTCTTTCATAGAATTTAAAGACCTATTTAATCGTGGACTTGACAGCGCG  29654305

Query  1058      GATAATCACCGAAGGTACATATTACACGACATTTACCGAAATACAATTTCCGGGCGCTTT  1117
                 | |  ||   ||| |||| || | |||||||||||| |||| |    | |||| ||||||
Sbjct  29654306  GCTGCTCGAAGAAAGTACCTAGTTCACGACATTTACAGAAACAGGGCTACCGGACGCTTT  29654365

Query  1118      -CGGGAAAATGATAGGGGAGTCGTACCCAAGGACACAGGCAACTCATCATCGGCATGGAC  1176
                  |    ||||||||  ||| || ||||| | |  ||||| ||| |  |   | | |||||
Sbjct  29654366  GCTTCGAAATGATACAGGAATCTTACCCCAAGCAACAGGGAACGCGGCGGGGACGTGGAC  29654425

Query  1177      ATTCGTCGTCTTCCAGCACGGCCAGCATGTCCACGTCATCCACAAGTGCCGATACGAAAG  1236
                     ||||||| | | |  || || || ||||||||| ||||    || || ||||| ||
Sbjct  29654426  GACTGTCGTCTGCAAACGTGGACAACACGTCCACGTCCTCCATCTCTGTCGCTACGACAG  29654485

Query  1237      GTCGTGGTGCTCGTGCGCACTCACGAAGAAAGCGGAAGAGCGATTCGGACGACGAATTGG  1296
                  |||||||| ||||||||| |||  || |||||||||  |    |||||||| || ||
Sbjct  29654486  TTCGTGGTGTTCGTGCGCAATCATTAAAAAAGCGGAAAGGGTCCTCGGACGAAGATTTAA  29654545

Query  1297      AAGACATACTTTACGAGGCAGTAGGATCACAGAAGGCCACCTCGAACATCTCGCAAAT  1354
                 | ||| || | |||   || || || |||      |  ||| ||||| ||||  |||||
Sbjct  29654546  ACGACGTAGTATACACTGCCGTCGGCTCAGCTCGGAGCACGTCGAAAATCTGTCAAAT  29654603


Range 2: 29654074 to 29654186
Identities      Gaps            Strand
79/113(70%)     0/113(0%)       Plus/Plus

Query  798       CAGGAAATTCAGGACTTTATCGATCGATCTCCCCTTTTGACAGCGATACAGGATTACTCC  857
                 ||||  ||||||||||| || ||| ||||||| |    |||  ||  | || |  | | |
Sbjct  29654074  CAGGCCATTCAGGACTTCATAGATGGATCTCCTCACCTGAGCGCTCTTCAAGCCTGCACG  29654133

Query  858       ATGGAACACCCCCTCGGCCTTCTTCCGTTGCCTCGACTGATGTCCGAGTTCGT  910
```

```
                    ||||| ||  | || || |||||| || | || || || ||| | ||||||||
Sbjct  29654134  ATGGATCATACGCTGGGTCTTCTTACGGTTCCACGGCTCATGCCGGAGTTCGT  29654186
```

The protein encoded in the integrated sequence (Rep endonuclease motifs)

```
ORF5              1 --------------MTTFTETGLPDALLRNDTGILPQATGNAAGTWTTVV     36
                                  :.......:......||.|::|:.|||::..||.||
BtaDV_NS1       201 CAEFGRLDNHRRYILHDIYRNTISGRFRENDRGVVPKDTGNSSSAWTFVV    250

ORF5             37 CKRGQHVHVLHLCRYDSSWCSCAIIKKAERVLGRRFKRRSIHCRRLSSEH     86
                    .:.||||||:|.|||:.||||||:.||||...|||..|.::...|::..|
BtaDV_NS1       251 FQHGQHVHVIHKCRYERSWCSCALTKKAEERFGRRIGRHTLRGSRITEGH    300

ORF5             87 VENLSN--------------------------------------------     92
                    :|:|:|
BtaDV_NS1       301 LEHLANYIQKGRRRLLQAFLAGRERRLSRQAGLLQDTQSSQSGKGELVEG    350
```

BtaDV-related EVEs in the genome of *B. tabaci* Q1 (BtQ1) from China (BioProject PRJNA276952)

Descriptions | Graphic Summary | Alignments

Sequences producing significant alignments — Download — Manage columns — Show 100

select all — *21 sequences selected* — GenBank — Graphics — Distance tree of results — MSA Viewer

Description	Max Score	Total Score	Query Cover	E value	Per. Ident	Acc. Len	Accession
Bemisia tabaci isolate CAASIVF2012BtQ Scaffold_783_2	186	186	7%	6e-44	71.54%	79528	LIED01027279.1
Bemisia tabaci isolate CAASIVF2012BtQ unplaced genomic scaffold Scaffold_783	186	186	7%	6e-44	71.54%	317319	ML134514.1
Bemisia tabaci isolate CAASIVF2012BtQ Scaffold_436_3	106	106	13%	4e-20	64.24%	46007	LIED01020174.1
Bemisia tabaci isolate CAASIVF2012BtQ unplaced genomic scaffold Scaffold_436	106	106	13%	4e-20	64.24%	462213	ML134278.1
Bemisia tabaci isolate CAASIVF2012BtQ Scaffold_54_38	95.1	95.1	3%	8e-17	75.18%	29365	LIED01022795.1
Bemisia tabaci isolate CAASIVF2012BtQ unplaced genomic scaffold Scaffold_54	95.1	95.1	3%	8e-17	75.18%	1170270	ML133946.1
Bemisia tabaci isolate CAASIVF2012BtQ Scaffold_48_22	89.7	148	3%	3e-15	74.83%	31732	LIED01021406.1
Bemisia tabaci isolate CAASIVF2012BtQ unplaced genomic scaffold Scaffold_48	89.7	148	3%	3e-15	74.83%	1229738	ML133940.1
Bemisia tabaci isolate CAASIVF2012BtQ Scaffold_30_56	66.2	66.2	2%	4e-08	76.67%	11487	LIED01014544.1
Bemisia tabaci isolate CAASIVF2012BtQ unplaced genomic scaffold Scaffold_30	66.2	174	4%	4e-08	76.67%	1461140	ML133923.1
Bemisia tabaci isolate CAASIVF2012BtQ Scaffold_2636_2	65.3	65.3	7%	1e-07	64.75%	51115	LIED01012506.1
Bemisia tabaci isolate CAASIVF2012BtQ unplaced genomic scaffold Scaffold_2636	65.3	65.3	7%	1e-07	64.75%	111835	ML135507.1
Bemisia tabaci isolate CAASIVF2012BtQ Scaffold_2366_7	59.0	59.0	2%	6e-06	77.78%	21894	LIED01011057.1
Bemisia tabaci isolate CAASIVF2012BtQ unplaced genomic scaffold Scaffold_2366	59.0	59.0	2%	6e-06	77.78%	125497	ML135379.1
Bemisia tabaci isolate CAASIVF2012BtQ Scaffold_30_55	55.4	108	3%	7e-05	74.71%	37985	LIED01014543.1
Bemisia tabaci isolate CAASIVF2012BtQ Scaffold_2332_2	53.6	53.6	2%	2e-04	71.20%	121972	LIED01010852.1
Bemisia tabaci isolate CAASIVF2012BtQ unplaced genomic scaffold Scaffold_2332	53.6	53.6	2%	2e-04	71.20%	127663	ML135362.1
Bemisia tabaci isolate CAASIVF2012BtQ Scaffold_104_8	52.7	52.7	2%	8e-04	72.38%	36319	LIED01000588.1
Bemisia tabaci isolate CAASIVF2012BtQ Scaffold_2022_7	51.8	155	2%	8e-04	70.16%	10446	LIED01008845.1
Bemisia tabaci isolate CAASIVF2012BtQ unplaced genomic scaffold Scaffold_104	52.7	52.7	2%	9e-04	72.38%	904854	ML133992.1
Bemisia tabaci isolate CAASIVF2012BtQ unplaced genomic scaffold Scaffold_2022	51.8	155	2%	9e-04	70.16%	146841	ML135215.1

Distribution of the top 30 Blast Hits on 21 subject sequences

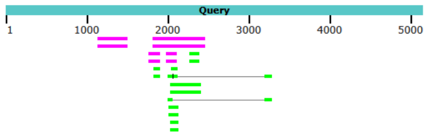

Bemisia tabaci isolate CAASIVF2012BtQ Scaffold_783_2, whole genome shotgun sequence

```
Sequence ID: LIED01027279.1 Length: 79528 Number of Matches: 1
Identities      Gaps          Strand
269/376(72%)    3/376(0%)     Plus/Minus

Query  1125   ATGATAGGGGAGTCGTACCCAAGGACACAGGCAACTCATCATCGGCATGGACATTCGTCG  1184
              ||||| | ||| || |||||||   |||||| ||| |   | ||  | ||| || ||||||
Sbjct  50196  ATGATGGCGGAATCTTACCCAAAAGCACAGGGAACGCGGCGTCAACGTGGTCAATCGTCG  50137

Query  1185   TCTTCCAGCACGGCCAGCATGTCCACGTCATCCACAAGTGCCGATACGAAAGGTCGTGGT  1244
              | | | | |||||    || ||| |  || |||||   || |||||||| || || ||||
Sbjct  50136  TGTGCGAACACGGAAGCCACGTCTATCTCCTCCACCTATGACGATACGACAGATCCTGGT  50077

Query  1245   GCTCGTGCGCACTCACGAAGAAAGCGGAA-GAGCGATTCGGACGACGAATTGGAAGACAT  1303
              ||||||| ||  |||  || || |||||| ||| | | ||||||||||||||| || ||
Sbjct  50076  GCTCGTGTGCGGTCAAAAAAAAGGCGGAAAGAGTGCT-CGGACGACGAATTGGACGATAT  50018

Query  1304   ACTTTACGAGGCAGTAGGATCACAGAAGGCCACCTCGAACATCTCGCAAATTATATACAA  1363
              ||| | ||| ||| | || || |  |||  ||| || || ||||| ||| |||| |||||
Sbjct  50017  ACTATTCGAAGCATTCGGCTCTCTAAAGAGCACATCAAAAATCTCTCAAGTTATCTACAA  49958

Query  1364   AAAGGGCGACGCAGGCTCCTTCAAGCTTTCCTCGCCGGGCGAGAAAGGCGATTATCTCGT  1423
              |||||||  || ||| || ||||||||||  || || |||| ||| || |||||  |  |
Sbjct  49957  AAAGGGCAGCGAAGGGTCATTCAAGCTTTTTCTCCAGGCGGGAATGGAGATTACATGAT  49898

Query  1424   CAA-GCTGGACTTTTACAAGACACGCAATCTTCGCAGAGTGGCAAAGGAGAACTGGTGGA  1482
              ||| | | ||| | |||   |||||  | ||  | ||||   | ||||| ||||||||||
Sbjct  49897  CAAGGTTAGACCTGTACCGCACACGAGAACTGAGGAGAGCACCGAAGGAAAACTGGTGGA  49838

Query  1483   AGGAGGCCGATTACCG  1498
              |  | || || |||||
Sbjct  49837  AACAAGCGGACTACCG  49822
```

```
ORF12             1 -------------RYDRSWCSCAVKKKAERVLGRRIGRYTIRSIRLSKEH     37
                                 ||:||||||||:.||||...||||||:|:|..|:::.|
BtaDV_NS1       251 FQHGQHVHVIHKCRYERSWCSCALTKKAEERFGRRIGRHTLRGSRITEGH    300

ORF12            38 IKNLSSYLQKGQRRVIQAFFSRREWRLHDQG-------------------     68
                    :::|::|:|||:||::|||.:.:.||.||..|.
BtaDV_NS1       301 LEHLANYIQKGRRRLLQAFLAGRERRLSRQAGLLQDTQSSQSGKGELVEG    350
```

Bemisia tabaci isolate CAASIVF2012BtQ unplaced genomic scaffold Scaffold_436, whole genome shotgun sequence

```
Sequence ID: ML134278.1 Length: 462213 Number of Matches: 1
Identities      Gaps          Strand
424/660(64%)    22/660(3%)    Plus/Minus

Query  1800   TATTTCAGTACCAGTTAAATATTCCTTTCTCAAAATTGTTTTATAATTGCGATAATCCTG  1859
              ||||||| |  ||| | |||||||| || |||||| | |||||| |||   || |||||
Sbjct  89954  TATTTCAATTGCAGCTTAATATTCCATTTTCAAAACTATTTTATGATTCATATGATCCTT  89895

Query  1860   AGGATTATTTTTTGAATTTATCTGCTAGCATCCTTTGCATTGAAACTTTATTGAAACACC  1919
                   | |||||  ||||   | | |||||  |     | |||| |||||| |  || |
Sbjct  89894  CCTCCCAATTTTTAGATTTGATTTCCAGCATAATAATATTAGAAAGTTTATTAATCCATC  89835

Query  1920   AGGTTGGCAGTTTCGGATTCGTAA---ATTTTCTCGAAAATTT--GTTAAATATTCTTGA  1974
              | ||||| ||   ||     || |   ||||| |  | |||||  |||  || ||   ||
Sbjct  89834  AAGTTGGTAG---CGATAATGTTATGGATTTTTTATATAATTTAAGTTGCATTTTA--GA  89780

Query  1975   TAAGCGAATTCCAAAAGTTAATACTTTGTTAATTGCTGGTCCTGCTAATTCAGGAAAAAA  2034
              ||||  |||||| ||    ||||| || || |||       ||  ||||| | || |||||
Sbjct  89779  TAAGAAAATTCCCAAGAAAAATACATTATTTATTTTATCGCCGCCTAATGCTGGTAAAAA  89720
```

```
Query  2035   tttttttttGATGCAGTGACACAAGCTATGATAAATTGTGGTCACGTCGGAAATTTTAA  2094
              |||||||||||||   ||  |||| || ||||| |||   || ||| | |||||||||||
Sbjct  89719  TTTTTTTTTGATTGCGTAGCACAGGCAATGATTAATGCAGGCCACATAGGAAATTTTAA  89660

Query  2095   CAGATTTTGTAACTTTCCATTACAAGAGTGCAACA-TGAAAAGGATCTTATTATGGAATG  2153
              ||| ||||     |||||  ||   ||| |||  |   || || || ||| |||||||||
Sbjct  89659  CAGGTTTTCATCATTTCCGCTAATGGAG-GCAGTAGACAAGAGAATTTTAATATGGAATG  89601

Query  2154   AACCGGTTTGCGAACCATCCGCGGTGGAAACTATAAAAATGCTTTTCGCTGGGGACCCTT  2213
              |||||  |   || || || ||  | |||  | ||||||   | || |  || || ||||
Sbjct  89600  AACCGAATGCAGAGCCTTCAGCCTTTGAAGATTTAAAAAAATTATTTGGAGGTGATCCTT  89541

Query  2214   GCCCCGCAAAAATTAAATACAAAAATGATGCGACAATCATGCGCACTCCCGTAATAGTTC  2273
              |   |   ||| ||||||  ||   ||||   | | | ||| | ||||| || || |||
Sbjct  89540  GTAACTGTAAAGTTAAATTTAAGGGTGATAACATAGTTATGAGAACTCCAGTTATTGTTT  89481

Query  2274   TAACGAACAACAATCCTTTCCCTAATGACCCGCCA----TTTAATAG-TAGAATGATTAA  2328
              || | ||  |  ||  ||| ||      | |||  |    ||| | ||  |||||||| ||
Sbjct  89480  TATCAAATGAAGATATTTTTCC-----ATCCGAAAAAGCTTTCAGAGCAAGAATGATAAA  89426

Query  2329   ATACACATGGCGACAGTCACCTTTCTTGGCAGAAATTCATAAAAAACCTCACCCTTTAGC  2388
              |   | ||||  |    | ||||| ||   ||| ||| | || ||||| || || |||||
Sbjct  89425  AGTAAAATGGAAAGCTGCTCCTTTTTTAAAAGATATTAAAAAGAAACCGCATCCATTAGC  89366

Query  2389   ACTTTTTTATGTTTTGGCAAAGTATAACCTTATTGATACGTTGGAATTGGATAATTTCGA  2448
                |||||||| | |||||||| || ||| |  | ||||| |  || || || ||||| ||
Sbjct  89365  TTTTTTTTATTTATTGGCAAAATACAACATCTTAGATACATATGATTTAGAAAATTTTGA  89306
```

```
ORF11               1 -----FQLQLNIPFSKLFYDSYDPSSQFLDLISSIIILESLLIHQVGSDN    45
                           ||.||||||||||:..:|...||:|.:||:.:|:||.|||||..
BtaDV_NS1         451 TVQQIFQYQLNIPFSKLFYNCDNPEDYFLNLSASILCIETLLKHQVGSFG   500

ORF11              46 VMDFLYNLSCILDKKIPKKNTLFILSPPNAGKNFFFDCVAQAMINAGHIG    95
                      .::||.||..||||:|||.|||.|..|.|:|||||||.|.|||||.||:|
BtaDV_NS1         501 FVNFLENLLNILDKRIPKVNTLLIAGPANSGKNFFFDAVTQAMINCGHVG   550

ORF11              96 NFNRFSSFPLMEAVDKRILIWNEPNAEPSAFEDLKKLFGGDPCNCKVKFK   145
                      |||||.:|||.|...||||:||||..||||.|.:|.||.||||..|:|:|
BtaDV_NS1         551 NFNRFCNFPLQECNMKRILLWNEPVCEPSAVETIKMLFAGDPCPAKIKYK   600

ORF11             146 GDNIVMRTPVIVLSNEDIFPSEKAFRARMIKVKWKAAPFLKDIKKKPHPL   195
                      .|..:||||||||:|.:.||::..|.:||||..|:.:|||.:|.||||||
BtaDV_NS1         601 NDATIMRTPVIVLTNNNPFPNDPPFNSRMIKYTWRQSPFLAEIHKKPHPL   650

ORF11             196 AFFYLLAKYNILDTYDLENF------------------------------   215
                      |.||:||||||::||.:|:||
BtaDV_NS1         651 ALFYVLAKYNLIDTLELDNFDSSLLQDLSVTTFIQEATHDIDSSIRSTEA   700
```

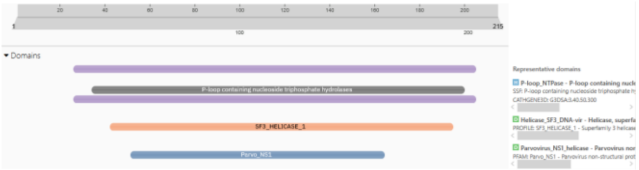

BtaDV-related EVEs in the genome of *B. tabaci* AsiaII1 pooled male and female from China (BioProject PRJNA553779)

Descriptions | Graphic Summary | Alignments

Sequences producing significant alignments Download Manage columns Show 100

select all *5 sequences selected* GenBank Graphics Distance tree of results MSA Viewer

	Description	Max Score	Total Score	Query Cover	E value	Per. Ident	Acc. Len	Accession
☑	Bemisia tabaci isolate AsiaII1 CTG4135_PILON_PILON_PILON_PILON	114	162	10%	8e-23	66.51%	25342	VMEH01002382.1
☑	Bemisia tabaci isolate AsiaII1 CTG588_PILON_PILON_PILON_PILON	62.6	108	7%	5e-07	66.08%	203000	VMEH01003073.1
☑	Bemisia tabaci isolate AsiaII1 CTG1066_PILON_PILON_PILON_PILON	56.3	56.3	2%	7e-05	72.07%	73581	VMEH01000214.1
☑	Bemisia tabaci isolate AsiaII1 CTG58_PILON_PILON_PILON_PILON	50.0	50.0	3%	0.003	69.47%	1135435	VMEH01003078.1
☑	Bemisia tabaci isolate AsiaII1 CTG121_PILON_PILON_PILON_PILON	47.3	47.3	4%	0.035	67.02%	642075	VMEH01000671.1

Distribution of the top 7 Blast Hits on 5 subject sequences

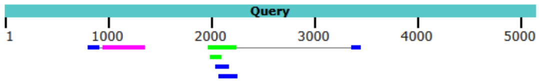

BtaDV-related EVEs in the genome of *B. tabaci* AsiaII6 pooled male and female from China (BioProject PRJNA553780)

Descriptions | Graphic Summary | Alignments

Sequences producing significant alignments Download Manage columns Show 100

select all *6 sequences selected* GenBank Graphics Distance tree of results MSA Viewer

	Description	Max Score	Total Score	Query Cover	E value	Per. Ident	Acc. Len	Accession
☑	Bemisia tabaci isolate AsiaII6 CTG692_PILON_PILON_PILON_PILON	1110	1110	27%	0.0	77.95%	142467	VMEG01003373.1
☑	Bemisia tabaci isolate AsiaII6 CTG434_PILON_PILON_PILON_PILON	138	138	8%	7e-30	68.09%	947840	VMEG01002263.1
☑	Bemisia tabaci isolate AsiaII6 CTG449_PILON_PILON_PILON_PILON	122	169	10%	6e-25	66.82%	220288	VMEG01002335.1
☑	Bemisia tabaci isolate AsiaII6 CTG13_PILON_PILON_PILON_PILON	73.4	73.4	2%	3e-10	74.81%	3945770	VMEG01000767.1
☑	Bemisia tabaci isolate AsiaII6 CTG350_PILON_PILON_PILON_PILON	51.8	51.8	2%	8e-04	71.17%	275467	VMEG01001872.1
☑	Bemisia tabaci isolate AsiaII6 CTG44_PILON_PILON_PILON_PILON	47.3	47.3	4%	0.036	67.02%	1747175	VMEG01002336.1

Distribution of the top 7 Blast Hits on 6 subject sequences

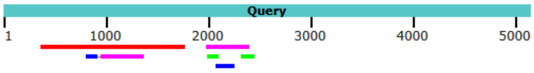

Bemisia tabaci isolate AsiaII6 CTG692_PILON_PILON_PILON_PILON, whole genome shotgun sequence

```
Sequence ID: VMEG01003373.1Length: 142467 Number of Matches: 1
Range 1: 35132 to 36529
Identities     Gaps          Strand
```

```
1096/1406(78%) 13/1406(0%)    Plus/Minus

Query  358    TGATAAGAGTTTAAACAAG-GTAGGTAAATAAAAGGTGGCTCGAGTGCGGGATTCGTCGC  416
              |||||| ||  |||||||  ||| |||||| |||||||   |||| | ||||    ||
Sbjct  36529  TGATAAAAGAGTAAACAACCGTATGTAAATGAAAGGTG---CGAGCGAGGGAACGATCAT  36473

Query  417    AGTTC--AGtttttttGCCGGTATGTCTGCTCATTTAAGCGACGATAACAGTTCTCAAGA  474
              |  ||  |  |||||||  | ||||||||  ||| ||   ||||||||||| ||  || |
Sbjct  36472  ATATCGTATGTTTTTTG--GATATGTCTGTGCATCTATCTGACGATAACAGCTCGGAAAA  36415

Query  475    TTCATTTTCAGTGATAGATACAGATAGTAGTGAATTCGTGCAAGTTGAACAATTATCCCC  534
              ||| |||||| | |||||||| |||  |||||| || |||||||||||||| ||||| ||
Sbjct  36414  TTCGTTTTCAATTATAGATACCGATTCTAGTGATTTTGTGCAAGTTGAACAGTTATCTCC  36355

Query  535    TAGTGAAGAAAGGTACGTTGAACATTGTATCGCAACAAACCAGCCTGTTTATTTAGTCAG  594
                  ||||||| |||||| ||||||||||||  ||| || ||||| ||||||||||| ||
Sbjct  36354  CGCAGAAGAAAAGTACGTGGAACATTGTATCCAAACTAATCAGCCGGTTTATTTAGTGAG  36295

Query  595    GGACGGAGTAACAAAGTGTATCCCGATAGTTTGTGCAGAAGATGTCCTCaaaaaaaaGCC  654
               |||||  | || ||||| || ||  |||| |||||||||||||||||| |||| |
Sbjct  36294  AGACGGGATCACTAAGTGCATTCCCGTAGTGTGTGCAGAAGATGTCCTCCAAAAGAGCAT  36235

Query  655    GGAAAACCCGTCAAACATCTCCGGAGACGGAAATGCTTTCGAATTTGTCGGAAGTAGACA  714
               |||    |||||| ||||||| |||||||||||||||||||| || ||  ||| ||| |
Sbjct  36234  AGAAGGTACGTCAAGCATCTCCAGAGACGGAAATGCTTTCGAAATTATCCCAAGCAGAGA  36175

Query  715    AACAGAAAAAAGTCCATATCTTGGAGAAGGATCCTCCGGAGCCTCAAGCGGGTCCATCCC  774
              |||     ||||||| |  || ||||  || |  |||||| ||   |||||||||||||
Sbjct  36174  AACCCGGGAAAGTCCCTTCCTCGGAGCCGGCTGTTCCGGAACCAGTAGCGGGTCCATCCG  36115

Query  775    TCGAGATGACGGAAACAAGGAAGCAGGAAATTCAGGACTTTATCGATCGATCTCCCCTTT  834
              | || ||| |||||||   |||||| |  || || || ||||||||||  |||||| | |
Sbjct  36114  TGGAAATGTCGGAAACTCAGAAGCAAGTGATCCAAGATTTTATCGATCAGTCTCCC-TGT  36056

Query  835    TGACAGCGATACAGGATTACTCCATGGAACACCCCCTCGGCCTTCTTCCGTTGCCTCGAC  894
              || ||||| | ||||||| | |  ||||||| || || || ||  | ||  | || || |
Sbjct  36055  TGGCAGCGCTTCAGGATTTCACTTTGGAACATCCGCTGGGTCTATTACCCCTCCCCCGTC  35996

Query  895    TGATGTCCGAGTTCGTAGGCGTAATCGAAGCAAACGGATTGACGAATATTTCTCAGGAGC  954
              ||||||| |||||||||||  |||| |||    ||| ||||||||||||| | |  || |
Sbjct  35995  TGATGTCGGAGTTCGTAGGGTTAATAGAAAGCGACGCATTGACGAATATTACACCAGAAC  35936

Query  955    AGAAAGTTCTTTGGCGGGAAGCTCAAATAGCTACCAAGAAAGTTTTGAAACCGTTTCTTT  1014
              |||| | ||| ||||||||||| ||||| ||||| ||||||||||||||||| |||||||
Sbjct  35935  AGAAGGCTCTATGGCGGGAAGCACAAATTGCTACGAAGAAAGTTTTGAAACCCTTTCTTT  35876

Query  1015   CGAAAAATTTAAAAGCTTATTCTGTGCAGAATTTGGCCGACTGGATAATCACCGAAGGTA  1074
              | || ||||||||  | | |||||||||||||||||| || |||||||| ||||| ||||
Sbjct  35875  CAAAGAATTTAAAGACCTTTTCTGTGCAGAATTTGGCAGAATGGATAATAACCGACGGTA  35816

Query  1075   CATATTACACGACATTTACCGAAATACAATTTCCGGGCGCTTTCGGGAAAATGATAGGGG  1134
              ||| || |||||||||||| ||||| |  || |||| ||| |||  ||||||||| ||||
Sbjct  35815  CATGTTGCACGACATTTACAGAAATCCGTTTCCCGGCCGCCTTCTCGAAAATGATCGGGG  35756

Query  1135   AGTCGTACCCAAGGACACAGGCAACTCATCATCGGCATGGACATTCGTCGTCTTCCAGCA  1194
              | ||||||||||||||||||||||||| ||  || | ||  | |||||||||||| ||||
Sbjct  35755  ACTCGTACCCAAGGACACAGGCAACTCGTCGGCGTCGTGATCGTTCGTCGTCTTCGAGCA  35696

Query  1195   CGGCCAGCATGTCCACGTCATCCACAAGTGCCGATACGAAAGGTCGTGGTGCTCGTGCGC  1254
               ||  ||||| ||||||||||||||  ||||||||||||| | || |||||||| |||||
Sbjct  35695  TGGAAAGCATATCCACGTCATCCACTTGTGCCGATACGAACGATCTTGGTGCTCATGCGC  35636

Query  1255   ACTCACGAAGAAAGCGGAAGAGCGATTCGGACGACGAATTGGAAGACATACTTTACGAGG  1314
               ||||| |||||||||||| | ||  |||||||| ||||||||||| ||||| | ||| |
Sbjct  35635  GCTCACAAAGAAAGCGGAAAAACGGCTCGGACGAAGAATTGGAAGATATACTCTTCGAAG  35576

Query  1315   CAGTAGGATCACAGAAGGCCACCTCGAACATCTCGCAAATTATATACAAAAAGGGCGACG  1374
              ||||||| |||||||| ||||||||  | || |||||||||||||||||||||||| | |
Sbjct  35575  CAGTAGGCTCACAGAAAGCCACCTCCGAAATATCGCAAATTATATACAAAAAGGGCAAAG  35516

Query  1375   CAGGCTCCTTCAAGCTTTCCTCGCCGGGCGAGAAAGGCGATTATCTCGTCAAGCTGGACT  1434
               ||| | ||||||||||| ||| || || ||||||||||||||  |  |||| || ||||
Sbjct  35515  AAGGTTGCTTCAAGCTTTTCTCTCCCGGAGAGAAAGGCGATTACATGATCAAACTCGACT  35456

Query  1435   TTTACAAGACACGCAATCTTCGCAGAGTGGCAAAGGAGAACTGGTGGAAGGAGGCCGATT  1494
              |||||||||  || |||||  | ||||| || ||||| || |||| || |||||| || |
Sbjct  35455  TTTACAAGATTCGGAATCTGAGGAGAGTAGCGAAGGATAATTGGTCGAGGGAGGCGGACT  35396

Query  1495   ACCGGAGCACATTTCTTCTTTCTTACGCGGAACCTGCCGCAAA-CAGCATCaaaaaaaTC  1553
              |||| | ||| ||  | ||||||||| |||| || || || |  ||| || || || ||
Sbjct  35395  ACCGAACCACGTTCGTGCTTTCTTACACGGATCCGGCAGCCACGCAG-ATAAAGAAGATA  35337

Query  1554   CTGAAAGATCAAGTGAAGAAGATAGCGAAAATTCCGGAGAGGAAGGTGGAGTACCTGCCC  1613
              |||||||| || || |||||||||||    ||||||||  ||||| | || |   | |||
Sbjct  35336  CTGAAAGAGCAGGTCAAGAAGATAGCAGCGATTCCGGATCGGAAGATCGAATTTTTACCC  35277

Query  1614   CAACAAAAAGGGAGAAAATAATTCAATTTCT-TCAGTCGTGCGTAATAACTCCTTTTTCT  1672
               |||||||||||| ||||||||  ||||| | ||| || |||||||| || || ||||||
Sbjct  35276  AAACAAAAAGGGAAAAAATAATCGAATTTTTATCA-TCTTGCGTAATTACCCCGTTTTCT  35218

Query  1673   CGAATTTTTCAATCCCAATTGTACTTATTCGATAAATCTTTATATTTTATTACACCTGAA  1732
              || ||||| |||||| | || ||  | |||||||||||||||||||||||||| || |||
Sbjct  35217  CGTATTTTCCAATCCGATTTATATCTGTTCGATAAATCTTTATATTTTATTACGCCCGAA  35158

Query  1733   TCTATGTCTTGTCAGCTTGCCATTTT  1758
              || |||||||||||| | || |||||
Sbjct  35157  TCAATGTCTTGTCAGTTAGCAATTTT  35132
```

The protein(s) encoded in the integrated sequence (Rep endonuclease motifs)

```
ORF8          1 MSVHLSDDNSSENSFSIIDTDSSDFVQVEQLSPAEEKYVEHCIQTNQPVY     50
                ||.||||||||::|||:||||||:|||||||||:||:||||||.||||||
BtaDV_NS1     1 MSAHLSDDNSSQDSFSVIDTDSSEFVQVEQLSPSEERYVEHCIATNQPVY     50

ORF8         51 LVRDGITKCIPVVCAEDVLQKSIEGTSSISRDGNAFEIIPSRETRESPFL    100
                |||||:|||||:|||||||:|..|..|:||.||||||.:.||:|.:||:|
BtaDV_NS1    51 LVRDGVTKCIPIVCAEDVLKKKPENPSNISGDGNAFEFVGSRQTEKSPYL    100

ORF8        101 GAGCSGTSSGSIRGNVGNSEASDPRFYRSVSLLAALQDFTLEHPLGLLPL    150
                |.|.||.|||||..:.||.||.:...|||:|...:  |..|.|  |..|.
BtaDV_NS1   101 GEGSSGASSGSIPRDDGNKEAGNSGLYRSISPFDS--DTGLLH--GTPPR    146

ORF8        151 PRLMSEFVGLIESDALTNITPEQKALWREAQIATKKVLKPFLSKNLKTFS    200
                |          .|.|.|::                :|.:...||.:..:
BtaDV_NS1   147 P----------SSVASTDV----------------RVRRRNRSKRIDEY-    169


ORF27         1 ----------------------------------------------SFVV      4
                                                              :|||
BtaDV_NS1   201 CAEFGRLDNHRRYILHDIYRNTISGRFRENDRGVVPKDTGNSSSAWTFVV    250

ORF27         5 FEHGKHIHVIHLCRYERSWCSCALTKKAEKRLGRRIGRYTLRSSRLTESH     54
                |:||:|:||||.|||||||||||||||||:|.||||||:|||.||:||.|
BtaDV_NS1   251 FQHGQHVHVIHKCRYERSWCSCALTKKAEERFGRRIGRHTLRGSRITEGH    300

ORF27        55 LRNIANYIQKGQRRLLQAFLSRRERRLHDQTRLLQDSESEESSEG-----     99
                |.::|||||||:||||||||:.|||||..|..||||::|.:|.:|
BtaDV_NS1   301 LEHLANYIQKGRRRLLQAFLAGRERRLSRQAGLLQDTQSSQSGKGELVEG    350


ORF28         1 ----------------------------------------------MVEG      4
                                                              :|||
BtaDV_NS1   301 LEHLANYIQKGRRRLLQAFLAGRERRLSRQAGLLQDTQSSQSGKGELVEG    350

ORF28         5 GGLPNHVRAFLHGSGSHADKEDTERAGQEDSSDSGSEDRIFTQTKREKII     54
                |.||.|:.:||.|:.....:::.||:.:|||.:||.|..:...|||||||
BtaDV_NS1   351 GRLPEHISSFLRGTCRKQHQKNPERSSEEDSENSGEEGGVPAPTKREKII    400

ORF28        55 EFLSSCVITPFSRIFQSDLYLFDKSLYFITPESMSCQLAI----------     94
                :||.|||||||||||||.||||||||||||||||||||||
BtaDV_NS1   401 QFLQSCVITPFSRIFQSQLYLFDKSLYFITPESMSCQLAILQHTHTINKM    450
```

Bemisia tabaci isolate AsiaII6 CTG434_PILON_PILON_PILON_PILON, whole genome shotgun sequence

```
Sequence ID: VMEG01002263.1Length: 947840 Number of Matches: 1
Range 1: 479851 to 480270
Identities      Gaps           Strand
288/423(68%)    6/423(1%)      Plus/Minus

Query  1967    ATTCTTGATAAGCGAATTCC--AAAAGTTAATACTTTGTTAATTGCTGGTCCTGCTAATT  2024
               ||||| |||||| || | |   |||||  |||||||| || |||  ||| ||| ||||||
Sbjct  480270  ATTCTGGATAAG-GAGTACATGAAAAGA-AATACTTTATTGATTCTTGGACCTCCTAATT  480213

Query  2025    CAGGAAAAAAttttttttttGATGCAGTGACACAAGCTATGATAAATTGTGGTCACGTCG  2084
               ||||||||||||| ||||| |||    ||   | |||   | | ||||| || ||| ||| ||
Sbjct  480212  CAGGAAAAAAATTTCTTTTTCGATTGTGTTTCTCAATTTTTAATAAACTGCGGTTACGCCG  480153

Query  2085    GAAATTTTAACAGATTTTGTAACTTTCCATTACAAGAGTGCAACATGAAAAGGATCTTAT  2144
               | |||  ||| ||||||    || ||||| || ||||| |||    |  |||||  |  |
Sbjct  480152  GTAATAGTAATAGATTTCAAAATTTTCCGTTTCAAGAATGCGTAAAAAAAAGATTAATTG  480093

Query  2145    TATGGAATGAACCGGTTTGCGAACCATCCGCGGTGGAAACTATAAAAATGCTTTTCGCTG  2204
               | ||||| ||||||    || ||| | || ||  | |||  | ||||||    | ||||| |
Sbjct  480092  TGTGGAACGAACCGCAGTGTGAAACTTCAGCTTTTGAAGATGTAAAAAAATTGTTCGCAG  480033

Query  2205    GGGACCCTTGCCCCGCAAAAATTAAATACAAAAATGATGCGACAATCATGCGCACTCCCG  2264
               | || || |||    | |||||||||||| ||||  ||||| | | |  || | |||||
Sbjct  480032  GAGATCCGTGCAATGTAAAAATTAAATATAAAATGGATGCAATAGTTCTGAGAACTCCAT  479973

Query  2265    TAATAGTTCTAACGAACAACAATCCTTTCCCTAATGACCCGCCATTTAATAGTAGAATGA  2324
               | || ||||||    || ||| |    ||| ||| ||||      ||||||  ||||| ||
Sbjct  479972  TTATTGTTCTATGTAATAACGACATTTTTCCTGATGATAAAGCATTTAGAAGTAGGATTT  479913

Query  2325    TTAAATACACATGGCGACAGTCACCTTTCTTGGCAGAAATTCAT-AAAAAACCTCACCCT  2383
                ||||| |  ||||  | |  ||  ||| ||   || | | | | ||||||||||||||
Sbjct  479912  GTAAATCCGAATGGAAATACGCAGATTT-TTTAAAGTACTGCTTGAAAAAACCTCACCCG  479854

Query  2384    TTA  2386
               |||
Sbjct  479853  TTA  479851
```

The protein encoded in the integrated sequence (SF3 helicase Parvo NS1)

```
ORF6              1 ----------ILDKEYMKRNTLLILGPPNSGKNFFFDCVSQFLINCGYAG     40
                              ||||...|.|||||.||.|||||||||.|:|.:||||:.|
BtaDV_NS1       501 FVNFLENLLNILDKRIPKVNTLLIAGPANSGKNFFFDAVTQAMINCGHVG     550

ORF6             41 NSNRFQNFPFQECVKKRLIVWNEPQCETSAFEDVKKLFAGDPCNVKIKYK     90
                    |.|||.|||.|||..||:::||||.||.||.|.:|.||||||||..|||||
BtaDV_NS1       551 NFNRFCNFPLQECNMKRILLWNEPVCEPSAVETIKMLFAGDPCPAKIKYK     600

ORF6             91 MDAIVLRTPFIVLCNNDIFPDDKAFRSRICKSEWKYADFLKYCLKKPHPL     140
                    .||.::|||.|||.||:.||:|..|.||:.|..|:.:.||....||||||
BtaDV_NS1       601 NDATIMRTPVIVLTNNNPFPNDPPFNSRMIKYTWRQSPFLAEIHKKPHPL     650

ORF6            141 --------------------------------------------------     140

BtaDV_NS1       651 ALFYVLAKYNLIDTLELDNFDSSLLQDLSVTTFIQEATHDIDSSIRSTEA     700
```

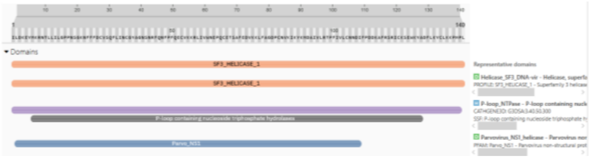

BtaDV-related EVEs in the genome of *B. tabaci* AsiaII7 pooled male and female from China (BioProject PRJNA553780)

Descriptions | Graphic Summary | Alignments

Sequences producing significant alignments — Download — Manage columns — Show 100

select all *6 sequences selected* — GenBank · Graphics · Distance tree of results · MSA Viewer

Description	Max Score	Total Score	Query Cover	E value	Per. Ident	Acc. Len	Accession
Bemisia tabaci isolate AsiaII7 CTG162_PILON_PILON_PILON_PILON	1110	1110	27%	0.0	77.95%	1485075	VLTD01000847.1
Bemisia tabaci isolate AsiaII7 CTG393_PILON_PILON_PILON_PILON	128	175	10%	1e-26	67.22%	725614	VLTD01001896.1
Bemisia tabaci isolate AsiaII7 CTG30_PILON_PILON_PILON_PILON	73.4	73.4	2%	2e-10	74.81%	3481354	VLTD01001557.1
Bemisia tabaci isolate AsiaII7 CTG156_PILON_PILON_PILON_PILON	58.1	58.1	6%	2e-05	65.16%	1514610	VLTD01000808.1
Bemisia tabaci isolate AsiaII7 CTG113_PILON_PILON_PILON_PILON	47.3	47.3	4%	0.034	67.02%	1897303	VLTD01000353.1
Bemisia tabaci isolate AsiaII7 CTG702_PILON_PILON_PILON_PILON	46.4	46.4	3%	0.034	67.83%	133293	VLTD01003060.1

Distribution of the top 7 Blast Hits on 6 subject sequences

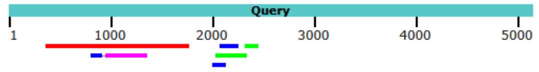

Bemisia tabaci isolate AsiaII7 CTG162_PILON_PILON_PILON_PILON, whole genome shotgun sequence

```
Sequence ID: VLTD01000847.1Length: 1485075Number of Matches: 1
Range 1: 1432692 to 1434089GenBankGraphics
Next Match
Previous Match
Alignment statistics for match #1 Score        Expect          Identities      Gaps            Strand
1110 bits(1230)                 0.0            1096/1406(78%)                  13/1406(0%)     Plus/Plus

Query  358      TGATAAGAGTTTAAACAAG-GTAGGTAAATAAAAGGTGGCTCGAGTGCGGGATTCGTCGC  416
                |||||| ||  |||||||  ||| |||||| |||||||    |||| | ||||     ||
Sbjct  1432692  TGATAAAAGAGTAAACAACCGTATGTAAATGAAAGGTG---CGAGCGAGGGAACGATCAT  1432748

Query  417      AGTTC--AGtttttttGCCGGTATGTCTGCTCATTTAAGCGACGATAACAGTTCTCAAGA  474
                |  ||  |  |||||||  | ||||||||  ||| ||   ||||||||||| ||  || |
Sbjct  1432749  ATATCGTATGTTTTTTG--GATATGTCTGTGCATCTATCTGACGATAACAGCTCGGAAAA  1432806

Query  475      TTCATTTTCAGTGATAGATACAGATAGTAGTGAATTCGTGCAAGTTGAACAATTATCCCC  534
                ||| |||||| | |||||||| |||  |||||| || |||||||||||||| ||||| ||
Sbjct  1432807  TTCGTTTTCAATTATAGATACCGATTCTAGTGATTTTGTGCAAGTTGAACAGTTATCTCC  1432866

Query  535      TAGTGAAGAAAGGTACGTTGAACATTGTATCGCAACAAACCAGCCTGTTTATTTAGTCAG  594
                    ||||||| |||||| ||||||||||||  ||| || ||||| ||||||||||| ||
Sbjct  1432867  CGCAGAAGAAAAGTACGTGGAACATTGTATCCAAACTAATCAGCCGGTTTATTTAGTGAG  1432926

Query  595      GGACGGAGTAACAAAGTGTATCCCGATAGTTTGTGCAGAAGATGTCCTCaaaaaaaaGCC  654
                 |||||  | || ||||| || ||  |||| ||||||||||||||||| |||| |
Sbjct  1432927  AGACGGGATCACTAAGTGCATTCCCGTAGTGTGTGCAGAAGATGTCCTCCAAAAGAGCAT  1432986

Query  655      GGAAAACCCGTCAAACATCTCCGGAGACGGAAATGCTTTCGAATTTGTCGGAAGTAGACA  714
                 |||    |||||| ||||||| |||||||||||||||||| || ||  ||| ||| |
Sbjct  1432987  AGAAGGTACGTCAAGCATCTCCAGAGACGGAAATGCTTTCGAAATTATCCCAAGCAGAGA  1433046

Query  715      AACAGAAAAAAGTCCATATCTTGGAGAAGGATCCTCCGGAGCCTCAAGCGGGTCCATCCC  774
                |||     ||||||| |  || ||||  || |  |||||| ||   |||||||||||||
Sbjct  1433047  AACCCGGGAAAGTCCCTTCCTCGGAGCCGGCTGTTCCGGAACCAGTAGCGGGTCCATCCG  1433106
```

```
Query  775      TCGAGATGACGGAAACAAGGAAGCAGGAAATTCAGGACTTTATCGATCGATCTCCCCTTT  834
                | || ||| |||||||   |||||| |  || || || ||||||||||  |||||| | |
Sbjct  1433107  TGGAAATGTCGGAAACTCAGAAGCAAGTGATCCAAGATTTTATCGATCAGTCTCCC-TGT  1433165

Query  835      TGACAGCGATACAGGATTACTCCATGGAACACCCCCTCGGCCTTCTTCCGTTGCCTCGAC  894
                || ||||| | ||||||| | |  ||||||| || || || ||  | ||  | || || |
Sbjct  1433166  TGGCAGCGCTTCAGGATTTCACTTTGGAACATCCGCTGGGTCTATTACCCCTCCCCCGTC  1433225

Query  895      TGATGTCCGAGTTCGTAGGCGTAATCGAAGCAAACGGATTGACGAATATTTCTCAGGAGC  954
                ||||||| |||||||||||  |||| |||    ||| ||||||||||||| | |  || |
Sbjct  1433226  TGATGTCGGAGTTCGTAGGGTTAATAGAAAGCGACGCATTGACGAATATTACACCAGAAC  1433285

Query  955      AGAAAGTTCTTTGGCGGGAAGCTCAAATAGCTACCAAGAAAGTTTTGAAACCGTTTCTTT  1014
                |||| | ||| ||||||||||| ||||| ||||| ||||||||||||||||| |||||||
Sbjct  1433286  AGAAGGCTCTATGGCGGGAAGCACAAATTGCTACGAAGAAAGTTTTGAAACCCTTTCTTT  1433345

Query  1015     CGAAAAATTTAAAAGCTTATTCTGTGCAGAATTTGGCCGACTGGATAATCACCGAAGGTA  1074
                | || ||||||||  | | |||||||||||||||||| || |||||||| ||||| ||||
Sbjct  1433346  CAAAGAATTTAAAGACCTTTTCTGTGCAGAATTTGGCAGAATGGATAATAACCGACGGTA  1433405

Query  1075     CATATTACACGACATTTACCGAAATACAATTTCCGGGCGCTTTCGGGAAAATGATAGGGG  1134
                ||| || |||||||||||| ||||| |  || |||| ||| |||  ||||||||| ||||
Sbjct  1433406  CATGTTGCACGACATTTACAGAAATCCGTTTCCCGGCCGCCTTCTCGAAAATGATCGGGG  1433465

Query  1135     AGTCGTACCCAAGGACACAGGCAACTCATCATCGGCATGGACATTCGTCGTCTTCCAGCA  1194
                | ||||||||||||||||||||||||| ||  || | ||  | |||||||||||| ||||
Sbjct  1433466  ACTCGTACCCAAGGACACAGGCAACTCGTCGGCGTCGTGATCGTTCGTCGTCTTCGAGCA  1433525

Query  1195     CGGCCAGCATGTCCACGTCATCCACAAGTGCCGATACGAAAGGTCGTGGTGCTCGTGCGC  1254
                 ||  ||||| ||||||||||||||  ||||||||||||| | || |||||||| |||||
Sbjct  1433526  TGGAAAGCATATCCACGTCATCCACTTGTGCCGATACGAACGATCTTGGTGCTCATGCGC  1433585

Query  1255     ACTCACGAAGAAAGCGGAAGAGCGATTCGGACGACGAATTGGAAGACATACTTTACGAGG  1314
                 ||||| |||||||||||| | ||  |||||||| ||||||||||| ||||| | ||| |
Sbjct  1433586  GCTCACAAAGAAAGCGGAAAAACGGCTCGGACGAAGAATTGGAAGATATACTCTTCGAAG  1433645

Query  1315     CAGTAGGATCACAGAAGGCCACCTCGAACATCTCGCAAATTATATACAAAAAGGGCGACG  1374
                ||||||| |||||||| ||||||||  | || |||||||||||||||||||||||| | |
Sbjct  1433646  CAGTAGGCTCACAGAAAGCCACCTCCGAAATATCGCAAATTATATACAAAAAGGGCAAAG  1433705

Query  1375     CAGGCTCCTTCAAGCTTTCCTCGCCGGGCGAGAAAGGCGATTATCTCGTCAAGCTGGACT  1434
                 ||| | ||||||||||| ||| || || ||||||||||||||  |  |||| || ||||
Sbjct  1433706  AAGGTTGCTTCAAGCTTTTCTCTCCCGGAGAGAAAGGCGATTACATGATCAAACTCGACT  1433765

Query  1435     TTTACAAGACACGCAATCTTCGCAGAGTGGCAAAGGAGAACTGGTGGAAGGAGGCCGATT  1494
                |||||||||  || |||||  | ||||| || ||||| || |||| || |||||| || |
Sbjct  1433766  TTTACAAGATTCGGAATCTGAGGAGAGTAGCGAAGGATAATTGGTCGAGGGAGGCGGACT  1433825

Query  1495     ACCGGAGCACATTTCTTCTTTCTTACGCGGAACCTGCCG-CAAACAGCATCaaaaaaaTC  1553
                |||| | ||| ||  | ||||||||| |||| || || | ||  ||| || || || ||
Sbjct  1433826  ACCGAACCACGTTCGTGCTTTCTTACACGGATCCGGCAGCCACGCAG-ATAAAGAAGATA  1433884

Query  1554     CTGAAAGATCAAGTGAAGAAGATAGCGAAAATTCCGGAGAGGAAGGTGGAGTACCTGCCC  1613
                |||||||| || || |||||||||||    ||||||||  ||||| | || |   | |||
Sbjct  1433885  CTGAAAGAGCAGGTCAAGAAGATAGCAGCGATTCCGGATCGGAAGATCGAATTTTTACCC  1433944

Query  1614     CAACAAAAAGGGAGAAAATAATTCAATTTCT-TCAGTCGTGCGTAATAACTCCTTTTTCT  1672
                 |||||||||||| ||||||||  ||||| | ||| || |||||||| || || ||||||
Sbjct  1433945  AAACAAAAAGGGAAAAAATAATCGAATTTTTATCA-TCTTGCGTAATTACCCCGTTTTCT  1434003

Query  1673     CGAATTTTTCAATCCCAATTGTACTTATTCGATAAATCTTTATATTTATTACACCTGAA  1732
                || ||||| |||||| | || ||  | |||||||||||||||||||||||||| || |||
Sbjct  1434004  CGTATTTTCCAATCCGATTTATATCTGTTCGATAAATCTTTATATTTATTACGCCCGAA  1434063

Query  1733     TCTATGTCTTGTCAGCTTGCCATTTT  1758
                || |||||||||||| | || |||||
Sbjct  1434064  TCAATGTCTTGTCAGTTAGCAATTTT  1434089
```

The protein(s) encoded in the integrated sequence (Rep endonuclease motifs)

```
ORF3              1 MSVHLSDDNSSENSFSIIDTDSSDFVQVEQLSPAEEKYVEHCIQTNQPVY     50
                    ||.||||||||::|||:||||||:|||||||||:||:||||||.||||||
BtaDV_NS1         1 MSAHLSDDNSSQDSFSVIDTDSSEFVQVEQLSPSEERYVEHCIATNQPVY     50

ORF3             51 LVRDGITKCIPVVCAEDVLQKSIEGTSSISRDGNAFEIIPSRETRESPFL    100
                    |||||:|||||:|||||||:|..|..|:||.||||||.:.||:|.:||:|
BtaDV_NS1        51 LVRDGVTKCIPIVCAEDVLKKKPENPSNISGDGNAFEFVGSRQTEKSPYL    100

ORF3            101 GAGCSGTSSGSIRGNVGNSEASDPRFYRSVSLLAALQDFTLEHPLGLLPL    150
                    |.|.||.|||||..:.||.||.:...|||:|...:  |..|.|  |..|.
BtaDV_NS1       101 GEGSSGASSGSIPRDDGNKEAGNSGLYRSISPFDS--DTGLLH--GTPPR    146


ORF9              1 ----------------------SKILSISLPVGSASGFHFGTSAGSIT     26
                                          ....||| |..|.:|...||.....:
BtaDV_NS1       101 GEGSSGASSGSIPRDDGNKEAGNSGLYRSIS-PFDSDTGLLHGTPPRPSS    149

ORF9             27 PPPSDVGVRRVNRKRRIDEYYTRTEGSMAGSTNCYEESFETLSFKEFKDL     76
                    ...:||.|||.||.:|||||::..|.|:|||:|.|:|||||:||::||.|
BtaDV_NS1       150 VASTDVRVRRRNRSKRIDEYFSGAESSLAGSSNSYQESFETVSFEKFKSL    199

ORF9             77 FCAEFGRMDNNRRYMLHDIYRNPFPGRLLENDRGLVPKDTGNSSAS----    122
                    |||||||:||:|||:|||||||...||..|||||:|||||||||::
BtaDV_NS1       200 FCAEFGRLDNHRRYILHDIYRNTISGRFRENDRGVVPKDTGNSSSAWTFV    249


ORF10             1 ----------------------------------------------SFVV      4
                                                                  :|||
BtaDV_NS1       201 CAEFGRLDNHRRYILHDIYRNTISGRFRENDRGVVPKDTGNSSSAWTFVV    250

ORF10             5 FEHGKHIHVIHLCRYERSWCSCALTKKAEKRLGRRIGRYTLRSSRLTESH     54
                    |:||:|:||||.||||||||||||||||:|.||||||:|||.||:||.|
BtaDV_NS1       251 FQHGQHVHVIHKCRYERSWCSCALTKKAEERFGRRIGRHTLRGSRITEGH    300

ORF10            55 LRNIANYIQKGQRRLLQAFLSRRERRLHDQTRLLQDSESEESSEG-----     99
                    |.::|||||||:||||||||:.|||||..|..||||::|.:|.:|
BtaDV_NS1       301 LEHLANYIQKGRRRLLQAFLAGRERRLSRQAGLLQDTQSSQSGKGELVEG    350
```

BtaDV-related EVEs in the genome of *B. tabaci* Asia1 pooled male and female from China (BioProject PRJNA553782)

Descriptions | Graphic Summary | Alignments

Sequences producing significant alignments — Download — Manage columns — Show 100

select all *5 sequences selected* — GenBank Graphics Distance tree of results MSA Viewer

Description	Max Score	Total Score	Query Cover	E value	Per. Ident	Acc. Len	Accession
Bemisia tabaci isolate Asia1 CTG376_PILON_PILON_PILON_PILON	113	113	5%	3e-22	70.49%	324121	VMEI01001666.1
Bemisia tabaci isolate Asia1 CTG82_PILON_PILON_PILON_PILON	80.6	80.6	3%	2e-12	72.66%	5307194	VMEI01002994.1
Bemisia tabaci isolate Asia1 CTG224_PILON_PILON_PILON_PILON	74.3	74.3	2%	3e-10	73.85%	2895224	VMEI01001104.1
Bemisia tabaci isolate Asia1 CTG286_PILON_PILON_PILON_PILON	52.7	105	6%	9e-04	68.39%	394193	VMEI01001351.1
Bemisia tabaci isolate Asia1 CTG18_PILON_PILON_PILON_PILON	46.4	46.4	0%	0.036	100.00%	2769033	VMEI01000939.1

Distribution of the top 6 Blast Hits on 5 subject sequences

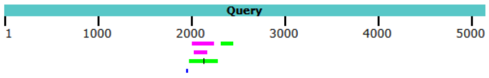

Bemisia tabaci isolate Asia1 CTG376_PILON_PILON_PILON_PILON, whole genome shotgun sequence

```
Sequence ID: VMEI01001666.1Length: 324121 Number of Matches: 1
Range 1: 143292 to 143534
Identities      Gaps            Strand
172/244(70%)    1/244(0%)       Plus/Plus

Query  1994    AATACTTTGTTAATTGCTGGTCCTGCTAATTCAGGAAAAAAtttttttttGATGCAGTG  2053
               ||||| ||  ||||||  || |||   ||||||||||||| |||||||     ||   ||
Sbjct  143292  AATACATTACTAATTGTAGGCCCTCAGAATTCAGGAAAAAAATTTTTTTCGACTGT-GTT  143350

Query  2054    ACACAAGCTATGATAAATTGTGGTCACGTCGGAAATTTTAACAGATTTTGTAACTTTCCA  2113
                |||||  |||||| ||||| ||  | |  ||||| |  || ||||||   || |||||
Sbjct  143351  TCACAATTTATGATTAATTGCGGATATGCTGGAAACTCAAATAGATTTCAGAATTTTCCG  143410

Query  2114    TTACAAGAGTGCAACATGAAAAGGATCTTATTATGGAATGAACCGGTTTGCGAACCATCC  2173
               || ||||||||    |  |||||  |  || | ||||| ||||||   || ||| ||||
Sbjct  143411  TTTCAAGAGTGTGTTAAAAAAAGATTAATAGTTTGGAACGAACCGCAGTGTGAAACATCA  143470

Query  2174    GCGGTGGAAACTATAAAAATGCTTTTCGCTGGGGACCCTTGCCCCGCAAAAATTAAATAC  2233
               ||  | |||  | ||||||  ||||||||||| || |||||     | ||||||||||||
Sbjct  143471  GCTTTCGAAGATGTAAAAAAACTTTTCGCTGGAGATCCTTGTAATGTAAAAATTAAATAT  143530

Query  2234    AAAA  2237
               ||||
Sbjct  143531  AAAA  143534
```

The protein encoded in the integrated sequence (SF3 helicase Parvo NS1)

```
ORF1            1 -------------------YITNCRPSEFRKKIFFDCVSQFMINCGYAG     30
                                     .:....|:...|..|||.|:|.|||||:.|
BtaDV_NS1     501 FVNFLENLLNILDKRIPKVNTLLIAGPANSGKNFFFDAVTQAMINCGHVG    550

ORF1           31 NSNRFQNFPFQECVKKRLIVWNEPQCETSAFEDVKKLFAGDPCNVKIKYK     80
                  |.|||.|||.|||..||:::||||.||.||.|.:|.||||||||..|||||
BtaDV_NS1     551 NFNRFCNFPLQECNMKRILLWNEPVCEPSAVETIKMLFAGDPCPAKIKYK    600

ORF1           81 --------------------------------------------------     80

BtaDV_NS1     601 NDATIMRTPVIVLTNNNPFPNDPPFNSRMIKYTWRQSPFLAEIHKKPHPL    650
```

BtaDV-related EVEs in the genome of *B. tabaci* SSA1 whole body adult from Tanzania (BioProject PRJNA418329)

Descriptions | Graphic Summary | Alignments

Sequences producing significant alignments Download Manage columns Show 100

select all *5 sequences selected* GenBank Graphics Distance tree of results MSA Viewer

	Description	Max Score	Total Score	Query Cover	E value	Per. Ident	Acc. Len	Accession
☑	Bemisia tabaci isolate SSA1 scaffold1088	996	996	28%	0.0	75.40%	237694	PGTP01000808.1
☑	Bemisia tabaci isolate SSA1 scaffold8482	318	318	8%	3e-84	77.67%	70856	PGTP01004674.1
☑	Bemisia tabaci isolate SSA1 scaffold223	298	383	14%	3e-78	72.20%	196188	PGTP01000195.1
☑	Bemisia tabaci isolate SSA1 scaffold482	101	101	6%	2e-18	67.99%	3397063	PGTP01002155.1
☑	Bemisia tabaci isolate SSA1 scaffold9552	64.4	64.4	5%	1e-07	66.27%	163386	PGTP01070670.1

Distribution of the top 6 Blast Hits on 5 subject sequences

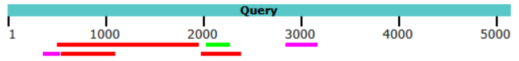

Bemisia tabaci isolate SSA1 scaffold1088, whole genome shotgun sequence

```
Sequence ID: PGTP01000808.1Length: 237694 Number of Matches: 1
Range 1: 169650 to 171091
Identities      Gaps            Strand
1091/1447(75%)  10/1447(0%)     Plus/Plus

Query  502     TAGTGAATTCGTGCAAGTTGAACAATTATCCCCTAGTGAAGAAAGGTACGTTGAACATTG  561
               |||||| || |||||||| || || ||||| ||     ||||||| ||||||||||||||
Sbjct  169650  TAGTGATTTTGTGCAAGTGGATCAGTTATCTCCAGCGGAAGAAAAGTACGTTGAACATTG  169709

Query  562     TATCGCAACAAACCAGCCTGTTTATTTAGTCAGGGACGGAGTAACAAAGTGTATCCCGAT  621
                ||   ||| || |||||||| ||||||||  | ||||| || || ||||| |||||   |
Sbjct  169710  CATACAAACCAATCAGCCTGTGTATTTAGTGCGCGACGGTGTGACTAAGTGCATCCCCGT  169769

Query  622     AGTTTGTGCAGAAGATGTCCTCaaaaa----aaaGCCGGAAAACCCGTCAAACATCTCCG  677
               ||| |||||||||||||||||| ||||     ||||  || |     ||  |    ||| |||
Sbjct  169770  AGTGTGTGCAGAAGATGTCCTCGAAAAGAGTAAAGAAGGTA----CGCAACGTATCGCCG  169825

Query  678     GAGACGGAAATGCTTTCGAATTTGTCGGAAGTAGACAAACAGAAAAAAGTCCATATCTTG  737
               || |||| |||||||||||||||||| ||    || || |      | ||||| |  || |
Sbjct  169826  GAAACGGCAATGCTTTCGAATTTGTCGCAAACCGAGAAGCCAGGGACAGTCCGTTCCTCG  169885

Query  738     GAGAAGGATCCTCCGGAGCCTCAAGCGGGTCCATCCCTCGAGATGACGGAAACAAGGAAG  797
               |||  || |  |||||| ||      ||||||| ||  | || || ||||||      ||||
Sbjct  169886  GAGCCGGTTGTTCCGGAACCAGTTCCGGGTCCGTCGTTGGAAATAACGGAAGAGCAGAAG  169945

Query  798     CAGGAAATTCAGGACTTTATCGATCGATCTCCCCTTTTGACAGCGATACAGGATTACTCC  857
               |||  ||| || || ||||||||||  |||||||| ||||||||| | ||||||||| |
Sbjct  169946  CAGATAATACAAGATTTTATCGATCAGTCTCCCCTGTTGACAGCGTTTCAGGATTACACT  170005

Query  858     ATGGAACACCCCCTCGGCCTTCTTCCGTTGCCTCGACTGATGTCCGAGTTCGTAGGCGTA  917
                ||| ||| ||  | || ||  | ||  | || || |||||| |||||||||| ||  ||
Sbjct  170006  CTGGGACATCCGTTGGGTCTATTACCCCTCCCCCGTCTGATGGCCGAGTTCGTGGGGTTA  170065

Query  918     ATCGAAGCAAACGGATTGACGAATATTTCTCAGGAGCAGAAAGTTCTTTGGCGGGAAGCT  977
                | |||    ||| ||||||||||||| | || || ||||| | ||| |||||||||||
Sbjct  170066  GTAGAAAGCGACGCATTGACGAATATTACGCAAGAACAGAAGGCTCTGTGGCGGGAAGCA  170125

Query  978     CAAATAGCTACCAAGAAAGTTTTGAAACCGTTTCTTTCGAAAAATTTAAAAGCTTATTCT  1037
               ||||| ||||| |||| |||||||||||| ||||||||||||||||||||   |||||||
Sbjct  170126  CAAATTGCTACGAAGAGAGTTTTGAAACCATTTCTTTCGAAAAATTTAAAGATTTATTCT  170185

Query  1038    GTGCAGAATTTGGCCGACTGGATAATCACCGAAGGTACATATTACACGACATTTACCGAA  1097
               ||||||||||| || |  |||||  |||| || |||||||  | ||||| ||||||| ||
Sbjct  170186  GTGCAGAATTTAGCAGGGTGGATTCTCACAGACGGTACATGCTGCACGATATTTACCCAA  170245

Query  1098    ATACAATTTCCGGGCGCTTTCGGGAAAATGATAGGGGAGTCGTACCCAAGGACACAGGCA  1157
               || |   | |||| ||| ||   ||||||||| ||||| |||||||||||  |||||| |
Sbjct  170246  ATCCGCGTCCCGGCCGCCTTTCTGAAAATGATCGGGGAATCGTACCCAAGAGCACAGGGA  170305

Query  1158    ACTCATCATCGGCATGGACATTCGTCGTCTTCCAGCACGGCCAGCATGTCCACGTCATCC  1217
               |||| ||  || | ||| |||||||||| ||| |||||||| |||| ||||| |||||||
Sbjct  170306  ACTCGTCGGCGTCGTGGTCATTCGTCGTGTTCGAGCACGGCAAGCACGTCCATGTCATCC  170365

Query  1218    ACAAGTGCCGATACGAAAGGTCGTGGTGCTCGTGCGCACTCACGAAGAAAGCGGAAGAGC  1277
               ||  |||| ||||||| || || ||||||||||||||| ||||| || ||||| ||||
Sbjct  170366  ACCTGTGCAGATACGAGAGATCATGGTGCTCGTGCGCGCTCACAAAAAAAGCAGAAGGAA  170425

Query  1278    GATTCGGACGACGAATTGGAAGACATACTTTACGAGGCAGTAGGATCACAGAAGGCCACC  1337
               | ||||||||| ||||||||||| ||||| | ||| || ||||| |||||||| |||||
Sbjct  170426  GGTTCGGACGAAGAATTGGAAGATATACTCTTCGAAGCCGTAGGCTCACAGAAAGCCACA  170485

Query  1338    TCGAACATCTCGCAAATTATATACAAAAAGGGCGACGCAGGCTCCTTCAAGCTTTCCTCG  1397
               ||     || |||||| ||||||||||||||||  | | ||||| ||||||||||| ||||
Sbjct  170486  TCCGCAATATCGCAAGTTATATACAAAAAGGGAAAAGAAGGCTGCTTCAAGCTTTTCTCG  170545
```

```
Query  1398    CCGGGCGAGAAAGGCGATTATCTCGTCAAGCTGGACTTTTACAAGACACGCAATCTTCGC  1457
               || || ||||||||||| ||  |  ||||||| || || |||  ||| ||  | ||  |
Sbjct  170546  CCAGGAGAGAAAGGCGAATACATGATCAAGCTCGATTTCTACCGGACCCGTCAGCTGAGG  170605

Query  1458    AGAGTGGCAAAGGAGAACTGGTGGAAGGAGGCCGATTACCGGAGCACATTTCTTCTTTCT  1517
               |||||||| ||||| || ||||||| ||| || || |||||   ||| ||  | ||||||
Sbjct  170606  AGAGTGGCGAAGGACAATTGGTGGAGGGAAGCGGACTACCGAGCCACGTTCGTGCTTTCT  170665

Query  1518    TACGCGGAACCTGCCGCAA-ACAGCATCaaaaaaaTCCTGAAAGATCAAGTGAAGAAGAT  1576
               ||| |||| ||||| ||||  ||| || || || ||||| || || || || ||||||||
Sbjct  170666  TACACGGATCCTGCAGCAACTCAG-ATAAAGAAGATCCTCAAGGAGCAGGTCAAGAAGAT  170724

Query  1577    AGCGAAAATTCCGGAGAGGAAGGTGGAGTACCTGCCCCAACAAAAAGGGAGAAAATAATT  1636
               |||    ||||||||  ||||| | || |   | ||  ||||||||||| ||||| |||
Sbjct  170725  AGCAGCGATTCCGGATCGGAAGATCGAATTTTTACCAAAACAAAAAGGGAAAAAATGATT  170784

Query  1637    CAATTTCTTCAGTCGTGCGTAATAACTCCTTTTTCTCGAATTTTTCAATCCCAATTGTAC  1696
                ||||| |    || |||||||| || ||||||||||| ||||||||||| | |||||
Sbjct  170785  GAATTTTTATCCTCATGCGTAATTACCCCTTTTTCTCGTATTTTTCAATCCGAGTTGTAA  170844

Query  1697    TTATTCGATAAATCTTTATATTTTATTACACCTGAATCTATGTCTTGTCAGCTTGCCATT  1756
                  ||||||||||| |||||||  ||||| || ||||| ||| | |||||| | || ||
Sbjct  170845  CGTTTCGATAAATCGTTATATTACATTACCCCCGAATCCATGGCCTGTCAGTTAGCGATA  170904

Query  1757    TTACAGCACACACATACGATTAACAAGATGACTGTGCAACAAATATTTCAGTACCAGTTA  1816
                | || ||||| || || ||||| ||||||| |||   | ||| |||||||||| || |||
Sbjct  170905  ATGCAACACACTCACACCATTAATAAGATGAGTGTAAATCAATTATTTCAGTATCAATTA  170964

Query  1817    AATATTCCTTTCTCAAAATTGTTTTATAATTGCGATAATCCTGAGGATTATTTTTTGAAT  1876
               || ||||| || || ||||| ||||| |||||   |   ||| || || |||||||| ||
Sbjct  170965  AACATTCCGTTTCTAAATTATTTTACAATTGTAACGCTCCGGATGAATATTTTTTAAAC  171024

Query  1877    TTATCTGCTAGCATCCTTTGCATTGAAACTTTATTGAAACACCAGGTTGGCAGTTTCGGA  1936
               ||    || || ||  | || |||||||| || ||||| || || || || || | |||
Sbjct  171025  TTGGAAGCGAGTATTTTGTGTATTGAAACATTGTTGAAGCATCAAGTCGGAAGCTACGGT  171084

Query  1937    TTCGTAA  1943
               |||| ||
Sbjct  171085  TTCGGAA  171091
```

The protein(s) encoded in the integrated sequence (Rep endonuclease motifs)

```
ORF5             1 ---------------------SDFVQVDQLSPAEEKYVEHCIQTNQPVY     28
                                        |:||||:||||:||:||||||.||||||
BtaDV_NS1        1 MSAHLSDDNSSQDSFSVIDTDSSEFVQVEQLSPSEERYVEHCIATNQPVY     50

ORF5            29 LVRDGVTKCIPVVCAEDVLEKSKEGTQRIAGNGNAFEFVANREARDSPFL     78
                   |||||||||||:|||||||:|..|....|:|:|||||||.:|:...||:|
BtaDV_NS1       51 LVRDGVTKCIPIVCAEDVLKKKPENPSNISGDGNAFEFVGSRQTEKSPYL    100

ORF5            79 GAGCSGTSSGSVVGNNGRAEADNTRFYRSVSPVDSVSGLHSGTSVGSITP    128
                   |.|.||.||||:..::|..||.|:..|||:||.||.:||..||     .|
BtaDV_NS1      101 GEGSSGASSGSIPRDDGNKEAGNSGLYRSISPFDSDTGLLHGT-----PP    145

ORF5           129 PPS-----DGRVRGVSRKRRIDEYYARTEGSVAGSTNCYEESFETISFEK    173
                   .||     |.|||..:|.:||||||::..|.|:|||:|.|:|||||:||||
BtaDV_NS1      146 RPSSVASTDVRVRRRNRSKRIDEYFSGAESSLAGSSNSYQESFETVSFEK    195

ORF5           174 FKDLFCAEFSRVDSHRRYMLHDIYPNPRPGRLSENDRGIVPKSTGNSSAS    223
                   ||.||||||.|:|:||||:|||||.|...||..|||||:|||.||||::
BtaDV_NS1      196 FKSLFCAEFGRLDNHRRYILHDIYRNTISGRFRENDRGVVPKDTGNSSSA    245

ORF5           224 WSFVVFEHGKHVHVIHLCRYERSWCSCALTKKAEGRFGRRIGRYTLRSRR    273
                   |:||||:||:||||||.||||||||||||||||.||||||||:|||..|
BtaDV_NS1      246 WTFVVFQHGQHVHVIHKCRYERSWCSCALTKKAEERFGRRIGRHTLRGSR    295

ORF5           274 LTESHIRNIASYIQKGKRRLLQAFLARRERRIHDQARFLPDPSAEESGEG    323
                   :||.|:.::|:|||||:|||||||||.||||:..||..|.|..:.:||:|
BtaDV_NS1      296 ITEGHLEHLANYIQKGRRRLLQAFLAGRERRLSRQAGLLQDTQSSQSGKG    345

ORF5           324 QLVEGSGLPSHVRAFLHGSCSNSDKEDPQGAGQEDSSDSGSEDRIFTKTK    373
                   :||||..||.|:.:||.|:|....:::|:.:.:|||.:||.|..:...||
BtaDV_NS1      346 ELVEGGRLPEHISSFLRGTCRKQHQKNPERSSEEDSENSGEEGGVPAPTK    395

ORF5           374 REKMIEFLSSCVITPFSRIFQSEL-------------------------    397
                   |||:|:||.|||||||||||||:|
BtaDV_NS1      396 REKIIQFLQSCVITPFSRIFQSQLYLFDKSLYFITPESMSCQLAILQHTH    445
```

Bemisia tabaci isolate SSA1 scaffold8482, whole genome shotgun sequence

Sequence ID: PGTP01004674.1Length: 70856 Number of Matches: 1
Range 1: 29232 to 29641

Identities	Gaps	Strand
320/412(78%)	3/412(0%)	Plus/Minus

```
Query  1969   TCTT-GATAAGCGAATTCCAAAAGTTAATACTTTGTTAATTGCTGGTCCTGCTAATTCAG  2027
              |||| |||||||||||||| ||||| |||||   | ||   | |   ||||| || ||||| |
Sbjct  29641  TCTTAGATAAGCGAATTCCTAAAGTAAATACAATCTTGGTCGAAGGTCCGGCAAATTCGG  29582

Query  2028   GAAAAAAtttttttttGATGCAGTGACACAAGCTATGATAAATTGTGGTCACGTCGGAA  2087
              | ||||| |||||||| ||||| || || | |||||||||||| || || ||| ||||||
Sbjct  29581  GTAAAAACTTTTTTTT-GATGCGGTTACCCTAGCTATGATAAACTGCGGGCACATCGGAA  29523

Query  2088   ATTTTAACAGATTTTGTAACTTTCCATTACAAGAGTGCAACATGAAAAGGATCTTATTAT  2147
              |||||||  | ||||| |  ||||| || || || || ||||||||| | |||||  | |
Sbjct  29522  ATTTTAATCGTTTTTGCAGTTTTCCTTTGCAGGAATGTAACATGAAACGTATCTTGATGT  29463

Query  2148   GGAATGAACCGGTTTGCGAACCATCCGCGGTGGAAACTATAAAAATGCTTTTCGCTGGGG  2207
              |||||||||| | ||||||||| || ||||||||||| || |||||||| || || || |
Sbjct  29462  GGAATGAACCCGCTTGCGAACCTTCGGCGGTGGAAACAATTAAAATGCTCTTTGCAGGTG  29403

Query  2208   ACCCTTGCCCCGCAAAAATTAAATACAAAAATGATGCGACAATCATGCGCACTCCCGTAA  2267
              | || ||||| || |||||||| ||| |||| || || ||||||||| | || ||  | |
Sbjct  29402  ATCCATGCCCAGCGAAAATTAAGTAC-AAAACGACGCAACAATCATGAGAACACCAATTA  29344

Query  2268   TAGTTCTAACGAACAACAATCCTTTCCCTAATGACCCGCCATTTAATAGTAGAATGATTA  2327
              | ||| |||| ||||| |||||||| || |||||  |||| |||||||| |||||||| |
Sbjct  29343  TTGTTTTAACTAACAATAATCCTTTTCCCAATGATGCGCCTTTTAATAGCAGAATGATAA  29284

Query  2328   AATACACATGGCGACAGTCACCTTTCTTGGCAGAAATTCATAAAAAAACCTCA  2379
              | || || ||  | ||| | ||||| || || || ||| | |||||||| ||
Sbjct  29283  AGTATACTTGAAGGCAGGCGCCTTTTTAGCGGATATTTACAAAAAAACCGCA  29232
```

The protein encoded in the integrated sequence (SF3 helicase Parvo NS1)

```
ORF10            1 -----------LDKRIPKVNTILVEGPANSGKNFFLMRLP----------     29
                              ||||||||||:|:.||||||||||...:.
BtaDV_NS1      501 FVNFLENLLNILDKRIPKVNTLLIAGPANSGKNFFFDAVTQAMINCGHVG    550


ORF9             1 --------------------------------KLFFDAVTLAMINCGHIG     18
                                                   ..||||||.|||||||:|
BtaDV_NS1      501 FVNFLENLLNILDKRIPKVNTLLIAGPANSGKNFFFDAVTQAMINCGHVG    550

ORF9            19 NFNRFCSFPLQECNMKRILMWNEPACEPSAVETIKMLFAGDPCPAKIKYK     68
                   ||||||:||||||||||||:||||.|||||||||||||||||||||||||
BtaDV_NS1      551 NFNRFCNFPLQECNMKRILLWNEPVCEPSAVETIKMLFAGDPCPAKIKYK    600

ORF9            69 TTQQS---------------------------------------------     73
                   .....
BtaDV_NS1      601 NDATIMRTPVIVLTNNNPFPNDPPFNSRMIKYTWRQSPFLAEIHKKPHPL    650


ORF8             1 ------------------------------------------------VQ      2
                                                                   .:
BtaDV_NS1      551 NFNRFCNFPLQECNMKRILLWNEPVCEPSAVETIKMLFAGDPCPAKIKYK    600

ORF8             3 NDATIMRTPIIVLTNNNPFPNDAPFNSRMIKYT-----------------     35
                   |||||||||:||||||||||||.||||||||||
BtaDV_NS1      601 NDATIMRTPVIVLTNNNPFPNDPPFNSRMIKYTWRQSPFLAEIHKKPHPL    650
```

Bemisia tabaci isolate SSA1 scaffold482, whole genome shotgun sequence

```
Sequence ID: PGTP01002155.1Length: 3397063 Number of Matches: 1
Range 1: 1445016 to 1445338
Identities      Gaps            Strand
223/328(68%)    10/328(3%)      Plus/Minus

Query  2830     ACGCGCTTCTTGCCAACTGAGAGTCCGTCATG-CAAAGGCACCGTGTCGTGGTAGTAGAT  2888
                ||| | |||||||||| ||| |  || |  || || |||    || |||| |||| | |
Sbjct  1445338  ACGAGTTTCTTGCCAAGTGATAAACC-TGTTGACAGAGGGTTAGTTTCGTCGTAGAACAA  1445280

Query  2889     AGTGATATCGTCATTCCACATAACAGCGTCAGTACCACCAGTAAACGCAGAGTCAACATT  2948
                 |||| ||| ||||||||||||||||    || |||  ||||| ||||||| |||||||
Sbjct  1445279  TGTGACATCATCATTCCACATAACAG---GAGCACCTTCAGTATACGCAGAATCAACATG  1445223

Query  2949     ACTGCTTATCTCAATATAACACTCCACTTCAAAGTATGCCGCTGAATTCTGAAATGA---  3005
                |     ||  |  ||  ||||   ||||||||| || |||||||| |||||| | ||
Sbjct  1445222  ATAAGATAATTTTATTGAACAAGACACTTCAAAATAAGCCGCTGAGTTCTGATAAGAGTG  1445163

Query  3006     AACACTTGAGTTTGCAGGATTAATGTTTGGCGTCGGCATGAGGCCTATATGTACTTGAGG  3065
                | || | || |  || ||||| |  ||||| ||||| |  |  |||||||| |  |||||
Sbjct  1445162  AGCATTCGACTGAGCTGGATTTAAATTTGGAGTCGGGAGTAAACCTATATGAAGCTGAGG  1445103

Query  3066     TTGAATATTTTCACCACCTGCGGCCTTG-AAATTGTGAGAGTAAGGACCTGACTCTAACA  3124
                ||| | ||| | ||  |    |   ||| ||||| |     | |||||| || |||| ||
Sbjct  1445102  TTGCACATTGT-ACTGCGATGGAAATTGAAAATTATTCATATTAGGACCAGATTCTATCA  1445044

Query  3125     TGCGATTATATTTGCACTCAAATGGAAC  3152
                ||||||||||  |    | |||||||||
Sbjct  1445043  TGCGATTATACGTCTCTTTAAATGGAAC  1445016
```

BtaDV-related EVEs in the genome of *B. tabaci* SSA1-SG1 population from Uganda (BioProject: PRJEB28507)

Descriptions | Graphic Summary | Alignments

Sequences producing significant alignments — Download — Manage columns — Show 100

select all — *6 sequences selected* — GenBank — Graphics — Distance tree of results — MSA Viewer

	Description	Max Score	Total Score	Query Cover	E value	Per. Ident	Acc. Len	Accession
☑	Bemisia tabaci strain SSA1-SG1 Uganda F8 genome assembly, contig: CONTIG63	524	524	16%	5e-146	74.22%	14051705	CADEAZ010000004.1
☑	Bemisia tabaci strain SSA1-SG1 Uganda F8 genome assembly, contig: CONTIG124	318	318	8%	4e-84	77.67%	10992293	CADEAZ010000007.1
☑	Bemisia tabaci strain SSA1-SG1 Uganda F8 genome assembly, contig: CONTIG20	108	108	8%	1e-20	66.51%	1952563	CADEAZ010000076.1
☑	Bemisia tabaci strain SSA1-SG1 Uganda F8 genome assembly, contig: CONTIG255	97.8	97.8	5%	2e-17	68.91%	6632645	CADEAZ010000013.1
☑	Bemisia tabaci strain SSA1-SG1 Uganda F8 genome assembly, contig: CONTIG473	61.7	61.7	2%	2e-06	73.87%	4922878	CADEAZ010000028.1
☑	Bemisia tabaci strain SSA1-SG1 Uganda F8 genome assembly, contig: CONTIG297	50.9	50.9	2%	0.003	73.75%	3179661	CADEAZ010000050.1

Distribution of the top 6 Blast Hits on 6 subject sequences

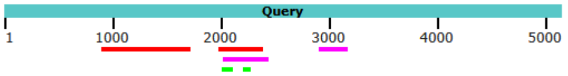

Bemisia tabaci strain SSA1-SG1 Uganda F8 genome assembly, contig: CONTIG63, whole genome shotgun sequence

```
Sequence ID: CADEAZ010000004.1 Length: 14051705 Number of Matches: 1
Identities      Gaps            Strand
616/830(74%)    15/830(1%)      Plus/Plus

Query  888      CCTCGACTGATGTCCGAGTTCGTAGGCGTAATCGAAGCAAACGGATTGACGAATATTTCT  947
                ||| | |||||| | || |   ||||  ||||||||    |||||||||| |||||| |
Sbjct  680128   CCTTGTCTGATGGCGGAATGGCTAGGAATAATCGAAAACGACGGATTGACCAATATTACC  680187

Query  948      CAGGAGCAGAAAGTTCTTTGGCGGGAAGCTCAAATAGCTACCAAGAAAGTTTTGAAACCG  1007
                |  || ||||| | ||| |||   || |  || || |||   ||   |||| ||| |||
Sbjct  680188   CCCGATCAGAAGGCTCTATGGAAAGAGGGACAGATTGCTTTGAAACGAGTTATGAGACCT  680247

Query  1008     TTTCTTTCGAAAAATTTAAAAGCTTATTCTGTGCAGAATTTGGCCGACTGGATAATCACC  1067
                | ||||||||| ||||||||||||||||||||||  |||||||||| ||  ||||  |
Sbjct  680248   TATCTTTCGAAGAATTTAAAAGCTTATTCTGTCGAGAATTTGGCAGAATGGATCCTGGAA  680307

Query  1068     GAAGGTACATATTACACGACATTTACCGAAATACAATTTCCGGGCGCTTTCGGGAAAATG  1127
                || ||||| |  | |||||||||||  ||||| |  ||| ||| ||| |||  ||| |||
Sbjct  680308   GACGGTACCTGCTCCACGACATTTATAGAAATCCGTTTCCCGGCCGCCTTCTCGAAGATG  680367

Query  1128     ATAGGGGAGTCGTACCCAAGGACACAGGCAACTCATCATCGGCATGGACATTCGTCGTCT  1187
                |||||||| || ||||| |    || ||||||||||| ||| ||||||| |||||| |||
Sbjct  680368   ATAGGGGACTCATACCCCACAGTACGGGCAACTCATCGTCGTCATGGACGTTCGTCATCT  680427

Query  1188     TCCAGCACGGCCAGCATGTCCACGTCATCCACAAGTGCCGATACGAAAGGTCGTGGTGCT  1247
                || | |||||  ||||  | ||||| |||||||  ||| |||||||| ||||||||||||
Sbjct  680428   TCGAACACGGGAAGCACCTGCACGTTATCCACAGATGCAGATACGAACGGTCGTGGTGCT  680487

Query  1248     CGTGCGCACTCACGAAGAAAGCGGAAGAGCGATTCGGACGACGAATTGGAAGACATACTT  1307
                | || ||||||||||||||||||||| ||||  |||||||||||| |||||||||||||
Sbjct  680488   CATGTGCACTCACGAAGAAAGCGGAAAAGCGGCTCGGACGACGAGTTGGAAGACATACTC  680547

Query  1308     TACGAGGCAGTAGGATCACAGAAGGCCACCTCGAACATCTCGCAAATTATATACAAAAAG  1367
                | | | || || || || ||||| ||||||||  | |||||||| ||||| | || ||||
Sbjct  680548   TTCAATGCCGTCGGGTCTCAGAAAGCCACCTCAGAAATCTCGCAGATTATCTTCAGAAAG  680607

Query  1368     GGCGACGCAGGCTCCTTCAAGCTTTCCTCGCCGGGCGAGAAAGGCGATTATCTCGTCAAG  1427
                |||   | ||||| |||||||||||||||||||||||||||| |||||  | || ||
Sbjct  680608   GGCAGAGAAGGCTGCTTCAAGCTTTCCTCGCCGGGCGAGAAAGGAGATTACATGGTGAAA  680667

Query  1428     CTGGACTTTTACAAGACACGCAATCTTCGCAGAGTGGCAAAGGAGAACTGGTGGAAGGAG  1487
                |||||||| ||||||||  |    ||  | ||||| || ||||| | ||||||||| |||
Sbjct  680668   CTGGACTTCTACAAGACGAGGCGGCTGCGGAGAGTAGCCAAGGAAAGCTGGTGGAAAGAG  680727

Query  1488     GCCGATTACCGGAGCACATTTCTTCTTTCTTACGCGGAACCTGCCGCAAACAGC----AT  1543
                || ||||| || |  |||||  | ||||| ||| |||| || |    |||| ||    ||
Sbjct  680728   GCGGATTATCGAACGACATTCTTACTTTCATACACGGACCCAG----AAACTGCGCAAAT  680783

Query  1544     CaaaaaaaTCCTGAAAGATCAAGTGAAGAAGAT---AGCGAAAATTCCGGAGAGGAAGGT  1600
                 || |  || ||||| ||   ||| |||| |||   ||||   || | | |||  |||| ||
Sbjct  680784   AAAGAGGATTCTGAAGGAGGCAGTCAAGAGGATTTCAGCG---ATACAGGATCGGAAAGT  680840

Query  1601     GGAGTACCTGCCCCAACAAAAAGGGAGAAAATAATTCAATTTCTTCAGTCGTGCGTAATA  1660
                ||| | | | ||| | | ||||| || ||||| ||  | ||  |  | ||||| ||  |
Sbjct  680841   GGAATTCATACCCAATCGAAAAGAGA-AAAATGATAGAGTTCTTGGAATCGTGTGTTGTG  680899

Query  1661     ACTCCTTTTTCTCGAATTTTTCAATCCCAATTGTACTTATTCGATAAATC  1710
                || |||||||| || ||||| ||||| || ||||| |  || || |||||
Sbjct  680900   ACCCCTTTTTCCCGCATTTTCCAATCTCATTTGTATTTGTTTGACAAATC  680949
```

The protein encoded in the integrated sequence (Rep endonuclease motifs highlighted)

```
ORF5              1 -LSDGGMARNNRKRRIDQYYPRSEGSMERGTDCFETSYETLSFEEFKSLF     49
                     .:|..:.|.||.:|||:|:..:|.|:...::.::.|:||:|||:|||||
BtaDV_NS1       151 ASTDVRVRRRNRSKRIDEYFSGAESSLAGSSNSYQESFETVSFEKFKSLF    200

ORF5             50 CREFGRMDPGRRYLLHDIYRNPFPGRLLEDDRGLIPHSTGNSSSSWTFVI     99
                    |.||||:|..|||:|||||||...||..|:|||::|..||||||:||||:
BtaDV_NS1       201 CAEFGRLDNHRRYILHDIYRNTISGRFRENDRGVVPKDTGNSSSAWTFVV    250
```

```
ORF5           100 FEHGKHLHVIHRCRYERSWCSCALTKKAEKRLGRRVGRHTLQCRRVSESH   149
                   |:||:|:||||:|||||||||||||||||:|.|||:|||||:..|::|.|
BtaDV_NS1      251 FQHGQHVHVIHKCRYERSWCSCALTKKAEERFGRRIGRHTLRGSRITEGH   300

ORF5           150 LRNLADYLQKGQRRLLQAFLAGRERRLHGETGLLQDEAAAESSQGKLVER   199
                   |.:||:|:|||:||||||||||||||..:.|||||..:::|.:|:|||.
BtaDV_NS1      301 LEHLANYIQKGRRRLLQAFLAGRERRLSRQAGLLQDTQSSQSGKGELVEG   350

ORF5           200 GGLSNDILTFIHGPRNCANKEDSEGGSQEDFSDTGSESGIHTQSKREK--   247
                   |.|...|.:|:.|.....::::.|..|:||..::|.|.|:...:||||
BtaDV_NS1      351 GRLPEHISSFLRGTCRKQHQKNPERSSEEDSENSGEEGGVPAPTKREKII   400
```

Bemisia tabaci strain SSA1-SG1 Uganda F8 genome assembly, contig: CONTIG124, whole genome shotgun sequence

```
Sequence ID: CADEAZ010000007.1Length: 10992293 Number of Matches: 1
Identities      Gaps          Strand
320/412(78%)    3/412(0%)     Plus/Minus

Query  1969      TCTT-GATAAGCGAATTCCAAAAGTTAATACTTTGTTAATTGCTGGTCCTGCTAATTCAG  2027
                 |||| |||||||||||||| ||||| |||||   | ||   | |   ||||| || ||||| |
Sbjct  10097482  TCTTAGATAAGCGAATTCCTAAAGTAAATACAATCTTGGTCGAAGGTCCGGCAAATTCGG  10097423

Query  2028      GAAAAAAtttttttttGATGCAGTGACACAAGCTATGATAAATTGTGGTCACGTCGGAA  2087
                 | ||||| |||||||| ||||| || || | ||||||||||||| || || ||| ||||||
Sbjct  10097422  GTAAAAACTTTTTTTT-GATGCGGTTACCCTAGCTATGATAAACTGCGGGCACATCGGAA  10097364

Query  2088      ATTTTAACAGATTTTGTAACTTTCCATTACAAGAGTGCAACATGAAAAGGATCTTATTAT  2147
                 |||||||  | ||||| |  ||||| || || || || ||||||||| | |||||  | |
Sbjct  10097363  ATTTTAATCGTTTTTGCAGTTTTCCTTTGCAGGAATGTAACATGAAACGTATCTTGATGT  10097304

Query  2148      GGAATGAACCGGTTTGCGAACCATCCGCGGTGGAAACTATAAAAATGCTTTTCGCTGGGG  2207
                 ||||||||||| | ||||||||| || ||||||||||| || |||||||| || || || |
Sbjct  10097303  GGAATGAACCCGCTTGCGAACCTTCGGCGGTGGAAACAATTAAAATGCTCTTTGCAGGTG  10097244

Query  2208      ACCCTTGCCCCGCAAAAATTAAATACAAAAATGATGCGACAATCATGCGCACTCCCGTAA  2267
                 | || ||||| || |||||||| ||| |||| || || ||||||||| | || ||  | |
Sbjct  10097243  ATCCATGCCCAGCGAAAATTAAGTAC-AAAACGACGCAACAATCATGAGAACACCAATTA  10097185

Query  2268      TAGTTCTAACGAACAACAATCCTTTCCCTAATGACCCGCCATTTAATAGTAGAATGATTA  2327
                 | ||| |||| ||||| |||||||| || |||||  |||| |||||||| |||||||| |
Sbjct  10097184  TTGTTTTAACTAACAATAATCCTTTTCCCAATGATGCGCCTTTTAATAGCAGAATGATAA  10097125

Query  2328      AATACACATGGCGACAGTCACCTTTCTTGGCAGAAATTCATAAAAAAACCTCA  2379
                 | || || ||  | ||| | ||||| || || || ||| | ||||||||| ||
Sbjct  10097124  AGTATACTTGAAGGCAGGCGCCTTTTTAGCGGATATTTACAAAAAAACCGCA  10097073
```

```
ORF9             1 -------------------------------KLFFDAVTLAMINCGHIG     18
                                                  ..||||||.|||||||:|
BtaDV_NS1      501 FVNFLENLLNILDKRIPKVNTLLIAGPANSGKNFFFDAVTQAMINCGHVG    550

ORF9            19 NFNRFCSFPLQECNMKRILMWNEPACEPSAVETIKMLFAGDPCPAKIKYK     68
                   ||||||:||||||||||||:||||.||||||||||||||||||||||||||
BtaDV_NS1      551 NFNRFCNFPLQECNMKRILLWNEPVCEPSAVETIKMLFAGDPCPAKIKYK    600

ORF9            69 TTQQS---------------------------------------------     73
                   .....
BtaDV_NS1      601 NDATIMRTPVIVLTNNNPFPNDPPFNSRMIKYTWRQSPFLAEIHKKPHPL    650
```

BtaDV-related EVEs in the genome of *B. tabaci* Sweetpotato F5 population from Uganda (BioProject PRJEB39408)

Descriptions | Graphic Summary | Alignments

Sequences producing significant alignments — Download — Manage columns — Show 100

select all 6 sequences selected — GenBank — Graphics — Distance tree of results — MSA Viewer

Description	Max Score	Total Score	Query Cover	E value	Per. Ident	Acc. Len	Accession
Bemisia tabaci strain Uganda Sweetpotato F5 genome assembly, contig: SCAFFOLD2728	895	895	35%	0.0	71.97%	87476	CAJEWG010002621.1
Bemisia tabaci strain Uganda Sweetpotato F5 genome assembly, contig: SCAFFOLD3135	244	244	12%	7e-62	69.18%	142443	CAJEWG010001601.1
Bemisia tabaci strain Uganda Sweetpotato F5 genome assembly, contig: SCAFFOLD749	151	151	6%	1e-33	71.86%	175788	CAJEWG010001139.1
Bemisia tabaci strain Uganda Sweetpotato F5 genome assembly, contig: SCAFFOLD1439	97.8	97.8	5%	2e-17	69.92%	413723	CAJEWG010000119.1
Bemisia tabaci strain Uganda Sweetpotato F5 genome assembly, contig: SCAFFOLD1447	56.3	56.3	2%	7e-05	72.07%	151609	CAJEWG010001463.1
Bemisia tabaci strain Uganda Sweetpotato F5 genome assembly, contig: SCAFFOLD608	49.1	49.1	1%	0.010	79.63%	535012	CAJEWG010000039.1

Distribution of the top 6 Blast Hits on 6 subject sequences

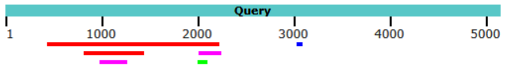

Bemisia tabaci strain Uganda Sweetpotato F5 genome assembly, contig: SCAFFOLD2728, whole genome shotgun sequence

```
Sequence ID: CAJEWG010002621.1 Length: 87476 Number of Matches: 1
Range 1: 59644 to 61422
Identities      Gaps          Strand
1299/1805(72%)  52/1805(2%)   Plus/Plus

Query  436    TATGTCTGCTCATTTAAGCGACGATAACAGTTCTCAAGATTCATTTTCAGTGATAGATAC  495
              |||||| || ||  ||   ||||| | ||||||||| | || ||    || |||||| |
Sbjct  59644  TATGTCGGCGCACCTATCTGACGACGATAGTTCTCAAAACTCTTTCGTGGTAATAGATTC  59703

Query  496    AGAT---AGT---AGTGAATTCGTGCAAGTTGAACAATTATCC-CCTAGTGAAGAAAGGT  548
              |||    |||   ||||| || | ||||| || | ||||||| ||||  ||||||  ||
Sbjct  59704  AGACTCAAGTCTTAGTGATCTCATTCAAGTGGACCCATTATCCTCCTAC-GAAGAACAGT  59762

Query  549    ACGTTGAACATTGTATCGCAACAAACCAGCCTGTTTATTTAGTCAGGGACGGAGTAACAA  608
              ||||| | |||||| | |  ||||| || ||||||||||||     | ||||| ||||||
Sbjct  59763  ACGTTAAGCATTGTCTAGATACAAATCAACCTGTTTATTTAACGCGAGACGGTGTAACAC  59822

Query  609    AGTGTATCCCGATAGTTTGTGCAGAAGATGTCCTCaaaaaaaaGCCGGAAAACCCGTCAA  668
              ||||||| ||  | |||||||| ||| |||||  |||| |||   || | ||   ||||
Sbjct  59823  AGTGTATTCCTGTTGTTTGTGCGGAAAATGTCGACAAAGAAAT--CGAAGAA---GTCAC  59877

Query  669    ACA-----TCTCCGGAGACGGAAATGCTTTCGAATTTGTCGGAAGTAGACAAACAGAAAA  723
              |||     ||||||||  |||| ||| |    || || ||| || | ||         |||
Sbjct  59878  ACAATCAGTCTCCGGAATCGGAGATGTTGCAAAACTTATCGAAAATCGA--------AAA  59929

Query  724    AAGTCCATATCTTGGAGAAGGATCC--TCCGGAGCCTCAAGCGGGTCCATCCCTCGAGAT  781
               | | |  |||  |   || | ||   |||||| ||    || ||||| || |  ||||
Sbjct  59930  GACTTCCGATCGGGCCCAATGCTCGGATCCGGAACCAGTGGCAGGTCCGTCGCAGGAGAA  59989

Query  782    GACGGAAACAAG-GAAGCAGGAAATTCAGGACTTTATCGATCGATCTCCCCTTTTGACAG  840
               | |   | | | ||| || | | ||||||| || || ||   |||||||||  | ||||
Sbjct  59990  AA-GTCCAGAGGAGAAACAAGCAGTTCAGGATTTCATAGACAAATCTCCCCTCCTCACAG  60048

Query  841    CGATACAGGATTACTCCATGGAACACCCCCTCGGCCTTCTTCCGTTGCCTCGACTGATGT  900
              |  | ||||||||  |  |||| || ||     || || |||||| | ||  ||||||||
Sbjct  60049  CCCTGCAGGATTATACTCTGGAGCATCCTTCTGGGCTCCTTCCGATACCGAGACTGATGG  60108

Query  901    CCGAGTTCGTAGGCGTAA-TCGAAGCAAACGGATTGACGAATATTTCTCAGGAGCAGAAA  959
              ||||||| || ||| ||  |   |||   ||| ||||||||||||| |   ||||||  |
Sbjct  60109  CCGAGTTTGTCGGCCTAGCTACCAGCGG-CGGCTTGACGAATATTACGACGGAGCAACAG  60167

Query  960    GTTCTTTGGCGGGAAGCTCAAATAGCTACCAAGAAAGTTTTGAAACCGTTTCTTTCGAAA  1019
              | ||| |||||  |||||||||| || || ||||||||| ||||||||||||||||||
Sbjct  60168  GATCTGTGGCGCCAAGCTCAAATTGCGACAAAGAAAGTTATGAAACCGTTTCTTTCGAAG  60227
```

```
Query  1020   AATTTAAAAGCTTATTCTGTGCAGAATTTGGCCGACTGGATAATCACCGAAGGTACATAT  1079
              || |||||  | | ||||||  |||||| ||| || |||||  ||| ||||  | ||| |
Sbjct  60228  AACTTAAAGACCTTTTCTGTCGAGAATT-GGCAGAGTGGATTCTCAGCGAAAATTCATGT  60286

Query  1080   TACACGACATTTACCGAAATACAATTTCCGGGCGCTTTCGGGAAAATGATAGGGGAGTCG  1139
              ||||||| |||||||  |||    ||  |||  || |||   |||||||||||||| ||
Sbjct  60287  TACACGATATTTACCCGAATCGTGTTGTCGGAGGCATTCTTAAAAATGATAGGGGAATCT  60346

Query  1140   TACCCAAGGACACAGGCAACTCATCATCGGCATGGACATTCGTCGTCTTCCAGCACGGCC  1199
              |||||||  ||||||| |||||  |  || | ||| |  || |||||  | |||||||
Sbjct  60347  TACCCAAAAACACAGGGAACTCGGCGGCGACGTGGTCGATCATCGTCGCCGAGCACGGGA  60406

Query  1200   AGCATGTCCACGTCATCCACAAGTGCCGATACGAAAGGTCGTGGTGCTCGTGCGCACTCA  1259
              | || ||||||| ||||||||| |||| |||||||  |    ||||||||||||||  |||
Sbjct  60407  AACACGTCCACCTCATCCACATGTGCAGATACGACCGAAGCTGGTGCTCGTGCGCGGTCA  60466

Query  1260   CGAAGAAAGC-GGAAGAGCGATTCGGACGACGAATTGGAAGACATACTTTACGAGGCAGT  1318
              |||| || || | ||||  | || ||||||||||||||| || || || |||| |||||
Sbjct  60467  CGAAAAAGGCAGAAAGAATGCTT-GGACGACGAATTGGACGATATTCTATACGAAGCAGT  60525

Query  1319   AGGATCACAGAAGGCCACCTCGAACATCTCGCAAATTATATACAAAAAGGGCGACGCAGG  1378
               || || ||  |||||||| || || || || ||||| ||||||||||||  ||  ||
Sbjct  60526  CGGGTCTCAAAAAACCACCTCCAAAATATCCCACATTATTTACAAAAAGGGCAGCGACGG  60585

Query  1379   CTCCTTCAAGCTTTCCTCGCCGGGCGAGAAAGGCGATTATCTCGTCAAGCTGGACTTTTA  1438
               ||||||||||||| ||||||| ||||||||| ||||| ||  | |||||| |||| |||||
Sbjct  60586  ATCCTTCAAGCTTTTCTCGCCCGGCGAGAATGGCGACTACATGGTCAAGTTGGATTTTTA  60645

Query  1439   CAAGACACGCA--ATCTTCGCAGAGTGGCAAAGGAGAACTGGTGGAAGGAGGCCGATTAC  1496
              | ||| |||||  | ||  | || ||| | ||||||||||||||||||||||||| |||
Sbjct  60646  C-AGA-ACGCAACAGCTGAGAAGGGTGCCGAAGGAGAACTGGTGGAAGGAGGCCGACTAC  60703

Query  1497   CGGAGCACATTTCTTCTTTCTTACGCGGAACCTGCCGCAAACAGCATCaaaaaaTCCTG  1556
              || |  || ||  | ||||| || | ||| ||     | |  ||  || || || || ||
Sbjct  60704  CGCACAACTTTCGTACTTTCATATGTGGATCCCCATGTAGCCATGATAAAGAAGATACTA  60763

Query  1557   AAAGATCAAGTGAAGAAGATAGCGAAAATTCCGGAGAGGAAGGTGGAGTACCTGCCCCAA  1616
              || || ||||| |||||  | ||||  ||||||||  ||||| | ||||| ||      ||
Sbjct  60764  AAGGAGCAAGTCAAGAAAGTGGCGATGATTCCGGATCGGAAGATAGAGTATCTCGAAAAA  60823

Query  1617   CAAAAAGGGAGAAAATAATTCAATTTCTTCAGTCGTGCGTAATAACTCCTTTTTCTCGAA  1676
              |||||||||| ||| ||||| | ||| |     || || || ||||| || ||||| || |
Sbjct  60824  CAAAAAGGGAAAAAAGTAATTGATTTTTTGTCTTCATGTGTTATAACACCCTTTTCCCGTA  60883

Query  1677   TTTTTCAATCCCAATTGTACTTATTCGATAAATCTTTATATTTTATTACACCTGAATCTA  1736
              ||||| ||||  | || || || || |||||||| |||| ||  ||| | |||||||  |
Sbjct  60884  TTTTTAAATCAGATTTATATTTGTTTGATAAATCATTATTTTACATTTCTCCTGAATGCA  60943

Query  1737   TGTCTTGTCAGCTTGCCATT-TTACAGCACACACATACGATTAACAAGATGACTGTGCAA  1795
              | ||  ||||  ||| |||| ||||||||||| || || || ||||| |||| |||
Sbjct  60944  TTTCCAGTCA-ATTGGCATTATTACAGCACACGCAAACTATGAACAAAATGAATGTTACT  61002

Query  1796   CAAATATTTCAGTACCAGTTAAATATTCCTTTCTCAAAATTGTTTTATAATTGCGATAAT  1855
              ||| | ||||| || || |||  ||||||| | ||||| || ||||| |||||| || ||
Sbjct  61003  CAACTGTTTCAATATCACTTACGTATTCCTCTATCAAAGTTATTTTACAATTGCAATGAT  61062

Query  1856   CCTGAGGATTATTTTT-TGAATTTATCTGCTAGCATCCTTTGCATTGAAACTTTATTGAA  1914
              || || || ||||||| |  ||| |   || || ||  | ||| |||||||||||||||
Sbjct  61063  CCAGAAGAATATTTTTATCCATTGA--AGCAAGTATTTTATGCCTTGAAACTTTATTGAA  61120

Query  1915   ACACCAGGTTGGCAGTTTCGGATTCGTAAATTTTCTCGAAAATTTGTTAAATATTCTTGA  1974
              ||| ||  |||| |   |   |||     | ||||| | ||| || |  ||||||| |||
Sbjct  61121  ACATCAAATTGGTA-CATATAATTTTACCAATTTCTTGCAAAATTATGTAATATTC-TGA  61178

Query  1975   TAAGCGAATTCCAAAAGTTAATACTTTGTTAATTGCTGGTCCTGCTAATTCAGGAAAAAA  2034
              ||| || ||||||||||| || ||| | ||| | |  || || || ||||| ||||||||
Sbjct  61179  TAAACGCATTCCAAAAGTAAACACTCTTTTAGTGGAAGGCCCAGCAAATTCTGGAAAAAA  61238

Query  2035   tttttttttGATGCAGTGACACAAGCTATGATAAATTGTGGTCACGTCGGAAATTTTAA  2094
              ||| ||||| |||||||| ||  |||| ||| ||||||| ||||| || |||||||||||
Sbjct  61239  TTTCTTTTTCGATGCAGTAACTTAAGCAATGTTAAATTGCGGTCATGTTGGAAATTTTAA  61298

Query  2095   CAGATTTTGTAACTTTCCATTACAAGAGTGCAACATGAAAAGGA-TCTTAT----TATGG  2149
              |||| |||||||||||||  | |||||||||||| | ||| | | ||| ||    | |||
Sbjct  61299  CAGA-TTTGTAACTTTCCTCTTCAAGAGTGCAACTTAAAACGCATTCTAATCAAATGTGG  61357

Query  2150   AATGAACCGGTTTGCGAACCATCCGCGGTGGAAACTATAAAAATGCTTTTCGCTGGGGAC  2209
              |||||||| ||||| ||||| ||||| || |||||  | |||||| | || || || ||
Sbjct  61358  AATGAACCTGTTTGTGAACCTTCCGCTGTTGAAACGGTGAAAATGTTATTTGCGGGAGAT  61417

Query  2210   CCTTG  2214
              |||||
Sbjct  61418  CCTTG  61422
```

The protein encoded in the integrated sequence

```
ORF5             1 MSAHLSDDDSSQNSFVVIDSDSSLSDLIQVDPLSSYEEQYVKHCLDTNQP    50
                   ||||||||:|||:||.|||:||  |:.:||:.||..||:||:||:.||||
BtaDV_NS1        1 MSAHLSDDNSSQDSFSVIDTDS--SEFVQVEQLSPSEERYVEHCIATNQP    48

ORF5            51 VYLTRDGVTQCIPVVCAENVDKEIEEVTQSVSGIGDVAKLIENRKDFRSG   100
                   |||.|||||:|||:||||:|.|:..|...::||.|:..:.:.:|:..:|
BtaDV_NS1       49 VYLVRDGVTKCIPIVCAEDVLKKKPENPSNISGDGNAFEFVGSRQTEKS-    97

ORF5           101 PMLGSGTSGR---SVAGEKSRGETSSSGFHRQISPPHSPAGLYSGASFWA   147
                   |.||.|:||.   |:..:....|..:||.:|.|||..|..||..|.....
BtaDV_NS1       98 PYLGEGSSGASSGSIPRDDGNKEAGNSGLYRSISPFDSDTGLLHGTPPRP   147

ORF5           148 PSDTETDGRVCRPSYQRRLDEYYDGATGSVAPSSNCDKESYETVSFEELK   197
                   .|...||.||.|.:..:|:|||:.||..|:|.|||..:||:|||||||:.|
BtaDV_NS1      148 SSVASTDVRVRRRNRSKRIDEYFSGAESSLAGSSNSYQESFETVSFEKFK   197

ORF5           198 DLFCRELAEWILSENSCYTIFTRIVLSEAFLKMIGESYPKTQGTRRRRGR   247
                   .|||   ||:...:|     ..|.:|.:.:...|        .|..|...|
BtaDV_NS1      198 SLFC---AEFGRLDN-----HRRYILHDIYRNTI-------SGRFRENDR   232


ORF3             1 -----------------------------------------------RPF     3
                                                                  ..|
BtaDV_NS1      151 ASTDVRVRRRNRSKRIDEYFSGAESSLAGSSNSYQESFETVSFEKFKSLF   200

ORF3             4 LSRIGRVDSQRKFMLHDIYPNRVVGGILKNDRGILPKNTGNSAATWSIIV    53
                   .:..||:|:.|:::|||||.|.:.|...:||||::||:||||::.|:.:|
BtaDV_NS1      201 CAEFGRLDNHRRYILHDIYRNTISGRFRENDRGVVPKDTGNSSSAWTFVV   250

ORF3            54 AEHGKHVHLIHMCRYDRSWCSCAVTKKAERMLGRRIGRYSIRSSRVSKNH   103
                   .:||:|||:||.|||:||||||||:|||||...||||||:::|.||:::.|
BtaDV_NS1      251 FQHGQHVHVIHKCRYERSWCSCALTKKAEERFGRRIGRHTLRGSRITEGH   300

ORF3           104 LQNIPHYLQKGQRRILQAFLARREWRLHGQVGFLQNATAEKGAEGELVEG   153
                   |::::.:|:|||:||:||||||.||.||..|.|.||:..:.:..:||||||
BtaDV_NS1      301 LEHLANYIQKGRRRLLQAFLAGRERRLSRQAGLLQDTQSSQSGKGELVEG   350

ORF3           154 GRLPHNFRTFICGSPCSHDKEDTKGASQESGDDSGSEDRVSRKTKREKVI   203
                   ||||.:..:|:.|:.....:::.:.:|:|..::||.|..|...||||||:|
BtaDV_NS1      351 GRLPEHISSFLRGTCRKQHQKNPERSSEEDSENSGEEGGVPAPTKREKII   400

ORF3           204 DFLSSCVITPFSRIFKSDLYLFDKSLFYISPECISSQLALLQHTQTMNKM   253
                   .||.|||||||||||:|.|||||||||::|:||.:|.|||:||||.|:|||
BtaDV_NS1      401 QFLQSCVITPFSRIFQSQLYLFDKSLYFITPESMSCQLAILQHTHTINKM   450

ORF3           254 NVTQLFQYHLRIPLSKLFYNCNDPEEYFYPLKQVFYALKLY---------   294
                   .|.|:|||.|.||.|||||||::||:||..|......::..
BtaDV_NS1      451 TVQQIFQYQLNIPFSKLFYNCDNPEDYFLNLSASILCIETLLKHQVGSFG   500
```

BtaDV-related EVEs in the genome of *B. tabaci* SSA1-SG1 pooled male and female from Nigeria (BioProject PRJEB28507)

Descriptions | Graphic Summary | Alignments

Sequences producing significant alignments — Download — Manage columns — Show 100

select all *5 sequences selected* — GenBank Graphics Distance tree of results MSA Viewer

	Description	Max Score	Total Score	Query Cover	E value	Per. Ident	Acc. Len	Accession
☑	Bemisia tabaci strain SSA1-SG1 Nigeria F8 genome assembly, contig: CONTIG824	313	313	8%	2e-82	77.40%	920676	CADEBA010000113.1
☑	Bemisia tabaci strain SSA1-SG1 Nigeria F8 genome assembly, contig: CONTIG944	97.8	97.8	5%	2e-17	68.91%	569628	CADEBA010000225.1
☑	Bemisia tabaci strain SSA1-SG1 Nigeria F8 genome assembly, contig: CONTIG3390	69.8	69.8	2%	3e-09	77.78%	814547	CADEBA010000141.1
☑	Bemisia tabaci strain SSA1-SG1 Nigeria F8 genome assembly, contig: CONTIG582	64.4	64.4	5%	1e-07	66.27%	545742	CADEBA010000237.1
☑	Bemisia tabaci strain SSA1-SG1 Nigeria F8 genome assembly, contig: CONTIG1271	55.4	55.4	2%	7e-05	73.33%	4147067	CADEBA010000006.1

Distribution of the top 5 Blast Hits on 5 subject sequences

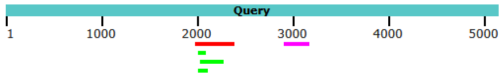

Bemisia tabaci strain SSA1-SG1 Nigeria F8 genome assembly, contig: CONTIG824, whole genome shotgun sequence

```
Sequence ID: CADEBA010000113.1 Length: 920676 Number of Matches: 1
Range 1: 729067 to 729471
Identities      Gaps          Strand
315/407(77%)    2/407(0%)     Plus/Minus

Query  1973    GATAAGCGAATTCCAAAAGTTAATACTTTGTTAATTGCTGGTCCTGCTAATTCAGGAAAA  2032
               |||||||||||||| ||||| |||||  | ||    |  ||||| || ||||| || |||
Sbjct  729471  GATAAGCGAATTCCTAAAGTAAATACAATCTTGGGCGAAGGTCCGGCAAATTCGGGTAAA  729412

Query  2033    AAttttttttttGATGCAGTGACACAAGCTATGATAAATTGTGGTCACGTCGGAAATTTT  2092
               || |||||||| ||||| || || | |||||||||||| || || ||| ||||||||||
Sbjct  729411  AACTTTTTTTT-GATGCGGTTACCCTAGCTATGATAAACTGCGGGCACATCGGAAATTTT  729353

Query  2093    AACAGATTTTGTAACTTTCCATTACAAGAGTGCAACATGAAAAGGATCTTATTATGGAAT  2152
               ||  | ||||| |  ||||| || || || || ||||||||| | |||||  | ||||||
Sbjct  729352  AATCGTTTTTGCAGTTTTCCTTTGCAGGAATGTAACATGAAACGTATCTTGATGTGGAAT  729293

Query  2153    GAACCGGTTTGCGAACCATCCGCGGTGGAAACTATAAAAATGCTTTTCGCTGGGGACCCT  2212
               ||||| | ||||||||| || ||||||||||| || |||||||| || || || || ||
Sbjct  729292  GAACCCGCTTGCGAACCTTCGGCGGTGGAAACAATTAAAATGCTCTTTGCAGGTGATCCA  729233

Query  2213    TGCCCCGCAAAAATTAAATACAAAAATGATGCGACAATCATGCGCACTCCCGTAATAGTT  2272
               ||||| || |||||||| ||| |||| || || ||||||||| | || ||  | || |||
Sbjct  729232  TGCCCAGCGAAAATTAAGTAC-AAAACGACGCAACAATCATGAGAACACCAATTATTGTT  729174

Query  2273    CTAACGAACAACAATCCTTTCCCTAATGACCCGCCATTTAATAGTAGAATGATTAAATAC  2332
                |||| ||||| |||||||| || |||||  |||| |||||||| |||||||| || ||
Sbjct  729173  TTAACTAACAATAATCCTTTTCCCAATGATGCGCCTTTTAATAGCAGAATGATAAAGTAT  729114

Query  2333    ACATGGCGACAGTCACCTTTCTTGGCAGAAATTCATAAAAAACCTCA  2379
               || ||  | ||| | ||||| || || || ||| | |||||||| ||
Sbjct  729113  ACTTGAAGGCAGGCGCCTTTTTAGCGGATATTTACAAAAAACCGCA  729067
```

Bemisia tabaci strain SSA1-SG1 Nigeria F8 genome assembly, contig: CONTIG944, whole genome shotgun sequence

```
Sequence ID: CADEBA010000225.1Length: 569628 Number of Matches: 1
Range 1: 484304 to
Identities      Gaps          Strand
184/267(69%)    8/267(2%)     Plus/Minus

Query  2890    GTGATATCGTCATTCCACATAACAGCGTCAGTACCACCAGTAAACGCAGAGTCAACATTA  2949
               |||| ||| ||||||||||||||||    || |||  ||||| ||||||| ||||||| |
Sbjct  484566  GTGACATCATCATTCCACATAACAG---GAGCACCTTCAGTATACGCAGAATCAACATGA  484510

Query  2950    CTGCTTATCTCAATATAACACTCCACTTCAAAGTATGCCGCTGAATTCTGAAATGA---A  3006
                    ||  || ||  ||||   ||||||||| || |||||||| |||||| | ||   |
Sbjct  484509  TAAGATAATTCTATTGAACAAGACACTTCAAAATAAGCCGCTGAGTTCTGATAAGAGTGA  484450

Query  3007    ACACTTGAGTTTGCAGGATTAATGTTTGGCGTCGGCATGAGGCCTATATGTACTTGAGGT  3066
                || | || |  || ||||| |  ||||| ||||| |  |  |||||||| |  ||||||
Sbjct  484449  GCATTCGACTGAGCTGGATTTAAATTTGGAGTCGGGAGTAAACCTATATGAAGCTGAGGT  484390

Query  3067    TGAATATTTTCACCACCTGCGGCCTTG-AAATTGTGAGAGTAAGGACCTGACTCTAACAT  3125
               || | ||| | ||  |    |   ||| ||||| |      | |||||| || |||| |||
Sbjct  484389  TGCACATTGT-ACTGCGATGGAAATTGAAAATTATTCATATTAGGACCAGATTCTATCAT  484331

Query  3126    GCGATTATATTTGCACTCAAATGGAAC  3152
               |||||||||  |    | |||||||||
Sbjct  484330  GCGATTATACGTCTCTTTAAATGGAAC  484304
```

The protein encoded in the integrated sequence, homologous to VP

```
ORF1            1 ----------------------------------VPFKETYNRMIESGP    15
                                                    |||:..||||:||||
BtaDV_VP      551 PWNINIDGWASDVDHDKNKGRAYYKSVNSATSDMYVPFECKYNRMLESGP   600

ORF1           16 NMNNFQFPSQYNVQPQLHIGLLPTPNLNPAQSNAHSYQNSAAYFEVSCSI    65
                  ..:||:.....|:|||:||||:||||:|||.|:. |:||||||||||.|.|
BtaDV_VP      601 YSHNFKAAGGENIQPQVHIGLMPTPNINPANSSV-SFQNSAAYFEVECYI   649

ORF1           66 ELSYHVDSAYTEGAP-VMWNDDV--------------------------    87
                  |:|.:||||:|.|.. ||||||:
BtaDV_VP      650 EISSNVDSAFTGGTDAVMWNDDITIYYHDTVPLHDGLSVGKKRVKPSSAS   699
```

BtaDV-related EVEs in the genome of *B. tabaci* SSA3 population from Nigeria (BioProject: PRJEB35304)

Descriptions | Graphic Summary | Alignments

Sequences producing significant alignments — Download — Manage columns — Show 100

select all *6 sequences selected* — GenBank Graphics Distance tree of results MSA Viewer

	Description	Max Score	Total Score	Query Cover	E value	Per. Ident	Acc. Len	Accession
☑	Bemisia tabaci strain SSA3 Nigeria F8 genome assembly, contig: CONTIG1136	880	1725	25%	0.0	75.80%	4288651	CAJEWH010000031.1
☑	Bemisia tabaci strain SSA3 Nigeria F8 genome assembly, contig: CONTIG157	289	365	14%	2e-75	71.84%	5733838	CAJEWH010000020.1
☑	Bemisia tabaci strain SSA3 Nigeria F8 genome assembly, contig: CONTIG934	92.4	92.4	6%	1e-15	67.38%	1937629	CAJEWH010000061.1
☑	Bemisia tabaci strain SSA3 Nigeria F8 genome assembly, contig: CONTIG1439	76.1	76.1	4%	7e-11	69.05%	265405	CAJEWH010000378.1
☑	Bemisia tabaci strain SSA3 Nigeria F8 genome assembly, contig: CONTIG678	66.2	66.2	5%	4e-08	66.13%	1531938	CAJEWH010000075.1
☑	Bemisia tabaci strain SSA3 Nigeria F8 genome assembly, contig: CONTIG876	57.2	57.2	2%	2e-05	75.56%	949618	CAJEWH010000108.1

Distribution of the top 8 Blast Hits on 6 subject sequences

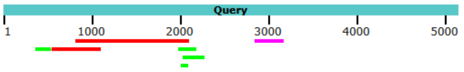

Bemisia tabaci strain SSA3 Nigeria F8 genome assembly, contig: CONTIG1136, whole genome shotgun sequence

```
Sequence ID: CAJEWH010000031.1Length: 4288651 Number of Matches: 2
Range 1: 4094806 to 4096064
Identities      Gaps            Strand
974/1285(76%)   30/1285(2%)     Plus/Minus

Query  814      TTATCGATCGATCTCCCCTTTTGACAGCGATACAGGATTACTCCATGGAACACCCCCTCG  873
                |||||||||  |||||||| ||||||||  | ||||||||| |  ||||||| ||  | |
Sbjct  4096064  TTATCGATCAGTCTCCCCTGTTGACAGCTTTTCAGGATTACACTCTGGAACATCCGTTGG  4096005

Query  874      GCCTTCTTCCGTTGCCTCGACTGATGTCCGAGTTCGTAGGCGTAATCGAAGCAAACGGAT  933
                | ||  | ||  | ||||| |||||| |||||||||| ||  || | |||    |||  |
Sbjct  4096004  GTCTATTACCCCTCCCTCGTCTGATGGCCGAGTTCGTGGGGTTAGTAGAAAGCGACGCGT  4095945

Query  934      TGACGAATATTTCTCAGGAGCAGAAAGTTCTTTGGCGGGAAGCTCAAATAGCTACCAAGA  993
                ||||||||  | | || || ||||| | ||||||||||||||| ||||| ||||| ||||
Sbjct  4095944  TGACGAAT--TACGCAAGAACAGAAGGCTCTTTGGCGGGAAGCACAAATTGCTACGAAGA  4095887

Query  994      AAGTTTTGAAACCGTTTCTTTCGAAAAATTTAAAAGCTTATTCTGTGCAGAATTTGGCCG  1053
                 |||||||||||| | ||| |                |||||||||||||||||| ||||
Sbjct  4095886  GAGTTTTGAAACCATCTCTCT---------------TTTATTCTGTGCAGAATTTAGCCG  4095842

Query  1054     ACTGGATAATCACCGAAGGTACATATTACACGACATTTACCGAAATACAATTTCCGGGCG  1113
                  |||||  ||| ||| |||||||  | ||||| ||||||| |||| |   | |||| ||
Sbjct  4095841  GGTGGATTCTCA-CGACGGTACATGCTGCACGATATTTACCCAAATCCGCGTCCCGGCCG  4095783

Query  1114     -CTTTCGGGAAAATGATAGGGGAGTCGTACCCAAGGACACAGGCAACTCATCATCGGCAT  1172
                 ||||| |  ||||||| ||||| |||||||||||  |||||| ||||| ||  || | |
Sbjct  4095782  CCTTTCTG--AAATGATCGGGGAATCGTACCCAAGAGCACAGGGAACTCGTCGGCGTCGT  4095725

Query  1173     GGACATTCGTCGTCTTCCAGCACGGCCAGCATGTCCACGTCATCCACAAGTGCCGATACG  1232
                || |||||||||| ||| |||||||| |||| ||||| |||||||||  |||| ||||||
Sbjct  4095724  GGTCATTCGTCGTGTTCGAGCACGGCAAGCACGTCCATGTCATCCACCTGTGCAGATACG  4095665

Query  1233     AAAGGTCGTGGTGCTCGTGCGCACTCACGAAGAAAGCGGAAGAGCGATTCGGACGACGAA  1292
                | || || |||||||||| ||| ||||| || ||||| ||||   | ||||||||| |||
Sbjct  4095664  AGAGATCATGGTGCTCGTTCGCGCTCACCAA-AAAGCAGAAGGAAGGTTCGGACGAAGAA  4095606

Query  1293     TTGGAAGACATACTTTACGAGGCAGTAGGATCACAGAAGGCCACCTCGAACATCTCGCAA  1352
                |||||||| ||||| | ||| |||||||| || ||||| ||||| ||    || ||||||
Sbjct  4095605  TTGGAAGATATACTCTTCGAAGCAGTAGGCTCTCAGAAAGCCACATCCGCAATATCGCAA  4095546

Query  1353     ATTATATACAAAAAGGGCGACGCAGGCTCCTTCAAGCTTTCCTCGCCGGGCGAGAAAGGC  1412
                 || |||||||||||||  | | ||||| ||||||||||| |||||| || |||||||||
Sbjct  4095545  GTTGTATACAAAAAGGGAAAAGAAGGCTGCTTCAAGCTTTTCTCGCCAGGAGAGAAAGGC  4095486

Query  1413     GATTATCTCGTCAAGCTGGACTTTTACAAGACACGCAATCTTCGCAGAGTGGCAAAGGAG  1472
                || ||  |  ||||||| || || |||  ||| ||  | ||  | ||||| || |||||
Sbjct  4095485  GAATACATGATCAAGCTCGATTTCTACCGGACCCGTCAGCTGAGGAGAGTAGCGAAGGAC  4095426

Query  1473     AACTGGTGGAAGGAGGCCGATTACCGGAGCACATTTCTTCTTTCTTACGCGGAACCTGCC  1532
                || ||||||| ||| || || ||||||  ||| ||  | ||||||||| |||| |||||
Sbjct  4095425  AATTGGTGGAGGGAAGCGGACTACCGGGCCACGTTCGTGCTTTCTTACACGGATCCTGCA  4095366

Query  1533     GCAA-ACAGCATCaaaaaaaTCCTGAAAGATCAAGTGAAGAAGATAGCGAAAATTCCGGA  1591
                ||||  ||| || || || ||||| || || || || |||||||||||    ||||||||
Sbjct  4095365  GCAACTCAG-ATAAAGAAGATCCTCAAGGAGCAGGTCAAGAAGATAGCAGCGATTCCGGA  4095307

Query  1592     GAGGAAGGTGGAGTACCTGCCCCAACAAAAAGGGAGAAAATAATTCAATTTCT-TCAGTC  1650
                 |||||| | || |   | ||  |||||||||||| ||||||||| ||||| | ||| ||
Sbjct  4095306  TAGGAAGATCGAATTTTTACCAAAACAAAAAGGGAAAAAATAATTGAATTTTTATCA-TC  4095248

Query  1651     GTGCGTAATAACTCCTTTTTCTCGAATTTTTCAATCCCAATTGTACTTATTCGATAAATC  1710
                 |||||||| || ||||||||||| || ||||||||| | |||||| | |||||||||||
Sbjct  4095247  ATGCGTAATTACCCCTTTTTCTCGTATCTTTCAATCCGAGTTGTACCTTTTCGATAAATC  4095188

Query  1711     TTTATATTTTATTACACCTGAATCTATGTCTTGTCAGCTTGCCATTTTACAGCACACACA  1770
                 |||||||  ||| | || ||||||||| | |||||| | || ||  | || ||||| ||
Sbjct  4095187  GTTATATTACATT-CTCCCGAATCTATGGCCTGTCAGTTAGCGATAATGCAACACACTCA  4095129

Query  1771     TACGATTAACAAGATGACTGTGCAACAAATATTTCAGTACCAGTTAAATATTCCTTTCTC  1830
                 || ||||| ||||||| |||  | ||| |||||||||| || ||||| |||||  | ||
Sbjct  4095128  CACCATTAATAAGATGAGTGTAAATCAATTATTTCAGTATCAATTAAACATTCCGATTTC  4095069

Query  1831     AAAATTGTTTTATAATTGCGATAATCCTGAGGATTATTTTTTGAATTTATCTGCTAGCAT  1890
                 ||||| ||||| |||||  |   ||| || || || ||||| || ||    || || ||
Sbjct  4095068  TAAATTATTTTACAATTGTAACGCTCCGGATGAATA-TTTTTAAACTTGGAAGCAAGTAT  4095010

Query  1891     CCTTTGCATTGAAACTTTATTGAAACACCAGGTTGGCAGTTTCGGATTCGTAAATTTTCT  1950
                  | || |||||||| || || || || || ||||| || | ||| ||||| |||||| |
Sbjct  4095009  TTTGTGTATTGAAACATTGTT-AAGCATCAAGTTGGAAGCTACGGTTTCGTTAATTTTTT  4094951

Query  1951     CGAAAATTTGTTAAATATTCTTGATAAGCGAATTCCAAAAGTTAATACTTTGTTAATTGC  2010
                 |||||| |  | | |||  | |||||||||||||| ||||| |||||  | ||  | |
Sbjct  4094950  GGAAAATCTAATCAGTATCTTAGATAAGCGAATTCCTAAAGTAAATACAATCTTGGTCGA  4094891

Query  2011     TGGTCCTGCTAATTCAGG-AAAAAAttttttttttGATGCAGTGACACAAGCTATGATAA  2069
                 ||||| || ||||| || ||||| ||||||||||||||| || || |||||||||||||
Sbjct  4094890  AGGTCCGGCAAATTCGGGTAAAAACTTTTTTTTTTGATGCGGTTACCCAAGCTATGATAA  4094831

Query  2070     ATTGTGGTCACGTCGGAAATTTTAA  2094
                | || || ||| |||||||||||||
Sbjct  4094830  ACTGCGGGCACATCGGAAATTTTAA  4094806

Range 2: 4097378 to 4098559
Identities      Gaps            Strand
905/1187(76%)   7/1187(0%)      Plus/Minus

Query  814      TTATCGATCGATCTCCCCTTTTGACAGCGATACAGGATTACTCCATGGAACACCCCCTCG  873
                |||||||||  |||||||| ||||||||  | ||||||||| |  ||||||| ||  | |
Sbjct  4098559  TTATCGATCAGTCTCCCCTGTTGACAGCTTTTCAGGATTACACTCTGGAACATCCGTTGG  4098500

Query  874      GCCTTCTTCCGTTGCCTCGACTGATGTCCGAGTTCGTAGGCGTAATCGAAGCAAACGGAT  933
                | ||  | ||  | || || |||||| |||||||||| ||  || | |||    |||  |
Sbjct  4098499  GTCTATTACCCCTCCC-CGTCTGATGGCCGAGTTCGTGGGGTTAGTAGAAAGCGACGCGT  4098441

Query  934      TGACGAATATTTCTCAGGAGCAGAAAGTTCTTTGGCGGGAAGCTCAAATAGCTACCAAGA  993
                ||||||||||| | || || ||||| | |||| |||||||||| ||||| ||||| ||||
Sbjct  4098440  TGACGAATATTACGCAAGAACAGAAGGCTCTT-GGCGGGAAGCACAAATTGCTACGAAGA  4098382

Query  994      AAGTTTTGAAACCGTTTCTTTCGAAAAATTTAAAAGCTTATTCTGTGCAGAATTTGGCCG  1053
                 |||||||||||| | ||||||||| ||||||||   |||||||||||||||||| ||||
Sbjct  4098381  GAGTTTTGAAACCATCTCTTTCGAAGAATTTAAAGATTTATTCTGTGCAGAATTTAGCCG  4098322

Query  1054     ACTGGATAATCACCGAAGGTACATATTACACGACATTTACCGAAATACAATTTCCGGGCG  1113
                  |||||  |||| || |||||||  | ||||| ||||||| |||| |   | |||| ||
Sbjct  4098321  GGTGGATTCTCACAGACGGTACATGCTGCACGATATTTACCCAAATCCGCGTCCCGGCCG  4098262

Query  1114     CTTTCGGGAAAATGATAGGGGAGTCGTACCCAAGGACACAGGCAACTCATCATCGGCATG  1173
                | ||   ||||||||| ||||| |||||||||||  |||||| ||||| ||  || | ||
Sbjct  4098261  CCTTTCTGAAAATGATCGGGGAATCGTACCCAAGAGCACAGGGAACTCGTCGGCGTCGTG  4098202

Query  1174     GACATTCGTCGTCTTCCAGCACGGCCAGCATGTCCACGTCATCCACAAGTGCCGATACGA  1233
                | |||||||||| ||| |||||||| |||| ||||| |||||||||  |||| |||||||
Sbjct  4098201  GTCATTCGTCGTGTTCGAGCACGGCAAGCACGTCCATGTCATCCACCTGTGCAGATACGA  4098142

Query  1234     AAGGTCGTGGTGCTCGTGCGCACTCACGAAGAAAGCGGAAGAGCGATTCGGACGACGAAT  1293
                 || || ||||||| || ||| ||||| || ||||| ||||   | ||||||||| ||||
Sbjct  4098141  GAGATCATGGTGCTTGTTCGCGCTCACCAAAAAAGCAGAAGGAAGGTTCGGACGAAGAAT  4098082

Query  1294     TGGAAGACATACTTTACGAGGCAGTAGGATCACAGAAGGCCACCTCGAACATCTCGCAAA  1353
                ||||||| ||||| | ||| |||||||| || ||||| ||||| ||    || ||||||
Sbjct  4098081  TGGAAGATATACTCTTCGAAGCAGTAGGCTCTCAGAAAGCCACATCCGCAATATCGCAAG  4098022
```

```
Query  1354     TTATATACAAAAAGGGCGACGCAGGCTCCTTCAAGCTTTCCTCGCCGGGCGAGAAAGGCG  1413
                ||||| ||||||||||  | | ||||| ||||||||||| |||||| || ||||||||||
Sbjct  4098021  TTATAGACAAAAAGGGAAAAGAAGGCTGCTTCAAGCTTT-CTCGCCAGGAGAGAAAGGCG  4097963

Query  1414     ATTATCTCGTCAAGCTGGACTTTTACAAGACACGCAATCTTCGCAGAGTGGCAAAGGAGA  1473
                | ||  |  ||||||| || || |||  ||| ||  | ||  | ||||| || ||||| |
Sbjct  4097962  AATACATGATCAAGCTCGATTTCTACCGGACCCGTCAGCTGAGGAGAGTAGCGAAGGACA  4097903

Query  1474     ACTGGTGGAAGGAGGCCGATTACCGGAGCACATTTCTTCTTTCTTACGCGGAACCTGCCG  1533
                | ||||||| ||| || || ||||||  ||| ||  | ||||||||| |||| ||||| |
Sbjct  4097902  ATTGGTGGAGGGAAGCGGACTACCGGGCCACGTTCGTGCTTTCTTACACGGATCCTGCAG  4097843

Query  1534     CAA-ACAGCATCaaaaaaaTCCTGAAAGATCAAGTGAAGAAGATAGCGAAAATTCCGGAG  1592
                |||  ||| || || || ||||| || || || || || ||||||||    ||||||||
Sbjct  4097842  CAACTCAG-ATAAAGAAGATCCTCAAGGAGCAGGTCAACAAGATAGCAGCGATTCCGGAT  4097784

Query  1593     AGGAAGGTGGAGTACCTGCCCCAACAAAAAGGG-AGAAAATAATTCAATTTCTTCAGTCG  1651
                 ||||| | || |   | ||  ||||||| ||| | ||||||||| |||||  ||| ||
Sbjct  4097783  CGGAAGATCGAATTTTTACCAAAACAAAAGGGGAAAAAAATAATTGAATTTTATCA-TCA  4097725

Query  1652     TGCGTAATAACTCCTTTTTCTCGAATTTTTCAATCCCAATTGTACTTATTCGATAAATCT  1711
                |||||||| || ||||||||||| || ||||||||| | |||||| | |||||||||||
Sbjct  4097724  TGCGTAATTACCCCTTTTTCTCGTATCTTTCAATCCGAGTTGTACCTTTTCGATAAATCG  4097665

Query  1712     TTATATTTTATTACACCTGAATCTATGTCTTGTCAGCTTGCCATTTTACAGCACACACAT  1771
                |||||||  ||||| || ||||||||| | |||||| | || |   | || ||||| ||
Sbjct  4097664  TTATATTACATTACTCCCGAATCTATGGCCTGTCAGTTAGCGACAATGCAACACACTCAC  4097605

Query  1772     ACGATTAACAAGATGACTGTGCAACAAATATTTCAGTACCAGTTAAATATTCCTTTCTCA  1831
                || ||||| ||||||| |||  | ||| |||||||||| || ||||| |||||  | ||
Sbjct  4097604  ACCATTAATAAGATGAGTGTTAATCAATTATTTCAGTATCAATTAAACATTCCGATTTCT  4097545

Query  1832     AAATTGTTTTATAATTGCGATAATCCTGAGGATTATTTTTTGAATTTATCTGCTAGCATC  1891
                ||||| ||||| |||||  |   ||| || || |||||||| || ||    || || ||
Sbjct  4097544  AAATTATTTTACAATTGTAACGCTCCGGATGAATATTTTTTAAACTTGGAAGCAAGTATT  4097485

Query  1892     CTTTGCATTGAAACTTTATTGAAACACCAGGTTGGCAGTTTCGGATTCGTAAATTTTCTC  1951
                 | || |||||||| || || || || || ||||| || | ||| ||||| |||||| |
Sbjct  4097484  TTGTGTATTGAAACATTGTTAAAGCATCAAGTTGGAAGCTACGGTTTCGTTAATTTTTTG  4097425

Query  1952     GAAAATTTGTTAAATATTCTTGATAAGCGAATTCCAAAAGTTAATAC  1998
                |||||| |  | | |||  | |||||||||||||| ||||| |||||
Sbjct  4097424  GAAAATCTAATCAGTATCTTAGATAAGCGAATTCCTAAAGTAAATAC  4097378
```

The proteins encoded in the two BtaDV-related EVEs present in the B. tabaci SS3 Nigeria are homologous the BtaDV NS1 Rep endonuclease domain and more related to one of the two types of NS1 Rep-encoding EVEs of the B. tabaci SS2 Nigeria genome (see below)

```
>1_SS3_Nigeria
RILRKNRRLLAGSTNCYEESFETISFEEFKDLFCAEFSRVDSHRRYMLHDIYPNPRPGRLSENDRGIVPKSTGNSSASWSFVVFEHGKHVHVIHLCRYERSWCLFALTKKAEGRFGRRIGRYTLRSSRLSESHIRNIASYRQKGKRRLLQAFSPGEKGEYMIKLDFYRTRQLRRVAKDNWWREADYRA
TFVLSYTDPAATQIKKILKEQVNKIAAIPDRKIEFLPKQKGKKIIEFYHHA
>2_SS3_Nigeria
NDRGIVPKSTGNSSASWSFVVFEHGKHVHVIHLCRYERSWCSFALTKKQKEGSDEELEDILFEAVGSQKATSAISQVVYKKGKEGCFKLFSPGEKGEYMIKLDFYRTRQLRRVAKDNWWREADYRATFVLSYTDPAATQIKKILKEQVKKIAAIPDRKIEFLPKQKGKK

1_SS3_Nigeria      SENDRGIVPKSTGNSSASWSFVVFEHGKHVHVIHLCRYERSWCLFALTKKAEGRFGRRIG
2_SS3_Nigeria      --NDRGIVPKSTGNSSASWSFVVFEHGKHVHVIHLCRYERSWCSFALTKKQKEGSDEELE
                     ***************************************** ****** :   .  :

1_SS3_Nigeria      R--YTLRSSRLSESHIRNIASYRQKGKRRLLQAFSPGEKGEYMIKLDFYRTRQLRRVAKD
2_SS3_Nigeria      DILFEAVGSQKATSAISQVVY--KKGKEGCFKLFSPGEKGEYMIKLDFYRTRQLRRVAKD
                      :   .*. : * * ::.   :***   :: ***************************

1_SS3_Nigeria      NWWREADYRATFVLSYTDPAATQIKKILKEQVNKIAAIPDRKIEFLPKQKGKKIIEFYHH
2_SS3_Nigeria      NWWREADYRATFVLSYTDPAATQIKKILKEQVKKIAAIPDRKIEFLPKQKGKK-------
                   ********************************:********************

1_SS3              1 -----RILRKNR--RL----------LAGSTNCYEESFETISFEEFKDLF     33
                          |:.|:||  |:          ||||:|.|:|||||:|||:||.||
BtaDV_NS1        151 ASTDVRVRRRNRSKRIDEYFSGAESSLAGSSNSYQESFETVSFEKFKSLF    200

1_SS3             34 CAEFSRVDSHRRYMLHDIYPNPRPGRLSENDRGIVPKSTGNSSASWSFVV     83
                     ||||.|:|:||||:|||||.|...||..|||||:|||.||||||::|:|||
BtaDV_NS1        201 CAEFGRLDNHRRYILHDIYRNTISGRFRENDRGVVPKDTGNSSSAWTFVV    250

1_SS3             84 FEHGKHVHVIHLCRYERSWCLFALTKKAEGRFGRRIGRYTLRSSRLSESH    133
                     |:||:||||||.||||||||..|||||||.||||||||:|||.||::|.|
BtaDV_NS1        251 FQHGQHVHVIHKCRYERSWCSCALTKKAEERFGRRIGRHTLRGSRITEGH    300

1_SS3            134 IRNIASYRQKGKRRLLQAFSPGEKGEYMIKLDFYRTRQLRRVA---KDNW    180
                     :.::|:|.|||:|||||||..|             |.|:|.|.|   :|
BtaDV_NS1        301 LEHLANYIQKGRRRLLQAFLAG------------RERRLSRQAGLLQD--    336

>1_SS2_Nigeria
PGGFSQTVHAARYLPKSASRRLSENDRGIVPKSTGNSSASWSFVVFEHGKHVHVIHLCRYERSWCLFALTKKAEGRFGRRIGRYTLRSSRLSESHIRNIASYRQKGKRRLLQAFLARRERRIHDQARFLPDPSAEESSEGQLVEGSGLPGHVRAFLHGSCSNSDKEDPQGAGQEDSSDSGSEDRIFTK
TKREKK
>5_SS2_Nigeria
TWKHSIDDYRSVSPVDSFSGLHSGTSVGSITPPPSDGRVRGVSRKRRIDEYYARTEGSLAGSTNCYEESFETISFEEFKDLFCAEFSRVDSHRRYMLHDIYPNPRPGRLSENDRGIVPKSTGNSSASWSFVVFEHGKHVHVIHLCRYERSWCLFALTKKAEGRFGRRIGRYTLRSSRLSESHIRNIAS
YRQKGKRGCFKLFSPGEKGEYMIKLDFYRTRQLRRVAKDNWWREADYRATFVLSYTDPAATQIKKILKEQVKKIAAIPDRKIEFLQKQKGKKNN

1_SS3_Nigeria  GSTNCYEESFETISFEEFKDLFCAEFSRVDSHRRYMLHDIYPNPRPGRLSENDRGIVPKSTGNSSASWSFVVFEHGKHVHVIHLCRYERSWCLFALTKKAEGRFGRRIGRYTLRSSRLSE
5_SS2_Nigeria  GSTNCYEESFETISFEEFKDLFCAEFSRVDSHRRYMLHDIYPNPRPGRLSENDRGIVPKSTGNSSASWSFVVFEHGKHVHVIHLCRYERSWCLFALTKKAEGRFGRRIGRYTLRSSRLSE
               ************************************************************************************************************************
1_SS3_Nigeria  SHIRNIASYRQKGKRRLLQAFSPGEKGEYMIKLDFYRTRQLRRVAKDNWWREADYRATFVLSYTDPAATQIKKILKEQVNKIAAIPDRKIEFLPKQKGKKIIEFYHHA
5_SS2_Nigeria  SHIRNIASYRQKGKRGCFKLFSPGEKGEYMIKLDFYRTRQLRRVAKDNWWREADYRATFVLSYTDPAATQIKKILKEQVKKIAAIPDRKIEFLQKQKGKK------NN
               ***************  :: *************************************************:************* ******      :
1_SS2_Nigeria      ---------------------PGGFSQTVHAARYL-----PKSASRRLSENDRGIVPKS
1_SS3_Nigeria      GSTNCYEESFETISFEEFKDLFCAEFSRVDSHRRYMLHDIYPNPRPGRLSENDRGIVPKS
5_SS2_Nigeria      GSTNCYEESFETISFEEFKDLFCAEFSRVDSHRRYMLHDIYPNPRPGRLSENDRGIVPKS
                                         . **..    **:     *:. . *************

1_SS2_Nigeria      TGNSSASWSFVVFEHGKHVHVIHLCRYERSWCLFALTKKAEGRFGRRIGRYTLRSSRLSE
1_SS3_Nigeria      TGNSSASWSFVVFEHGKHVHVIHLCRYERSWCLFALTKKAEGRFGRRIGRYTLRSSRLSE
5_SS2_Nigeria      TGNSSASWSFVVFEHGKHVHVIHLCRYERSWCLFALTKKAEGRFGRRIGRYTLRSSRLSE
                   ************************************************************

1_SS2_Nigeria      SHIRNIASYRQKGKRRLLQAFLARRERRIHDQARFLPDPSAEESSEGQLVEGSGLPGHVR
1_SS3_Nigeria      SHIRNIASYRQKGKRRLLQAFSPGEKGEYMIKLDFYRTRQLRRVAKDNWWREADY----R
5_SS2_Nigeria      SHIRNIASYRQKGKRGCFKLFSPGEKGEYMIKLDFYRTRQLRRVAKDNWWREADY----R
                   ***************  :: *  .  :      :  *     .    ::.:    :.     *
```

BtaDV-related EVEs in the genome of *B. tabaci* SSA2 population from Nigeria (BioProject: PRJEB28507)

Descriptions | Graphic Summary | Alignments

Sequences producing significant alignments — Download — Manage columns — Show 100

select all *5 sequences selected* — GenBank — Graphics — Distance tree of results — MSA Viewer

	Description	Max Score	Total Score	Query Cover	E value	Per. Ident	Acc. Len	Accession
☑	Bemisia tabaci strain SSA2 Nigeria F6 genome assembly, contig: CONTIG78	894	21385	26%	0.0	75.76%	8195669	CAJEWI010000021.1
☑	Bemisia tabaci strain SSA2 Nigeria F6 genome assembly, contig: CONTIG682	298	387	14%	3e-78	72.20%	5433705	CAJEWI010000032.1
☑	Bemisia tabaci strain SSA2 Nigeria F6 genome assembly, contig: CONTIG644	92.4	92.4	6%	9e-16	67.38%	12776201	CAJEWI010000010.1
☑	Bemisia tabaci strain SSA2 Nigeria F6 genome assembly, contig: CONTIG570	86.9	154	4%	4e-14	71.35%	20083235	CAJEWI010000004.1
☑	Bemisia tabaci strain SSA2 Nigeria F6 genome assembly, contig: CONTIG322	68.9	68.9	5%	1e-08	66.67%	971963	CAJEWI010000085.1

Distribution of the top 35 Blast Hits on 5 subject sequences

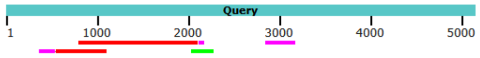

Bemisia tabaci strain SSA2 Nigeria F6 genome assembly, contig: CONTIG78, whole genome shotgun sequence

Sequence ID: CAJEWI010000021.1Length: 8195669 **Number of Matches: 29**

```
Range 1: 602822 to 604120
Identities      Gaps          Strand
994/1312(76%)   17/1312(1%)   Plus/Plus

Query  787     AAACAAGGAAGCAGGAAATTCAGGACTTTATCGATCGATCTCCCCTTTTGACAGCGATAC  846
               ||||  |||||||    ||| | ||  |||||||||  |||||||| ||||||||  | |
Sbjct  602822  AAACTTGGAAGCATTCGATTGACGA--TTATCGATCAGTCTCCCCTGTTGACAGCTTTTC  602879

Query  847     AGGATTACTCCATGGAACACCCCCTCGGCCTTCTTCCGTTGCCTCGACTGATGTCCGAGT  906
               |||||||| |  ||||||| ||  | || ||  | || |  || || | |||| ||||||
Sbjct  602880  AGGATTACACTCTGGAACATCCGTTGGGTCTATTACCCTCCCC-CGTCCGATGGCCGAGT  602938

Query  907     TCGTAGGCGTAATCGAAGCAAACGGATTGACGAATATTTCTCAGGAGCAGAAAGTTCTTT  966
               |||| ||  || | |||    ||| ||||||||||||| | || || ||||| | |||||
Sbjct  602939  TCGTGGGGTTAGTAGAAAGCGACGCATTGACGAATATTACGCAAGAACAGAAGGCTCTTT  602998

Query  967     GGCGGGAAGCTCAAATAGCTACCAAGAAAGTTTTGAAACCGTTTCTTTCGAAAAATTTAA  1026
               |||||||||| ||||| ||||| |||| |||||||||||| | ||||||||| |||||||
Sbjct  602999  GGCGGGAAGCACAAATTGCTACGAAGAGAGTTTTGAAACCATCTCTTTCGAAGAATTTAA  603058

Query  1027    AAGCTTATTCTGTGCAGAATTTGGCCGACTGGATAATCACCGAAGGTACATATTACACGA  1086
               |   ||||||||||||||||| | |||  |||||  |||| || |||||||  | |||||
Sbjct  603059  AGATTTATTCTGTGCAGAATTAG-CCGGGTGGATTCTCACAGACGGTACATGCTGCACGA  603117

Query  1087    CATTTACCGAAATACAATTTCCGGGCGCTTTCGGGAAAATGATAGGGGAGTCGTACCCAA  1146
                ||| ||| |||| |   | |||| ||| ||   ||||||||| ||||| ||||||||||
Sbjct  603118  TATT-ACC-AAATCCGCGTCCCGGCCGCCTTTCTGAAAATGATCGGGGAATCGTACCCAA  603175

Query  1147    GGACACAGGCAACTCATCATCGGCATGGACATTCGTCGTCTTCCAGCACGGCCAGCATGT  1206
               |  |||||| |||||  ||  || | ||| |||||||||| ||| |||||||| |||| ||
Sbjct  603176  GAGCACAGGGAACTCGTCGGCGTCGTGGTCATTCGTCGTGTTCGAGCACGGCAAGCACGT  603235

Query  1207    CCACGTCATCCACAAGTGCCGATACGAAAGGTCGTGGTGCTCGTGCGCACTCACGAAGAA  1266
               ||| |||||||||  |||| ||||||| || || ||||||| || ||| ||||| || ||
Sbjct  603236  CCATGTCATCCACCTGTGCAGATACGAGAGATCATGGTGCTTGTTCGCGCTCACCAAAAA  603295

Query  1267    AGCGGAAGAGCGATTCGGACGACGAATTGGAAGACATACTTTACGAGGCAGTAGGATCAC  1326
               ||| ||||   | ||||||||| ||||||||||| ||||| | ||| |||||||| || |
Sbjct  603296  AGCAGAAGGAAGGTTCGGACGAAGAATTGGAAGATATACTCTTCGAAGCAGTAGGCTCTC  603355

Query  1327    AGAAGGCCACCTCGAACATCTCGCAAATTATATACAAAAAGGGCGACGCAGGCTCCTTCA  1386
               |||| ||||| ||    || |||||| ||||| ||||||||||  | | ||||| |||||
Sbjct  603356  AGAAAGCCACATCCGCAATATCGCAAGTTATAGACAAAAAGGGAAAAGAAGGCTGCTTCA  603415

Query  1387    AGCTTTCCTCGCCGGGCGAGAAAGGCGATTATCTCGTCAAGCTGGACTTTTACAAGACAC  1446
               |||||| |||||| || ||||||||||| ||  |  ||||||| || || |||  ||| |
Sbjct  603416  AGCTTTTCTCGCCAGGAGAGAAAGGCGAATACATGATCAAGCTCGATTTCTACCGGACCC  603475

Query  1447    GCAATCTTCGCAGAGTGGCAAAGGAGAACTGGTGGAAGGAGGCCGATTACCGGAGCACAT  1506
               |  | ||  | ||||| || ||||| || ||||||| ||| || || ||||||  ||| |
Sbjct  603476  GTCAGCTGAGGAGAGTAGCGAAGGACAATTGGTGGAGGGAAGCGGACTACCGGGCCACGT  603535

Query  1507    TTCTTCTTTCTTACGCGGAACCTGCCGCAA-ACAGCATCaaaaaaaTCCTGAAAGATCAA  1565
               |  | ||||||||| |||| ||||| ||||  ||| || || || ||||| || || || 
Sbjct  603536  TCGTGCTTTCTTACACGGATCCTGCAGCAACTCAG-ATAAAGAAGATCCTCAAGGAGCAG  603594

Query  1566    GTGAAGAAGATAGCGAAAATTCCGGAGAGGAAGGTGGAGTACCTGCCCCAACAAAAAGGG  1625
               || |||||||||||    ||||||||  ||||| | || |   | |   |||||||||||
Sbjct  603595  GTCAAGAAGATAGCAGCGATTCCGGATCGGAAGATCGAATTTTTACAAAAACAAAAAGGG  603654

Query  1626    --AGAAAATAATTCAATTTCT-TCAGTCGTGCGTAATAACTCCTTTTTCTCGAATTTTTC  1682
                 | ||||||||| ||||| | ||| || |||||||| || || |||||||| || ||||
Sbjct  603655  AAAAAAAATAATTGAATTTTTATCA-TCATGCGTAATTAC-CCCTTTTCTCGTATCTTTC  603712

Query  1683    AATCCCAATTGTACTTATTCGATAAATCTTTATATTTTATTACACCTGAATCTATGTCTT  1742
               ||||| | |||||  | ||||||||||| |||||||  ||||| || ||||||||| | |
Sbjct  603713  AATCCGAGTTGTATCTTTTCGATAAATCGTTATATTACATTACTCCCGAATCTATGGCCT  603772

Query  1743    GTCAGCTTGCCATTTTACAGCACACACATACGATTAACAAGATGACTGTGCAACAAATAT  1802
               ||||| | || ||  | || ||||| || || ||||| ||||||| |||  | ||| |||
Sbjct  603773  GTCAGTTAGCGATAATGCAACACACTCACACCATTAATAAGATGAGTGTAAATCAATTAT  603832

Query  1803    TTCAGTACCAGTTAAATATTCCTTTCTCAAAATTGTTTTATAATTGCGATAATCCTGAGG  1862
               ||||||| || ||||| |||||  | || ||||| ||||| |||||  |   ||| || |
Sbjct  603833  TTCAGTATCAATTAAACATTCCGATTTCTAAATTATTTTACAATTGTAACGCTCCGGATG  603892

Query  1863    ATTATTTTTTGAATTTATCTGCTAGCATCCTTTGCATTGAAACTTTATTGAAACACCAGG  1922
               | |||||||| || ||    || || ||  | || |||||||| || || || || || |
Sbjct  603893  AATATTTTTTAAACTTGGAAGCAAGTATTTTGTGTATTGAAACATTGTTAAAGCATCAAG  603952

Query  1923    TTGGCAGTTTCGGATTCGTAAATTTTCTCGAAAATTTGTTAAATATTCTTGATAAGCGAA  1982
               |||| || | ||| ||||| |||||| | ||||| |   | | |||  | ||||||||||
Sbjct  603953  TTGGAAGCTACGGTTTCGTTAATTTTTTGGAAAATCTAATCAGTATCTTAGATAAGCGAA  604012

Query  1983    TTCCAAAAGTTAATACTTTGTTAATTGCTGGTCCTGCTAATTCAGGAAAAAAtttttttt  2042
               |||| ||||| |||||  | ||  | |  ||||| || ||||| || |||||     |||
Sbjct  604013  TTCCTAAAGTAAATACAATCTTGGTCGAAGGTCCGGCAAATTCGGGTAAAAA----CTTT  604068

Query  2043    ttGATGCAGTGACACAAGCTATGATAAATTGTGGTCACGTCGGAAATTTTAA  2094
               ||||||| || || |||||||||||||| || || ||| |||||||||||||
Sbjct  604069  TTGATGCGGTTACCCAAGCTATGATAAACTGCGGGCACATCGGAAATTTTAA  604120
```

The protein encoded in the integrated sequence (Rep motifs highlighted)

```
ORF6              1 -------------------------------------QLFRITLWNIRWV     13
                                                         .|...|
BtaDV_NS1       101 GEGSSGASSGSIPRDDGNKEAGNSGLYRSISPFDSDTGLLHGT-------    143

ORF6             14 YYPP-PS-----DGRVRGVSRKRRIDEYYARTEGSLAGSTNCYEESFETI     57
                      || ||     |.|||..:|.:|||||::..|.|||||:|.|:|||||:
BtaDV_NS1       144 --PPRPSSVASTDVRVRRRNRSKRIDEYFSGAESSLAGSSNSYQESFETV    191

ORF6             58 SFEEFKDLFCAELA------GWILTDGTCCTILPNPRPGRLSENDRGIVP    101
                    |||:||.|||||..      .:||.|     |..|...||..|||||:||
BtaDV_NS1       192 SFEKFKSLFCAEFGRLDNHRRYILHD-----IYRNTISGRFRENDRGVVP    236

ORF6            102 KSTGNSSASWSFVVFEHGKHVHVIHLCRYERSWCLFALTKKAEGRFGRRI    151
                    |.|||||::|:||||:||:||||||.||||||||..|||||||.||||||
BtaDV_NS1       237 KDTGNSSSAWTFVVFQHGQHVHVIHKCRYERSWCSCALTKKAEERFGRRI    286

ORF6            152 GRYTLRSSRLSESHIRNIASYRQKGKRRLLQAFLARRERRIHDQARFLPD    201
                    ||:|||.||::|.|:.::|:|:|.|||:|||||||||.||||:..||..|.|
BtaDV_NS1       287 GRHTLRGSRITEGHLEHLANYIQKGRRRLLQAFLAGRERRLSRQAGLLQD    336

ORF6            202 PSAEESSEGQLVEGSGLPGHVRAFLHGSCSNSDKEDPQGAGQEDSSDSGS    251
                    ..:.:|.:|:||||..||.|:.:||.|:|....:::|:.:.:|||.:||.
BtaDV_NS1       337 TQSSQSGKGELVEGGRLPEHISSFLRGTCRKQHQKNPERSSEEDSENSGE    386

ORF6            252 EDRIFTKTKREKK-------------------------------------    264
                    |..:...|||||.
BtaDV_NS1       387 EGGVPAPTKREKIIQFLQSCVITPFSRIFQSQLYLFDKSLYFITPESMSC    436
```

The 32.5 Kb EVE with an array or NS1 Rep nikase domain coding sequences

```
>CAJEWI010000021.1:570594-604120 Bemisia tabaci strain SSA2 Nigeria F6 genome assembly, contig: CONTIG78, whole genome shotgun sequence
TTATCGATCAGTCTCCCCTGTTGACAGCTTTTCAGGATTACACTCTGGAACATTCGTTGGGTCTATTACCCCTCCCCCGTCTGATGGCCGAGTTCGTGGGGTTAGTAGAAAGCGACGCATTGACGAATATTACGCAAGAACAGAAGGCTCTTTGGCGGGAAGCACAAATTGCTACGAAGAGAGTTTGAAACCATCTCTTTCGAAGAATTTAAAGATTTATTCTGTGCAGAATTTAGCCGGGTGGATTCTC
ACAGACGGTACATGCTGCACGATATTTACCCAAATCCGCGTCCCGGCGCCTTTCTGAAAATGATCGGGGAATCGTACCCAAGAGCACAGGGAACTCGTCGGCGTCGTGGTCATTCGTCGTGTTCGAGCACGGCAAGCACGTCCATGTCATCCACCTGTGCAGATACGAGAGATCATGGTGCTTGTTCGCGCTCACCAAAAAAGCAGAAGGAAGGTTCGGACGAAGAATTGGAAGATATACTCTTCGAAGC
AGTAGGCTCTCAGAAAGCCACATCCGCAATATCGCAAGTTATAGACAAAAAGGGAAAAGAAGGCTGCTTCAAGCTTTTCTCGCCAGGAGAGAAAGGCGAATACATGATCAAGCTCGATTTCTACCGGACCCGTCAGCTGAGGAGAGTAGCGAAGGACAATTGGTGGAGGGAAGCGGACTACCGGGCCACGTTCGTGCTTTCTTACACGGATCCTGCAGCAACTCAGATAAAGAAGATCCTCAAGGAGCAG
GTCAAGAAGATAGCAGCGATTCCGGATCGGAAGATCGAATTTTTACAAAAACAAAAAGGGAAAAAAAATAATTGAATTTTTATCATCATGCGTAATTACCCCTTTTCTCGTATCTTTCAATCCGAGTTGTATCTTTTCGATAAATCGTTATATTACATTACTCCCGAATCTATGGCCTGTCAGTTAGCGATAATGCAACACACTCACACCATTAATAAGATGAGTGTAAATCAATTATTTCAGTATCAAT
TAAACATTCCGATTTCTAAATTATTTTACAATTGTAACGCTCCGGATGAATATTTTTAAACTTGGAAGCATTCGATTGACGATTATCGATCAGTCTCCCCTGTTGACAGCTTTTCAGGATTACACTCTGGAACATCCGTTGGGTCTATTACCCCTCCCCCGTCCGATGGCCGAGTTCGTGGGGTTAGTAGAAAGCGACGCATTGACGAATATTACGCAAGAACAGAAGGCTCTTTGGCGGGAAGCACAAA
TTGCTACGAAGAGAGTTTGAAACCATCTCTTTCGAAGAATTTAAAGATTTATTCTGTGCAGAATTTAGCCGGGTGGATTCTCACAGACGGTACATGCTGCACGATATTTACCCAAATCCGCGTCCCGGCCGCCTTTCTGAAAATGATGGGAATCGTACCCAAGAGCACAGGGAACTCGTCGGCGTCGTGGTCATTCGTCGTGTTCGAGCACGGCAAGCACGTCCATGTCATCCACCTGTGCAGATACGA
```

[illegible]

>2

[illegible]

>3

[illegible]

>4

[illegible]

>5

[illegible]

>6

[illegible]

>7

[illegible]

>8

[illegible]

>9

[illegible]

>10

[illegible]

>11

[illegible]

AATTGGAAGATATACTCTTCGAAGCAGTAGGCTCTCAGAAAGCCACATCCGCAATATCGCAAGTTATAGACAAAAAGGGAAAAGAAGGCTGCTTCAAGCTTTTCTCGCCAGGAGAGAAAGGCGAATACATGATCAAGCTCGATTTCTACCGGACCCGTCAGCTGAGGAGAGTAGCGAAGGACAATTGGTGGAGGGAAGCGGACTACGGGCCACGTTCGTGCTTTCTTACACGGATCCTGCAGCAACTCAG
ATAAAGAAGATCCTCAAGGAGCAGGTCAAGAAGATAGCAGCGATTCCGGATCGGAAGATCGATTTTACAAAAACAAAAAGGGAAAAAAATAATTGAATTTTTATCATCATGCGTAATTACCCCTTTTCTCGTATCTTTCAATCCGAGTTGTATCTTTTCGATAAATCGTTATATTACATTACTCCCGAATCTATGGCCTGTCAGTTAGCGATAATGCAACACACTCACACCATTAATAAGATGAGTGT
AAATCAATTATTTCAGTATCAATTAAACATTCCGATTTCTAAATTATTTTACAATTGTAACGCTCCGGATGAATATTTTTT
>12
AAACTTGGAAGCATTCGATTGACGATTATCGATCAGTCTCCCCTGTTGACAGCTTTTCAGGATTACACTCTGGAACATCCGTTGGGTCTATTACCCCTCCCCCGTCCGATGGCCGAGTTCGTGGGGTTAGTAGAAAGCGACGCATTGACGAATATTACGCAAGAACAGAAGGCTCTTTGGCGGGAAGCACAAATTGCTACGAAGAGAGTTTTGAAACCATCTCTTTCGAAGAATTTAAAGATTTATTCTG
TGCAGAATTTAGCCGGGTGGATTCTCACAGACGGTACATGCTGCACGATATTTACCCAAATCCGCGTCCCGGCCGCCTTTCTGAAAATGATCGGGGAATCGTACCCAAGAGCACAGGGAACTCGTCGGCGTCGTGGTCATTCGTCGTGTTCGAGCACGGCAAGCACGTCCATGTCATCCACCTGTGCAGATACGAGAGATCATGGTGCTTGTTCGCGCTCACCAAAAAAGCAGAAGGAAGGTTCGGACGA
AGAATTGGAAGATATACTCTTCGAAGCAGTAGGCTCTCAGAAAGCCACATCCGCAATATCGCAAGTTATAGACAAAAAGGGAAAAGAAGGCTGCTTCAAGCTTTTCTCGCCAGGAGAGAAAGGCGAATACATGATCAAGCTCGATTTCTACCGGACCCGTCAGCTGAGGAGAGTAGCGAAGGACAATTGTGGAGGGAAGCGGACTACCGGGCCACGTTCGTGCTTTCTTACACGGATCCTGCAGCAACTC
AGATAAAGAAGATCCTCAAGGAGCAGGTCAAGAAGATAGCAGCGATTCCGGATCGGAAGATCGAATTTTTACAAAAACAAAAAGGGAAAAAAATAATTGATTTTTATCATCATGCGTAATTACCCCTTTTCTCGTATCTTTCAATCCGAGTTGTATCTTTTCGATAAATCGTTATATTACATTACTCCCGAATCTATGGCCTGTCAGTTAGCGATAATGCAACACACTCACACCATTATAAGATGAGTGT
AAATCAATTATTTCAGTATCAATTAAACATTCCGATTTCTAAATTATTTTACAATTGTAACGCTCCGGATGAATATTTTT
>13
AAACTTGGAAGCATTCGATTGACGATTATCGATCAGTCTCCCCTGTTGACAGCTTTTCAGGATTACACTCTGGAACATCCGTTGGGTCTATTACCCCTCCCCCGTCCGATGGCCGAGTTCGTGGGGTTAGTAGAAAGCGACGCATTGACGAATATTACGCAAGAACAGAAGGCTCTTTGGCGGGAAGCACAAATTGCTACGAAGAGAGTTTTGAAACCATCTCTTTCGAAGAATTTAAAGATTTATTCTG
TGCAGAATTTAGCCGGTGGATTCTCACAGACGGTACATGCTGCACGATATTTACCCAAATCCGCGTCCCGGCCGCCTTTCTGAAAATGATCGGGGAATCGTACCCAAGAGCACAGGGAACTCGTCGGCGTCGTGGTCATTCGTCGTGTTCGAGCACGGCAAGCACGTCCATGTCATCCACCTGTGCAGATACGAGAGATCATGGTGCTTGTTCGCGCTCACCAAAAAAGCAGAAGGAAGGTTCGGACGAA
GAATTGGAAGATATACTCTTCGAAGCAGTAGGCTCTCAGAAAGCCACATCCGCAATATCGCAAGTTATAGACAAAAAGGGAAAAGAAGGCTGCTTCAAGCTTTTCTCGCCAGGAGAGAAAGGCGAATACATGATCAAGCTCGATTTCTACCGGACCCGTCAGCTGAGGAGAGTAGCGAAGGACAATTGGTGGAGGGAAGCGGACTACCGGGCCACGTTCGTGCTTTCTTACACGGATCCTGCAGCAACTC
AGATAAAGAAGATCCTCAAGGAGCAGGTCAAGAAGATAGCAGCGATTCCGGATCGGAAGATCGAATTTTTACAAAAACAAAAAGGGAAAAAAATAATTGAATTTTTATCATCATGCGTAATTACCCCTTTTCTCGTATCTTTCAATCCGAGTTGTATCTTTTCGATAAATCGTTATATTACATTACTCCCGAATCTATGGCCTGTCAGTTAGCGATAATGCAACACACTCACACCATTAATAAGATGAGT
GTAAATCAATTATTTCAGTATCAATTAAACATTCCGATTTCTAAATTATTTTACAATTGTAACGCTCCGGATGAATATTTTTT
>14
AAACTTGGAAGCATTCGATTGACGATTATCGATCAGTCTCCCCTGTTGACAGCTTTTCAGGATTACACTCTGGAACATCCGTTGGGTCTATTACCCCTCCCCGTCCGATGGCCGAGTTCGTGGGGTTAGTAGAAAGCGACGCATTGACGAATATTACGCAAGAACAGAAGGCTCTTTGGCGGGAAGCACAAATTGCTACGAAGAGAGTTTTGAAACCATCTCTTTCGAAGAATTTAAAGATTTATTCTGT
GCAGAATTTAGCCGGGTGGATTCTCACAGACGGTACATGCTGCACGATATTTACCCAAATCCGCGTCCCGGCCGCCTTTCTGAAAATGATCGGGGAATCGTACCCAAGAGCACAGGGAACTCGTCGGCGTCGTGGTCATTCGTCGTGTTCGAGCACGGCAAGCACGTCCATGTCATCCACCTGTGCAGATACGAGAGATCATGGTGCTTGTTCGCGCTCACCAAAAAAGCAGAAGGAAGGTTCGGACGAA
GAATTGGAAGATATACTCTTCGAAGCAGTAGGCTCTCAGAAAGCCACATCCGCAATATCGCAAGTTATAGACAAAAAGGGAAAAGAAGGCTGCTTCAAGCTTTTCTCGCCGGGAGAGAAAGGCGAATACATGATCAAGCTCGATTTCTACCGGACCCGTCAGCTGAGGAGAGTAGCGAAGGACAATTGGTGGAGGGAAGCGGACTACCGGGCCACGTTCGTGCTTTCTTACACGGATCCTGCAGCAACTC
AGATAAAGAAGATCCTCAAGGAGCAGGTCAAGAAGATAGCAGCGATTCCGGATCGGAAGATCGAATTTTTACAAAAACAAAAGGGAAAAAAATAATTGAATTTTTATCATCATGCGTAATTACCCCTTTTCTCGTATCTTTCAATCCGAGTTGTATCTTTTCGATAAATCGTTATATTACATTACTCCCGAATCTATGGCCTGTCAGTTAGCGATAATGCAACACACTCACACCATTAATAAGATGAGT
GTAAATCAATTATTTCAGTATCAATTAAACATTCCGATTTCTAAATTATTTTACAATTGTAACGCTCCGGATGAATATTTTT
>15
AAACTTGGAAGCATTCGATTGACGATTATCGATCAGTCTCCCCTGTTGACAGCTTTTCAGGATTACACTCTGGAACATCCGTTGGGTCTATTACCCCTCCCCCGTCCGATGGCCGAGTTCGTGGGGTTAGTAGAAAGCGACGCATTGACGAATATTACGCAAGAACAGAAGGCTCTTTGGCGGGAAGCACAAATTGCTACGAAGAGAGTTTTGAAACCATCTCTTTCGAAGAATTTAAAGATTTATTCTG
TGCAGAATTTAGCCGGGTGGATTCTCACAGACGGTACATGCTGCACGATATTTACCCAAATCCGCGTCCCGGCCGCCTTTCTGAAAATGATCGGGGAATCGTACCCAGAGCACAGGGAACTCGTCGGCGTCGTGGTCATTCGTCGTGTTCGAGCACGGCAAGCACGTCCATGTCATCCACCTGTGCAGATACGAGAGATCATGGTGCTTGTTCGCGCTCACCAAAAAAGCAGAAGGAAGGTTCGGACGAA
GAATTGGAAGATATACTCTTCGAAGCAGTAGGCTCTCAGAAAGCCACATCCGCAATATCGCAAGTTATAGACAAAAAGGGAAAAGAAGGCTGCTTCAAGCTTTTCTCGCCAGGAGAGAAAGGCGAATACATGATCAAGCTCGATTTCTACCGGACCCGTCAGCTGAGGAGAGTAGCGAAGGACAATTGGTGGAGGGAAGCGGACTACCGGGCCACGTTCGTGCTTTCTTACACGGATCCTGCAGCAACTCA
GATAAAGAAGATCCTCAAGGAGCAGGTCAAGAAGATAGCAGCGATTCCGGATCGGAAGATCGAATTTTTACAAAAACAAAAAGGGAAAAAAATAATTGAATTTTTATCATCATGCGTAATTACCCCTTTTCTCGTATCTTTCAATCCGAGTTGTATCTTTTCGATAAATCGTTATATTACATTACTCCCGAATCTATGGCCTGTCAGTTAGCGATAATGCAACACTCACACCATTAATAAGATGAGTGT
AAATCAATTATTTCAGTATCAATTAAACATTCCGATTTCTAAATTATTTTACAATTGTAACGCTCCGGATGAATATTTTT
>16
AAACTTGGAAGCATTCGATTGACGATTATCGATCAGTCTCCCCTGTTGACAGCTTTTCAGGATACACTCTGGAACATCCGTTGGGTCTATTACCCCTCCCCGTCCGATGGCCGAGTTCGTGGGGTTAGTAGAAAGCGACGCATTGACGAATATTACGCAAGAACAGAAGGCTCTTTGGCGGGAAGCACAAATTGCTACGAAGAGAGTTTTGAAACCATCTCTTTCGAAGAATTTAAAGATTTATTCTGTG
CAGAATTTAGCCGGGGTGATTCTCACAGACGGTACATGCTGCACGATATTTACCCAAATCCGCGTCCCGGCCGCCTTTCTGAAAATGATCGGGGAATCGTACCCAAGAGCACAGGGAACTCGTCGGCGTCGTGGTCATTCGTCGTGTTCGAGCACGGCAAGCACGTCCATGTCATCCACCTGTGCAGATACGAGAGATCATGGTGCTTGTTCGCGCTCACCAAAAAAGCAGAAGGAAGGTTCGGACGAAGA
ATTGGAAGATATACTCTTCGAAGCAGTAGGCTCTCAGAAAGCCACATCCGCAATATCGCAAGTTATAGACAAAAGGGAAAAGAAGGCTGCTTCAAGCTTTTCTCGCCAGGAGAGAAAGGCGAATACATGATCAAGCTCGATTTCTACCGGACCCGTCAGCTGAGGAGAGTAGCGAAGGACAATTGGTGGAGGGAAGCGGACTACCGGGCCACGTTCTTGCTTTCTTACACGGATCCTGCAGCAACTCAGA
TAAGAAGATCCTCAAGGAGCAGGTCAAGAAGATAGCAGCGATTCCGGATCGGAAGATCGAATTTTTACAAAACAAAAAGGGAAAAAAATAATTGAATTTTTATCATTATGCGTAATTACCCCTTTTCTCGTATCTTTCAATCCGAGTTGTATCTTTTCGATAAATCGTTATATTACATTACTCCCGAATCTATGGCCTGTCAGTTAGCGATAATGCAACACACTCACACCATTAATAAGATGAGTGTAAA
TCAATTATTTCAGTATCAATTAAACATTCCGATTTCTAAATTATTTTACAATTGTAACGCTCCGGATGAATATTTTT
>18
AAACTTGGAAGCATTCGATTGACGATTATCGATCAGTCTCCCCTGTTGACAGCTTTTCAGGATTACACTCTGGAACATCCGTTGGGTCTATTACCCCTCCCCGTCCGATGGCCGAGTTCGTGGGTTAGTAGAAAGCGACGCATTGACGATATTACGCAAGAACAGAAGGCTCTTTGGCGGGAAGCACAAATTGCTACGAAGAGAGTTTTGAAACCATCTCTTTCGAAGAATTTAAAGATTTATTCTGTGC
AGAATTTAGCCGGTGGATTCTCACAGACGGTACATGCTGCACGATATTTACCCAAATCCGCGTCCCGGCCGCCTTTCGAAATGATCGGGGAATCGTACCCAAGAGCACAGGGAACTCGTCGGCGTCGTGGTCATTCGTCGTGTTCGAGCACGGCAAGCACGTCCATGTCATCCACCTGTGCAGATACGAGAGATCATGGTGCTTGTCTGCGCTCACCAAAAAAGCAGAAGGAAGGTTCGGACGAGAATTG
GAAATATACTCTTCGAAGCAGTAGGCTCTCAGAAAGCCACATCCGCAATATCGCAAGTTATAGACAAAAGGGAAAAGAAGGCTGCTTCAAGCTTTTCTCGCCCAGAGAGAAAGGCGAATACATGATCAAGCTCGATTTCTACCGGACCCGTCAGCTGAGGAGAGTAGCGAAGGACAATGGTGGAGGGAAGCGGACTACCGGCCACGTTCGTGCTTTCTTACACGGATCCTGCAGCAACTCAGATAAAGAA
GATCCTCAAGGAGCAGGTCAAGAAGATAGCAGCGATTCCGGATCGGAAGATCGAATTTTACAAAAAAAAAAGGGAAAAAAAAATTGAATTTTTATCATCATGCGTAATTACCCCTTTTCTCGTATCTTTCAATCCGAGTTGTATCTTTTCGATAAATCGTTATATTACATACTCCCGAATCTATGGCCTGTCAGTTAGCGATAATGCAACACACTCACACCATTAATAAGATGAGTGTAAATCAATTATT
TCAGTATCAATTAAACATTCCGATTCTAAATTATTTACAATTGTAACGCTCCGGATGAATATTTT
>19
AAACTTGGAAGCATTCGATTGACGATTATCGATCAGTCTCCCCTGTTGACAGCTTTTCAGGATTACACTCTGGAACATCCGTTGGGTCTATTACCCCTCACCCCGTCCGATGGCCGAGTTCGTGGGGTTAGTAGAAAGCGACGCATTGACGAATATTACGCAAGAACAGAAGGCTCTTTGGCGGGAAGCACAAATGCTACGAAGAGAGTTTTGAAACCATCTCTTTCGAAGAATTTAAAGATTTATTCTG
TGCAGAATTTAGCCGGGTGGATTCTCACAGACGGTACATGCTGCACGATATTTACCAAATCCGCGTCCCGGCCGCCTTTCTGAAAATGATCGGGAATCGTACCAAGAGCACAGGGAACTCGTCGGCGTCGTGGTCATTCGTCGTGTTCGAGCCGGCAAGCACGTCCATGTCATCCACCTGTGCAGATACGAGATCATGGTGCTGTTCGCGCTCCCAAAAAAGCAGAAGGAAGGTTCGGACGAAGAATTGG
AAGATATACTCTTCGAAGCAGTAGGCTCTCAGAAAGCCACATCCGCAATATCGCAAGTTATAGACAAAAAGGGAAAAGAAGGCTGCTTCAAGCTTTCTCGCCAGGAGAGAAAGGCGAATACATGATCAAGCTCGATTTCTACCGGACCCGTCAGCTGAGGAGAGTAGCGAAGGACAATTGGTGGAGGGAAGCGGACTACCGGGCCACGTTCGTGCTTTCTTACACGGATCCTGCAGCAACTCAGATAAAG
AAGATCCTCAAGGAGCAGGTCAAGAAGATAGCAGCGATTCCGGATCGGAAGAGTCGAATTTTTACAAAAACAAAAAGGGAAAAAAATATTGAATTTTTATCATCATGCGTAATTACCCCTTTTCTCGTATCTTTCAATCCGAGTTGTATCTTTCGATAAATCGTTATATTACATTACTCCCGAATCTATGGCCTGTCAGTTAGCGATAATGCAACACACTCACACCATTAATAAGATGAGTGTAAATCA
TTATTCAGTATCAATTAAACATTCCGATTTCTAAATTATTTACAATTGTAACGCTCCGGATGAATATTTTT
>20
AAACTTGGAAGCATTCGATTGACGATTATCGATCAGTCTCCCCTGTTGACAGCTTTTCAGGATTACACTCTGGAACATCCGTTGGGTCTATTACCCTCCCCCGTCCGATGGCCGAGTTCGTGGGGTTAGTAGAAAGCGACGCATTGACGAATATTACGCAAGAACAGAAGGCTCTTTGCGGGAAGCACAAATTGCTACGAAGAGAGTTTTGAAACCATCTCTTTCGAAGAATTTAAAGATTTATTCTGGC
AGAATTTAGCCGGGTGGATTCTCACAGACGGTACATGCTGCACGATATTTACCCAAATCCGCGTCCCGGCCGCCTTTCTGAAAATGATCGGGGAATCGTACCCAAGAGCACAGGGAACTCGTCGGCGTCGTGGTCATTCGTCGTGTTCGAGCACGGCAAGCACGTCCATGTCATCCACCTGTGCAGATACGAGAGATCATGGTGCTTGTTCGCGCTCACCAAAAAAGCAGAAGGAAGGTCGGGACGAAGA
ATTGGAAGATATACTCTTCGAAGCAGTAGGCTCTCAGAAAGCCACATCCGCAATATCGCAAGTTATAGACAAAAAGGGAAAAGAAGGCTGCTTCAAGCTTTTCTCGCCAGGAGAGAAAGGCGAATACATGATCAAGCTCGATTTCTACCGGACCCGTCAGCTGAGGAGAGTAGCGAAGGACAATTGGTGGAGGGAAGCGGACTACCGGGCCACGTTCGTGCTTTCTTACACGGATCCTGCAGCAACTCAGA
TAAAGAAGATCCTCAAGGAGCAGGTCAAGAAGATAGCAGCGATTCCGGATCGGAAGATCGAATTTTACAAAAACAAAAAGGGAAAAAAATAATTGAATTTTTATCATCATGCGTAATTACCCCTTTTCTCGTATCTTTCAATCCGAGTTGTATCTTTTCGATAAATCGTTATATTACATTACTCCCGAATCTATGGCCTGTCAGTTAGCGATAATGCAACACACTCACACCATAATAAGATGAGTGTAA
ATCAATTATTCAGTATCAATTAAACATTCCGATTTCTAAATTATTTTACAATTGTAACGCTCCGGATGAATATTTTTT
>21
AAACTTGGAAGCATTCGATTGACGATTATCGATCAGTCTCCCCTGTTGACAGCTTTCAGGATTACACTCTGGAACATCCGTTGGGTCTATTACCCCTCCCCGTCCGATGGCCGAGTTCGTGGGGTTAGTAGAAAGCGACGCATTGACGAATATTACGCAAGAACAGAAGGCTCTTTGGCGGGAAGCACAAATTGCTACGAAGAGAGTTTTGAAACCATCTCTTTCGAAGAATTTAAAGATTTATTCTGTG
CAGAATTTAGCCGGGTGGATTCTCACAGACGGTACATGCTGCACGATATTTACCCAAATCCGCGTCCCGGCCGCCTTTCTGAAAATGATCGGGGAATCGTACCCAAGAGCACAGGGAACTCGTCGGCGTCGTGGTCATTCGTCGTGTTCGAGCACGGCAAGCACGTCCATGTCATCCACCTGTGCAGATACGAGAGATCATGGTGCTTGTTCGCGCTCACCAAAAAAGCAGAAGGAAGGTTCGGACGAAGA
ATTGGAAGATATACTCTTCGAAGCAGTAGGCTCTCAGAAAGCCACATCCGCAATATCGCAAGTTATAGACAAAAAGGGAAAAGAAGGCTGCTTCAAGCTTTTCTCGCCAGGAGAGAAAGGCGAATACATGATCAAGCTCGATTTCTACCGGACCCGTCAGCTGAGGAGAGTAGCGAAGGACAATTGGTGGAGGGAAGCGGACTACCGGGCCACGTTCGTGCTTTCTTACACGGATCCTGCAGCAACTCAGA
TAAAGAAGATCCTCAAGGAGCAGGTCAAGAAGATAGCAGCGATTCCGGATCGGAAGATCGAATTTTACAAAAACAAAAAGGGAAAAAAATATTGAATTTTTATCATCATGCGTAATTACCCCTTTTCTCGTATCTTTCAATCCGAGTTGTATCTTTTCGATAAATCGTTATATTACATTACTCCCGAATCTATGGCCTGTCAGTTAGCGATAATGCAACACACTCACACCATTAATAAGATGAGTGTAA
ATCAATTATTTCAGTATCAATTAAACATTCCGATTTCTAAATTATTTTACAATTGTAACGCTCCGGATGAATATTTTTT
>22
AAACTTGGAAGCATTCGATTGACGATTATCGATCAGTCTCCCCTGTGACAGCTTTTCAGGATTACACTCTGGAACATCCGTTGGGTCTATACCCCTCCCCGTCCGATGCCGAGTTCGTGGGGTTAGTAGAAAGCGACGCATTGACGAATATTACGCAAGAACAGAAGGCTCTTTGGCGGGAAGCACAAATTGCTACGAAGAGAGTTTTGAAACCATCTCTTTCGAAGAATTTAAGATTATTCTGTGCAGA
ATTTAGCGGGTGGATTCTCACAGACGGTACATGCTGCACGATATTACCCAAATCCGCGTCCCGGCGCCTTTCTGAAAATGATCGGGGAATCGTACCCAAGAGCACAGGGAACTCGTCGGCGTCGTGGTCATTCGTCGTGTTCGAGCACGGCAAGCACGTCCATGTCATCACCTGTGCAGATACGAGAGATCATGGTGCTTGTTCGCGCTCACCAAAAAGCAGAAGGAAGGTTCGGACGAAGAATTGGAG
ATATACTCTTCGAAGCAGTAGGCTCTCAGAAAGCCACATCCGCAATATCGCAAGTTATAGACAAAAAGGGAAAAGAAGGCTGCTTCAAGCTTTTCGCCAGGAGAGAAAGGCGAATACATGATCAAGCTCGATTTCTACCGGACCCGTCAGCTGAGGAGAGTAGCGAAGGACAATGGTGGAGGGAAGCGGACTACCGGGCCACGTTCGTGTTTTCTTACACGGACCCTGCAGCAACTCAGATAAAGAAGAT
CCTCAAGGAGCAGGTCAAGAAGATAGCAGCGATTCGGATCGGAAGATCGAATTTTTACAAAACAAAAAGGGAAAAAAATAATGAATTTTTATCAATCATGCGTAATTACCCCTTTTCTCGTATCTTTCAATCCGAGTTGTATCTTTTCGATAAATCGTTATATTACATTACTCCCGAATCTATGGCCTGTCAGTTAGCGATAATGCAACACACTCACACCATTAATAAGATGAGTGAAATCAATTATTTC
AGTATCAATTAAACATTCCGATTTCTAAATTATTTACAATTGTAACGCTCCGGATGAATATTTTTT
>23
AAACTTGGAAGCATTCGATTGACGATTATCGATCAGTCTCCCCTGTTGACAGCTTTTCAGGATACACTTGGAACATCCGTTGGGTCTATTACCCCTCCCCCGTCCGATGGCGAGTTCGTGGGGTTAGTAGAAAGCGACGCATTGACGAATTAACGCAAGAACAGAAGGCTCTTTGGCGGGAAGCACAAATTGCTACGAAGAGAGTTTTGAAACCATCTCTTTCGAAGAATTTAAAGATTTTTCTGTGCAG
AATTTAGCCGGGTGGATTCTCAAGACGGTACATGCTGCACGATATTTACCCAAATCCGCGTCCGGCCGCCTTTCTGAAAATGATCGGGGAATCGTACCCAAGAGCACAGGGAACTCGTCGGCGTCGTGGTCATTCGTCGTGTTCGAGCACGGCAAGCACGTCCATGTCATCCACCTGTGCAGATACGAGAGATCATGGTGCTTGTTCGCGCTCACCAAAAAAGCAGAAGGAAGGTTCGGACGAAGAATTG
GAAGATATACTCTTCGAAGCAGTAGGCTCTCAGAAAGCCACATCCGCAATATCGCAAGTTATAGACAAAAAGGGAAAAGAAGGCTGCTTCAAGCTTTTCTCGCCAGGAGAGAAAGGCGAATACATGATCAAGCTCGATTTCTACCGGACCCGTCAGCTGAGGAGAGTAGCGAAGGACAATTGGTGGAGGGAAGCGGACTACCGGGCCACGTTCGTGCTTTTACACGGATCCTGCAGCAACTCAGATAAAG
AAGATCCTCAAGGAGCAGGTCAAGAAGATAGCAGCGATTCCGGATCGGAAGATCGAATTTTTACAAAAACAAAAAGGGAAAAAAATAATTGAATTTTTATCATCATGCGTAATTACCCCTTTTCTCGTATCTTTCAATCCGAGTTGTATCTTTTCGATAAATCGTTATATTACATTACTCCCGAATCTATGGCCTGTCAGTTAGCGATAATGCAACACACTCACACCATTAATAAGATGAGTGTAAATC
AATTATTTCAGTATCAATTAAACATTCCGATTTCTAAATTATTTTACAATTGTAACGCTCCGGATGAATATTTTTT
>24
AAACTTGGAAGCATTCGATTGACGATTATCGATCAGTCTCCCCTGTTGACAGCTTTTCAGGATTACACTCTGGAACATCCGTTGGGTCTATTACCCCTCCCCGTCCGATGGCCGAGTTCGTGGGGTTAGTAGAAAGCGACGCATTGACGAATATTACGCAAGAACAGAAGGCTCTTTGGCGGGAAGCACAAATTGCTACGAAGAGAGTTTTGAAACCATCTCTTTCGAAGAATTTAAAGATTTATTCTGT
GCAGAATTTAGCGGGTGGATTCTCACAGACGGTACATGCTGCACGATATTTACCCAAATCCGCGTCCCGGCCGCCTTTCTGAAAATGATCGGGGAATCGTACCCAAGAGCACAGGGAACTCGTCGGCGTCGTGGTCATTCGTCGTGTTCGAGCACGGCAAGCACGTCCATGTCATCCACCTGTGCAGATACGAGAGATCATGGTGCTTGTTCGCGCTCACCAAAAAAGCAGAAGGAAGGTTCGGACGAAG
AATTGGAAGATATACTCTTCGAAGCAGTAGGCTCTCAGAAAGCCACATCCGCAATATCGCAAGTTATAGACAAAAAGGGAAAAGAAGGCTGCTTCAAGCTTTTCTCGCCAGGAGAGAAAGGCGAATACATGATCAAGCTCGATTTCTACCGGACCCGTCAGCTGAGGAGAGTAGCGAAGGACAATTGGTGGAGGGAAGCGGACTACCGGGCCACGTTCGTGCTTTCTTACACGGATCCTGCAGCAACTCA
GATAAAGAAGATCCTCAAGGAGCAGGTCAAGAAGATAGCAGCGATTCCGGATCGGAAGATCGAATTTTTACAAAAACAAAAAGGGAAAAAAATAATGATTTTTATCATATGCGTAATTACCCTTTTCTCGTATCTTTCAATCCGAGTTGATTCTTTCGATAAATCGTTATATTACATTACTCCCGAATCTATGGCTGTCAGTTAGCGATAATGCAATACACTCACCCATTAATAAGATGAGTGTAACAT
CATTATTTCAGTATCATAAACATTCGATTTCTAAATTATTTTACAATTGTAACGCTCGATGAATAGTTTT
>26
AAACTTGGAAGATTCGATTGACGATTATCGATCAGTCTCCCCTGTTGACAGCTTTTCAGGATTACACTTCGGAACATCCGTTGGTCTATTACCTCCCCCGTCGATGGCCGAGTTCGTGGGGTTAGTAGAAAGCGACCGCATTGAGAATACGTAGAAGCACGAAGGCTCTTGGCGGGAAGCCAAATTGCTACGAAGAGAGTTTTGAAACCAGTCTCTTTCGAAGATTTAAAGATTTATTCTGTGCAGAATT
TAGCCGGGTGGATTCTCACAGACGGTACATGCTGCACGATATTTACCCAAATCCGCGTCCCGGCCGCCTTTCTGAAAATGATCGGGGAATCGTACCCAAGAGCACAGGGAACTCGTCGGCGTCGTGGTCATTCGTCGTGTTCGAGCACGGCAAGCACGTCCATGTCATCCACCTGTGCAGATACGAGAGATCATGGTGCTTGTTCGCGCTCACCAAAAAAGCAGAAGGAAGGTTCGGACGAAGAATTGGA
AGATATACTCTTCGAAGCAGTAGGCTCTCAGAAAGCCACATCCGCAATATCGCAAGTTATAGACAAAAAGGGAAAAGAAGGCTGCTTCAAGCTTTTCTCGCCAGGAGAGAAAGGCGAATACATGATCAAGCTCGATTTCTACCGGACCCGTCAGCTGAGGAGAGTAGCGAAGGACAATTGGTGGAGGGAAGCGGACTACCGGGCCACGTTCGTGCTTTCTTACACGGATCCTGCAGCAACTCAGATAAAG
AAGATCCTCAAGGAGCAGGTCAAGAAGATAGCAGCGATTCCGGATCGGAAGATCGAATTTTACAAAAACAAAAAGGGAAAAAAATAATTGAATTTTTATCATCATGCGTAATTACCCCTTTTCTGTATCTTTCAATCCGAGTTGTATCTTTTCGATAAATCGTTATATTACATTACTCCCGAATCTATGGCCTGTCAGTTAGCGATAATGCAACACACTCACACCATAATAAGATGAGTGTAAATCAATTA
TTTCAGTATCAATTAAACATTCCGATTCTAAATTATTTTACAATTGTAACGCTCCGGATGAATATTTTTT
>27
AAACTTGGAAGCATTCGATTGACGATTATCGATCAGTCTCCCTGTTGACAGCTTTTCAGGATTACACTCTGGAACATCCGTTGGGTCTATTACCCCTCCCCCGTCCGATGGCCGAGTTCGTGGGGTTAGTAGAAAGCGACGCATTGACGAATATTACGCAAGAACAGAAGGCTCTTTGGCGGGAAGCACAAATTGCTACGAAGAGAGTTTTGAAACCATCTCTTTCGAAGAATTTAAAGATTTATTCTGT
GCAGAATTTAGCCGGGTGGATTCTCACAGACGGTACATGCTGCACGATATTTACCCAAATCCGCGTCCCGGCCGCCTTTCTGAAAATGATCGGGGAATCGTACCCAAGAGCACAGGGAACTCGTCGGCGTCGTGGTCATTCGTCGTGTTCGAGCACGGCAAGCACGTCCATGTCATCCACCTGTGCAGATACGAGAGATCATGGTGCTTGTTCGCGCTCACCAAAAAAGCAGAAGGAAGGTTCGGACGAAG
AATTGGAAGATATACTCTTCGAAGCAGTAGGCTCTCAGAAAGCCACATCCGCAATATCGCAAGTTATAGACAAAAAGGGAAAAGAAGGCTGCTTCAAGCTTTTCTCGCCAGGAGAGAAAGGCGAATACATGATCAAGCTCGATTTCTACCGGACCCGTCAGCTGAGGAGAGTAGCGAAGGACAATTGGTGGAGGGAAGCGGACTACCGGGCCACGTTCTTGCTTTCTTACACGGATCCTGCAGCAACTCA
GATAAAGAAGATCCTCAAGGAGCAGGTCAAGAAGATAGCAGCGATTCCGGATCGGAAGATCGAATTTTTACAAAAACAAAAAGGGAAAAAAATAATTGAATTTTTATCATTATGCGTAATTACCCCTTTTCTCGTATCTTTCAATCCGAGTTGTATCTTTTCGATAAATCGTTATATTACATTACTCCCGAATCTATGGCCTGTCAGTTAGCGATAATGCAACACACTCACACCATTAATAAGATGAGT
GTAAATCAATTATTTCAGTATCAATTAAACATTCCGATTTCTAAATTATTTTACAATTGTAACGCTCCGGATGAATATTTTTT
>31
AAACTTGGAAGCATTCGATTGACGATTATCGATCAGTCTCCCCTGTTGACAGCTTTTCAGGATTACACTCTGGAACATCCGTTGGGTCTATTACCCTCCCCCGTCCGATGGCCGAGTTCGTGGGGTTAGTAGAAAGCGACGCATTGACGAATATTACGCAAGAACAGAAGGCTCTTTGGCGGGAAGCACAAATTGCTACGAAGAGAGTTTTGAAACCATCTCTTTCGAAGAATTTAAAGATTTATTCTGT
GCAGAATTAGCCGGGTGGATTCTCACAGACGGTACATGCTGCACGATATTTACCAAATCCGCGTCCCGGCCGCCTTTCTGAAAATGATCGGGGAATCGTACCCAAGAGCACAGGGAACTCGTCGGCGTCGTGGTCATTCGTCGTGTTCGAGCACGGCAAGCACGTCCATGTCATCCACCTGTGCAGATACGAGAGATCATGGTGCTTGTTCGCGCTCACCAAAAAAGCAGAAGGAAGGTTCGGACGAAGAA
TTGGAAGATATACTCTTCGAAGCAGTAGGCTCTCAGAAAGCCACATCCGCAATATCGCAAGTTATAGACAAAAAGGGAAAAGAAGGCTGCTTCAAGCTTTTCTCGCCAGGAGAGAAAGGCGAATACATGATCAAGCTCGATTTCTACCGGACCCGTCAGCTGAGGAGAGTAGCGAAGGACAATTGGTGGAGGGAAGCGGACTACCGGGCCACGTTCGTGCTTTCTTACACGGATCCTGCAGCAACTCAGA
TAAAGAAGATCCTCAAGGAGCAGGTCAAGAAGATAGCAGCGATTCCGGATCGGAAGATCGAATTTTTACAAAAACAAAAAGGGAAAAAAATAATTGAATTTTTATCATCATGCGTAATTACCCCTTTTCTCGTATCTTTCAATCCGAGTTGTATCTTTTCGATAAATCGTTATATTACATTACTCCCGAATCTATGGCCTGTCAGTTAGCGATAATGCAACACACTCACACCATTAATAAGATGAGTGT
AAATCAATTATTTCAGTATCAATTAAACATTCCGATTTCTAAATTATTTTACAATTGTAACGCTCCGGATGAATATTTTTT

Incomplete or rearranged copies
>1
TTATCGATCAGTCTCCCCTGTTGACAGCTTTTCAGGATTACACTCTGGAACATTCGTTGGGTCTATTACCCCTCCCCCGTCTGATGGCCGAGTTCGTGGGGTTAGTAGAAAGCGACGCATTGACGAATATTACGCAAGAACAGAAGGCTCTTTGGCGGGAAGCACAAATTGCTACGAAGAGAGTTTTGAAACCATCTCTTTCGAAGAATTTAAAGATTTATTCTGTGCAGAATTTAGCCGGGTGGATTCTC
ACAGACGGTACATGCTGCACGATATTTACCCAAATCCGCGTCCCGGCCGCCTTTCTGAAAATGATCGGGGAATCGTACCCAAGAGCACAGGGAACTCGTCGGCGTCGTGGTCATTCGTCGTGTTCGAGCACGGCAAGCACGTCCATGTCATCCACCTGTGCAGATACGAGAGATCATGGTGCTTGTTCGCGCTCACCAAAAAAGCAGAAGGAAGGTTCGGACGAAGAATTGGAAGATATACTCTTCGAAGC
AGTAGGCTCTCAGAAAGCCACATCCGCAATATCGCAAGTTATAGACAAAAAGGGAAAAGAAGGCTGCTTCAAGCTTTTCTCGCCAGGAGAGAAAGGCGAATACATGATCAAGCTCGATTTCTACCGGACCCGTCAGCTGAGGAGAGTAGCGAAGGACAATTGGTGGAGGGAAGCGGACTACCGGGCCACGTTCGTGCTTTCTTACACGGATCCTGCAGCAACTCAGATAAAGAAGATCCTCAAGGAGCAG
GTCAAGAAGATAGCAGCGATTCCGGATCGGAAGATCGAATTTTTACAAAAACAAAAAGGGAAAAAAATAATTGAATTTTTATCATCATGCGTAATTACCCCTTTTCTCGTATCTTTCAATCCGAGTTGTATCTTTTCGATAAATCGTTATATTACATTACTCCCGAATCTATGGCCTGTCAGTTAGCGATAATGCAACACACTCACACCATTAATAAGATGAGTGTAAATCAATTATTTCAGTATCAAT
TAAACATTCCGATTTCTAAATTATTTTACAATTGTAACGCTCCGGATGAATATTTTT
>17
AAACTTGGAAGCATTCGATTGACGATTATCGATCAGTCTCCCCTGTTGACAGCTTTTCAGATAACTTGAACTCGTGGTATACCCCCCTCGTGCGAGTTCGGGGGTATAAACGCATGACGATACGCAAGAAGCTCTGGGAGCAAATGTACGAGAGTTGAAACATCTCTTCGAAATTAAGATTCTGTGCATTGCCGGTGGATTCCACGACGTACAGCACTATACCAAACGCCGGCGCTTGAAATGTGGATTA
CCAAGCCAGACTCGTGCGCTTTCGTGGTCGAGCCGGAGGTCTACCTGGCGCGGTCATGGTGTCGCCCAAAAAGAAGGTTCGACGAGATGAATTCGAGCAGGCCTCAGAACCATCCAATACGCATATGCAAAGGAAGAGCTGTCGTTTCTGCAGGGAAGCGAACTGATCATCGATTCTACGACCGCTGAGAGTAGCGAAGCAATGTGGGAAGCGACTACCGGCACGTCTGCTTCTCACGATCCGCAGCAC
TCAGATAGAAGATCCTCAGAGCAGTCAAGATAGCCGATTCGATCGGAAGTCGATTTTACAAACAAAGGAAAAATATGATTTATCATCATGCGTATTACCCCTTCTGTATCTTCAATCGAGTTGTATCTTTTCGTAATCGTTATACATTACTCCCGAATCTAGGCCTTCAGTAGCGATAATGCACACCACACCATATAAGTGAGTAAATATATTCGTATCATAACATCGATTCTAATATTACATTGTAACGC
CCGGATGAATATTTAACTTGGACATTCGATGACGATATCGATCAGTCCCGTGACAGCTTTCAGATTACACTCTGAACATCCTGGCTTTACCCTCCCCGTCGATGCCAGTTCGTGGTAGTAAAAGCGGCATGACGAATATTACCAGAACGAAGCTTTGGCGGGAAGCACAAATTGCTACGAAGAGAGTTTTGAAACCATCTCTTTCGAAGAATTTAAAGATTTATTCTGTGCAGAATTTAGCCGGGTGGAT
TCTCACAGACGGTACATGCTGCACGATATTTACCCAAATCCGCGTCCCGGCCGCCTTTCTGAAAATGATCGGGGAATCGTACCCAAGAGCACAGGGAACTCGTCGGCGTCGTGGTCATTCGTCGTGTTCGAGCACGGCAAGCACGTCCATGTCATCCACCTGTGCAGATACGAGAGATCATGGTGCTTGTTCGCGCTCACCAAAAAAGCAGAAGGAAGGTTCGGACGAAGAATTGGAAGATATACTCTTCG
AAGCAGTAGGCTCTCAGAAAGCCACATCCGCAATATCGCAAGTTATAGACAAAAAGGGAAAAGAAGGCTGCTTCAAGCTTTTCTCGCCAGGAGAGAAAGGCGAATACATGATCAAGCTCGATTTCTACCGGACCCGTCAGCTGAGGAGAGTAGCGAAGGACAATTGGTGGAGGGAAGCGGACTACCGGGCCACGTTCGTGCTTTCTTACACGGATCCTGCAGCAACTCAGATAAAGAAGATCCTCAAGGA
GCAGGTCAAGAAGATAGCAGCGATTCCGGATCGGAAGATCGAATTTTTACAAAAACAAAAAGGGAAAAAAATAATTGAATTTTTATCATCATGCGTAATTACCCCTTTTCTCGTATCTTTCAATCCGAGTTGTATCTTTTCGATAAATCGTTATATTACATTACTCCCGAATCTATGGCCTGTCAGTTAGCGATAATGCAACACACTCACACCATTAATAAGATGAGTGTAATCAATTATTTCAGTATCA
ATTAAACATTCCGATTTCTAAATTATTTTACAATTGTAACGCTCCGGATGAATATTTTT
>25
AAACTTGGAAGCATTCGATTGACGATTTCGATCAGTCCCCCTGTGACAGCTTTCAGGATTACACTCTGGAACATCCGTTGGGTCTATTACCCCTCCCCCGTCCGATGCCAGTTCGTGGGGTTAGTAGAAAGGCGCATTGACGATATACGCAAGAACAGAAGCCTTTGGCGGGAAGCACAAATTCAGAAAGAGTTTGAAACATCTCTTTCGAAGAATTTAAAGATTTATTCTTGCAGAATTTAGACGGGTG
GATTCGACGTACCATGCTGCACGAATTTACCCAAATCCGCGTCCCGGCCGCTTTCTGAAAATGATCGGGGATCGTACCCAAGAGCACAGGAACCTGTCGGCGTCGTGGTCATGCGTCGTGTTCGAGACAGCAAGCACGTCCATTGTCATCCACCTGGCAGATACGAGAGTCATGGTGCTGTTCGCGTCACCAAAAAGCAGAAGGAAGGTTCGGACGAAGAATTGGAAGATATACTCTTCAAGCAGTAGGC
TCTCAGAAGCACATCCGCAATCGCAAGTTATAGACAAAAAGGGAAAAGAAGCTGCTTCAAGCTTTCTCGCCAGGAGAGAAGGAGATACATGATCAAGCTCGATTTCTACCGGACCCGCAGCTGAGAGATGTAGCGAGGACAATTGGTGGAGGGAAGCGGACTACCGGGCCACGTTCGTGCTTTTAACGGATCCTGCGCAACTCAGATAAAGAAATCCTCAAGGAGCAGGTCAAGAAGATAGCAGCGATTCC
GGATCGGAAGATCGAATTTTTACAAAAACAAAAGGAAAAAAATAATGATTTTTATACATGGTAATACCCCTTTTCTCGATTCAATCCGAGTTGTATCTTTACGAAAAATCTTATATTACATACTCCCGATCTATGGCCTGTCAGTTAGCGATAATCAACACATCACACATTAATAAGATGAGTAAATCAATTATTCAGTTACATTAAACATTCGATTTCTAAATATTTTACAATTGTAACGTCCGTATAG
AATATTTTT
>28
AAACTTGGAAGCAAGTATTTTGTGTATTGAAACATTGTTAAAGCATCAAGTTGGAAGCTACGGTTTCGTATATTTTT
>29
GGAAAATCTAATCAGTATCTTAGATAAGCGAATTCCTAAAGTAAATACAATCTTGGTCGAAGGTCAGAGCTTTCCCTTTCACTTCTCCAAGAAGTGTGATAAAAGTGAGATACGCAAAAACCTCTGTTCTCTTGAATTCTGAGGGTCCAGTGAGATTTCTTTCTGGAGGGCCCAAAATATTACCCTCAAGTGAAAGAATCAAATAAATAAATTCCTTACCAAAAACAAAAGAAAAAACCGACAACCCGAT
TTTGAGATAAACCCTCCGAATTAACTTGAAATCATAGGCTGTTTCATTCCAGAGCCTAAAAAGAGGAAAGTCTCTAGGGGAGATGTGCCTGAGTTGGGACGGGTTTGAGTTGGGACAGTTAGTTTTTTCATATGACAGTTGGTAGCAGGACGTCGATTTTTGGTGCGGTGGTACCCCTGAGACATCCAAGTTAACAGACCAGTGCTGCCATTTCCCGGATTTAATTCCGATTTTCGGAACTTTTCGAAT
TTTCGGAATTCTTCCCGGAATTATTTAGATTTTCGGAACTTTTGCTAATAATCGGATTATTTAATGCTCAATCCCGGAATTTTTCAAATTTTCGGAACTTTTCCCGGAATTTTTCAGATTTTCGGAACTTTTGCCAAAAATCCGGATTGATTATACTTTTTAATAATTATTTTCACCGGAACCGTACCATACACATGTCTACTAACATTATATTCCTTATTTCTTGATCTCTTGAAAAATGCCGTGAAAA
TTTAATTTTCTTTCCCATTCGTAGTTGTGGAGCTATCGTGCTGTGATACTAGTTTTAGTTATTTTATTCATTTTTATTTTT
>30
CAAACCCATTCCACCGTCTCTTCTTCACGTGCTCTCATGTCTCATAATGCTTATGCCGTGCCGTGACTGCCGCAAAATTAGTCTTTCCTTTCTCCTCTCTCAATTCTCATTCTCATTCGTATTTTGAGCTATCGTGCAGCGTGCAGTGAAATTTAAAATACTAATTAGTTTTAACCATTGCGATTGGATGCCGTAGTTTCATTCGACTGGACTCACCGGACAGTGTTTCACCAATGTTGTAGGTATGTAA
TATCTGTTTGTTGTGGTAATTTGTAACTTTTGATATAAATCATCTATCCATCTATTAACTAAAGATCTTCGCATATTAGTCACGGCCATACTGATCGGATCAAACTCATTTAAGTACTGATTTAAATCAAAGATCAGCTTTACCTCCAATGAGCCGCTTTGAAAGATGCAGCTCATTCAAAACACGCAGACGTTTCTGTGGAAAGAAATCATTCTGATCAGGCCAGCAGCCTGATTGTTGAAATTTGTGT
CGGCACGACAAGAATCGAAAATCTAGATTTTCGATTGACGATTATCGATCAGTCTCCCTGTTGACAGCTTTTCAGGATTACACTCTGGAACATCCGTTGGGTCTATTACCCCTCCCCCGTCCGATGGCCGAGTTCGTGGGGTTAGTAGAAAGCGACGCATTGACGAATATTACGCAAGAACAGAAGGCTCTTTGGCGGGAAGCACAAATTGCTACGAAGAGAGTTTTGAAACCATCTCTTTCGAAGAATT
TAAAGATTTATTCTGTGCAGAATTTAGCCGGGTGGATTCTCACAGACGGTACATGCTGCACGATATTTACCCAAATCCGCGTCCCGGCCGCCTTTCTGAAAATGATCGGGGAATCGTACCCAAGAGCACAGGGAACTCGTCGGCGTCGTGGTCATTCGTCGTGTTCGAGCACGGCAAGCACGTCCATGTCATCCACCTGTGCAGATACGAGAGATCATGGTGCTTGTTCGCGCTCACCAAAAAAGCAGAA
GGAAGGTTCGGACGAAGAATTGGAAGATATACTCTTCGAAGCAGTAGGCTCTCAGAAAGCCACATCCGCAATATCGCAAGTTATAGACAAAAAGGGAAAAGAAGGCTGCTTCAAGCTTTTCTCGCCAGGAGAGAAAGGCGAATACATGATCAAGCTCGATTTCTACCGGACCCGTCAGCTGAGGAGAGTAGCGAAGGACAATTGGTGGAGGGAAGCGGACTACCGGGCCACGTTCGTGCTTTCTTACACG
GATCCTGCAGCAACTCAGATAAAGAAGATCCTCAAGGAGCAGGTCAAGAAGATAGCAGCGATTCCGGATCGGAAGATCGAATTTTTACAAAAACAAAAAGGGAAAAAAAAATAATTGAATTTTTTCATCATGCGTAATTACCCCTTTTCTCGTATCTTTCAATCCGAGTTGTATCTTTTCGATAAATCGTTATATTACATTACTCCCGAATCTATGGCCTGTCAGTTAGCGATAATGCAACACACTCACA
CCATTAATAAGATGAGTGTAAATCAATTATTTCAGTATCAATTAAACATTCCGATTTCTAAATTATTTTACAATTGTAACGCTCCGGATGAATATTTTT
>32
AAACTTGGAAGCAAGTATTTTGTGTATTGAAACATTGTTAAAGCATCAAGTTGGAAGCTACGGTTTCGTTAATTTTTTGGAAAATCTAATCAGTATCTTAGATAAGCGAATTCCTAAAGTAAATACAATCTTGGTCGAAGGTCCGGCAAATTCGGGTAAAAACTTTTTGATGCGGTTACCCAAGCTATGATAAACTGCGGGCACATCGGAAATTTTAA

Rep endonuclease motifs-containing proteins encoded in the multiple repeats of the partial NS1/Rep gene coding sequence

>1
PGGFSQTVHAARYLPKSASRRLSENDRGIVPKSTGNSSASWSFVVFEHGKHVHVIHLCRYERSWCLFALTKKAEGRFGRRIGRYTLRSSRLSESHIRNIASYRQKGKRRLLQAFLARRERRIHDQARFLPDPSAEESSEGQLVEGSGLPGHVRAFLHGSCSNSDKEDPQGAGQEDSSDSGSEDRIFTKTKREKK
>2
MAEFVGLVESDALTNITQEQKALWREAQIATKRVLKPSLSKNLKIYSVQNLAGWILTDGTCCTIFTQIRVPAAFLKMMGIVPKSTGNSSASWSFVVFEHGKHVHVIHLCRYERSWCLFALTKKQKEGSDEELEDILFEAVGSQKATSAISQVIDKREKKAASSFLARRERRIHDQARFLPDPSAEESSEGQLVEGSGLPGHVRAFLHGPAATQIKKILKEQVKKIAAIPDRKIEFLQNKREKNN
>3
PGGFSRRYMLHDIYPNPRPGRLSENDRGIVPKSTGNRRASWSFVVFEHGKHVHVIHLCRYERSWCLFALTKKAEGRFGRRIGRYTLRSSRLSESHIRNIASYRQKGKEGCFKLFSPGRKANT
>4
SGIVPKSTGNSSASWSFVVFEHGKHVHVIHLCRYERSWCLFALTKKAEGRFGRRIGRYTLRSSRLSESHIRNIASYRQKGKEGCFKLFSPGEKANT
>5
TWKHSIDDYRSVSPVDSFSGLHSGTSVGSITPPPSDGRVRGVSRKRRIDEYYARTEGSLAGSTNCYEESFETISFEEFKDLFCAEFSRVDSHRRYMLHDIYPNPRPGRLSENDRGIVPKSTGNSSASWSFVVFEHGKHVHVIHLCRYERSWCLFALTKKAEGRFGRRIGRYTLRSSRLSESHIRNIASYRQKGKRGCFKLFSPGEKGEYMIKLDFYRTRQLRRVAKDNWWREADYRATFVLSYTDPAATQ
IKKILKEQVKKIAAIPDRKIEFLQKQKGKKNN
>6
QLFRITLWNIRWSITPPPSDGRVRGVSRKRRIDEYYARTEGSLAGSTNCYEESFETISFEEFKDLFCAEFSRVDSHRRYMLHDIYPNPRPGRLSENDRGIVPKSTGNSSASWSFVVFEHGKHVHVIHLCRYERSWCLFALTKKAEEGSDEELEDILFEAVGSQKATSAISQVIDKKGKEGCFKLFSPGEKANT
>7
QLSGLHSGTSVGSITPPPSDGRVRGVSRKRRIDEYYARTEGSLAGSTNCYEESFETISFEEFKDLFCAEFSRVDSHRRYMLHDIYPNPRPGRLSENDRGIVPKSTGNSSASWSFVVFEHGKHVHVIHLCRYERSWCLFALTKKAEGRFGRRIGRYTLRSSRLSESHIRNIASYRQKGKKAASSFSRQERKANT
>8
RFILCRFSRVDSHRRYMLHDIYPNPRPGRLSENDRGIVPKSTGNSSASWSFVVFEHGKHVHVIHLCRYERSWCLFALTKKQKEGSDEELEEYSSKQ
>9
SGIVPKSTGNSSASWSFVVFEHGKHVHVIHLCRYERSWCLFALTKKAEGRFGRRIGRYTLRSSRLSESHIRNIASYRQKGKEGCFKLFSPGEKGEYMIKLDFYRTRQLRRVAKDNW
>10
QLFRITLWNIRWVYYPSPVRWPSSWVSRKRRIDEYYARTEGSLAGSTNCYEESFETISFEEFKDLFCAEFSRVDSHRRYMLHDIYPNPRPGRLSENDRGIVPKSTGNSSASWSFVVFEHGKHVHVIHLCRYERSWCLFALTKKAEGRFGRRIGRYTLRSSRLSESHIRNIASYRQKGKRRLLQAFLARRERRIHDQARFLPDPSAEESSEGQLVEGSGLPGHVRAFLHGSCSNSDKRSSRSRSRDSSDSG
SEDRIFTKTKREKKY
>11
NHPFEEFKDLFCAEFSRVDSHRRYMLHDIYPNPRPGRLSENDRGIVPKSTGNSSASWSFVVFEHGKHVHVIHLCRYERSWCLFALTKKAEGRFGRRIGRYTLRSSRLSESHIRNIASYRQKGKRRLLQAFLARRERRIHDQARFLPDPSAEESSEGQLVEGSGLRATFVLSYTDPAATQIKKILKEQVKKIAAIPDRKIDFYKNKKGKKIIEFLSSCVITPFLVSFNPSCIFSINRYITLLPNLWPVS
>12
TWKHSIDDYRSVSPVDSFSGLHSGTSVGSITPPPSDGRVRGVSRKRRIDEYYARTEGSLAGSTNCYEESFETISFEEFKDLFCAEFSRVDSHRRYMLHDIYPNPRPGRLSENDRGIVPKSTGNSSASWSFVVFEHGKHVHVIHLCRYERSWCLFALTKKAEGRFGRRIGRYTLRSSRLSESHIRNIASYRQKGKRRLLQAFLARRERRIHDQARFLPDPSAEESSEGQLWREADYRATFVLSYTDPAATQ
IKKILKEQVKKIAAIPDRKIEFLQKQKGKKIIDFYHHA
>13
PVDSHRRYMLHDIYPNPRPGRLSENDRGIVPKSTGNSSASWSFVVFEHGKHVHVIHLCRYERSWCLFALTKKAEGRFGRRIGRYTLRSSRLSESHIRNIASYRQKGKRRLLQAFLARRERRIHDQARFLPDPSAEESSEGQLVEGSGLPGHVRAFLHGSCSNSDKEDPQGAGQEDSSDSGSEDRIFTKTKREKNN
>14
QLFRITLWNIRWVYYPSPSDGRVRGVSRKRRIDEYYARTEGSLAGSTNCYEESFETISFEEFKDLFCAEFSRVDSHRRYMLHDIYPNPRPGRLSENDRGIVPKSTGNSSASWSFVVFEHGKHVHVIHLCRYERSWCLFALTKKAEGRFGRRIGRYTLRSSRLSESHIRNIASYRQKGKRRLLQAFLAGRERRIHDQARFLPDPSAEESSEGQLVEGSGLPGHVRAFLHGSCSNSDKEDPQGAGQEDSSDS
GSEDRIFTKTKGKKNN
>15
SGNRTQSTGNSSASWSFVVFEHGKHVHVIHLCRYERSWCLFALTKKAEGRFGRRIGRYTLRSSRLSESHIRNIASYRQKGKEGCFKLFSPGEKGEYMIKLDFYRTRQLRRVAKDNWWREADYRATFVLSYTDPAATQIKKILKEQVKKIAAIPDRKIEFLQKQKGKKNN
>16
FSQTVHAARYYPNPRPGRLSENDRGIVPKSTGNSSASWSFVVFEHGKHVHVIHLCRYERSWCLFALTKKAEGRFGRRIGRYTLRSSRLSESHIRNIASYRQKGKEGCFKLFSPGEKGEYMIKLDFYRTRQLRRVAKDNWWREADYRATFLLSYTDPAATQIRRSSRSRSRR
>17
KRHDEYYQNEALAGSTNCYEESFETISFEEFKDLFCAEFSRVDSHRRYMLHDIYPNPRPGRLSENDRGIVPKSTGNSSASWSFVVFEHGKHVHVIHLCRYERSWCLFALTKKQKEGSDEELEDILFEAVGSQKATSAISQVIDKKGKEGCFKLFSPGEKGEYMIKLDFYRTRQLRRVAKDNWWREADYRATFVLSYTDPAATQIKKILKEQVKKIAAIPDRKIEFLQKQKGKKIIEFLSSCVITPFLVSF
NPSCIFSINRYITLLPNLWPVS
>18
RYYARTEGSLAGSTNCYEESFETISFEEFKDLFCAEFSRWILTDGTCCTIFTQIRVPAAFRNDRGIVPKSTGNSSASWSFVVFEHGKHVHVIHLCRYERSWCLSALTKKAEGRFGRELEIYSSKQ
>19
PGGFSQTVHAARYLPNPRPGRLSENDRESYQEHRELVGVVVIRRVRAGKHVHVIHLCRYEIMVLFALPKKQKEGSDEELEDILFEAVGSQKATSAISQVIDKKGKEGCFKLSRQERKANT
>20
RFILAEFSRVDSHRRYMLHDIYPNPRPGRLSENDRGIVPKSTGNSSASWSFVVFEHGKHVHVIHLCRYERSWCLFALTKKAEGRSGRRIGRYTLEAVGSQKATSAISQVIDKKGKEGCFKLFSPGEKGEYMIKLDFYRTRQLRRVAKDNWWREADYRATFVLSYTDPAATQIKKILKEQVKKIAAIPDRKIEFYKNKKGKKIIEFLSSCVITPFLVSFNPSCIFSINRYITLLPNLWPVS
>21

```
KLGSIRLTIIDQSPLLTAFRITLWNIRWVYYPSPSDGRVRGVSRKRRIDEYYARTEGSLAGSTNCYEESFETISFEEFKDLFCAEFSRVDSHRRYMLHDIYPNPRPGRLSENDRGIVPKSTGNSSASWSFVVFEHGKHVHVIHLCRYERSWCLFALTKKQKEGSDEELEDILFEAVGSQKPHPQYRKL
>22
TWKHSIDDYRSVSPVTAFQDYTLEHPLGLYPSPSDAEFVGLVESDALTNITQEQKALWREAQIATKRVLKPSLSKNLRLFCAEFSGWILTDGTCCTILPKSASRRLSENDRGIVPKSTGNSSASWSFVVFEHGKHVHVITCADTRDHGACSRSPKSRRKVRTKNWKIYSSKQ
>23
RINARTEGSLAGSTNCYEESFETISFEEFKDFSVQNLAGWILKTVHAARYLPKSASGRLSENDRGIVPKSTGNSSASWSFVVFEHGKHVHVIHLCRYERSWCLFALTKKAEGRFGRRIGRYTLRSSRLSESHIRNIASYRQKGKRRLLQAFLARRERRIHDQARFLPDPSAEESSEGQLVEGSGLPGHVRAFTRILQQLR
>24
RVDSHRRYMLHDIYPNPRPGRLSENDRGIVPKSTGNSSASWSFVVFEHGKHVHVIHLCRYERSWCLFALTKKAEGRFGRRIGRYTLRSSRLSESHIRNIASYRQKGKRRLLQAFLARRERRIHDQARFLPDPSAEESSEGQLVEGSGLPGHVRAFLHGSCSNSDKEDPQGAGQEDSSDSGSEDRIFTKTKREKK
>26
KATALRIRRSTKALGGKPNCYEESFETSLFRRFKDLFCAEFSRVDSHRRYMLHDIYPNPRPGRLSENDRGIVPKSTGNSSASWSFVVFEHGKHVHVIHLCRYERSWCLFALTKKAEGRFGRRIGRYTLRSSRLSESHIRNIASYRQKGKRRLLQAFLARRERRIHDQARFLPDPSAEESSEGQLVEGSGLPGHVRAFLHGSCSNSDKEDPQGAGQEDSSDSGSEDRILQKQKGKKIIEFYHHA
>27
PGGFSQTVHAARYYPNPRPGRLSENDRGIVPKSTGNSSASWSFVVFEHGKHVHVIHLCRYERSWCLFALTKKAEGRFGRRIGRYTLRSSRLSESHIRNIASYRQKGKRRLLQAFLARRERRIHDQARFLPDPSAEESSEGQLVEGSGLPGHVLAFLHGSCSNSDKEDPQGAGQEDSSDSGSEDRIFTKTKREKK
>31
QLFRITLWNIRWVYYPPPSDGRVRGVSRKRRIDEYYARTEGSLAGSTNCYEESFETISFEEFKDLFCAELAGWILTDGTCCTILPNPRPGRLSENDRGIVPKSTGNSSASWSFVVFEHGKHVHVIHLCRYERSWCLFALTKKAEGRFGRRIGRYTLRSSRLSESHIRNIASYRQKGKRRLLQAFLARRERRIHDQARFLPDPSAEESSEGQLVEGSGLPGHVRAFLHGSCSNSDKEDPQGAGQEDSSDSG
SEDRIFTKTKREKK
```

```
22              SENDRGIVPKSTGNSSASWSFVVFEH---GKHVHVIT-CADTRDHGACSRSPKS------
2               FLKMMGIVPKSTGNSSASWSFVVFEH---GKHVHVIHLCRYERSWCLFALTKKQKEGSDE
19              SENDR----ESYQEHRELVGVVVIRRVRAGKHVHVIHLCRYEIM-VLFALPKKQKEGSDE
20              SENDRGIVPKSTGNSSASWSFVVFEH---GKHVHVIHLCRYERSWCLFALTKKAEGRSGR
8               SENDRGIVPKSTGNSSASWSFVVFEH---GKHVHVIHLCRYERSWCLFALTKKQKEGSDE
21              SENDRGIVPKSTGNSSASWSFVVFEH---GKHVHVIHLCRYERSWCLFALTKKQKEGSDE
6               SENDRGIVPKSTGNSSASWSFVVFEH---GKHVHVIHLCRYERSWCLFALTKKAEEGSDE
17              SENDRGIVPKSTGNSSASWSFVVFEH---GKHVHVIHLCRYERSWCLFALTKKQKEGSDE
18              FRNDRGIVPKSTGNSSASWSFVVFEH---GKHVHVIHLCRYERSWCLSALTKKAEGRFGR
23              SENDRGIVPKSTGNSSASWSFVVFEH---GKHVHVIHLCRYERSWCLFALTKKAEGRFGR
1               SENDRGIVPKSTGNSSASWSFVVFEH---GKHVHVIHLCRYERSWCLFALTKKAEGRFGR
27              SENDRGIVPKSTGNSSASWSFVVFEH---GKHVHVIHLCRYERSWCLFALTKKAEGRFGR
10              SENDRGIVPKSTGNSSASWSFVVFEH---GKHVHVIHLCRYERSWCLFALTKKAEGRFGR
26              SENDRGIVPKSTGNSSASWSFVVFEH---GKHVHVIHLCRYERSWCLFALTKKAEGRFGR
31              SENDRGIVPKSTGNSSASWSFVVFEH---GKHVHVIHLCRYERSWCLFALTKKAEGRFGR
14              SENDRGIVPKSTGNSSASWSFVVFEH---GKHVHVIHLCRYERSWCLFALTKKAEGRFGR
13              SENDRGIVPKSTGNSSASWSFVVFEH---GKHVHVIHLCRYERSWCLFALTKKAEGRFGR
24              SENDRGIVPKSTGNSSASWSFVVFEH---GKHVHVIHLCRYERSWCLFALTKKAEGRFGR
3               SENDRGIVPKSTGNRRASWSFVVFEH---GKHVHVIHLCRYERSWCLFALTKKAEGRFGR
15              ----SGNRTQSTGNSSASWSFVVFEH---GKHVHVIHLCRYERSWCLFALTKKAEGRFGR
4               ----SGIVPKSTGNSSASWSFVVFEH---GKHVHVIHLCRYERSWCLFALTKKAEGRFGR
5               SENDRGIVPKSTGNSSASWSFVVFEH---GKHVHVIHLCRYERSWCLFALTKKAEGRFGR
9               ----SGIVPKSTGNSSASWSFVVFEH---GKHVHVIHLCRYERSWCLFALTKKAEGRFGR
16              SENDRGIVPKSTGNSSASWSFVVFEH---GKHVHVIHLCRYERSWCLFALTKKAEGRFGR
11              SENDRGIVPKSTGNSSASWSFVVFEH---GKHVHVIHLCRYERSWCLFALTKKAEGRFGR
12              SENDRGIVPKSTGNSSASWSFVVFEH---GKHVHVIHLCRYERSWCLFALTKKAEGRFGR
7               SENDRGIVPKSTGNSSASWSFVVFEH---GKHVHVIHLCRYERSWCLFALTKKAEGRFGR
                         :*  :     ..**: .   *******  *         : . *

2               FLKMMGIVPKSTGNSSASWSFVVFEHGKHVHVIHLCRYERSWCLFALTKKQKEGSDEELE
20              SENDRGIVPKSTGNSSASWSFVVFEHGKHVHVIHLCRYERSWCLFALTKKAEGRSGRRIG
8               SENDRGIVPKSTGNSSASWSFVVFEHGKHVHVIHLCRYERSWCLFALTKKQKEGSDEELE
21              SENDRGIVPKSTGNSSASWSFVVFEHGKHVHVIHLCRYERSWCLFALTKKQKEGSDEELE
6               SENDRGIVPKSTGNSSASWSFVVFEHGKHVHVIHLCRYERSWCLFALTKKAEEGSDEELE
17              SENDRGIVPKSTGNSSASWSFVVFEHGKHVHVIHLCRYERSWCLFALTKKQKEGSDEELE
18              FRNDRGIVPKSTGNSSASWSFVVFEHGKHVHVIHLCRYERSWCLSALTKKAEGRFGRELE
23              SENDRGIVPKSTGNSSASWSFVVFEHGKHVHVIHLCRYERSWCLFALTKKAEGRFGRRIG
1               SENDRGIVPKSTGNSSASWSFVVFEHGKHVHVIHLCRYERSWCLFALTKKAEGRFGRRIG
27              SENDRGIVPKSTGNSSASWSFVVFEHGKHVHVIHLCRYERSWCLFALTKKAEGRFGRRIG
10              SENDRGIVPKSTGNSSASWSFVVFEHGKHVHVIHLCRYERSWCLFALTKKAEGRFGRRIG
26              SENDRGIVPKSTGNSSASWSFVVFEHGKHVHVIHLCRYERSWCLFALTKKAEGRFGRRIG
31              SENDRGIVPKSTGNSSASWSFVVFEHGKHVHVIHLCRYERSWCLFALTKKAEGRFGRRIG
14              SENDRGIVPKSTGNSSASWSFVVFEHGKHVHVIHLCRYERSWCLFALTKKAEGRFGRRIG
13              SENDRGIVPKSTGNSSASWSFVVFEHGKHVHVIHLCRYERSWCLFALTKKAEGRFGRRIG
24              SENDRGIVPKSTGNSSASWSFVVFEHGKHVHVIHLCRYERSWCLFALTKKAEGRFGRRIG
3               SENDRGIVPKSTGNRRASWSFVVFEHGKHVHVIHLCRYERSWCLFALTKKAEGRFGRRIG
15              ----SGNRTQSTGNSSASWSFVVFEHGKHVHVIHLCRYERSWCLFALTKKAEGRFGRRIG
4               ----SGIVPKSTGNSSASWSFVVFEHGKHVHVIHLCRYERSWCLFALTKKAEGRFGRRIG
5               SENDRGIVPKSTGNSSASWSFVVFEHGKHVHVIHLCRYERSWCLFALTKKAEGRFGRRIG
9               ----SGIVPKSTGNSSASWSFVVFEHGKHVHVIHLCRYERSWCLFALTKKAEGRFGRRIG
16              SENDRGIVPKSTGNSSASWSFVVFEHGKHVHVIHLCRYERSWCLFALTKKAEGRFGRRIG
11              SENDRGIVPKSTGNSSASWSFVVFEHGKHVHVIHLCRYERSWCLFALTKKAEGRFGRRIG
12              SENDRGIVPKSTGNSSASWSFVVFEHGKHVHVIHLCRYERSWCLFALTKKAEGRFGRRIG
7               SENDRGIVPKSTGNSSASWSFVVFEHGKHVHVIHLCRYERSWCLFALTKKAEGRFGRRIG
                    *  .:****  *************************** ***** :   .  :

23              IVPKSTGNSSASWSFVVFEHGKHVHVIHLCRYERSWCLFALTKKAEGRFGRRIGRYTLRSSRLSESHIRNIASYRQKGKRRLLQAFLARRERRIHDQARFLPDPSAEESSEGQLVEGSGL
1               IVPKSTGNSSASWSFVVFEHGKHVHVIHLCRYERSWCLFALTKKAEGRFGRRIGRYTLRSSRLSESHIRNIASYRQKGKRRLLQAFLARRERRIHDQARFLPDPSAEESSEGQLVEGSGL
27              IVPKSTGNSSASWSFVVFEHGKHVHVIHLCRYERSWCLFALTKKAEGRFGRRIGRYTLRSSRLSESHIRNIASYRQKGKRRLLQAFLARRERRIHDQARFLPDPSAEESSEGQLVEGSGL
10              IVPKSTGNSSASWSFVVFEHGKHVHVIHLCRYERSWCLFALTKKAEGRFGRRIGRYTLRSSRLSESHIRNIASYRQKGKRRLLQAFLARRERRIHDQARFLPDPSAEESSEGQLVEGSGL
26              IVPKSTGNSSASWSFVVFEHGKHVHVIHLCRYERSWCLFALTKKAEGRFGRRIGRYTLRSSRLSESHIRNIASYRQKGKRRLLQAFLARRERRIHDQARFLPDPSAEESSEGQLVEGSGL
31              IVPKSTGNSSASWSFVVFEHGKHVHVIHLCRYERSWCLFALTKKAEGRFGRRIGRYTLRSSRLSESHIRNIASYRQKGKRRLLQAFLARRERRIHDQARFLPDPSAEESSEGQLVEGSGL
14              IVPKSTGNSSASWSFVVFEHGKHVHVIHLCRYERSWCLFALTKKAEGRFGRRIGRYTLRSSRLSESHIRNIASYRQKGKRRLLQAFLAGRERRIHDQARFLPDPSAEESSEGQLVEGSGL
13              IVPKSTGNSSASWSFVVFEHGKHVHVIHLCRYERSWCLFALTKKAEGRFGRRIGRYTLRSSRLSESHIRNIASYRQKGKRRLLQAFLARRERRIHDQARFLPDPSAEESSEGQLVEGSGL
24              IVPKSTGNSSASWSFVVFEHGKHVHVIHLCRYERSWCLFALTKKAEGRFGRRIGRYTLRSSRLSESHIRNIASYRQKGKRRLLQAFLARRERRIHDQARFLPDPSAEESSEGQLVEGSGL
3               IVPKSTGNRRASWSFVVFEHGKHVHVIHLCRYERSWCLFALTKKAEGRFGRRIGRYTLRSSRLSESHIRNIASYRQKGKEGCFKLFSPGRKANT-------------------------
15              NRTQSTGNSSASWSFVVFEHGKHVHVIHLCRYERSWCLFALTKKAEGRFGRRIGRYTLRSSRLSESHIRNIASYRQKGKEGCFKLFSPGEKGEYMIKLDFYRTRQLRRVAKDNWWREADY
4               IVPKSTGNSSASWSFVVFEHGKHVHVIHLCRYERSWCLFALTKKAEGRFGRRIGRYTLRSSRLSESHIRNIASYRQKGKEGCFKLFSPGEKANT-------------------------
5               IVPKSTGNSSASWSFVVFEHGKHVHVIHLCRYERSWCLFALTKKAEGRFGRRIGRYTLRSSRLSESHIRNIASYRQKGKRGCFKLFSPGEKGEYMIKLDFYRTRQLRRVAKDNWWREADY
9               IVPKSTGNSSASWSFVVFEHGKHVHVIHLCRYERSWCLFALTKKAEGRFGRRIGRYTLRSSRLSESHIRNIASYRQKGKEGCFKLFSPGEKGEYMIKLDFYRTRQLRRVAKDNW------
16              IVPKSTGNSSASWSFVVFEHGKHVHVIHLCRYERSWCLFALTKKAEGRFGRRIGRYTLRSSRLSESHIRNIASYRQKGKEGCFKLFSPGEKGEYMIKLDFYRTRQLRRVAKDNWWREADY
11              IVPKSTGNSSASWSFVVFEHGKHVHVIHLCRYERSWCLFALTKKAEGRFGRRIGRYTLRSSRLSESHIRNIASYRQKGKRRLLQAFLARRERRIHDQARFLPDPSAEESSEGQLVEGSGL
12              IVPKSTGNSSASWSFVVFEHGKHVHVIHLCRYERSWCLFALTKKAEGRFGRRIGRYTLRSSRLSESHIRNIASYRQKGKRRLLQAFLARRERRIHDQARFLPDPSAEESSEGQLWREADY
7               IVPKSTGNSSASWSFVVFEHGKHVHVIHLCRYERSWCLFALTKKAEGRFGRRIGRYTLRSSRLSESHIRNIASYRQKGKKAA--SSFSRQERKANT-----------------------
                  .:****  ********************************************************************         .  :

BtaDV_NS1       RGVVPKDTGNSSSAWTFVVFQHGQHVHVIHKCRYERSWCSCALTKKAEERFGRRIGRHTLRGSRITEGHLEHLANYIQKGRRRLLQAFLAGRERRLSRQAGLLQDTQSSQSGKGELVEGG
11              RGIVPKSTGNSSASWSFVVFEHGKHVHVIHLCRYERSWCLFALTKKAEGRFGRRIGRYTLRSSRLSESHIRNIASYRQKGKRRLLQAFLARRERRIHDQARFLPDPSAEESSEGQLVEGS
23              RGIVPKSTGNSSASWSFVVFEHGKHVHVIHLCRYERSWCLFALTKKAEGRFGRRIGRYTLRSSRLSESHIRNIASYRQKGKRRLLQAFLARRERRIHDQARFLPDPSAEESSEGQLVEGS
1               RGIVPKSTGNSSASWSFVVFEHGKHVHVIHLCRYERSWCLFALTKKAEGRFGRRIGRYTLRSSRLSESHIRNIASYRQKGKRRLLQAFLARRERRIHDQARFLPDPSAEESSEGQLVEGS
27              RGIVPKSTGNSSASWSFVVFEHGKHVHVIHLCRYERSWCLFALTKKAEGRFGRRIGRYTLRSSRLSESHIRNIASYRQKGKRRLLQAFLARRERRIHDQARFLPDPSAEESSEGQLVEGS
10              RGIVPKSTGNSSASWSFVVFEHGKHVHVIHLCRYERSWCLFALTKKAEGRFGRRIGRYTLRSSRLSESHIRNIASYRQKGKRRLLQAFLARRERRIHDQARFLPDPSAEESSEGQLVEGS
26              RGIVPKSTGNSSASWSFVVFEHGKHVHVIHLCRYERSWCLFALTKKAEGRFGRRIGRYTLRSSRLSESHIRNIASYRQKGKRRLLQAFLARRERRIHDQARFLPDPSAEESSEGQLVEGS
31              RGIVPKSTGNSSASWSFVVFEHGKHVHVIHLCRYERSWCLFALTKKAEGRFGRRIGRYTLRSSRLSESHIRNIASYRQKGKRRLLQAFLARRERRIHDQARFLPDPSAEESSEGQLVEGS
14              RGIVPKSTGNSSASWSFVVFEHGKHVHVIHLCRYERSWCLFALTKKAEGRFGRRIGRYTLRSSRLSESHIRNIASYRQKGKRRLLQAFLAGRERRIHDQARFLPDPSAEESSEGQLVEGS
13              RGIVPKSTGNSSASWSFVVFEHGKHVHVIHLCRYERSWCLFALTKKAEGRFGRRIGRYTLRSSRLSESHIRNIASYRQKGKRRLLQAFLARRERRIHDQARFLPDPSAEESSEGQLVEGS
24              RGIVPKSTGNSSASWSFVVFEHGKHVHVIHLCRYERSWCLFALTKKAEGRFGRRIGRYTLRSSRLSESHIRNIASYRQKGKRRLLQAFLARRERRIHDQARFLPDPSAEESSEGQLVEGS
                **:***.*****::*:****:**:****** ********  ******* ********:***.**::*.*: ::*.* ***.********* ****:  ** :* *..:.:*.:*:****.
```

CLUSTAL multiple sequence alignment by MUSCLE (3.8)

```
25              AAACTTGGAAGCATTCGATTGACGATT-TCGATCAGTC-CCCCTG-TGACAGCT-TTCAG
26              AAACTTGGAAG-ATTCGATTGACGATTATCGATCAGTCTCCCCTGTTGACAGCTTTTCAG
24              AAACTTGGAAGCATTCGATTGACGATTATCGATCAGTCTCCCCTGTTGACAGCTTTTCAG
18              AAACTTGGAAGCATTCGATTGACGATTATCGATCAGTCTCCCCTGTTGACAGCTTTTCAG
22              AAACTTGGAAGCATTCGATTGACGATTATCGATCAGTCTCCCCTG-TGACAGCTTTTCAG
14              AAACTTGGAAGCATTCGATTGACGATTATCGATCAGTCTCCCCTGTTGACAGCTTTTCAG
16              AAACTTGGAAGCATTCGATTGACGATTATCGATCAGTCTCCCCTGTTGACAGCTTTTCAG
20              AAACTTGGAAGCATTCGATTGACGATTATCGATCAGTCTCCCCTGTTGACAGCTTTTCAG
27              AAACTTGGAAGCATTCGATTGACGATTATCGATCAGTCT-CCCTGTTGACAGCTTTTCAG
2               AAACTTGGAAGCATTCGATTGACGATTATCGATCAGTCTCCCCTGTTGACAGCTTTTCAG
7               AAACTTGGAAGCATTCGATTGACGATTATCGATCAGTCTCCCCTGTTGACAGCT-TTCAG
23              AAACTTGGAAGCATTCGATTGACGATTATCGATCAGTCTCCCCTGTTGACAGCTTTTCAG
3               AAACTTGGAAGCATTCGATTGACGATTATCGATCAGTCT-CCCTGTTGACAGCTTTTCAG
31              AAACTTGGAAGCATTCGATTGACGATTATCGATCAGTCTCCCCTGTTGACAGCTTTTCAG
19              AAACTTGGAAGCATTCGATTGACGATTATCGATCAGTCTCCCCTGTTGACAGCTTTTCAG
8               AAACTTGGAAGCATTCGATTGACGATTATCGATCAGTCT-CCCTGTTGACAGCTTTTCAG
4               AAACTTGGAAGCATTCGATTGACGATTATCGATCAGTCTCCCCTGTTGACAGCTTTTCAG
11              AAACTTGGAAGCATTCGATTGACGATTATCGATCAGTC-CCCCTGTTGACAGCTTTTCAG
10              AAACTTGGAAGCATTCGATTGACGATTATCGATCAGTCTCCCCTGTTGACAGCTTTTCAG
9               AAACTTGGAAGCATTCGATTGACGATTATCGATCAGTCTCCCCTGTTGACAGCTTTTCAG
6               AAACTTGGAAGCATTCGATTGACGATTATCGATCAGTCTCCCCTGTTGACAGCTTTTCAG
21              AAACTTGGAAGCATTCGATTGACGATTATCGATCAGTCTCCCCTGTTGACAGCT-TTCAG
12              AAACTTGGAAGCATTCGATTGACGATTATCGATCAGTCTCCCCTGTTGACAGCTTTTCAG
15              AAACTTGGAAGCATTCGATTGACGATTATCGATCAGTCTCCCCTGTTGACAGCTTTTCAG
5               AAACTTGGAAGCATTCGATTGACGATTATCGATCAGTCTCCCCTGTTGACAGCTTTTCAG
13              AAACTTGGAAGCATTCGATTGACGATTATCGATCAGTCTCCCCTGTTGACAGCTTTTCAG
                *********** ************** **********  ***** ******** *****

25              GATTACACTCTGGAACATCCGTTGGGTCTATTACCCCTC-CCCCGTCCGATG--CCAGTT
26              GATTACACTTCGGAACATCCGTT-GGTCTATTACCTC---CCCCGT-CGATGGCCGAGTT
24              GATTACACTCTGGAACATCCGTTGGGTCTATTACCCCT--CCCCGTCCGATGGCCGAGTT
18              GATTACACTCTGGAACATCCGTTGGGTCTATTACCCCT--CCCCGTCCGATGGCCGAGTT
22              GATTACACTCTGGAACATCCGTTGGGTCTA-TACCCCT--CCCCGTCCGATG-CCGAGTT
14              GATTACACTCTGGAACATCCGTTGGGTCTATTACCCCT--CCCCGTCCGATGGCCGAGTT
16              GA-TACACTCTGGAACATCCGTTGGGTCTATTACCCCT--CCCCGTCCGATGGCCGAGTT
20              GATTACACTCTGGAACATCCGTTGGGTCTATTACCCTC--CCCCGTCCGATGGCCGAGTT
27              GATTACACTCTGGAACATCCGTTGGGTCTATTACCCCTC-CCCCGTCCGATGGCCGAGTT
2               GATTACACTCTGGAACATCCGTTGGGTCTATTACCCCTC-CCCCGTCCGATGGCCGAGTT
7               GATTACACTCTGGAACATCCGTTGGGTCTATTACCCCTC-CCCCGTCCGATGGCCGAGTT
23              GA-TACACT-TGGAACATCCGTTGGGTCTATTACCCCTC-CCCCGTCCGATGG-CGAGTT
3               GATTACACTCTGGAACATCCGTTGGGTCTATTACCCCTC-CCCCGTCCGATGGCCGAGTT
31              GATTACACTCTGGAACATCCGTTGGGTCTATTACCCTC--CCCCGTCCGATGGCCGAGTT
19              GATTACACTCTGGAACATCCGTTGGGTCTATTACCCCTCACCCCGTCCGATGGCCGAGTT
8               GATTACACTCTGGAACATCCGTTGGGTCTATTACCCCTC-CCCCGTCCGATGGCCGAGTT
4               GATTACACTCTGGAACATCCGTTGGGTCTATTACCCCT--CCCCGTCCGATGGCCGAGTT
11              GATTACACTCTGGAACATCCGTTGGGTCTATTACCCCTC-CCCCGTCCGATGGCCGAGTT
10              GATTACACTCTGGAACATCCGTTGGGTCTATTACCCCTC-CCCCGTCCGATGGCCGAGTT
9               GATTACACTCTGGAACATCCGTTGGGTCTATTACCCCTC-CCCCGTCCGATGGCCGAGTT
6               GATTACACTCTGGAACATCCGTT-GGTCTATTACCCCTC-CCCCGTCCGATGGCCGAGTT
21              GATTACACTCTGGAACATCCGTTGGGTCTATTACCCCT--CCCCGTCCGATGGCCGAGTT
12              GATTACACTCTGGAACATCCGTTGGGTCTATTACCCCTC-CCCCGTCCGATGGCCGAGTT
15              GATTACACTCTGGAACATCCGTTGGGTCTATTACCCCTC-CCCCGTCCGATGGCCGAGTT
5               GATTACACTCTGGAACATCCGTTGGGTCTATTACCCCTC-CCCCGTCCGATGGCCGAGTT
13              GATTACACTCTGGAACATCCGTTGGGTCTATTACCCCTC-CCCCGTCCGATGGCCGAGTT
                ** ******  ************ ****** ****     ****** *****  * ****

25              CGTGGGGTTAGTAGAAAGG---CGCATTGACG-ATA-TACGCAAGAACA-GAAG--CCTT
26              CGTGGGGTTAGTAGAAAGCGACCGCATTGAGAA----TACGTAGAAGCACGAAGGCTC-T
24              CGTGGGGTTAGTAGAAAGCGA-CGCATTGACGAATATTACGCAAGAACA-GAAGGCTCTT
18              CGT-GGGTTAGTAGAAAGCGA-CGCATTGACG-ATATTACGCAAGAACA-GAAGGCTCTT
22              CGTGGGGTTAGTAGAAAGCGA-CGCATTGACGAATATTACGCAAGAACA-GAAGGCTCTT
14              CGTGGGGTTAGTAGAAAGCGA-CGCATTGACGAATATTACGCAAGAACA-GAAGGCTCTT
16              CGTGGGGTTAGTAGAAAGCGA-CGCATTGACGAATATTACGCAAGAACA-GAAGGCTCTT
20              CGTGGGGTTAGTAGAAAGCGA-CGCATTGACGAATATTACGCAAGAACA-GAAGGCTCTT
27              CGTGGGGTTAGTAGAAAGCGA-CGCATTGACGAATATTACGCAAGAACA-GAAGGCTCTT
2               CGTGGGGTTAGTAGAAAGCGA-CGCATTGACGAATATTACGCAAGAACA-GAAGGCTCTT
7               CGTGGGGTTAGTAGAAAGCGA-CGCATTGACGAATATTACGCAAGAACA-GAAGGCTCTT
23              CGTGGGGTTAGTAGAAAGCGA-CGCATTGACGAAT-TAACGCAAGAACA-GAAGGCTCTT
3               CGTGGGGTTAGTAG-AAGCGA-CGCATTGACGAATATTACGCAAGAACA-GAAGGCTCTT
31              CGTGGGGTTAGTAGAAAGCGA-CGCATTGACGAATATTACGCAAGAACA-GAAGGCTCTT
19              CGTGGGGTTAGTAGAAAGCGA-CGCATTGACGAATATTACGCAAGAACA-GAAGGCTCTT
8               CGTGGGGTTAGTAGAAAGCGA-CGCATTGACGAATATTACGCAAGAACA-GAAGGCTCTT
4               CGT-GGGTTAGTAGAAAGCGA-CGCATTGACGAATATTACGCAAGAACA-GAAGGCTCTT
11              CGTGGGGTTAGTAGAAAGCGA-CGCATTGACGAATATTACGCAAGAACA-GAAGGCTCTT
10              CGT-GGGTTAGTAGAAAGCGA-CGCATTGACGAATATTACGCAAGAACA-GAAGGCTCTT
9               CGTGGGGTTAGTAGAAAGCGA-CGCATTGACGAATATTACGCAAGAACA-GAAGGCTCTT
6               CGTGGGGTTAGTAGAAAGCGA-CGCATTGACGAATATTACGCAAGAACA-GAAGGCTCTT
21              CGTGGGGTTAGTAGAAAGCGA-CGCATTGACGAATATTACGCAAGAACA-GAAGGCTCTT
12              CGTGGGGTTAGTAGAAAGCGA-CGCATTGACGAATATTACGCAAGAACA-GAAGGCTCTT
15              CGTGGGGTTAGTAGAAAGCGA-CGCATTGACGAATATTACGCAAGAACA-GAAGGCTCTT
5               CGTGGGGTTAGTAGAAAGCGA-CGCATTGACGAATATTACGCAAGAACA-GAAGGCTCTT
13              CGTGGGGTTAGTAGAAAGCGA-CGCATTGACGAATATTACGCAAGAACA-GAAGGCTCTT
```

```
              *** ********** ***    ********       *** *  * ** ****   * *
25            TGGCGGGAAGCACAAATT----CAGAAAGAGTTT--GAAACA-TCTCTTTCGAAGAATTT
26            TGGCGGGAAGC-CAAATTGCTACGAAGAGAGTTTTGAAACCAGTCTCTTTCGAAG-ATTT
24            TGGCGGGAAGCACAAATTGCTACGAAGAGAGTTTTGAAACCA-TCTCTTTCGAAGAATTT
18            TGGCGGGAAGCACAAATTGCTACGAAGAGAGTTTTGAAACCA-TCTCTTTCGAAGAATTT
22            TGGCGGGAAGCACAAATTGCTACGAAGAGAGTTTTGAAACCA-TCTCTTTCGAAGAATTT
14            TGGCGGGAAGCACAAATTGCTACGAAGAGAGTTTTGAAACCA-TCTCTTTCGAAGAATTT
16            TGGCGGGAAGCACAAATTGCTACGAAGAGAGTTTTGAAACCA-TCTCTTTCGAAGAATTT
20            T-GCGGGAAGCACAAATTGCTACGAAGAGAGTTTTGAAACCA-TCTCTTTCGAAGAATTT
27            TGGCGGGAAGCACAAATTGCTACGAAGAGAGTTTTGAAACCA-TCTCTTTCGAAGAATTT
2             TGGCGGGAAGCACAAATTGCTACGAAGAGAGTTTTGAAACCA-TCTCTTTCGAAGAATTT
7             TGGCGGGAAGCACAAATTGCTACGAAGAGAGTTTTGAAACCA-TCTCTTTCGAAGAATTT
23            TGGCGGGAAGCACAAATTGCTACGAAGAGAGTTTTGAAACCA-TCTCTTTCGAAGAATTT
3             TGGCGGGAAGCACAAATTGCTACGAAGAGAGTTTTGAAACCA-TCTCTTTCGAAGAA-TT
31            TGGCGGGAAGCACAAATTGCTACGAAGAGAGTTTTGAAACCA-TCTCTTTCGAAGAATTT
19            TGGCGGGAAGCACAAA-TGCTACGAAGAGAGTTTTGAAACCA-TCTCTTTCGAAGAATTT
8             TGGCGGGAAGCACAAATTGCTACGAAGAGAGTTTTGAAACCA-TCTCTTTCGAAGAATTT
4             TGGCGGGAAGCACAAATTGCTACGAAGAGAGTTTTGAAACCA-TCTCTTTCGAAGAATTT
11            TGGCGGGAAGCACAAATTGCTACGAAGAGAGTTTTGAAACCA-TC-CTTTCGAAGAATTT
10            TGGCGGGAAGCACAAATTGCTACGAAGAGAGTTTTGAAACCA-TCTCTTTCGAAGAATTT
9             TGGCGGGAAGCACAAATTGCTACGAAGAGAGTTTTGAAACCA-TCTCTTTCGAAGAATTT
6             TGGCGGGAAGCACAAATTGCTACGAAGAGAGTTTTGAAACCA-TCTCTTTCGAAGAATTT
21            TGGCGGGAAGCACAAATTGCTACGAAGAGAGTTTTGAAACCA-TCTCTTTCGAAGAATTT
12            TGGCGGGAAGCACAAATTGCTACGAAGAGAGTTTTGAAACCA-TCTCTTTCGAAGAATTT
15            TGGCGGGAAGCACAAATTGCTACGAAGAGAGTTTTGAAACCA-TCTCTTTCGAAGAATTT
5             TGGCGGGAAGCACAAATTGCTACGAAGAGAGTTTTGAAACCA-TCTCTTTCGAAGAATTT
13            TGGCGGGAAGCACAAATTGCTACGAAGAGAGTTTTGAAACCA-TCTCTTTCGAAGAATTT
              * ********* **** *    *  * *******   ** ** ** ********* * **

25            AAAGATTTATTCT-TGCAGAATTTAGACGGGTGGATTC-----GACGTACCATGCTGCAC
26            AAAGATTTATTCTGTGCAGAATTTAGCCGGGTGGATTCTCACAGACGGTACATGCTGCAC
24            AAAGATTTATTCTGTGCAGAATTTAG-CGGGTGGATTCTCACAGACGGTACATGCTGCAC
18            AAAGATTTATTCTGTGCAGAATTTAGCC-GGTGGATTCTCACAGACGGTACATGCTGCAC
22            AAGA--TTATTCTGTGCAGAATTTAG-CGGGTGGATTCTCACAGACGGTACATGCTGCAC
14            AAAGATTTATTCTGTGCAGAATTTAGCCGGGTGGATTCTCACAGACGGTACATGCTGCAC
16            AAAGATTTATTCTGTGCAGAATTTAGCCGGGGTGATTCTCACAGACGGTACATGCTGCAC
20            AAAGATTTATTCTG-GCAGAATTTAGCCGGGTGGATTCTCACAGACGGTACATGCTGCAC
27            AAAGATTTATTCTGTGCAGAATTTAGCCGGGTGGATTCTCACAGACGGTACATGCTGCAC
2             AAAGATTTATTCTGTGCAGAATTTAGCCGGGTGGATTCTCACAGACGGTACATGCTGCAC
7             AAAGATTTATTCTGTGCAGAATTTAGCCGGGTGGATTCTCACAGACGGTACATGCTGCAC
23            AAAGATTT-TTCTGTGCAGAATTTAGCCGGGTGGATTCTCA-AGACGGTACATGCTGCAC
3             AAAGATTTATTCTGTGCAGAATTTAGCCGGGTGGATTCTCAC-GACGGTACATGCTGCAC
31            AAAGATTTATTCTGTGCAGAA-TTAGCCGGGTGGATTCTCACAGACGGTACATGCTGCAC
19            AAAGATTTATTCTGTGCAGAATTTAGCCGGGTGGATTCTCACAGACGGTACATGCTGCAC
8             AAAGATTTATTCTGTGCAG-ATTTAGCCGGGTGGATTCTCACAGACGGTACATGCTGCAC
4             AAAGA-TTATTCTGTGCAGAATTTAGCCGGGTGGATTCTCAC-GACGGTACATGCTGCAC
11            AAAGATTTATTCTGTGCAGAATTTAGCCGGGTGGATTCTCACAGACGGTACATGCTGCAC
10            AAAGATTTATTCTGTGCAGAATTTAGCCGGGTGGATTCTCACAGACGGTACATGCTGCAC
9             AAAGATTTATTCTGTGCAGAATTTAGCCGGGTGGATTCTCACAGACGGTACATGCTGCAC
6             AAAGATTTATTCTGTGCAGAATTTAGCCGGGTGGATTCTCACAGACGGTACATGCTGCAC
21            AAAGATTTATTCTGTGCAGAATTTAGCCGGGTGGATTCTCACAGACGGTACATGCTGCAC
12            AAAGATTTATTCTGTGCAGAATTTAGCCGGGTGGATTCTCACAGACGGTACATGCTGCAC
15            AAAGATTTATTCTGTGCAGAATTTAGCCGGGTGGATTCTCACAGACGGTACATGCTGCAC
5             AAAGATTTATTCTGTGCAGAATTTAGCCGGGTGGATTCTCACAGACGGTACATGCTGCAC
13            AAAGATTTATTCTGTGCAGAATTTAGCC-GGTGGATTCTCACAGACGGTACATGCTGCAC
              **    ** ****  **** * **** * **  *****     ****   **********

25            GA-ATTTACCCAAATCCGCGTCCCGGCCG-CTTTCTGAAAATGATC-GGGGATCGTACCC
26            GATATTTACCCAAATCCGCGTCCCGGCCGCCTTTCTGAAAATGATCGGGGAATCGTACCC
24            GATATTTACCCAAATCCGCGTCCCGGCCGCCTTTCTGAAAATGATCGGGGAATCGTACCC
18            GATATTTACCCAAATCCGCGTCCCGGCCGCCTTTC--GAAATGATCGGGGAATCGTACCC
22            GATATT-ACCCAAATCCGCGTCCCGG-CGCCTTTCTGAAAATGATCGGGGAATCGTACCC
14            GATATTTACCCAAATCCGCGTCCCGGCCGCCTTTCTGAAAATGATCGGGGAATCGTACCC
16            GATATT-ACCCAAATCCGCGTCCCGGCCGCCTTTCTGAAAATGATCGGGGAATCGTACCC
20            GATATTTACCCAAATCCGCGTCCCGGCCGCCTTTCTGAAAATGATCGGGGAATCGTACCC
27            GATATT-ACCCAAATCCGCGTCCCGGCCGCCTTTCTGAAAATGATCGGGGAATCGTACCC
2             GATATTTACCCAAATCCGCGTCCCGGCCGCCTTTCTGAAAATGAT--GGGAATCGTACCC
7             GATATTTACCCAAATCCGCGTCCCGGCCGCCTTTCTGAAAATGATCGGGGAATCGTACCC
23            GATATTTACCCAAATCCGCGT-CCGGCCGCCTTTCTGAAAATGATCGGGGAATCGTACCC
3             GATATTTACCCAAATCCGCGTCCCGGCCGCCTTTCTGAAAATGATCGGGGAATCGTACCC
31            GATATT--ACCAAATCCGCGTCCCGGCCGCCTTTCTGAAAATGATCGGGGAATCGTACCC
19            GATATTTA-CCAAATCCGCGTCCCGGCCGCCTTTCTGAAAATGATC-GGGAATCGTA-CC
8             GATATTTACCCAAATCCGCGTCCCGGCCGCCTTTCTGAAAATGATCGGGGAATCGTACCC
4             GATATTTACCCAAATCCGCGTCCCGGCCGCCTTTCTGAAAATGATC-GGGAATCGTACCC
11            GATATTTACCCAAATCCGCGTCCCGGCCGCCTTTCTGAAAATGATCGGGGAATCGTACCC
10            GATATTTACCCAAATCCGCGTCCCGGCCGCCTTTCTGAAAATGATCGGGGAATCGTACCC
9             GATATTTACCCAAATCCGCGTCCCGGCCGCCTTTCTGAAAATGATC-GGGAATCGTACCC
6             GATATTTACCCAAATCCGCGTCCCGGCCGCCTTTCTGAAAATGATCGGGGAATCGTACCC
21            GATATTTACCCAAATCCGCGTCCCGGCCGCCTTTCTGAAAATGATCGGGGAATCGTACCC
12            GATATTTACCCAAATCCGCGTCCCGGCCGCCTTTCTGAAAATGATCGGGGAATCGTACCC
15            GATATTTACCCAAATCCGCGTCCCGGCCGCCTTTCTGAAAATGATCGGGGAATCGTACCC
5             GATATTTACCCAAATCCGCGTCCCGGCCGCCTTTCTGAAAATGATCGGGGAATCGTACCC
13            GATATTTACCCAAATCCGCGTCCCGGCCGCCTTTCTGAAAATGATCGGGGAATCGTACCC
              ** ***   ************ **** ** *****   *******  *** ****** **

25            AAGAGCACA-GGAACCTGTCG-GCGTCGTGGTCATGCGTCGTGTTCGAG-ACAGCAAGCA
26            AAGAGCACAGGGAACTCGTCG-GCGTCGTGGTCATTCGTCGTGTTCGAGCACGGCAAGCA
24            AAGAGCACAGGGAACTCGTCG-GCGTCGTGGTCATTCGTCGTGTTCGAGCACGGCAAGCA
18            AAGAGCACAGGGAACTCGTCG-GCGTCGTGGTCATTCGTCGTGTTCGAGCACGGCAAGCA
22            AAGAGCACAGGGAACTCGTCG-GCGTCGTGGTCATTCGTCGTGTTCGAGCACGGCAAGCA
14            AAGAGCACAGGGAACTCGTCG-GCGTCGTGGTCATTCGTCGTGTTCGAGCACGGCAAGCA
16            AAGAGCACAGGGAACTCGTCG-GCGTCGTGGTCATTCGTCGTGTTCGAGCACGGCAAGCA
20            AAGAGCACAGGGAACTCGTCG-GCGTCGTGGTCATTCGTCGTGTTCGAGCACGGCAAGCA
27            AAGAGCACAGGGAACTCGTCG-GCGTCGTGGTCATTCGTCGTGTTCGAGCACGGCAAGCA
2             AAGAGCACAGGGAACTCGTCG-GCGTCGTGGTCATTCGTCGTGTTCGAGCACGGCAAGCA
7             AAGAGCACAGGGAACTCGTCG-GCGTCGTGGTCATTCGTCGTGTTCGAGCACGGCAAGCA
23            AAGAGCACAGGGAACTCGTCG-GCGTCGTGGTCATTCGTCGTGTTCGAGCACGGCAAGCA
3             AAGAGCACAGGGAA-TCGTCGCGCGTCGTGGTCATTCGTCGTGTTCGAGCACGGCAAGCA
31            AAGAGCACAGGGAACTCGTCG-GCGTCGTGGTCATTCGTCGTGTTCGAGCACGGCAAGCA
19            AAGAGCACAGGGAACTCGTCG-GCGTCGTGGTCATTCGTCGTGTTCGAGC-CGGCAAGCA
8             AAGAGCACAGGGAACTCGTCG-GCGTCGTGGTCATTCGTCGTGTTCGAGCACGGCAAGCA
4             AAGAGCACAGGGAACTCGTCG-GCGTCGTGGTCATTCGTCGTGTTCGAGCACGGCAAGCA
11            AAGAGCACAGGGAACTCGTCG-GCGTCGTGGTCATTCGTCGTGTTCGAGCACGGCAAGCA
10            AAGAGCACAGGGAACTCGTCG-GCGTCGTGGTCATTCGTCGTGTTCGAGCACGGCAAGCA
9             AAGAGCACAGGGAACTCGTCG-GCGTCGTGGTCATTCGTCGTGTTCGAGCACGGCAAGCA
6             AAGAGCACAGGGAACTCGTCG-GCGTCGTGGTCATTCGTCGTGTTCGAGCACGGCAAGCA
21            AAGAGCACAGGGAACTCGTCG-GCGTCGTGGTCATTCGTCGTGTTCGAGCACGGCAAGCA
12            AAGAGCACAGGGAACTCGTCG-GCGTCGTGGTCATTCGTCGTGTTCGAGCACGGCAAGCA
15            -AGAGCACAGGGAACTCGTCG-GCGTCGTGGTCATTCGTCGTGTTCGAGCACGGCAAGCA
5             AAGAGCACAGGGAACTCGTCG-GCGTCGTGGTCATTCGTCGTGTTCGAGCACGGCAAGCA
13            AAGAGCACAGGGAACTCGTCG-GCGTCGTGGTCATTCGTCGTGTTCGAGCACGGCAAGCA
               ******** ****   **** ************* *************  * *******

25            CGTCCATTGTCATCCACCTG-GCAGATACGAGAG-TCATGGTGC-TGTTCGCG-TCACC-
26            CGTCCA-TGTCATCCACCTGTGCAGATACGAGAGATCATGGTGCTTGTTCGCGCTCACCA
24            CGTCCA-TGTCATCCACCTGTGCAGATACGAGAGATCATGGTGCTTGTTCGCGCTCACCA
18            CGTCCA-TGTCATCCACCTGTGCAGATACGAGAGATCATGGTGCTTGTCTGCGCTCACCA
22            CGTCCA-TGTCAT-CACCTGTGCAGATACGAGAGATCATGGTGCTTGTTCGCGCTCACC-
14            CGTCCA-TGTCATCCACCTGTGCAGATACGAGAGATCATGGTGCTTGTTCGCGCTCACCA
16            CGTCCA-TGTCATCCACCTGTGCAGATACGAGAGATCATGGTGCTTGTTCGCGCTCACCA
20            CGTCCA-TGTCATCCACCTGTGCAGATACGAGAGATCATGGTGCTTGTTCGCGCTCACCA
27            CGTCCA-TGTCATCCACCTGTGCAGATACGAGAGATCATGGTGCTTGTTCGCGCTCACCA
2             CGTCCA-TGTCATCCACCTGTGCAGATACGAGAGATCATGGTGCTTGTTCGCGCTCACC-
7             CGTCCA-TGTCATCCACCTGTGCAGATACGAGAGATCATGGTGCTTGTTCGCGCTCACCA
23            CGTCCA-TGTCATCCACCTGTGCAGATACGAGAGATCATGGTGCTTGTTCGCGCTCACCA
3             CGTCCA-TGTCATCCACCTGTGCAGATACGAGAGATCATGGTGCTTGTTCGCGCTCACCA
31            CGTCCA-TGTCATCCACCTGTGCAGATACGAGAGATCATGGTGCTTGTTCGCGCTCACCA
19            CGTCCA-TGTCATCCACCTGTGCAGATACGAGA--TCATGGTGC-TGTTCGCGCTC-CCA
8             CGTCCA-TGTCATCCACCTGTGCAGATACGAGAGATCATGGTGCTTGTTCGCGCTCACC-
4             CGTCCA-TGTCATCCACCTGTGCAGATACGAGAGATCATGGTGCTTGTTCGCGCTCACCA
11            CGTCCA-TGTCATCCACCTGTGCAGATACGAGAGATCATGGTGCTTGTTCGCGCTCACCA
10            CGTCCA-TGTCATCCACCTGTGCAGATACGAGAGATCATGGTGCTTGTTCGCGCTCACCA
9             CGTCCA-TGTCATCCACCTGTGCAGATACGAGAGATCATGGTGCTTGTTCGCGCTCACCA
6             CGTCCA-TGTCATCCACCTGTGCAGATACGAGAGATCATGGTGCTTGTTCGCGCTCACCA
21            CGTCCA-TGTCATCCACCTGTGCAGATACGAGAGATCATGGTGCTTGTTCGCGCTCACC-
12            CGTCCA-TGTCATCCACCTGTGCAGATACGAGAGATCATGGTGCTTGTTCGCGCTCACCA
15            CGTCCA-TGTCATCCACCTGTGCAGATACGAGAGATCATGGTGCTTGTTCGCGCTCACCA
5             CGTCCA-TGTCATCCACCTGTGCAGATACGAGAGATCATGGTGCTTGTTCGCGCTCACCA
13            CGTCCA-TGTCATCCACCTGTGCAGATACGAGAGATCATGGTGCTTGTTCGCGCTCACCA
              ****** ****** ****** ************  ********* ***   *** ** **

25            AAAAAGCAGAA-GAAGGTTCGGACGAAGAATTGGAAGATATACTCTTCAAGCAAGTAGGC
26            AAAAAGCAGAAGGAAGGTTCGGACGAAGAATTGGAAGATATACTCTTCGAAGCAGTAGGC
24            AAAAAGCAGAAGGAAGGTTCGGACGAAGAATTGGAAGATATACTCTTCGAAGCAGTAGGC
18            AAAAAGCAGAAGGAAGGTTCGGACG-AGAATTGGAA-ATATACTCTTCGAAGCAGTAGGC
22            AAAAAGCAGAAGGAAGGTTCGGACGAAGAATTGGAAGATATACTCTTCGAAGCAGTAGGC
14            AAAAAGCAGAAGGAAGGTTCGGACGAAGAATTGGAAGATATACTCTTCGAAGCAGTAGGC
16            AAAAAGCAGAAGGAAGGTTCGGACGAAGAATTGGAAGATATACTCTTCGAAGCAGTAGGC
20            AAAAAGCAGAAGGAAGGTCGGGACGAAGAATTGGAAGATATACTC-TCGAAGCAGTAGGC
27            AAAAAGCAGAAGGAAGGTTCGGACGAAGAATTGGAAGATATACTCTTCGAAGCAGTAGGC
2             AAAAAGCAGAAGGAAGGTTCGGACGAAGAATTGGAAGATATACTCTTCGAAGCAGTAGGC
7             AAAAAGCAGAAGGAAGGTTCGGACGAAGAATTGGAAGATATACTCTTCGAAGCAGTAGGC
23            AAAAAGCAGAAGGAAGGTTCGGACGAAGAATTGGAAGATATACTCTTCGAAGCAGTAGGC
3             AAAAAGCAGAAGGAAGGTTCGGACGAAGAATTGGAAGATATACTCTTCGAAGCAGTAGGC
31            AAAAAGCAGAAGGAAGGTTCGGACGAAGAATTGGAAGATATACTCTTCGAAGCAGTAGGC
19            AAAAAGCAGAAGGAAGGTTCGGACGAAGAATTGGAAGATATACTCTTCGAAGCAGTAGGC
8             AAAAAGCAGAAGGAAGGTTCGGACGAAGAATTGGAAGA-ATACTCTTCGAAGCAGTAGGC
4             AAAAAGCAGAAGGAAGGTTCGGACGAAGAATTGGAAGATATACTCTTCGAAGCAGTAGGC
11            AAAAAGCAGAAGGAAGGTTCGGACGAAGAATTGGAAGATATACTCTTCGAAGCAGTAGGC
10            AAAAAGCAGAAGGAAGGTTCGGACGAAGAATTGGAAGATATACTCTTCGAAGCAGTAGGC
9             AAAAAGCAGAAGGAAGGTTCGGACGAAGAATTGGAAGATATACTCTTCGAAGCAGTAGGC
6             AAAAAGCAGAA-GAAGGTTCGGACGAAGAATTGGAAGATATACTCTTCGAAGCAGTAGGC
21            AAAAAGCAGAAGGAAGGTTCGGACGAAGAATTGGAAGATATACTCTTCGAAGCAGTAGGC
12            AAAAAGCAGAAGGAAGGTTCGGACGAAGAATTGGAAGATATACTCTTCGAAGCAGTAGGC
15            AAAAAGCAGAAGGAAGGTTCGGACGAAGAATTGGAAGATATACTCTTCGAAGCAGTAGGC
5             AAAAAGCAGAAGGAAGGTTCGGACGAAGAATTGGAAGATATACTCTTCGAAGCAGTAGGC
13            AAAAAGCAGAAGGAAGGTTCGGACGAAGAATTGGAAGATATACTCTTCGAAGCAGTAGGC
              *********** ******  ***** ********** * ****** ** *   *******

25            TCTCAGAAG--CACATCCGCA--ATCGCAAGTTATAGACAAAAAGGGAAAAGAA-GCTGC
26            TCTCAGAAAGCCACATCCGCAATATCGCAAGTTATAGACAAAAAGGGAAAAGAAGGCTGC
24            TCTCAGAAAGCCACATCCGCAATATCGCAAGTTATAGACAAAAAGGGAAAAGAAGGCTGC
18            TCTCAGAAAGCCACATCCGCAATATCGCAAGTTATAGAC-AAAAGGGAAAAGAAGGCTGC
22            TCTCAGAAAGCCACATCCGCAATATCGCAAGTTATAGACAAAAAGGGAAAAGAAGGCTGC
14            TCTCAGAAAGCCACATCCGCAATATCGCAAGTTATAGACAAAAAGGGAAAAGAAGGCTGC
16            TCTCAGAAAGCCACATCCGCAATATCGCAAGTTATAGAC-AAAAGGGAAAAGAAGGCTGC
20            TCTCAGAAAGCCACATCCGCAATATCGCAAGTTATAGACAAAAAGGGAAAAGAAGGCTGC
27            TCTCAGAAAGCCACATCCGCAATATCGCAAGTTATAGACAAAAAGGGAAAAGAAGGCTGC
2             TCTCAGAAAGCCACATCCGCAATATCGCAAGTTATAGAC-AAAAGGGAAAAGAAGGCTGC
7             TCTCAGAAAGCCACATCCGCAATATCGCAAGTTATAGAC--AAAAGGGAAAGAAGGCTGC
23            TCTCAGAAAGCCACATCCGCAATATCGCAAGTTATAGACAAAAAGGGAAAAGAAGGCTGC
3             TCTCAGAAAGCCACATCCGCAATATCGCAAGTTATAGAC-AAAAAGGGAAAGAAGGCTGC
31            TCTCAGAAAGCCACATCCGCAATATCGCAAGTTATAGACAAAAAGGGAAAAGAAGGCTGC
19            TCTCAGAAAGCCACATCCGCAATATCGCAAGTTATAGACAAAAAGGGAAAAGAAGGCTGC
8             TCTCAGAAAGCCACATCCGCA-TATCGCAAGTTATAGAC-AAAAGGGAAAAGAAGGCTGC
4             TCTCAGAAAGCCACATCCGCAATATCGCAAGTTATAGAC-AAAAGGGAAAAGAAGGCTGC
11            TCTCAGAAAGCCACATCCGCAATATCGCAAGTTATAGACAAAAAGGGAAAAGAAGGCTGC
10            TCTCAGAAAGCCACATCCGCAATATCGCAAGTTATAGACAAAAAGGGAAAAGAAGGCTGC
9             TCTCAGAAAGCCACATCCGCAATATCGCAAGTTATAGAC-AAAAGGGAAAAGAAGGCTGC
```

```
6               TCTCAGAAAGCCACATCCGCAATATCGCAAGTTATAGACAAAAAGGGAAAAGAAGGCTGC
21              TCTCAGAA-GCCACATCCGCAATATCGCAAGTTATAGACAAAAAGGGAAAAGAAGGCTGC
12              TCTCAGAAAGCCACATCCGCAATATCGCAAGTTATAGACAAAAAGGGAAAAGAAGGCTGC
15              TCTCAGAAAGCCACATCCGCAATATCGCAAGTTATAGAC-AAAAAGGAAAAGAAGGCTGC
5               TCTCAGAAAGCCACATCCGCAATATCGCAAGTTATAGACAAAAAGGGAAAAG-AGGCTGC
13              TCTCAGAAAGCCACATCCGCAATATCGCAAGTTATAGACAAAAAGGGAAAAGAAGGCTGC
                ********   **********  ****************  *** ** **** * *****

25              TTCAAGC-TTTCTCGCCAGGAGAGAAGG--AGATACATGATCAAGCTCGATTTCTACCGG
26              TTCAAGCTTTTCTCGCCAGGAGAGAAAGGCGAATACATGATCAAGCTCGATTTCTACCGG
24              TTCAAGCTTTTCTCGCCAGGAGAGAAAGGCGAATACATGATCAAGCTCGATTTCTACCGG
18              TTCAAGCTTTTCTCGCCCAGAGAGAAAGGCGAATACATGATCAAGCTCGATTTCTACCGG
22              TTCAAGC--TTTTCGCCAGGAGAGAAAGGCGAATACATGATCAAGCTCGATTTCTACCGG
14              TTCAAGCTTTTCTCGCCGGGAGAGAAAGGCGAATACATGATCAAGCTCGATTTCTACCGG
16              TTCAAGCTTTTCTCGCCAGGAGAGAAAGGCGAATACATGATCAAGCTCGATTTCTACCGG
20              TTCAAGCTTTTCTCGCCAGGAGAGAAAGGCGAATACATGATCAAGCTCGATTTCTACCGG
27              TTCAAGCTTTTCTCGCCAGGAGAGAAAGGCGAATACATGATCAAGCTCGATTTCTACCGG
2               TTCAAGC-TTTCTCGCCAGGAGAGAAAGGCGAATACATGATCAAGCTCGATTTCTACCGG
7               TTCAAGCTTTTCTCGCCAGGAGAGAAAGGCGAATACATGATCAAGCTCGATTTCTACCGG
23              TTCAAGCTTTTCTCGCCAGGAGAGAAAGGCGAATACATGATCAAGCTCGATTTCTACCGG
3               TTCAAGCTTTTCTCGCCAGG-GAGAAAGGCGAATACATGATCAAGCTCGATTTCTACCGG
31              TTCAAGCTTTTCTCGCCAGGAGAGAAAGGCGAATACATGATCAAGCTCGATTTCTACCGG
19              TTCAAGC-TTTCTCGCCAGGAGAGAAAGGCGAATACATGATCAAGCTCGATTTCTACCGG
8               TTCAAGCTTTTCTCGCCAGGAGAGAAAGGCGAATACATGATCAAGCTCGATTTCTACCGG
4               TTCAAGCTTTTCTCGCCAGGAGAG-AAGGCGAATACATGATCAAGCTCGATTTCTACCGG
11              TTCAAGCTTTTCTCGCCAGGAGAGAAAGGCGAATACATGATCAAGCTCGATTTCTACCGG
10              TTCAAGCTTTTCTCGCCAGGAGAGAAAGGCGAATACATGATCAAGCTCGATTTCTACCGG
9               TTCAAGCTTTTCTCGCCAGGAGAGAAAGGCGAATACATGATCAAGCTCGATTTCTACCGG
6               TTCAAGCTTTTCTCGCCAGGAGAG-AAGGCGAATACATGATCAAGCTCGATTTCTACCGG
21              TTCAAGCTTTTCTCGCCAGGAGAGAAAGGCGAATACATGATCAAGCTCGATTTCTACCGG
12              TTCAAGCTTTTCTCGCCAGGAGAGAAAGGCGAATACATGATCAAGCTCGATTTCTACCGG
15              TTCAAGCTTTTCTCGCCAGGAGAGAAAGGCGAATACATGATCAAGCTCGATTTCTACCGG
5               TTCAAGCTTTTCTCGCCAGGAGAGAAAGGCGAATACATGATCAAGCTCGATTTCTACCGG
13              TTCAAGCTTTTCTCGCCAGGAGAGAAAGGCGAATACATGATCAAGCTCGATTTCTACCGG
                *******  ** *****  * *** * *    ****************************

25              ACCCG-CAGCTGAGAGATGTAGCG-AGGACAATTGGTGGA-GGAAGCGGACTACCGGGCC
26              ACCCGTCAGCTGAGGAGAGTAGCGAAGGACAATTGGTGGAGGGAAGCGGACTACCGGGCC
24              ACCCGTCAGCTGAGGAGAGTAGCGAAGGACAATTGGTGGAGGGAAGCGGACTACCGGGCC
18              ACCCGTCAGCTGAGGAGAGTAGCGAAGGACAA-TGGTGGAGGGAAGCGGACTACC-GGCC
22              ACCCGTCAGCTGAGGAGAGTAGCGAAGGACAA-TGGTGGAGGGAAGCGGACTACCGGGCC
14              ACCCGTCAGCTGAGGAGAGTAGCGAAGGACAATTGGTGGAGGGAAGCGGACTACCGGGCC
16              ACCCGTCAGCTGAGGAGAGTAGCGAAGGACAATTGGTGGAGGGAAGCGGACTACCGGGCC
20              ACCCGTCAGCTGAGGAGAGTAGCGAAGGACAATTGGTGGAGGGAAGCGGACTACCGGGCC
27              ACCCGTCAGCTGAGGAGAGTAGCGAAGGACAATTGGTGGAGGGAAGCGGACTACCGGGCC
2               ACCCGTCAGCTGAGGAGAGTAGCGAAGGACAATTGGTGGAGGGAAGCGGACTACCGGGCC
7               ACCCGTCAGCTGAGGAGAGTAGCGAAGGACAATTGGTGGAGGGAAGCGGACTACCGGGCC
23              ACCCGTCAGCTGAGGAGAGTAGCGAAGGACAATTGGTGGAGGGAAGCGGACTACCGGGCC
3               ACCCGTCAGCTGAGGAGAGTAGCGAAGGACAATTGGTGGAGGGAAGCGGACTACCGGGCC
31              ACCCGTCAGCTGAGGAGAGTAGCGAAGGACAATTGGTGGAGGGAAGCGGACTACCGGGCC
19              ACCCGTCAGCTGAGGAGAGTAGCGAAGGACAATTGGTGGAGGGAAGCGGACTACCGGGCC
8               ACCCGTCAGCTGAGGAGAGTAGCGAAGGACAATTGGTGGAGGGAAGCGGACTACCGGGCC
4               ACCCGTCAGCTGAGGAGAGTAGCGAAGGACAATTGGTGGAGGGAAGCGGACTACCGGGCC
11              ACCCGTCAGCTGAGGAGAGTAGCGAAGGACAATTGGTGGAGGGAAGCGGACTA-CGGGCC
10              ACCCGTCAGCTGAGGAGAGTAGCGAAGGACAATTGGTGGAGGGAAGCGGACTACCGGGCC
9               ACCCGTCAGCTGAGGAGAGTAGCGAAGGACAATTGGT-GAGGGAAGCGGACTACCGGGCC
6               ACCCGTCAGCTGAGGAGAGTAGCGAAGGACAATTGGTGGAGGGAAGCGGACTACCGGGCC
21              ACCCGTCAGCTGAGGAGAGTAGCGAAGGACAATTGGTGGAGGGAAGCGGACTACCGGGCC
12              ACCCGTCAGCTGAGGAGAGTAGCGAAGGACAATT-GTGGAGGGAAGCGGACTACCGGGCC
15              ACCCGTCAGCTGAGGAGAGTAGCGAAGGACAATTGGTGGAGGGAAGCGGACTACCGGGCC
5               ACCCGTCAGCTGAGGAGAGTAGCGAAGGACAATTGGTGGAGGGAAGCGGACTACCGGGCC
13              ACCCGTCAGCTGAGGAGAGTAGCGAAGGACAATTGGTGGAGGGAAGCGGACTACCGGGCC
                ***** ********    ****** ******* * ** ** ************ * ****

25              ACGTTCGTGCTTTT---AACGGATCCTGC-GCAACTCAGATAAAGAA-ATCCTCAAGGAG
26              ACGTTCGTGCTTTCTTACACGGATCCTGCAGCAACTCAGATAAAGAAGATCCTCAAGGAG
24              ACGTTCGTGCTTTCTTACACGGATCCTGCAGCAACTCAGATAAAGAAGATCCTCAAGGAG
18              ACGTTCGTGCTTTCTTACACGGATCCTGCAGCAACTCAGATAAAGAAGATCCTCAAGGAG
22              ACGTTCGTGTTTTCTTACACGGACCCTGCAGCAACTCAGATAAAGAAGATCCTCAAGGAG
14              ACGTTCGTGCTTTCTTACACGGATCCTGCAGCAACTCAGATAAAGAAGATCCTCAAGGAG
16              ACGTTCTTGCTTTCTTACACGGATCCTGCAGCAACTCAGATAA-GAAGATCCTCAAGGAG
20              ACGTTCGTGCTTTCTTACACGGATCCTGCAGCAACTCAGATAAAGAAGATCCTCAAGGAG
27              ACGTTCTTGCTTTCTTACACGGATCCTGCAGCAACTCAGATAAAGAAGATCCTCAAGGAG
2               ACGTTCGTGCTTTCTTACACGGA-CCTGCAGCAACTCAGATAAAGAAGATCCTCAAGGAG
7               ACGTTCGTGCTTTCTTACACGGATCCTGCAGCAACTCAGATAAAGAAGATCCTCAAGGAG
23              ACGTTCGTGCTTT--TACACGGATCCTGCAGCAACTCAGATAAAGAAGATCCTCAAGGAG
3               ACGTTCGTGCTTTCTTACACGGATCCTGCAGCAACTCAGATAAAG-AGATCCTCAAGGAG
31              ACGTTCGTGCTTTCTTACACGGATCCTGCAGCAACTCAGATAAAGAAGATCCTCAAGGAG
19              ACGTTCGTGCTTTCTTACACGGATCCTGCAGCAACTCAGATAAAGAAGATCCTCAAGGAG
8               ACGTTCGTGCTTTCTTACACGGATCCTGCAGCAACTCAGATAAAGAAGATCCTCAAGGAG
4               ACGTTCGTGCTTTCTTACACGGATCCTGCAGCAACTCAGATAAAGAAGATCCTCAAGGAG
11              ACGTTCGTGCTTTCTTACACGGATCCTGCAGCAACTCAGATAAAGAAGATCCTCAAGGAG
10              ACGTTCGTGCTTTCTTACACGGATCCTGCAGCAACTCAGATAAA--AGATCCTCAAGGAG
9               ACGTTCGTGCTTTCTTACACGGATCCTGCAGCAACTCAGATAAAGAAGATCCTCAAGGAG
6               ACGTTCGTGCTTTCTTACACGGATCCTGCAGCAACTCAGATAAAGAAGATCCTCAAGGAG
21              ACGTTCGTGCTTTCTTACACGGATCCTGCAGCAACTCAGATAAAGAAGATCCTCAAGGAG
12              ACGTTCGTGCTTTCTTACACGGATCCTGCAGCAACTCAGATAAAGAAGATCCTCAAGGAG
15              ACGTTCGTGCTTTCTTACACGGATCCTGCAGCAACTCAGATAAAGAAGATCCTCAAGGAG
5               ACGTTCGTGCTTTCTTACACGGATCCTGCAGCAACTCAGATAAAGAAGATCCTCAAGGAG
13              ACGTTCGTGCTTTCTTACACGGATCCTGCAGCAACTCAGATAAAGAAGATCCTCAAGGAG
                ****** ** ***     ***** ***** *************   * ************

25              CAGGTCAAGAAGATAGCAGCGATTCCGGATCGGAAGA-TCGAATTTTTACAAAAACAAAA
26              CAGGTCAAGAAGATAGCAGCGATTCCGGATCGGAAGA-TCGAA-TTTTACAAAAACAAAA
24              CAGGTCAAGAAGATAGCAGCGATTCCGGATCGGAAGA-TCGAATTTTTACAAAAACAAAA
18              CAGGTCAAGAAGATAGCAGCGATTCCGGATCGGAAGA-TCGAA-TTTTACAAAAAAAAAA
22              CAGGTCAAGAAGATAGCAGCGATT-CGGATCGGAAGA-TCGAATTTTTAC-AAAACAAAA
14              CAGGTCAAGAAGATAGCAGCGATTCCGGATCGGAAGA-TCGAATTTTTACAAAAACAAAA
16              CAGGTCAAGAAGATAGCAGCGATTCCGGATCGGAAGA-TCGAATTTTTAC-AAAACAAAA
20              CAGGTCAAGAAGATAGCAGCGATTCCGGATCGGAAGA-TCGAA-TTTTACAAAAACAAAA
27              CAGGTCAAGAAGATAGCAGCGATTCCGGATCGGAAGA-TCGAATTTTTACAAAAACAAAA
2               CAGGTCAAGAAGATAGCAGCGATTCCGGATCGGAAGA-TCGAATTTTTAC-AAAACAAAA
7               CAGGTCAAGAAGATAGCAGCGATTCCGGATCGGAAGA-TCGAATTTTTAC-AAAACAAAA
23              CAGGTCAAGAAGATAGCAGCGATTCCGGATCGGAAGA-TCGAATTTTTACAAAAACAAAA
3               CAGGTCAAGAAGATAGCAGCGATTCCGGATCGGAAGA-TCGAATTTTTACAAAAACAAAA
31              CAGGTCAAGAAGATAGCAGCGATTCCGGATCGGAAGA-TCGAATTTTTACAAAAACAAAA
19              CAGGTCAAGAAGATAGCAGCGATTCCGGATCGGAAGAGTCGAATTTTTACAAAAACAAAA
8               CAGGTCAAGAAGATAGCAGCGATTCCGGATCGGAAGA-TCGAATTTTTACAAAAACAAAA
4               CAGGTCAAGAAGATAGCAGCGATTCCGGATCGGAAGA-TCGAATTTTTACAAAAACAAAA
11              CAGGTCAAGAAGATAGCAGCGATTCCGGATCGGAAGA-TCG-ATTTTTACAAAAACAAAA
10              CAGGTCAAG-AGATAGCAGCGATTCCGGATCGGAAGA-TCGAATTTTTACAAAAACAAAA
9               CAGGTCAAGAAGATAGCAGCGATTCCGGATCGGAAGA-TCGAATTTTTACAAAAACAAAA
6               CAGGTCAAGAAGATAGCAGCGATTCCGGATCGGAAGA-TCGAATTTTTACAAAAACAAAA
21              CAGGTCAAGAAGATAGCAGCGATTCCGGATCGGAAGA-TCGAATTTTTACAAAAACAAAA
12              CAGGTCAAGAAGATAGCAGCGATTCCGGATCGGAAGA-TCGAATTTTTACAAAAACAAAA
15              CAGGTCAAGAAGATAGCAGCGATTCCGGATCGGAAGA-TCGAATTTTTACAAAAACAAAA
5               CAGGTCAAGAAGATAGCAGCGATTCCGGATCGGAAGA-TCGAATTTTTACAAAAACAAAA
13              CAGGTCAAGAAGATAGCAGCGATTCCGGATCGGAAGA-TCGAATTTTTACAAAAACAAAA
                ********* ************** ************ *** * ****** **** ****

25              GG---AAAAAAATAATGA---TTTTTATA---CATG-GTAAT-ACCCCTTTTCTCG----
26              GGGA-AAAAAAATAATTGAA--TTTTATC-ATCATGCGTAATTACCCCTTTTCT-GTATC
24              AGGGAAAAAAAAATAATGA---TTTTTATC-AT-ATGCGTAATTA-CCCTTTTCTCGTATC
18              GGG---AAAAAAAAATTGAA-TTTTTATC-ATCATGCGTAATTACCCCTTTTCTCGTATC
22              AGGGAAAAAAAAATAAT-GAA--TTTTATCAATCATGCGTAATTACCCCTTTTCTCGTATC
14              GGGA-AAAAAAATAATTGAA-TTTTTATC-ATCATGCGTAATTACCCCTTTTCTCGTATC
16              AGGG-AAAAAAATAATTGAA-TTTTTATC-ATTATGCGTAATTACCCCTTTTCTCGTATC
20              AGGGAAAAAAAAATAATTGAA-TTTTTATC-ATCATGCGTAATTACCCCTTTTCTCGTATC
27              AGGGAAAAAAAAATAATTGAA-TTTTTATC-ATTATGCGTAATTACCCCTTTTCTCGTATC
2               GGG--AAAAAAATAATTGAA-TTTTTATC-ATCATGCGTAATA--CCCTTTTCTCGTATC
7               GGG--AAAAAAATAATTGAA-TTTTTATC-ATCATGCGTAATTACCCCTTTTCTCGTATC
23              AGGGAAAAAAAAATAATTGAA-TTTTTATC-ATCATGCGTAATTACCCCTTTTCTCGTATC
3               AGGGAAAAAAAAATAATTGAA-TTTTTATC-ATCATGCGTAATTACCCCTTTTCTCGTATC
31              AGGGAAAAAAAAATAATTGAA-TTTTTATC-ATCATGCGTAATTACCCCTTTTCTCGTATC
19              AGGGAAAAAAAAT-ATTGAA-TTTTTATC-ATCATGCGTAATTACCCCTTTTCTCGTATC
8               AGGG-AAAAAAATAATTGAA-TTTTTATC-ATCATGCGTAATTACCCCTTTTCTCGTATC
4               GGGA-AAAAAAATAATTGAATTTTTTATC-ATCATGCGTAATTA-CCCTTTTCTCGTATC
11              AGGGAAAAAAAAATAATTGAA-TTTTTATC-ATCATGCGTAATTACCCCTTTTCTCGTATC
10              AGGGAAAAAAAAT-ATTGAA-TTTTTATC-ATCATGCGTAATTACCCCTTTTCTCGTATC
9               AGGGAAAAAAAAT-ATTGAA-TTTTTATC-ATCATGCGTAATTACCCCTTTTCTCGTATC
6               AGGGAAAAAAAAATAATTGAA-TTTTTATC-ATCATGCGTAATTACCCCTTTTCTCGTATC
21              AGGGAAAAAAAAT-ATTGAA--TTTTATC-ATCATGCGTAATTACCCCTTTTCTCGTATC
12              AGGG-AAAAAAATAATTGA--TTTTTATC-ATCATGCGTAATTACCCCTTTTCTCGTATC
15              AGGGAAAAAAAAATAATTGAA-TTTTTATC-ATCATGCGTAATTACCCCTTTTCTCGTATC
5               AGGGAAAAAAAAATAATTGAA-TTTTTATC-ATCATGCGTAATTACCCCTTTTCTCGTATC
13              AGGG-AAAAAAATAATTGAA-TTTTTATC-ATCATGCGTAATTACCCCTTTTCTCGTATC
                 *    ******  **      ******     *** *****   ********* *

25              ATTCAATCCGAGTTGTATCTTTACGAAAAATC-TTATATTACA-TACTCCCG-ATCTATG
26              TTTCAATCCGAGTTGTATCTTTTCGATAAATCGTTATATTACATTACTCCCGAATCTATG
24              TTTCAATCCGAGTTG-ATTCTTTCGATAAATCGTTATATTACATTACTCCCGAATCTATG
18              TTTCAATCCGAGTTGTATCTTTTCGATAAATCGTTATATTACA-TACTCCCGAATCTATG
22              TTTCAATCCGAGTTGTATCTTTTCGATAAATCGTTATATTACATTACTCCCGAATCTATG
14              TTTCAATCCGAGTTGTATCTTTTCGATAAATCGTTATATTACATTACTCCCGAATCTATG
16              TTTCAATCCGAGTTGTATCTTTTCGATAAATCGTTATATTACATTACTCCCGAATCTATG
20              TTTCAATCCGAGTTGTATCTTTTCGATAAATCGTTATATTACATTACTCCCGAATCTATG
27              TTTCAATCCGAGTTGTATCTTTTCGATAAATCGTTATATTACATTACTCCCGAATCTATG
2               TTTCAATCCGAGTTGTATCTTTTCGATAAATCGTTATATTACATTACTCCCGAATCTATG
7               TTTCAATCCGAGTTGTATCTTTTCGATAAATCGTTATATTACATTACTCCCGAATCTATG
23              TTTCAATCCGAGTTGTATCTTTTCGATAAATCGTTATATTACATTACTCCCGAATCTATG
3               TTTCAATCCGAGTTGTATCTTTTCGATAAATCGTTATATTACATTACTCCCGAATCTATG
31              TTTCAATCCGAGTTGTATCTTTTCGATAAATCGTTATATTACATTACTCCCGAATCTATG
19              TTTCAATCCGAGTTGTATCTTT-CGATAAATCGTTATATTACATTACTCCCGAATCTATG
8               TTTCAATCCGAGTTGTATCTTTTCGATAAATCGTTATATTACATTACT-CCGAATCTATG
4               TTTCAATCCGAGTTGTATCTTTTCGATAAATCGTTATATTACATTACTCCCGAATCTATG
11              TTTCAATCCGAGTTGTATCTTTTCGATAAATCGTTATATTACATTACTCCCGAATCTATG
10              TTTCAATCCGAGTTGTATCTTTTCGATAAATCGTTATATTACATTACTCCCGAATCTATG
9               TTTCAATCCGAGTTGTATCTTTTCGATAAATCGTTATATTACATTACTCCCGAATCTATG
6               TTTCAATCCGAGTTGTATCTTTTCGAT-AATCGTTATATTACATTACTCCCGAATCTATG
21              TTTCAATCCGAGTTGTATCTTTTCGATAAATCGTTATATTACATTACTCCCGAATCTATG
12              TTTCAATCCGAGTTGTATCTTTTCGATAAATCGTTATATTACATTACTCCCGAATCTATG
15              TTTCAATCCGAGTTGTATCTTTTCGATAAATCGTTATATTACATTACTCCCGAATCTATG
5               TTTCAATCCGAGTTGTATCTTTTCGATAAATCGTTATATTACATTACTCCCGAATCTATG
13              TTTCAATCCGAGTTGTATCTTTTCGATAAATCGTTATATTACATTACTCCCGAATCTATG
                 ************** **  ** ***  **** ********** **** *** *******

25              GCCTGTCAGTTAGCGATAAT-CA-ACACA-TCACA-CATTAATAAGATGAGT--AAATCA
26              GCCTGTCAGTTAGCGATAATGCA-ACACACTCACACCA-TAATAAGATGAGTGTAAATCA
24              G-CTGTCAGTTAGCGATAATGCA-ATACACTCAC-CCATTAATAAGATGAGTGTAACATC
18              GCCTGTCAGTTAGCGATAATGCA-ACACACTCACACCATTAATAAGATGAGTGTAAATCA
22              GCCTGTCAGTTAGCGATAATGCA-ACACACTCACACCATTAATAAGATGAGTG-AAATCA
14              GCCTGTCAGTTAGCGATAATGCA-ACACACTCACACCATTAATAAGATGAGTGTAAATCA
16              GCCTGTCAGTTAGCGATAATGCA-ACACACTCACACCATTAATAAGATGAGTGTAAATCA
20              GCCTGTCAGTTAGCGATAATGCA-ACACACTCACACCA-TAATAAGATGAGTGTAAATCA
27              GCCTGTCAGTTAGCGATAATGCA-ACACACTCACACCATTAATAAGATGAGTGTAAATCA
2               GCCTGTCAGTTAGCGATAATGCA-ACACACTCACACCATTAATAAGATGAGTGTAAATCA
7               GCCTGTCAGTTAGCGATAATGCA-ACACACTCACACCATTAAT-AGATGAGTGTAAATCA
23              GCCTGTCAGTTAGCGATAATGCA-ACACACTCACACCATTAATAAGATGAGTGTAAATCA
3               GCCTGTCAGTTAGCGATAATGCA-ACACACTCACACCATTAATAAGATGAGTGTAAATCA
31              GCCTGTCAGTTAGCGATAATGCA-ACACACTCACACCATTAATAAGATGAGTGTAAATCA
```

```
19          GCCTGTCAGTTAGCGATAATGCA-ACACACTCACACCATTAATAAGATGAGTGTAAATC-
8           GCCTGTCAGTTAGCGATAATGCACACACACTCACACCATTAATAAGATGAGTGTAAATCA
4           GCCTGTCAGTTAGCGATAATGCA-ACACACTCACACCATTAATAAGATGAGTGTAAATCA
11          GCCTGTCAGTTAGCGATAATGCA-ACACACTCACACCATTAATAAGATGAGTGTAAATCA
10          GCCTGTCAGTTAGCGATAATGCA-ACACACTCACACCATTAATAAGATGAGTGTAAATCA
9           GCCTGTCAGTTAGCGATAATGCA-ACACACTCACACCATTAATAAGATGAGTGTAAATCA
6           GCCTGTCAGTTAGCGATAATGCA-ACACACTCACACCATTAATAAGATGAGTGTAAATCA
21          GCCTGTCAGTTAGCGATAATGCA-ACACACTCACACCATTAATAAGATGAGTGTAAATCA
12          GCCTGTCAGTTAGCGATAATGCA-ACACACTCACACCATT-ATAAGATGAGTGTAAATCA
15          GCCTGTCAGTTAGCGATAATGCA---ACACTCACACCATTAATAAGATGAGTGTAAATCA
5           GCCTGTCAGTTAGCGATAATGCA-ACACACTCACACCATTAATAAGATGAGTGTAAATCA
13          GCCTGTCAGTTAGCGATAATGCA-ACACACTCACACCATTAATAAGATGAGTGTAAATCA
            * ****************** **   *** ****  ** * ** ********  **

25          ATTA-TTCAGTTACA-TTAAACATT-CGATTTCTAAA-TATTTTACAATTGTAACGTCCG
26          ATTATTTCAGTATCAATTAAACATTCCGA-TTCTAAATTATTTTACAATTGTAACGCTCC
24          ATTATTTCAGTATCA--TAAACATT-CGATTTCTAAATTA-TTTACAATTGTAACGCTC-
18          ATTATTTCAGTATCAATTAAACATTCCGA-TTCTAAATTA-TTTACAATTGTAACGCTCC
22          ATTATTTCAGTATCAATTAAACATTCCGATTTCTAAATTA-TTTACAATTGTAACGCTCC
14          ATTATTTCAGTATCAATTAAACATTCCGATTTCTAAATTATTTTACAATTGTAACGCTCC
16          ATTATTTCAGTATCAATTAAACATTCCGATTTCTAAATTATTTTACAATTGTAACGCTCC
20          ATTA-TTCAGTATCAATTAAACATTCCGATTTCTAAATTATTTTACAATTGTAACGCTCC
27          ATTATTTCAGTATCAATTAAACATTCCGATTTCTAAATTATTTTACAATTGTAACGCTCC
2           ATTATTTCAGTATCAATTAAACATTCCGATTTCTAAATTATTTTACAATTGTAACGCTC-
7           ATTATTTCAGTATCA-TTAAACATTCCGATTTCTAAATTATTTTACAATTGTAACGCTCC
23          ATTATTTCAGTATCAATTAAACATTCCGATTTCTAAATTATTTTACAATTGTAACGCTCC
3           ATTA-TTCAGTATCAATTAAACATTCCGATTTCAAAATTATTTTACAATTGTAACGCTCC
31          ATTATTTCAGTATCAATTAAACATTCCGATTTCTAAATTATTTTACAATTGTAACGCTCC
19          ATTA-TTCAGTATCAATTAAACATTCCGATTTCTAAATTA-TTTACAATTGTAACGCTCC
8           ATTATTTCAGTATCAATTAAACATTCCGATTTCTAAATTATTTTACAATTGTAACGCTCC
4           ATTATTTCAGTATCAATTAAACATTCCGATTTCTAAATTATTTTACAATTGTAACGCTCC
11          ATTATTTCAGTATCAATTAAACATTCCGATTTCTAAATTATTTTACAATTGTAACGCTCC
10          ATTATTTCAGTATCAATTAAACATTCCGATTTCTAAATTA-TTTACAATTGTAACGCTCC
9           ATTATTTCAGTATCAATTAAACATTCCGATTTCTAAATTA-TTTACAATTGTAACGCTCC
6           ATTATTTCAGTATCAATTAAACATTCCGATTTCTAAATTATTTTACAATTGTAACGCTCC
21          ATTATTTCAGTATCAATTAAACATTCCGATTTCTAAATTA-TTTACAATTGTAACGCTCC
12          ATTATTTCAGTATCAATTAAACATTCCGATTTCTAAATTATTTTACAATTGTAACGCTCC
15          ATTATTTCAGTATCAATTAAACATTCCGATTTCTAAATTATTTTACAATTGTAACGCTCC
5           ATTATTTCAGTATCAATTAAACATTCCGATTTCTAAATTATTTTACAATTGTAACGCTCC
13          ATTATTTCAGTATCAATTAAACATTCCGATTTCTAAATTATTTTACAATTGTAACGCTCC
            **** ******  **  ******** *** *** *** ** ***************  *

25          TATAGAATATTTTT-
26          GGATGAATATTTTTT
24          -GATGAATAGTTTT-
18          GGATGAATATTTTT-
22          GGATGAATATTTTTT
14          GGATGAATATTTTT-
16          GGATGAATATTTTT-
20          GGATGAATATTTTTT
27          GGATGAATATTTTTT
2           GGATGAATATTTTTT
7           GGATGAATATTTTTT
23          GGATGAATATTTTTT
3           GGATGAATATTTTTT
31          GGATGAATATTTTTT
19          GGATGAATATTTTT-
8           GGATGAATATTTTTT
4           GGATGAATATTTTTT
11          GGATGAATATTTTTT
10          GGATGAATATTTTTT
9           GGATGAATATTTTTT
6           GGATGAATATTTTTT
21          GGATGAATATTTTTT
12          GGATGAATATTTTT-
15          GGATGAATATTTTT-
5           GGATGAATATTTTTT
13          GGATGAATATTTTTT
                ***** ****
```
