# Supplementary material for: A Novel Parvovirus Associated with the Whitefly Bemisia tabaci
Source: Pathogens. 2025 Jul 19;14(7):714. doi: 10.3390/pathogens14070714 (PMC12297876; doi:10.3390/pathogens14070714)

Fig. S1. InterPro protein domain analysis for Bemisia tabaci ambidenosvirus (BtaDV) NS and VP proteins

NS1 of 719 amino acids

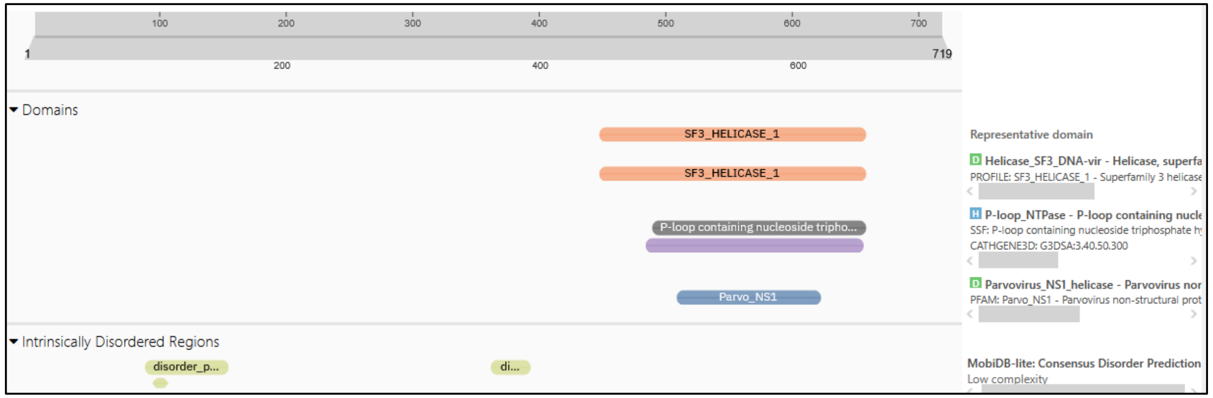

NS2 of 332 amino acids

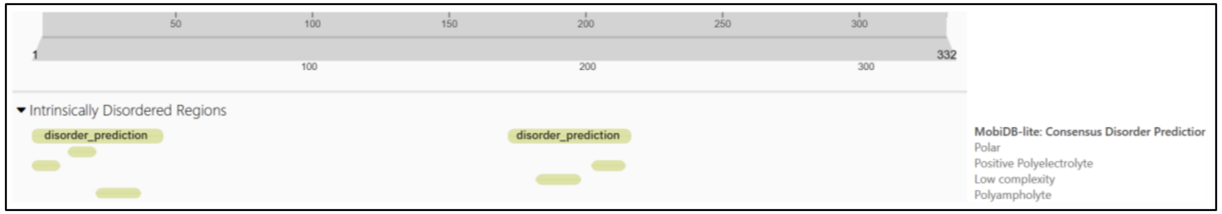

VP of 749 amino acids

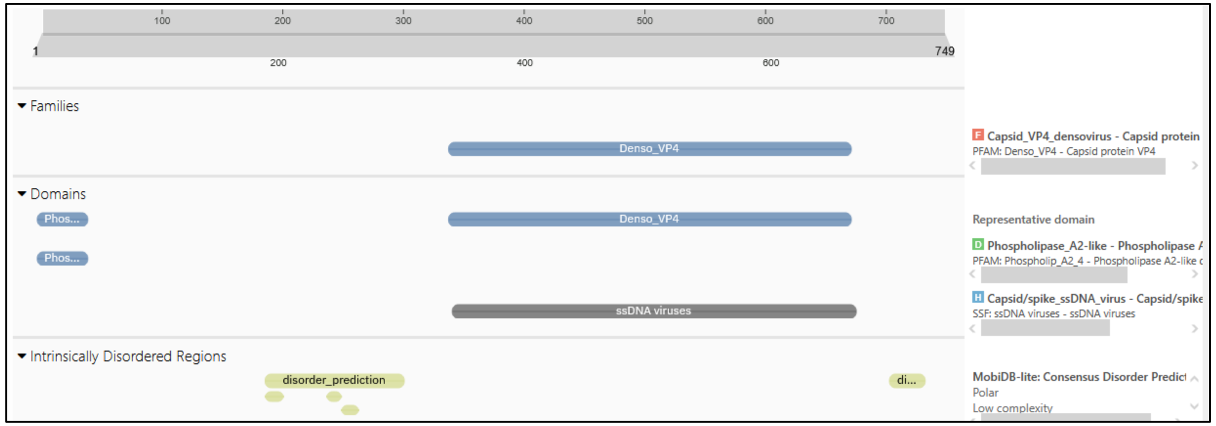

Fig. S2A. BEAST-generated phylogenetic tree of the NS1 protein SF3 helicase/Parvo NS1 domain of Bemisia tabaci ambidenosvirus (BtaDV) and all current members of the family *Parvoviridae*. Part of the tree corresponding to the subfamily Densovirinae is shown, with the currently established genera indicated by black brackets with respective names and the position of BtaDV and its tentative new genus named *Betaambidensovirus* indicated in red.

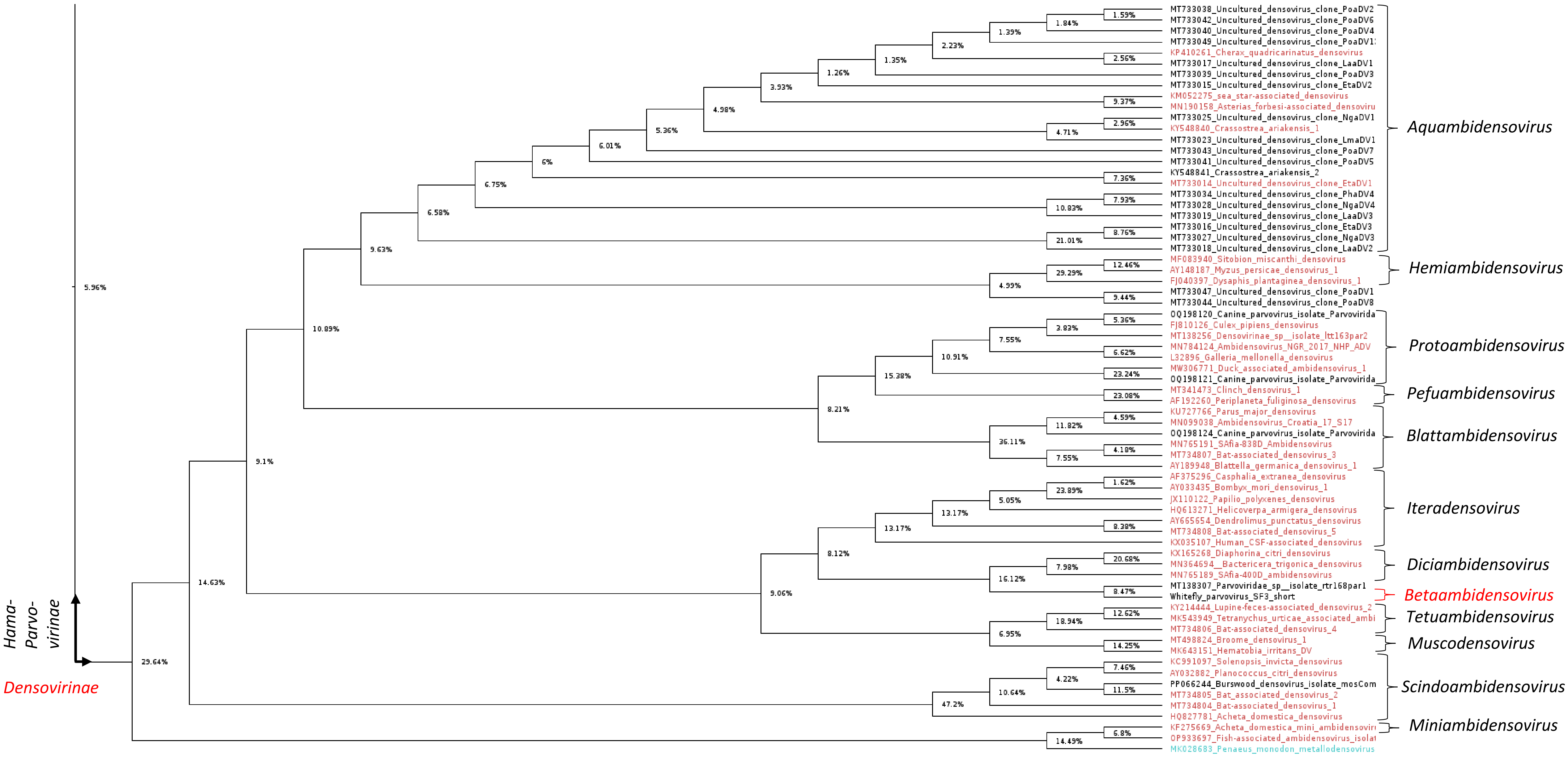

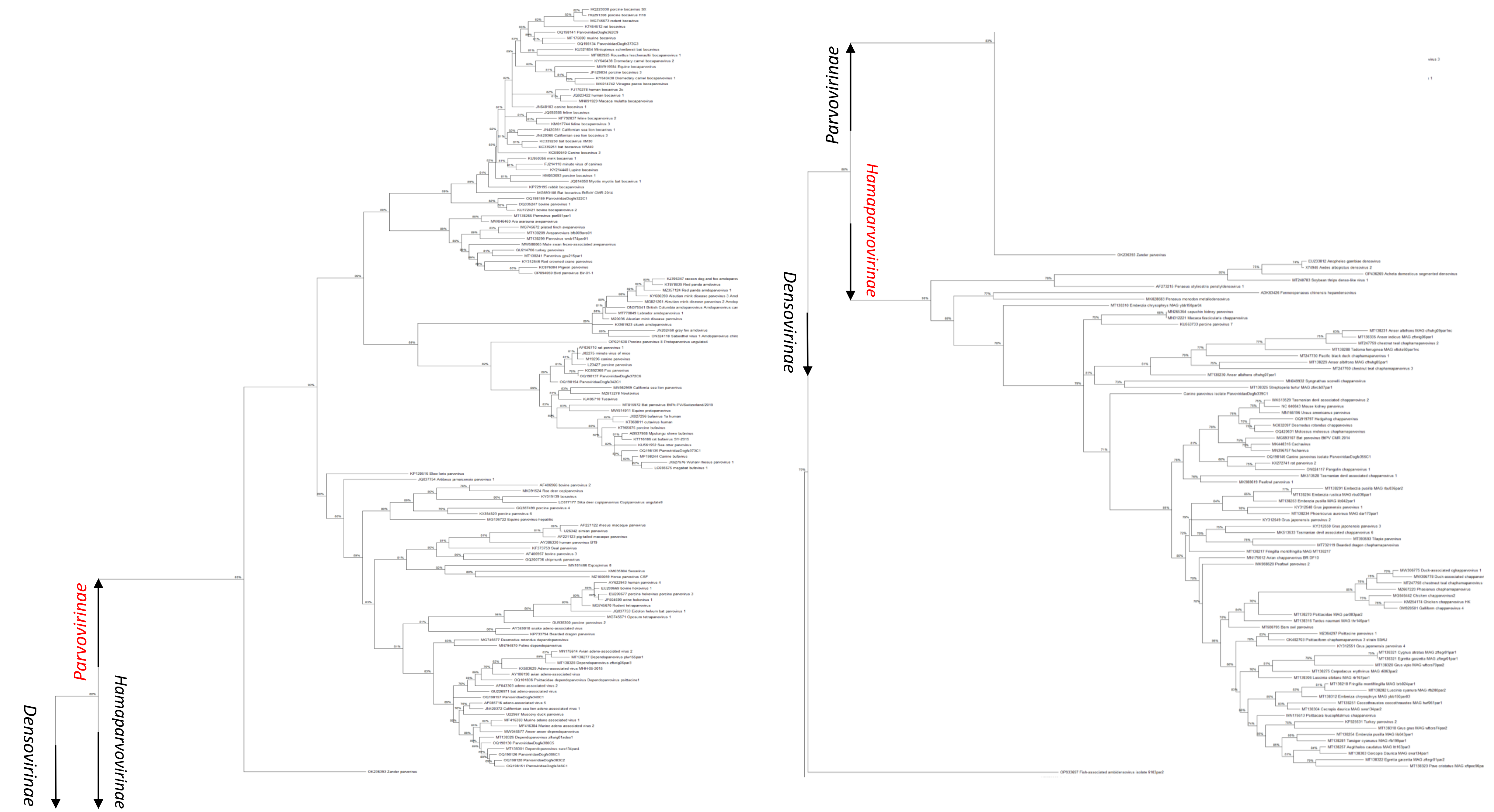

Fig. S2B. MEGA-generated Maximum Likelihood phylogenetic tree of the NS1 protein SF3 helicase/Parvo NS1 domain of Bemisia tabaci ambidenosvirus (BtaDV) and all current members of the family *Parvoviridae*. Parts of the tree corresponding to the subfamilies *Parvovirinae* (left) and *Hamaparvovirinae* (right) are shown, while the part corresponding to the subfamily *Densovirinae* shown below.

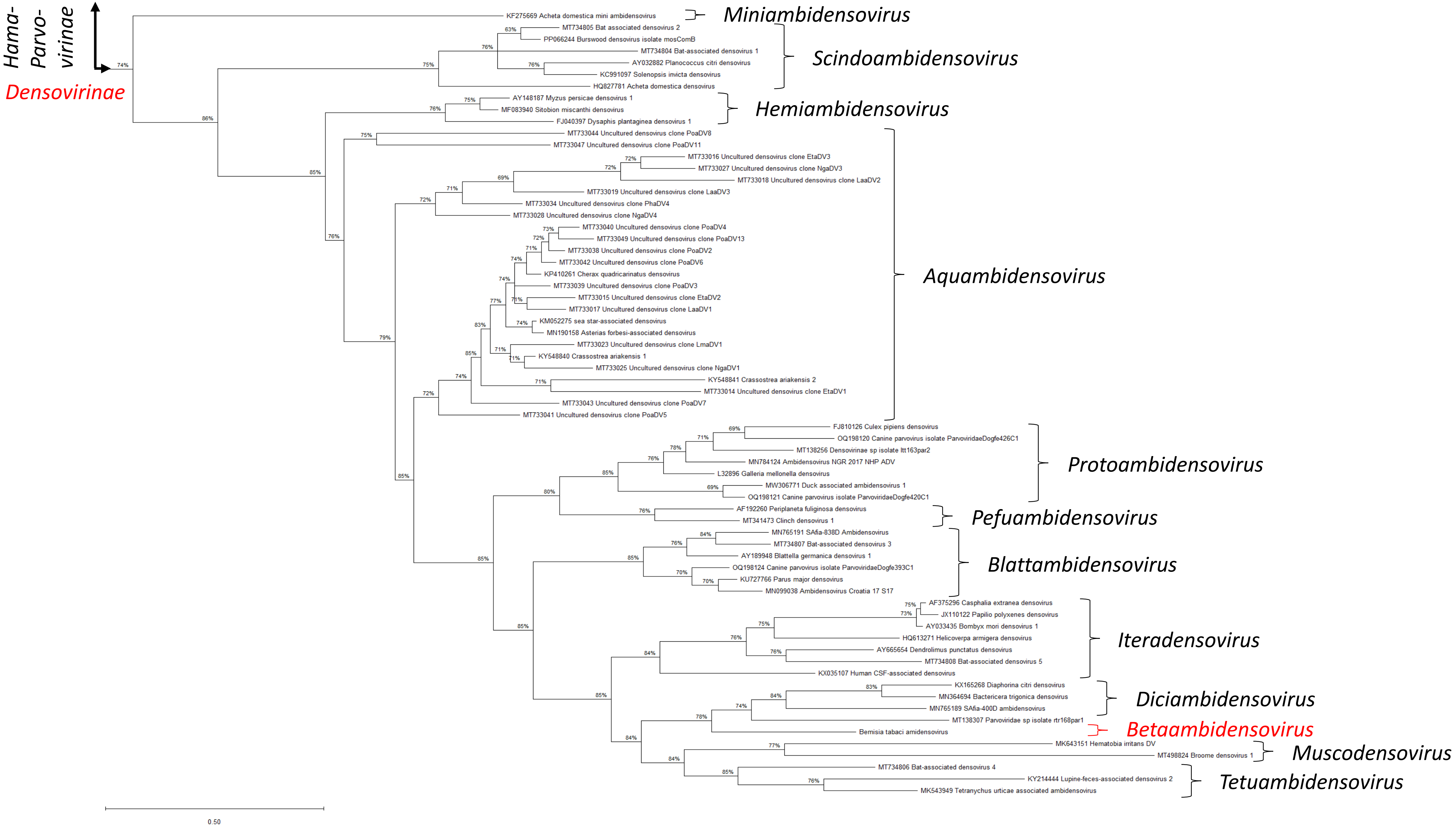

Hama-
Parvo-
virinae
Densovirinae
KF275669 Acheta domestica mini ambidensovirus
Miniambidensovirus
MT734805 Bat associated densovirus 2
PP066244 Burswood densovirus isolate mosComB
MT734804 Bat-associated densovirus 1
AY032882 Planococcus citri densovirus
KC991097 Solenopsis invicta densovirus
HQ827781 Acheta domestica densovirus
Scindoambidensovirus
AY148187 Myzus persicae densovirus 1
MF083940 Sitobion miscanthi densovirus
FJ040397 Dysaphis plantaginea densovirus 1
Hemiambidensovirus
MT733044 Uncultured densovirus clone PoaDV8
MT733047 Uncultured densovirus clone PoaDV11
MT733016 Uncultured densovirus clone EtaDV3
MT733027 Uncultured densovirus clone NgaDV3
MT733018 Uncultured densovirus clone LaaDV2
MT733019 Uncultured densovirus clone LaaDV3
MT733034 Uncultured densovirus clone PhaDV4
MT733028 Uncultured densovirus clone NgaDV4
MT733040 Uncultured densovirus clone PoaDV4
MT733049 Uncultured densovirus clone PoaDV13
MT733038 Uncultured densovirus clone PoaDV2
MT733042 Uncultured densovirus clone PoaDV6
KP410261 Cherax quadricarinatus densovirus
MT733039 Uncultured densovirus clone PoaDV3
MT733015 Uncultured densovirus clone EtaDV2
MT733017 Uncultured densovirus clone LaaDV1
KM052275 sea star-associated densovirus
MN190158 Asterias forbesi-associated densovirus
MT733023 Uncultured densovirus clone LmaDV1
KY548840 Crassostrea ariakensis 1
MT733025 Uncultured densovirus clone NgaDV1
KY548841 Crassostrea ariakensis 2
MT733014 Uncultured densovirus clone EtaDV1
MT733043 Uncultured densovirus clone PoaDV7
MT733041 Uncultured densovirus clone PoaDV5
Aquambidensovirus
FJ810126 Culex pipiens densovirus
OQ198120 Canine parvovirus isolate ParvoviridaeDogfe426C1
MT138256 Densovirinae sp isolate ltt163par2
MN784124 Ambidensovirus NGR 2017 NHP ADV
L32896 Galleria mellonella densovirus
MW306771 Duck associated ambidensovirus 1
OQ198121 Canine parvovirus isolate ParvoviridaeDogfe420C1
Protoambidensovirus
AF192260 Periplaneta fuliginosa densovirus
MT341473 Clinch densovirus 1
Pefuambidensovirus
MN765191 SAfia-838D Ambidensovirus
MT734807 Bat-associated densovirus 3
AY189948 Blattella germanica densovirus 1
OQ198124 Canine parvovirus isolate ParvoviridaeDogfe393C1
KU727766 Parus major densovirus
MN099038 Ambidensovirus Croatia 17 S17
Blattambidensovirus
AF375296 Casphalia extranea densovirus
JX110122 Papilio polyxenes densovirus
AY033435 Bombyx mori densovirus 1
HQ613271 Helicoverpa armigera densovirus
AY665654 Dendrolimus punctatus densovirus
MT734808 Bat-associated densovirus 5
KX035107 Human CSF-associated densovirus
Iteradensovirus
KX165268 Diaphorina citri densovirus
MN364694 Bactericera trigonica densovirus
MN765189 SAfia-400D ambidensovirus
Diciambidensovirus
MT138307 Parvoviridae sp isolate rtr168par1
Bemisia tabaci amidensovirus
Betaambidensovirus
MK643151 Hematobia irritans DV
MT498824 Broome densovirus 1
Muscodensovirus
MT734806 Bat-associated densovirus 4
KY214444 Lupine-feces-associated densovirus 2
MK543949 Tetranychus urticae associated ambidensovirus
Tetuambidensovirus
0.50

Fig. S2C. Sequence Demarcation Tool (SDT) analysis of the NS1 protein SF3 helicase/Parvo NS1 domain of Bemisia tabaci ambidenosvirus (BtaDV) and all current members of the subfamily *Densovirinae.*

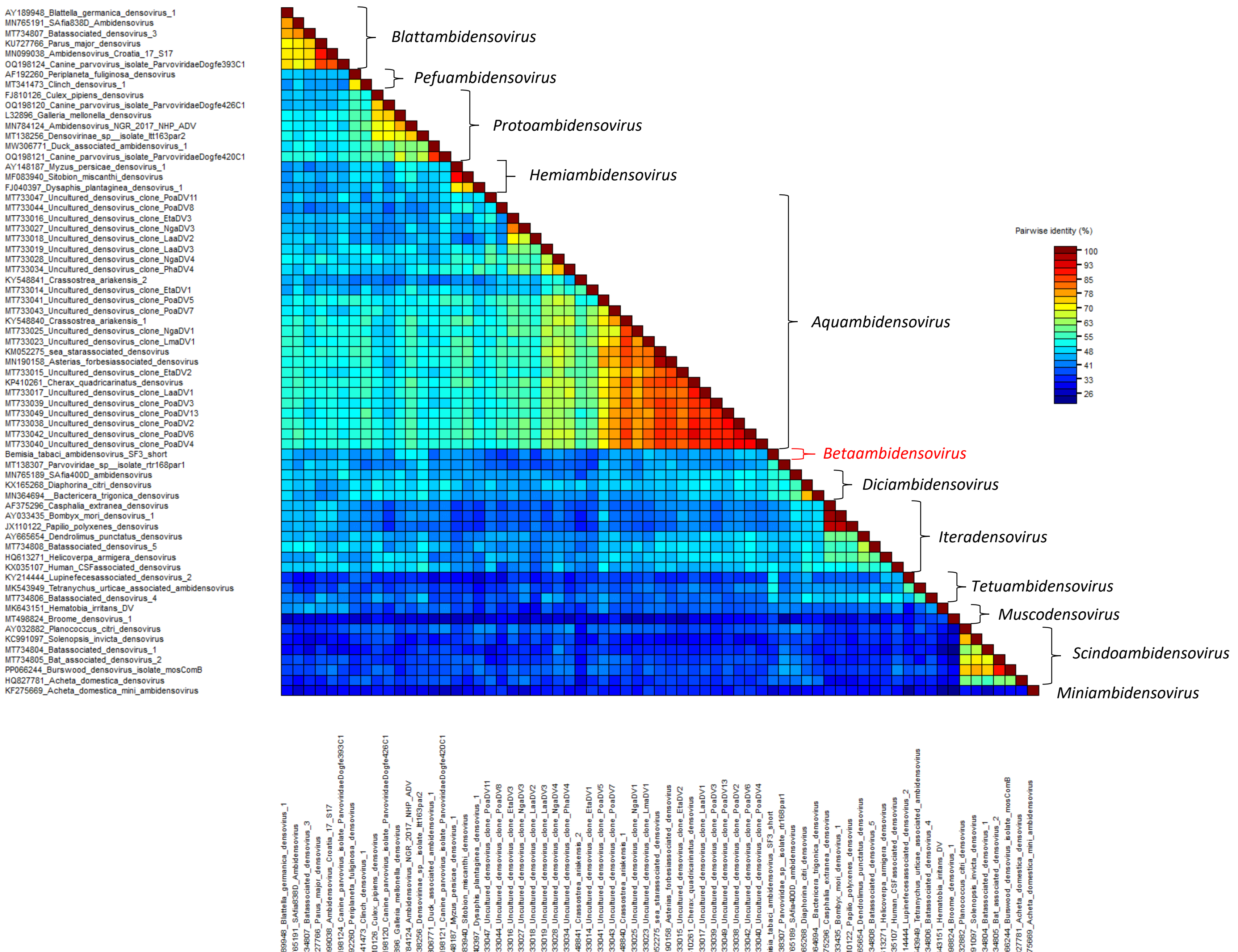

Fig. S3A. IGV visualization of the Illumina 150 nt reads from the ALYU-390 library mapped to the complete genome sequence of Bemisia tabaci ambidenosvirus (BtaDV). Snapshot of the IGV map is shown. Individual reads of white and black colors mapped to the hairpin-forming sequences of the 5'- and 3'- inverted terminal repeats indicate that they have respectively lower and much lower mapping quality than the reads colored in grey. Within individual reads, positions of SNPs are indicated with colored bars, while those of insertion with purple bars and deletion with black lines.

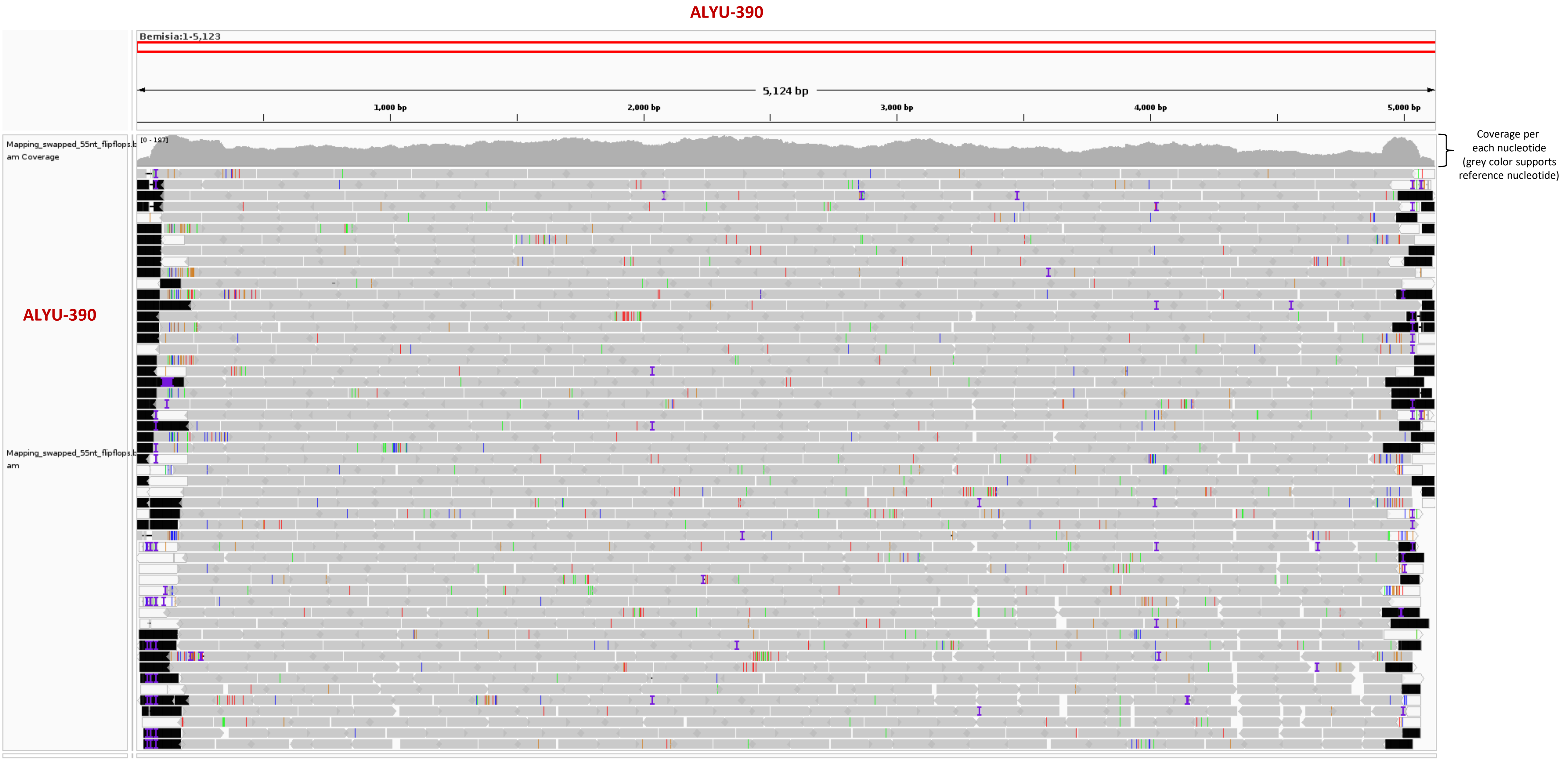

Fig. S3B. IGV visualization of the Illumina 150 nt reads from other ALYU libraries mapped to the complete genome sequence of Bemisia tabaci ambidenosvirus (BtaDV).

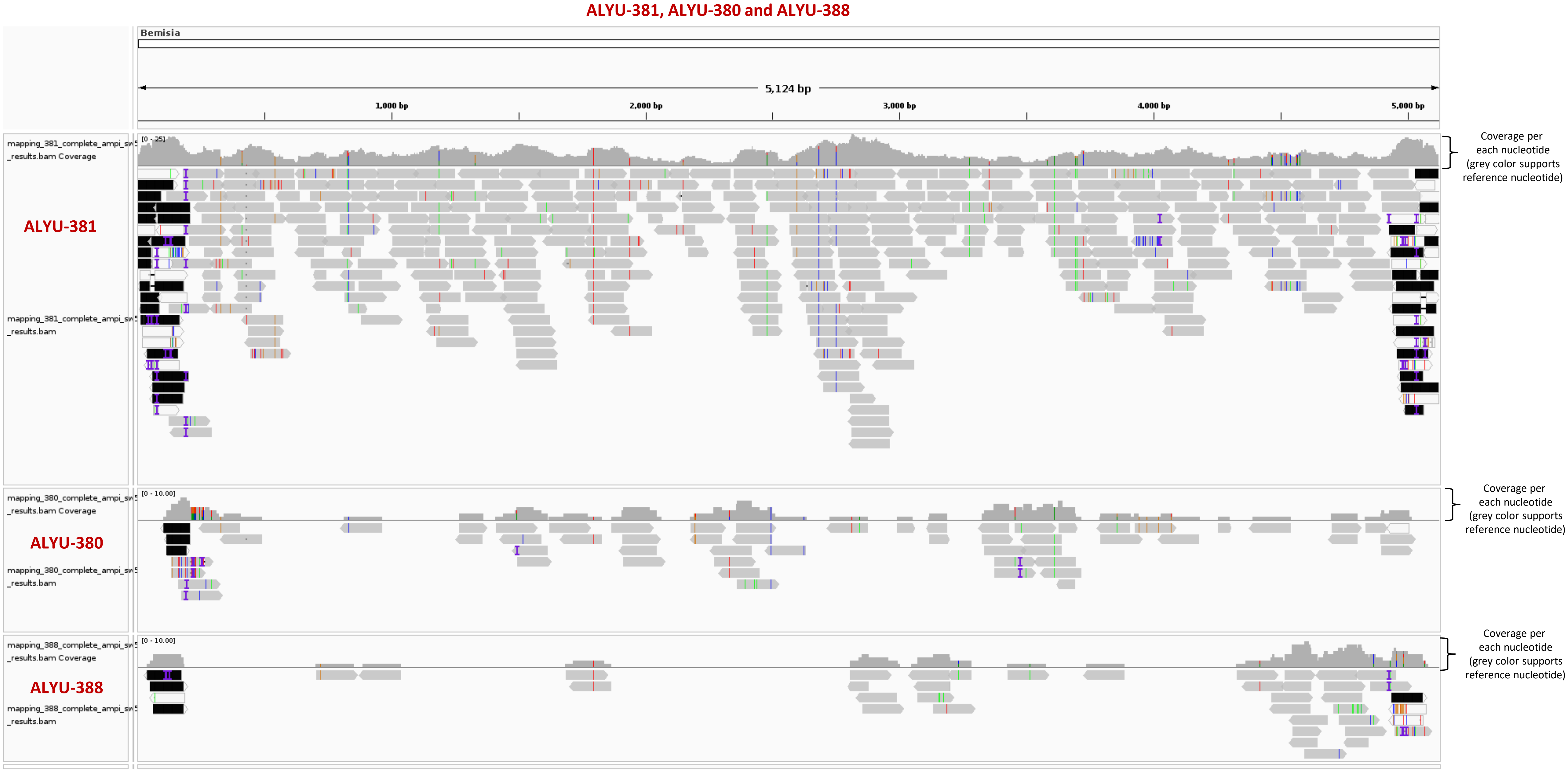

ALYU-387, ALYU-389, ALYU-384, ALYU-379, ALYU-386, ALYU-383, ALYU-385 and ALYU-382

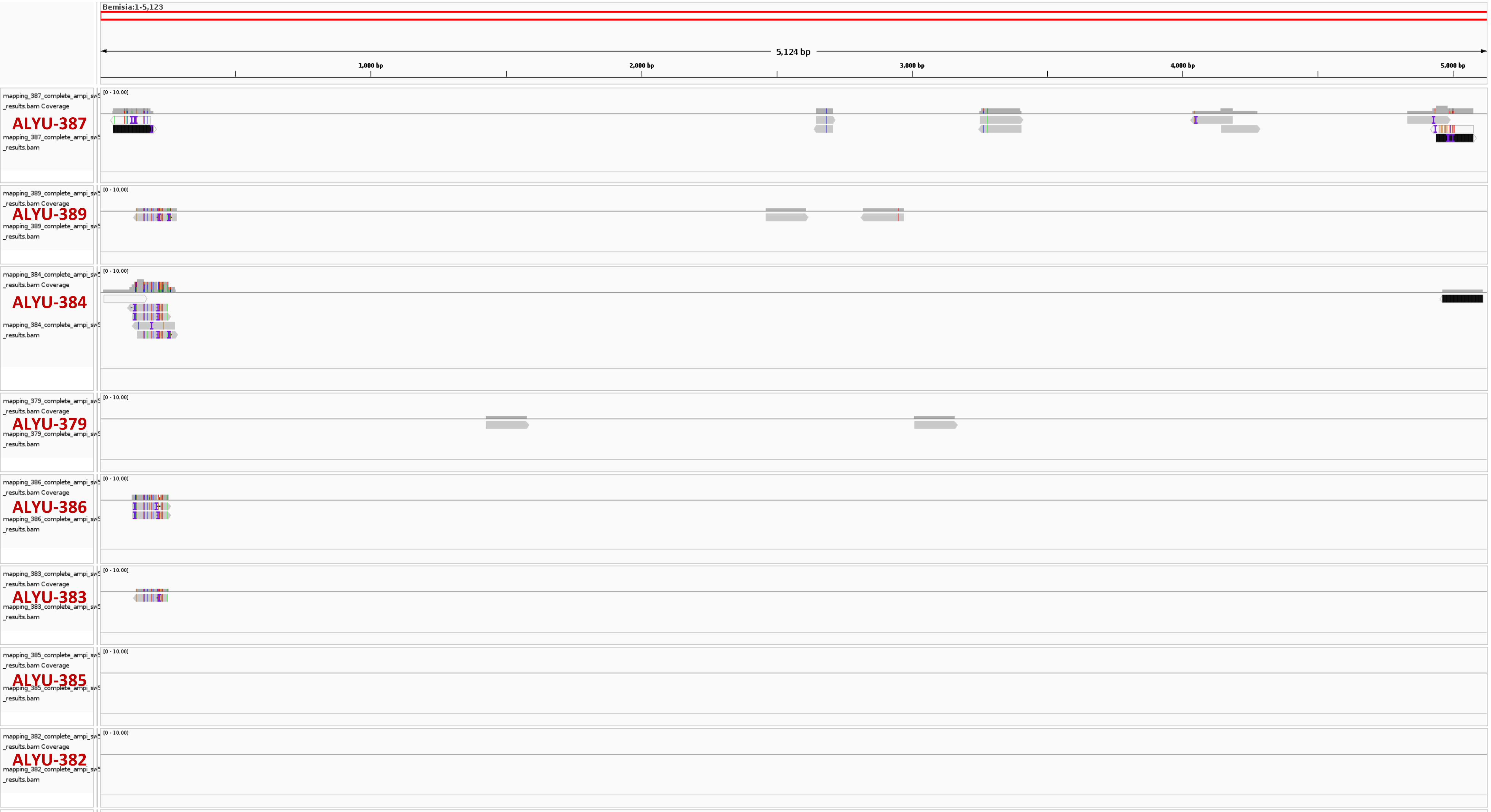

Fig. S3C. IGV visualization of the Illumina 150 nt reads from ALYU-381 library mapped to a consensus genome sequence of the ALYU-381 variants of Bemisia tabaci ambidenosvirus (BtaDV) reconstructed by mapping the ALYU-381 reads to the BtaDV ALYU-390 reference sequence.

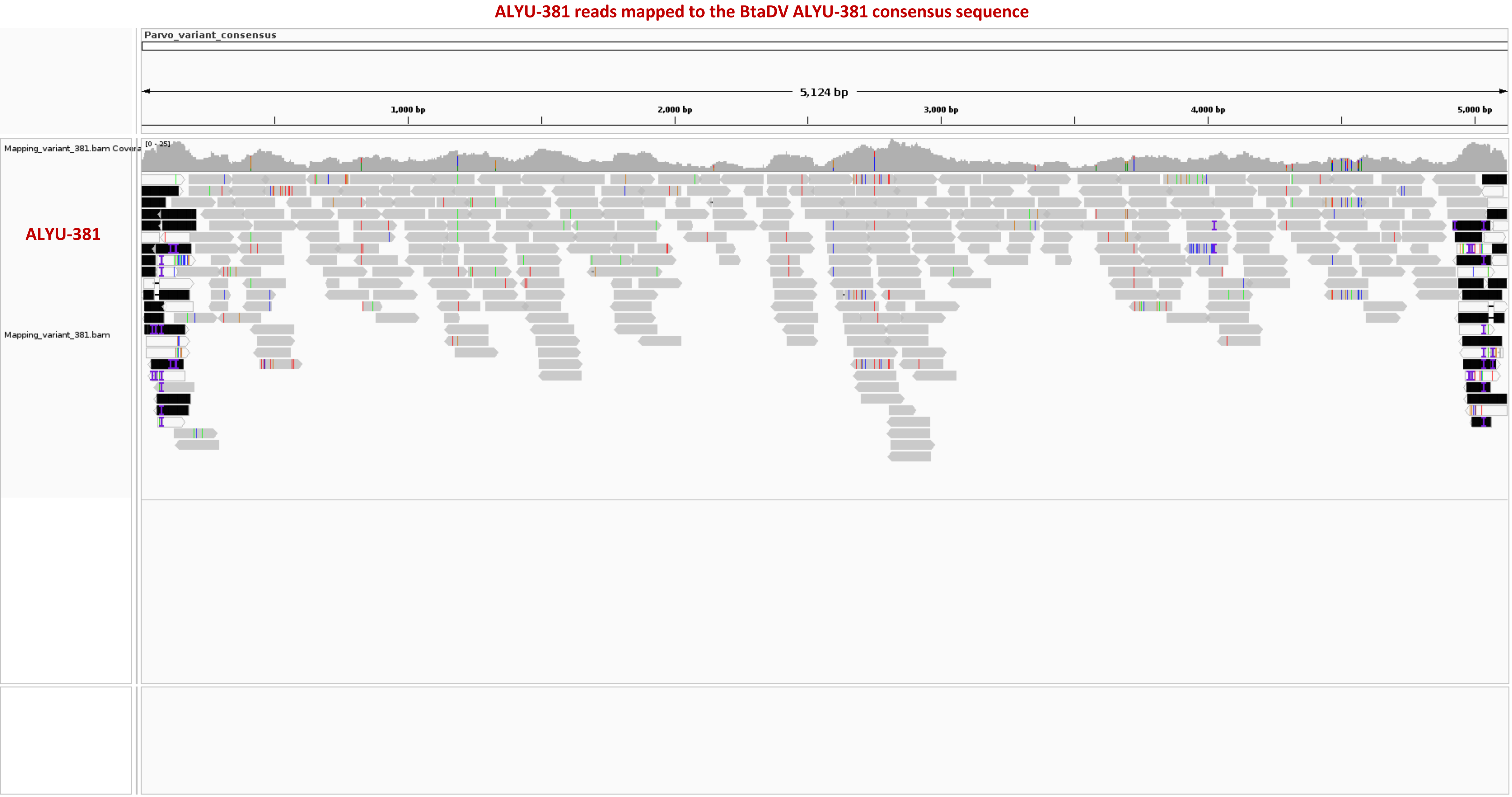

Supplement: Supplementary file 1 [file pathogens-14-00714-s001.zip › Supplementary Figures final.pdf]
